# Association between ambient air pollutants and upper respiratory tract infection and pneumonia disease burden in Thailand from 2000 to 2022: a high frequency ecological analysis

**DOI:** 10.1186/s12879-023-08185-0

**Published:** 2023-06-06

**Authors:** Esther Li Wen Choo, A. Janhavi, Joel Ruihan Koo, Steve H. L. Yim, Borame L Dickens, Jue Tao Lim

**Affiliations:** 1 Saw Swee Hock School of Public Health, National University of Singapore, Singapore, Singapore; 2 Department of Biological Sciences, National University of Singapore, Singapore, Singapore; 3 Lee Kong Chian School of Medicine, Nanyang Technological University, Singapore, Singapore; 4 Asian School of the Environment, Nanyang Technological University, Singapore, Singapore; 5 Earth Observatory of Singapore, Nanyang Technological University, Singapore, Singapore

**Keywords:** Upper respiratory tract infections, Pneumonia, Ambient air pollutants, Weather, Environmental epidemiology, Mixed data sampling methods

## Abstract

**Background:**

A pertinent risk factor of upper respiratory tract infections (URTIs) and pneumonia is the exposure to major ambient air pollutants, with short term exposures to different air pollutants being shown to exacerbate several respiratory conditions.

**Methods:**

Here, using disease surveillance data comprising of reported disease case counts at the province level, high frequency ambient air pollutant and climate data in Thailand, we delineated the association between ambient air pollution and URTI/Pneumonia burden in Thailand from 2000 – 2022. We developed mixed-data sampling methods and estimation strategies to account for the high frequency nature of ambient air pollutant concentration data. This was used to evaluate the effects past concentrations of fine particulate matter (PM_2.5_), sulphur dioxide (SO_2_), and carbon monoxide (CO) and the number of disease case count, after controlling for the confounding meteorological and disease factors.

**Results:**

Across provinces, we found that past increases in CO, SO_2,_ and PM_2.5_ concentration were associated to changes in URTI and pneumonia case counts, but the direction of their association mixed. The contributive burden of past ambient air pollutants on contemporaneous disease burden was also found to be larger than meteorological factors, and comparable to that of disease related factors.

**Conclusions:**

By developing a novel statistical methodology, we prevented subjective variable selection and discretization bias to detect associations, and provided a robust estimate on the effect of ambient air pollutants on URTI and pneumonia burden over a large spatial scale.

**Supplementary Information:**

The online version contains supplementary material available at 10.1186/s12879-023-08185-0.

## Introduction

Upper Respiratory Tract Infections (URTIs), such as pneumonia and influenza, are usually characterised by irritation and swelling of the upper airways. They are caused by a variety of bacteria and viruses and the infection can vary from a mild cold to life-threatening pneumonia [1]. Being one of the most common diseases, the global burden of URTI in 2019 is estimated to be 17.2 billion [2]. Although a majority of URTIs are quite harmless, the estimated economic burden of non-influenza related viral URTIs in the United States alone is estimated to be 22.5 billion USD [3] and an estimated 2 billion USD is spent on over-the-counter treatments for URTIs [4].

A pertinent risk factor of URTIs is the exposure to major ambient air pollutants, such as carbon monoxide (CO), nitrogen dioxide (NO_2_), sulphur dioxide (SO_2_), particulate matter (PM) and ozone (O_3_) [5]. Short term exposures to different air pollutants have shown to have detrimental immunological effects, and can exacerbate several respiratory conditions, including URTIs [6–10]. Whereas long-term exposures to different air pollutants, accounted through averaging pollutant measurements of past years and looking across large spatial scales, also found similar relationships between URTIs and ambient air pollutants [11]. Apart from linear relationships, previous research has also found non-linear effects of PM on URTI relative risk [12, 13].

Environmental variables, such as ambient air pollutant and climate measurements, can be collected almost instantaneously, but disease case counts are collated on daily or lower frequencies due to inherent limitations of disease surveillance systems. Exposures to environmental variables are also continuously occurring in nature, while the exposure and generation time to disease is disjointed. The differences in sampling frequencies and the relationships between exposure and response variables therefore complicates the analysis of environmental variables with that of disease case counts.

As evidenced from previous work looking at disease cases and ambient air pollution, a common way to account for variables of different frequencies comprise of averaging the values of higher frequency variables to that of a lower frequency [11]. In the case of time series analysis, to further account for the different ways each lagged covariate influence disease case counts, multiple lags of the covariate of interest may be included into a regression specification [11]. Crucially, the former leads to the loss of valuable information within the higher frequency variable, and the latter may lead to overfitting and errors in estimation due to the inclusion of many correlated parameters. It is thus important to bridge this discrepancy in data sampling frequencies in some optimal way.

To circumvent overfitting and model over-specification, mixed data sampling (MIDAS) models deal with resolving data collected at different frequencies by taking the weighting scheme of the higher frequency variable as unknown and parameterized by a function to be estimated. It also has several attractive properties, which include (1) model parsimony – requiring a small number of parameters to incorporate a large number of high frequency lagged observations and (2) flexibility – using a flexible, data driven weighing scheme for high frequency variables which reduces the possibility for omitted variable bias and model misspecification.

When frequencies are resolved among variables, a central related problem is inferring the exposure–response functions or curves. In literature, this translates to (1) choosing the number of lags in time-series models and (2) the number of terms to place into splines or kernels within non-linear regression models [14] through diagnostic tests [15], model selection criteria [16] and prior epidemiological information. Centrally, these measures tradeoff between a modeller’s prior on disease and being agnostic about assumptions on disease transmission dynamics – due to them comparing the discrepancy between the estimated model and observed data. MIDAS incorporates the full range of observed exposures as explanatory variables into the regression specification and allows the estimation procedure to decide appropriately on the temporal importance of exposures, thereby circumventing either problem.

In this paper, using disease surveillance and ambient air pollutant data collected for 2 decades, we employ MIDAS to understand the short to medium exposure–response between URTIs, pneumonia and major ambient air pollutants. We compared the ability of MIDAS to fit to disease case data versus standard time series models, map the subsequent exposure–response curves between URTIs, pneumonia and major ambient air pollutants across all provinces. By exploiting the large contiguous scale on which the data is collected, we also examined how these exposure-responses are heterogenous.

## Methods

### Disease surveillance data

Disease surveillance data was obtained from Thailand’s Ministry of Public Health (MOPH) disease surveillance system [17] where we obtained reported disease case counts from 2000 to 2022 at the province level. Suspected cases of pneumonia and influenza in provincial public health offices, hospitals and all health stations were reported through the disease surveillance system to the Bureau of Epidemiology, MOPH. Suspected cases were defined as patients who had symptoms matching the clinical criteria. Private hospitals were not covered by the surveillance system. Diagnoses were recorded using 10th revision of the International Classification of Disease (ICD) codes. As Bueng Khan was split from Nong Khai in 2011 to form a separate province, we have merged disease case counts from Bueng Khan back to Nong Khai from 2011 onwards, to allow consistent analysis over the timeframe of the dataset.

### Demographic data

Data on annual population size for each province from 2000 to 2022 was obtained from the Official Statistics Registration Systems of Thailand [18]. Similarly, as Bueng Khan was split from Nong Khai in 2011 to form a separate province, we have merged the population numbers from Bueng Khan back to Nong Khai from 2011 onwards, to allow consistent analysis over the timeframe of the dataset.

### Climate data

Climate data was obtained from ERA5, published by the European Centre for Medium-Range Weather Forecasts [19]. Each data point covers a 30 km grid, which we spatially averaged across each province. Mean, median and maximum of total precipitation, vegetation index, air temperature at 2 m and dew point temperature at 2 m was collected. Relative humidity and average humidity were calculated using standard formula.

### Ambient air pollutants data

Ambient air pollutant data was obtained from NASA's Goddard Earth Sciences Data and Information Services Center, GES DISC. The 1-Hourly CO Column Burden, CO Surface Concentration, and 1-Hourly Aerosol diagnostics were obtained from Global Modeling And Assimilation Office [20] to derive PM_2.5_ surface concentration. The surface model layer of the 3d 3-Hourly Aerosol Mixing Ratio was obtained from Global Modelling and Assimilation Office [21] to derive PM_1_ and PM_10_ surface concentration. These datasets are a part of Modern-Era Retrospective Analysis for Research and Applications, version 2 (MERRA 2), which is a reanalysis of the modern satellite data, and is produced by NASA’s Global Modelling and Assimilation Office (GMAO) [22].

### Mixed data sampling (MIDAS)

Consider reported disease case counts ${y}_{\tau +1}$ which is observed at the discrete time point between $\tau$ and $\tau +1$ and supposed that we have additional information arising from a set of *V* predictors ${{\varvec{x}}}_{\tau }^{(m)}=( {{\varvec{x}}}_{1}^{\left(m\right)}, {{\varvec{x}}}_{\tau }^{\left(m\right)},\dots , {{\varvec{x}}}_{V}^{(m)})$ which are observed *m* times between $\tau$ and $\tau +1$. The variables ${y}_{\tau +1}$ and ${{\varvec{x}}}_{\tau }^{(m)}$ can thus be said to be observed at different frequencies. The traditional and simplest manner of dealing with mixed frequency data would be averaging the high frequency predictors to the same frequency of the dependent variable ${y}_{\tau +1}$. However, this approach may result in omitted variable bias and model mis-specification if the true weighting scheme is not a simple average [11]. The mixed frequency data sampling (MIDAS) framework provides an alternative estimator that can account for different weighting schemes in the high frequency predictors. Specifically, the approach plugs in the high frequency lagged terms of predictors ${{\varvec{x}}}_{\tau }^{(m)}$ in a regression for the low frequency dependent variable ${y}_{\tau +1}$ as follows:

$${y}_{\tau +1}=\alpha +\sum_{j=0}^{{p}_{y}-1}{p}_{j+1}{y}_{\tau -j}+\sum_{j=0}^{{p}_{z}-1}\sum_{s=1}^{S}{\gamma}_{j+1 ,s}^\prime{z}_{\tau -j,s}+\sum_{v=1}^{V}{\beta }_{v}B\left({L}^\frac{1}{m};{{\varvec{\theta}}}_{v}\right){x}_{\tau ,v }^{(m)}+\epsilon$$

where ${y}_{\tau -j}$ denotes past disease case counts for a maximum of ${p}_{y}-1$ lags. We can additionally place $S$ exogenous variables ${z}_{\tau -j,s}$ recorded at time $\tau -j$ with the same frequency as $y$ for a maximum of ${p}_{z}-1$ lags. The high frequency predictors ${x}_{\tau ,v }^{(m)}$ are shrunk to the same frequency as $y$ and $z$ by a polynomial term $B$ to be defined later. $\alpha$ is an intercept term, ${p}_{y}$ autoregressive terms, ${\gamma }_{j+1 ,s}^{^{\prime}}$ coefficients denoting the effect of past ${z}_{s}$ on $y$ and ${\beta }_{v}$ are coefficients which capture the overall effect of $B\left({L}^\frac{1}{m};{{\varvec{\theta}}}_{v}\right)$ on ${y}_{\tau +1}$. We further assume i.i.d and normally distributed errors, with mean zero and finite variance ${\sigma }_{\epsilon }^{2}$:

$${\epsilon \sim N(0,\sigma }_{\epsilon }^{2})$$

The polynomial term is given by:

$$B\left({L}^\frac{1}{m};{{\varvec{\theta}}}_{v}\right)= \sum_{k=0}^{K-1}B\left(k,{{\varvec{\theta}}}_{k}\right){L}^{k/m}$$

where ${L}^{k/m}$ is a lag operator such that ${L}^{1/m}={x}_{\tau -1/m}^{(m)}.{\varvec{\theta}}_{v}$ are parameters which provide the shape of the polynomial term. We use the normalized beta probability density function on the polynomial term $B\left(k,{{\varvec{\theta}}}_{v}\right):$

$$B\left(k,{{\varvec{\theta}}}_{v}\right)= \frac{{x}_{k}^{{\gamma }_{1}-1}{\left(1-{x}_{k}\right)}^{{\gamma }_{2}-1}}{{\sum }_{k=1}^{K-1}{x}_{k}^{{\gamma }_{1}-1}{\left(1-{x}_{k}\right)}^{{\gamma }_{2}-1}}$$

where ${x}_{k}=(k-1)/(K-2)$. The beta polynomial was used as it only requires two parameters ${{\varvec{\theta}}}_{v}\in \{{\gamma }_{1},{\gamma }_{2}\}$ to specify and generate a large variety of weighting shapes [23]. In particular, further restrictions such as {${\gamma }_{1}$ = 1*,*${\gamma }_{2}$} or {1 + ${\gamma }_{1}$*,* 1 +${\gamma }_{1}$  + ${\gamma }_{2}$} allow the weighting structure to form only downward sloping and hump-shaped weights respectively, which were also explored.

### Implementing the MIDAS specification

In order to implement MIDAS, we consider the Bayesian paradigm and estimate parameters based on Markov chain Monte Carlo (MCMC) methods, which alleviates certain issues in frequentist parameter estimation. First, uncertainty and standard errors can be characterized easily using credible intervals. Second, the MCMC approach facilitates easy modification to allow shrinkage and sparsity, thereby allowing unimportant variables to be shrunk closer to zero.

Briefly, we first placed diffuse, conjugate priors on both the parameters of the observation equation ${\{p}_{j+1},{\gamma }_{j+1 ,s}^{^{\prime}},{\beta }_{v}\}$ and error variance ${\sigma }_{\epsilon }^{2}$. Gibbs sampling was used as conditional posterior distributions for these parameters were well defined. We also modified the baseline procedure of sampling parameters in the observation equation to induce shrinkage, through incorporating the structure of Bayesian Lasso [24]. This comprised changing the prior for the variance of the error term, to enable more probability weight to be placed on 0 for the parameters of the observation equation.

Following Ghysels [23], a Gamma distribution is placed as a prior on the parameters of the polynomial terms {${\gamma }_{1},{\gamma }_{2}\}$ with both shape and scale parameters being 1, which amounts to a flat weighting scheme that puts equal weight on the high frequency data. The polynomial terms were sampled using a random walk Metropolis-in-Gibbs step. The Metropolis step is an accept-reject step which takes a candidate draw from some proposal distribution, with acceptance for that draw is given by a probability that depends on the likelihood, parameter’s prior distribution and the proposal density. In this case, we use the Gamma proposal distribution as a proposal density, as it corresponds to the functional form of the MIDAS weighting polynomial. Full details for the estimation strategy are outlined in the supplementary information.

### Assessing model performance

Convergence of MCMC chains was first assessed by visual inspection of trace plots and Gewecke convergence diagnostic checks. Residual autocorrelation is computed for up to 20 week lags to ensure that the transmission dynamics are properly accounted for in each specification, across models. Quantile–quantile plots are used to see whether the specifications adequately account for the data structure. We additionally computed 4 other statistics on in-sample model fit, namely, the ${R}^{2}$ and in-sample mean-squared error to look at whether overall variations in data are captured, the adjusted ${R}^{2}$ to look at whether overall variations in data are captured while penalizing larger models. Lastly, the deviance information criterion [25] look at whether overall variations in data are captured while penalizing larger models with larger uncertainty in posterior distributions.

## Results

We first merged at the province level, with either monthly URTI or pneumonia case counts, climate confounders such as mean temperature total precipitation, absolute and relative humidity for the past 2 months, as well as ambient air pollutants for the past 40 days, recorded at the 6 hourly level (Table 1). MIDAS models were estimated with each ambient air pollutant taken separately in respective polynomial terms, while controlling for past climate measurements and past disease case counts for up to two months. We estimated (1) a baseline autoregressive model consisting of no MIDAS terms and only disease case counts and climate measurements for the past 2 months (2) MIDAS models with no variable shrinkage (3) MIDAS models with variable shrinkage. For (2), (3), we also explored separately the utility of placing no restrictions on MIDAS weights, downward sloping restrictions on MIDAS weights and hump-shaped restrictions on MIDAS weights. These help us understand whether constraints or the lack thereof in MIDAS weights would provide superior model performance compared to alternatives.

**Table 1:** Summary statistics for dependent variables of interest and covariates used for MIDAS specification

Variable (unit)	Mean	Range
Influenza Case Counts (Monthly)	102.13	(0, 13,214)
Pneumonia Case Counts (Monthly)	203.88	(0, 3144)
Absolute Humidity	26.78	(15.7, 40.98)
Relative Humidity (%)	76.36	(45.87, 91.68)
Total Precipitation (mm)	0.18	(0, 1.24)
Temperature (K)	300.08	(290.79, 307.65)
CO Surface Concentration (ppb)	172.19	(50.56, 1090.3)
SO2 Surface Concentration (mu/g)	5.07	(0.05, 59.52)
PM2.5 Surface Concentration (mu/g)	19.05	(1.51, 367.24)

### Impact of major ambient air pollutants on URTI and pneumonia

**Fig. 1:**
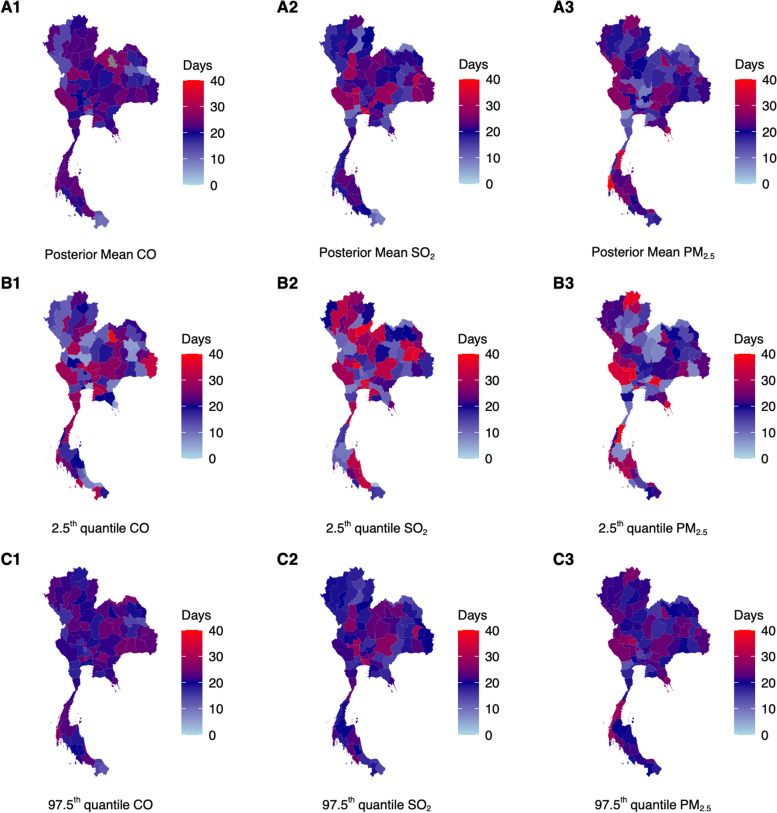
**A1** – **A3** Posterior mean estimates of duration where the highest MIDAS weights were placed on the importance of respective ambient air pollutant measurements for each province on influencing URTI disease case counts the following month. Darker red and blue shaded regions represent that ambient air pollutant measurements beyond 20 – 40 days are deemed more important in determining URTI disease case counts. **B1** – **B3** 2.5^th^ quantile value for MIDAS weights drawn from MCMC samples **C1** – **C3** 97.5^th^ quantile value for MIDAS weights drawn from MCMC samples

After adjustment for confounders, SO_2,_ PM_2.5_, and CO over a 40-day period were estimated to be positively associated to monthly URTI case counts in 41, 15 and 31 of 76 provinces, with their 95% credible intervals away from zero. In other provinces, ambient air pollutants were negatively associated to monthly URTI case counts. On average, greatest influence of ambient air pollutants were estimated to be around the 20^th^ day mark across provinces (Fig. 1A1-3), but the range of values differed greatly, where the 7 – 36, 6 – 41 and 7 – 40 day measurements for SO_2_, CO and PM_2.5_ (Fig. 1) were ascribed the greatest influence on case counts across provinces respectively. Exposure–response shapes were also highly heterogenous across provinces but were mainly hump-shaped across the 40-day period (See supplementary material).

**Fig. 2:**
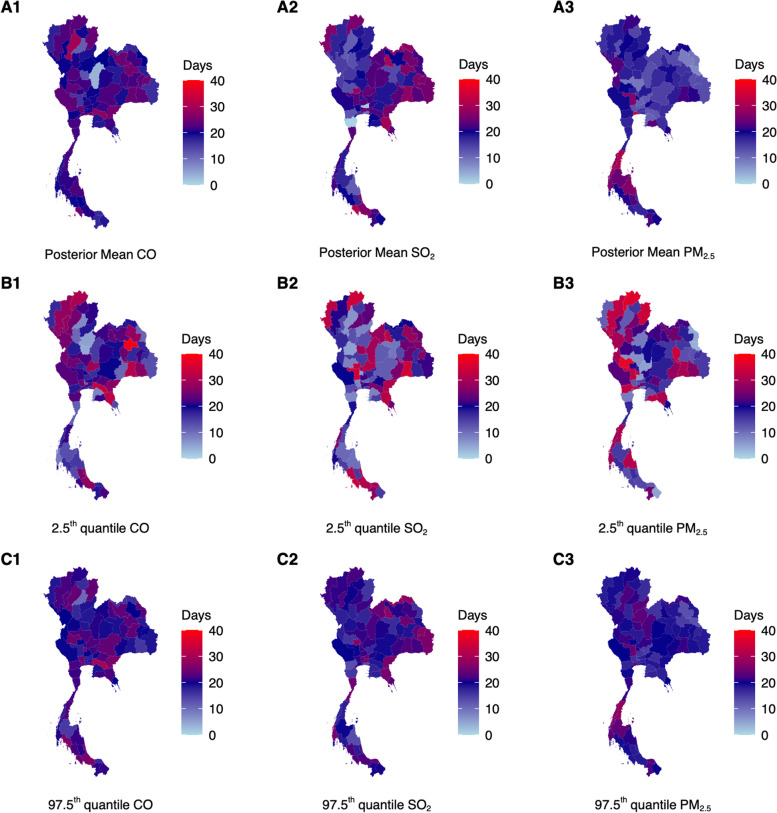
**A1** – **A3** Posterior mean estimates of duration where the highest MIDAS weights were placed on the importance of respective ambient air pollutant measurements for each province on influencing pneumonia disease case counts the following month. Darker red and blue shaded regions represent that ambient air pollutant measurements beyond 20 – 40 days are deemed more important in determining pneumonia disease case counts. **B1** – **B3** 2.5^th^ quantile value for MIDAS weights drawn from MCMC samples **C1** – **C3** 97.5^th^ quantile value for MIDAS weights drawn from MCMC samples

Whereas PM_2.5_, SO_2_ and CO over a 40-day period were positively associated to monthly pneumonia case counts in 15, 31 and 49 of 76 provinces (Fig. 2A1-3, B1-3, C1-3). Similar to URTI, we found on average that the greatest influence of ambient air pollutants were estimated to be in the 20^th^ day mark across provinces, but the range was noticeably narrower compared to case of URTIs, at the 0 – 32, 3 – 30 and 7 – 33 day mark for SO_2_, CO and PM_2.5_ respectively. Similarly, exposure–response shapes were highly heterogenous across provinces for ambient air pollutants and pneumonia case counts. These shapes were however mainly hump-shaped across the 40-day period (See supplementary material).

**Fig. 3:**
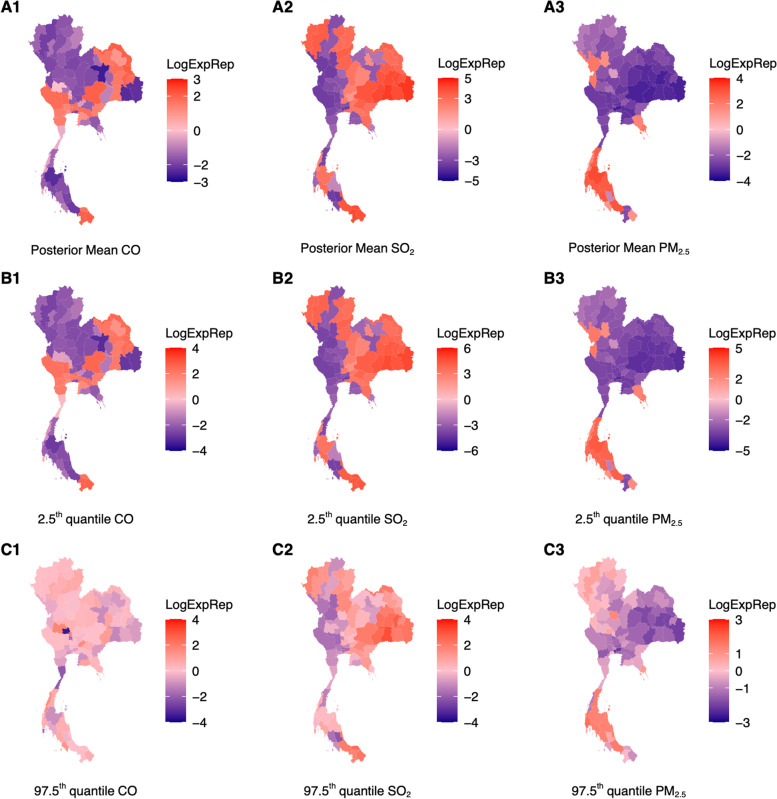
**A1** – **A3** Posterior mean estimates of mean exposure–response of respective ambient air pollutants over the past 40 day period on contemporaneous URTI disease case counts for a specific region. Red shades represent regions where a one unit increase in ambient air pollutant surface concentrations in the respective region for the past 40 days are associated to an increase in contemporaneous, monthly URTI disease case counts. **B1** – **B3** 2.5^th^ quantile value for exposure response drawn from MCMC samples of MIDAS weights **C1** – **C3** 97.5^th^ quantile value for exposure response drawn from MCMC samples of MIDAS weights

Controlling for all other variables, on average, a 1 μg/m^3^ rise in SO_2_ over the past 40 days would lead to an expected 1624.1 (Fig. 3, A2, B2, C2) increase in URTI case counts. However, an expected change of -15.1 (Fig. 3, A1, B1, C1) and -406.3 (Fig. 3, A3, B3, C3) in URTI case counts respectively are expected to occur with a 1 ppb rise in CO and 1 μg/m^3^ rise in PM_2.5_ surface concentrations over the past 40 days respectively across provinces.

**Fig. 4:**
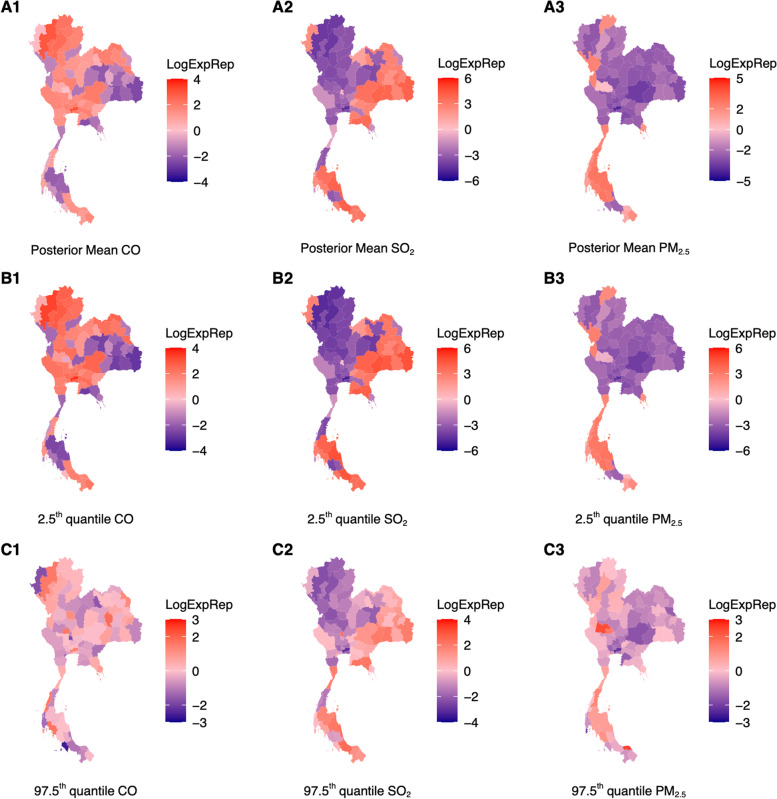
**A1** – **A3** Posterior mean estimates of mean exposure–response of respective ambient air pollutants over the past 40 day period on contemporaneous pneumonia case counts for a specific region. Red shades represent regions where a one unit increase in ambient air pollutant surface concentrations in the respective region for the past 40 days are associated to an increase in contemporaneous, monthly pneumonia case counts. **B1** – **B3** 2.5^th^ quantile value for exposure response drawn from MCMC samples **C1** – **C3** 97.5^th^ quantile value for exposure response drawn from MCMC samples

Whereas a 1 ppb rise in CO surface concentrations over 40 days would lead to an expected 39.3 change (Fig. 4, A1, B1, C1) in monthly pneumonia case counts, a 1 μg/m^3^ increase in SO_2_ and PM_2.5_ surface concentration would lead to an expected -943 (Fig. 4A2, B2, C2) and -401.8 (Fig. 4) change in monthly pneumonia case counts across provinces respectively. Comparing the range of posterior mean effect sizes and quantile values across provinces for major ambient air pollutants on monthly pneumonia and URTI case counts showed that associations between ambient air pollutants on pneumonia were far more variable compared to that of URTI (Figs. 3 and 4).

### Contributive effects and burden of climate, past disease and ambient air pollutants on URTI and pneumonia

Across provinces, controlling for past disease case counts and the effects of ambient air pollutants, increases in climate measurements the past two months have mixed associations to monthly URTI and pneumonia case counts. In particular, an increase in mean absolute humidity over the past 1 and 2 month was associated to decreases in contemporaneous URTI case counts in 22 and 9 of 76 provinces respectively. Whereas an increase in mean relative humidity 1 and 2 months prior was associated to decreases in contemporaneous URTI case counts in only 2 and 1 provinces respectively. However, in 8 provinces, increases in mean relative humidity 1 month prior was associated to increases in contemporaneous URTI case counts. In all provinces, higher temperature 1 and 2 months prior were associated to decreases in URTI in all provinces. Lastly, increases in total precipitation the past month were associated to increases and decreases in contemporaneous conjunctivitis case counts in 21 and 10 provinces respectively.

Similarly, increases in temperature for the past 1 and 2 months were associated to decreases in pneumonia case counts in 74 and 73 provinces respectively. Increases in absolute humidity the past 1 and 2 months were associated to decreases in contemporaneous pneumonia case counts in 16 and 11 provinces respectively. Whereas in only 3 provinces, changes in relative humidity were associated to any increase or decrease in contemporaneous pneumonia case counts.

**Fig. 5:**
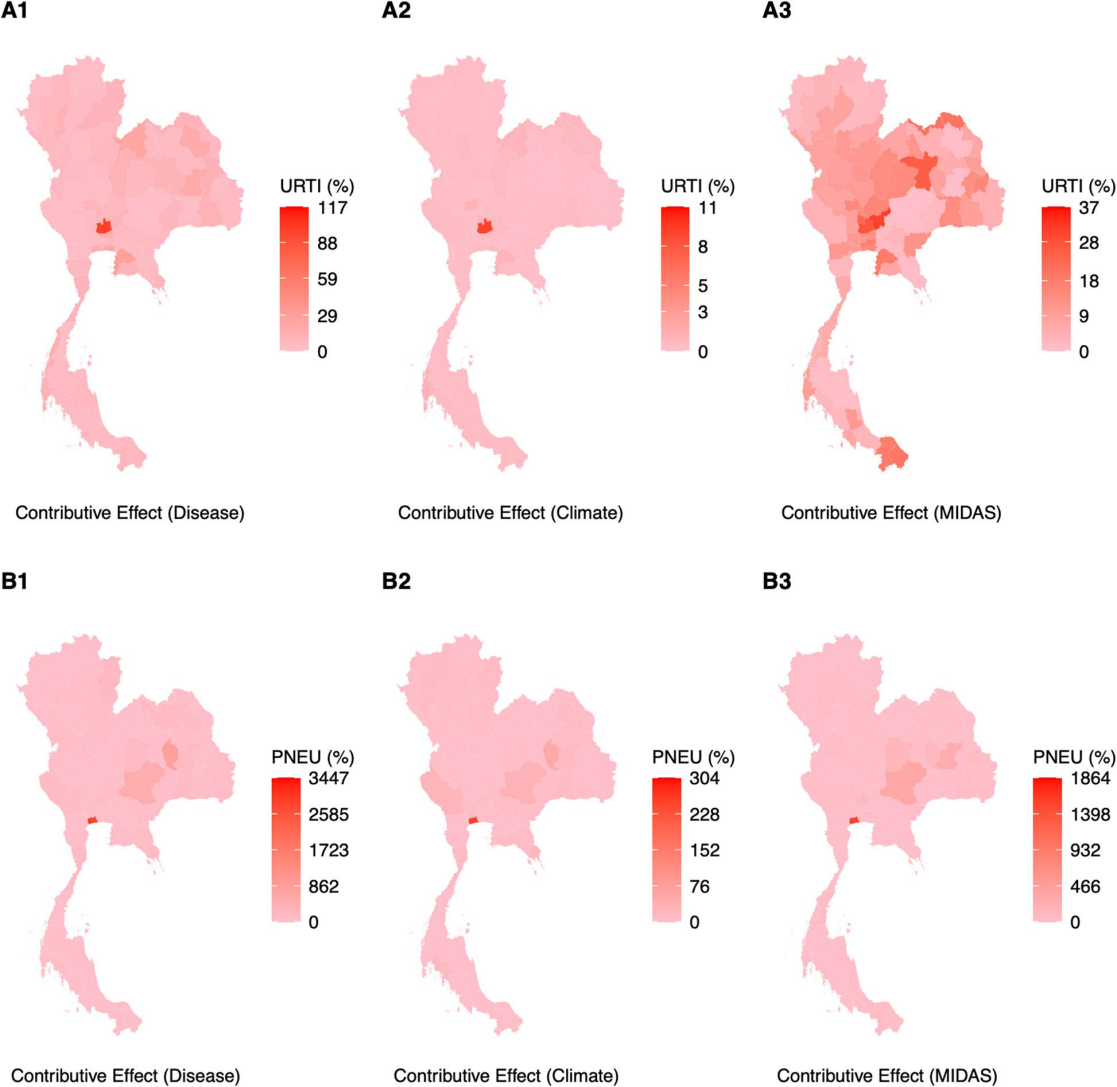
**A1** – **A3** Average absolute contribution of disease case counts the past two months, climate measurements the past two months (i.e. temperature, total precipitation, absolute humidity, relative humidity) as well as ambient air pollutants as weighted under MIDAS over the past 40 day period on predicted contemporaneous URTI case counts **B1** – **B3** Average absolute contribution of disease case counts the past two months, climate measurements the past two months (i.e. temperature, total precipitation, absolute humidity, relative humidity) as well as ambient air pollutants as weighted under MIDAS over the past 40 day period on predicted contemporaneous pneumonia case counts

Comparing the contributive effect of past disease case counts, ambient air pollutants and climate on disease case counts suggests that the mean impact of past climate variables across provinces on contemporaneous URTI case counts is minor, ranging from -0.7 – 0.17% (Fig. 5A2), while the mean impact of past climate variables across provinces on pneumonia case counts is higher at -27.8% – 15.8% (Fig. 5B2). Whereas the impact of ambient air pollutants were greater in influencing contemporaneous URTI and pneumonia case counts at -14.8% – 4.96% (Fig. 5A1, B1) and -79.78% – 107.5% (Fig. 5A3, B3) respectively. Lastly, the impact of past case counts in influencing contemporaneous URTI and pneumonia case counts at -17.4% – -0.16% (Fig. 5A1, B1) and -118.4% – 17.9% (Fig. 5A3, B3) respectively. For the case of pneumonia, wide estimates for contributive effect were partially attributed to the timepoints where case counts were low in some provinces (See supplementary material) and model predictions were pulled in separate directions by case count and ambient air pollutant measurements.

### Effects of normalizing disease case counts by annual population 

All analysis was repeated using case counts normalized by population size. That is, disease case counts per 100,000 individuals was used as the dependent variable of interest for the analysis. Similar to the analysis using raw disease case counts as the dependent variable of interest, we found that CO and SO_2_ surface concentrations the past 40 days were more likely to be positive associated to increases in disease case counts for the contemporaneous month. Mean duration where the pollutants were taken to be most important by MIDAS weights were also around the 18 – 21th day mark. The contributive burdens of different confounders on disease case counts were also similarly found to be higher on past disease case counts and ambient air pollutant measurements, rather than climate (See supplementary material).

### Effect of higher frequency data and variable shrinkage on model performance 

Quantile–quantile plots showed that models considered, with or without MIDAS terms, in general could capture distributional characteristics of disease case counts well. Visual diagnostics of trace-plots also showed that MCMC procedures have converged for all parameters in each model.

Overall, we found that incorporating MIDAS weights provided overall improvements over the baseline model where no MIDAS weights were incorporated, with overall lower model mean absolute errors, deviance information criterion and coefficients of determination. Furthermore, the model incorporating no restrictions on MIDAS weights worked better compared to models incorporating downward sloping or hump-shaped weights. These models had better model fit as demonstrated by the mean-squared error as well as the deviance information criterion in 40.8% and 37.5% of provinces across all models considered. Imposing restrictions for MIDAS weights to be downward sloping weights also performed reasonably well, as demonstrated by their comparatively better mean-squared error as well as the deviance information criterion in 34.9% and 40.8% of provinces across all models considered.

Incorporating variable shrinkage to the overall regression specification also improved model performance in terms of mean-squared error, unadjusted and adjusted coefficients of determination as well as the deviance information criterion in almost all provinces. These tests are reported in the supplementary information section.

Given these diagnostic tests, the model incorporating shrinkage in regression parameters, as well as no restrictions on MIDAS polynomials was taken as representative to infer exposure–response curves between ambient air pollutants and URTI/pneumonia case counts across all provinces.

## Discussion

Heightened exposure to ambient air pollutants the past 40 days was shown to have mixed associations with contemporaneous monthly URTI and pneumonia case counts across provinces (Figs. 3 and 4). Specifically, heightened SO_2_ and CO concentrations the past 40 days were shown to have a positive association with URTI and pneumonia case counts in around half the regions, with or without controlling for population size, and controlling for past disease case counts and climate data (Figs. 3 and 4). These associations also had credible intervals which crossed the zero bound.

SO_2_ and PM_2.5_ have been shown to have negative effects on the respiratory system and exacerbate breathing problem [26, 27]. In mouse models, SO_2_ exposure was observed to cause DNA damage and oxidative damage in the lungs [28, 29]. PM_2.5_ also leads to inflammation and cell damage, through the production of free radicals and reactive oxygen species [30]. The potential excess inflammation and respiratory system damage from PM could increase one’s susceptibility and severity to pneumonia and URTI [31, 32]. However, we found that the association of past SO_2_ and PM_2.5_ exposure on disease case counts were mixed across different provinces. Although SO_2_ is mostly known as an air pollutant, several studies suggested that SO2 had beneficial impacts on mammals, including anti-inflammatory effects [33–35]. Low levels of SO_2_ was suggested to have a protective effect on bacteria-induced pulmonary infections [36]. Furthermore, a study in mice found that low doses of SO_2_ exposure reduced the amount of pneumonia [37].

CO has been shown to be toxic on a systemic level [38]. In excess, CO causes hypoxia by decreasing the oxygen carrying capacity and O_2_ release in tissues [39]. Excessive CO can also lead to systemic immunological or inflammatory damage [39, 40]. While there is no clear direct biological mechanism between CO exposure and pneumonia, CO is a product of incomplete combustion [41]. Hence, CO might be a marker for other harmful inflammatory combustion products such as soot, which potentially explains the positive association found between CO and pneumonia [42]. However, an inverse association was also found in some provinces. While CO is toxic at high levels, low levels of CO have been found to possess anti-inflammatory effects and have been suggested for therapeutic use [38]. Low-dose CO could reduce lung inflammation and possibly decrease one’s susceptibility to pneumonia, resulting in the mixed associations observed [43].

In contrast, there is a stronger consensus in literature that PM_2.5_ has a positive relationship with URTI and pneumonia [44, 45]. Exposure to heightened PM_2.5_ can cause the development and progression of acute and chronic lung diseases, such as tracheal and pulmonary inflammation [46, 47]. In outpatient, emergency, and hospitalization-related data on respiratory infections showed that PM_2.5_ exposure was positively associated with the increased respiratory infections. Other studies showed that PM_2.5_ exposure was positively correlated with outpatient visits for upper respiratory tract infection [44]. While in certain provinces, especially in highly urbanized central Thailand (Fig. 3A1, 4A1) our analysis demonstrated a positive exposure–response between heightened PM_2.5_ concentrations and URTI/Pneumonia case counts, in other provinces we estimated converse associations between PM_2.5_ concentrations and URTI/Pneumonia case counts. To discern the reason for the difference in direction and effect sizes for associations between SO_2_ and PM_2.5_ and URTI/pneumonia case counts_,_ the surface concentrations of SO_2_ and PM_2.5_ across the time period over the provinces could be compared to the National Ambient Air Quality Standards established by the United States Environmental Protection Agency (EPA) [48]. SO_2_ and PM_2.5_ levels that are consistently lower than the EPA requirements were likely to have less or varying effects on URTI and pneumonia case counts.

Lastly, past disease case counts were found to have a significant contributive effect on disease case count. This is unsurprising as case counts tend to co-move. Additionally, we found that the effects of weather had little contribution on URTI and pneumonia case counts. This is perhaps due to the lack of large variations in weather in our study setting, which has a tropical climate. This is in contrast to climates with stark variations in weather, where colder months would lead to physiological and behavioural changes, such as spending more time indoors in poorly ventilated spaces, which would increase respiratory disease transmission risk [49, 50]. This changing behaviour and the resultant changing transmission risk would not be observed in tropical climates, explaining the lack of contribution of weather variables to URTI and pneumonia cases.

Our proposed MIDAS methodology has several strengths, it enables us to incorporate higher frequency ambient air pollutant measurements to infer their respective associations with future disease burden. This is conducted by first defining a flexible specification where the importance of each ambient air pollutant's lagged high frequency observations are decided by data, rather than taking a simple average to aggregate these variables to the same frequency as disease case counts. This prevents subjective lag selection in a model specification to detect associations [51–53] as all past pollutant measurements can be placed in a model, with their importance post-hoc decided. Furthermore, having a flexible weighting scheme reduces discretization bias due to simple averaging and increases the statistical efficiency of the model. Empirically, this is demonstrated from overall better model fit to observed data using the MIDAS framework across all provinces (See supplementary material).

Furthermore, while we used data over a large spatial scale, collected over a long period to delineate to historical impact of air pollutant concentrations on overall ambient air pollutant burden, the air pollutant concentrations in the study could not be representative of individual exposures, and the ecological design may cause ecological fallacy. Subgroup analyses by age and sex were not undertaken and should be considered in future studies. Also, while a primary strength of this study is the delineation of association on the provincial rather than national scale, conducting the analysis with national-level case counts could produce a generalized insight into the nationwide association of pneumonia and URTI with air pollutant exposure. Results could however be biased by spatial confounding. Unmeasured variables may be potentially confounded with the exposures of interest, such as income levels. Future directions could also comprise using more structured penalties, such as the group or fused LASSO [54, 55] to shrink MIDAS terms in a more informative manner, as well as consider MIDAS as a means to forecast instead of explain disease burden. National level analyses could also be undertaken, when additional spatial covariates are measured to adjust for unobserved confounding.

## Conclusion

In summary, we developed a novel statistical methodology to delineate the impacts of air pollutant concentrations on disease burden. Over a large spatial region, we found mixed but generally positive associations between increased O_3,_ SO_2,_ and PM_2.5_ concentrations on URTI and pneumonia case counts.

## Supplementary Information

**Additional file 1.**

## Data Availability

All disease surveillance data used to reproduce the results can be obtained at http://doe.moph.go.th/surdata/index.php.
