# Supplementary material for: Association between ambient air pollutants and upper respiratory tract infection and pneumonia disease burden in Thailand from 2000 to 2022: a high frequency ecological analysis
Source: BMC Infect Dis. 2023 Jun 6;23:379. doi: 10.1186/s12879-023-08185-0 (PMC10242813; doi:10.1186/s12879-023-08185-0)
Supplement: Supplementary file 1 — Additional file 1. [file 12879_2023_8185_MOESM1_ESM.pdf]

# Supplementary Information

November 1, 2022

# Contents


**1 Mixed data sampling (MIDAS) 1**
- 1.1 Model specification . . . 1
- 1.2 Implementing the baseline MIDAS specification . . . 2
- 1.3 Shrinkage in the observation equation . . . 4
- 1.4 Implementation of observation equation shrinkage through Bayesian LASSO . . . 5

**2 Results using disease case counts normalized by population size 6**

**3 Model assessment metrics with unnormalized case counts as dependent variables 11**

**4 Model assessment metrics for case counts per 100,000 19**

**5 Coefficient output for Bayesian MIDAS-LASSO per province 27**
- 5.1 Coefficient output for pneumonia case counts per 100,000 as the dependent variable 27
- 5.2 Coefficient output for URTI case counts per 100,000 as the dependent variable . . . 53
- 5.3 Coefficient output for pneumonia case counts as the dependent variable . . . 79
- 5.4 Coefficient output for URTI case counts as the dependent variable . . . 105


# 1 Mixed data sampling (MIDAS)

## 1.1 Model specification

Consider reported disease case count $y_{\tau+1}$ which is observed at the discrete time point between $\tau+1$ and $\tau$ and supposed that we have additional information arising from a set of $V$ predictors $\mathbf{x}_{\tau}^{(m)} = (x_{1,\tau}^{(m)}, \ldots, x_{V,\tau}^{(m)})$ which are observed $m$ times between $\tau$ and $\tau-1$. The variables $y_{\tau+1}$ and $\mathbf{x}_{\tau}^{(m)}$ can thus be said to be observed at different frequencies. The traditional and simplest manner of dealing with mixed frequency data would be averaging the high frequency predictors to the same frequency of the dependent variable $y_{\tau+1}$. However, this approach may result in omitted variable bias and model mis-specification if the true weighting scheme is not a simple average. The mixed frequency data sampling (MIDAS) framework provides an alternative estimator that can account for different weighting schemes in the high frequency predictors. Specifically, the approach plugs in the high-frequency lagged terms of predictors $\mathbf{x}_{\tau}^{(m)}$ in a regression for the low frequency dependent variable $y_{\tau+1}$ as follows:

$$y_{\tau+1} = \alpha + \sum_{j=0}^{p_y-1} p_{j+1} y_{\tau-j} + \sum_{j=0}^{p_z-1} \sum_{s=1}^{S} \gamma_{j+1,s}' z_{\tau-j,s} + \sum_{v=1}^{V} \beta_v B(L^{1/m}; \boldsymbol{\theta}_v) x_{\tau,v}^{(m)} + \epsilon_{\tau+1} \quad (1)$$

where $y_{\tau-j}$ denotes past disease case counts for a maximum of $p_y - 1$ lags. We can additionally place exogenous variables $z_{\tau-j,s}$ recorded at time $\tau - j$ with the same frequency as $y$ for a maximum of $p_z - 1$ lags and $S$ types. The high frequency predictors $x_{\tau,v}^{(m)}$ are shrunk to the same frequency as $y$ and $z$ by a polynomial term $B$ to be defined later. $\alpha$ is an intercept term, $p_{j+1}$ autoregressive terms, $\gamma'_{j+1,s}$ coefficients denoting the effect of past $z_s$ on $y$ and $\beta_v$ are coefficients which capture the overall effect of $B(L^{1/m};\boldsymbol{\theta}_v)x_{\tau,v}^{(m)}$ on $y_{\tau+1}$. We further assume i.i.d and normally distributed errors, with mean zero and finite variance $\sigma_\epsilon^2$:

$$\epsilon \sim N(0, \sigma_\epsilon^2) \tag{2}$$

The polynomial term is given by:

$$B(L^{1/m};\boldsymbol{\theta}_v) = \sum_{k=0}^{K-1} B(k;\boldsymbol{\theta}_v)L^{k/m} \tag{3}$$

Where $L^{k/m}$ is a lag operator such that $L^{1/m}x_\tau^{(m)} = x_{\tau-1/m}^{(m)}$. $\theta_v$ are parameters which provide the shape of the polynomial term. We use the normalized beta probability density function on the polynomial term $B(k;\boldsymbol{\theta}_v)$:

$$B(k;\boldsymbol{\theta}_v) = \frac{x_k^{\gamma_1-1}(1-x_k)^{\gamma_2-1}}{\sum_{k=1}^{K-1} x_k^{\gamma_1-1}(1-x_k)^{\gamma_2-1}} \tag{4}$$

where $x_k = (k-1)/(K-2)$. The beta polynomial was used as it only requires two parameters $\theta_v \in \{\gamma_1, \gamma_2\}$ to specify and generate a large variety of weighting shapes (Ghysels et al., 2007). In particular, further restrictions, such as $\{\gamma_1 = 1, \gamma_2\}$ or $\{1+\gamma_1, 1+\gamma_1+\gamma_2\}$ allow the weighting structure to form only downward sloping and hump-shaped weights.

Under this parameterization, by setting the dependent variable to any $h$ step ahead value, where $h \geq 1$, we can compress all MIDAS, autoregressive and exogenous variable parameter terms into the following matrix form by recasting (1):

$$y_{\tau+h} = \mathbf{Z}_\tau \mathbf{\Phi} + \epsilon_{\tau+1} \tag{5}$$

Where $\mathbf{\Phi} = \left(\alpha, p_1, p_2, \ldots, p_{p_y}, \gamma'_1, \gamma'_2, \ldots, \gamma'_{p_z}, \beta_1, \beta_2, \ldots, \beta_V\right)$ is a vector featuring $(1+p_y+p_z\times S+V)$ parameters and $\mathbf{Z}_\tau = \Big(1, y_\tau, \ldots, y_{\tau-p_y+1}, z_{\tau,1:S}, \ldots, z_{\tau-p_z+1,1:S}, B(L^{1/m};\boldsymbol{\theta}_v)x_{\tau,1}^{(m)}, \ldots, B(L^{1/m};\boldsymbol{\theta}_V)x_{\tau,V}^{(m)}\Big)$ is a matrix, comprising vectors of lagged observations of $y, z_{1:S}$ and the high frequency predictors $x$ compressed using the polynomial term $B(L^{1/m};\boldsymbol{\theta}_1)x_{\tau,v}^{(m)}$. We can obtain the $h$-step ahead direct forecast $y_{t+\tau}$ conditional on information available up to time $t$ by using (5):

$$\hat{y}_{\tau+h} = \hat{\mathbf{Z}}_\tau \hat{\mathbf{\Phi}} \tag{6}$$

## 1.2 Implementing the baseline MIDAS specification

In order to implement (5), our work considers Bayesian hierachical approaches based on Markov chain Monte Carlo (MCMC) methods, which alleviates certain issues in frequentist parameter estimation. First, uncertainty can be characterized using credible intervals. Second, the MCMC approach facilitates density forecasting using predictive distributions obtained from samples of the posterior distribution, which allows more forward information to be projected, compared to point

forecasts alone.

The following hierarchical representation below is considered for the baseline MIDAS model, placing diffuse priors on both the parameters of the observation equation $\mathbf{\Phi}$ and the variance $\sigma_\epsilon^2$. We supress subscripts for $\mathbf{y}$ and $\mathbf{Z}$ for clarity:

$$\begin{aligned} \mathbf{y}|\mathbf{\Phi}, \sigma_\epsilon^2, \mathbf{Z} &\sim N(\mathbf{Z\Phi}, \sigma_\epsilon^2) \\ \mathbf{\Phi} &\sim N(\mathbf{\Phi}_0 = 0, \sigma_\Phi^2 = 10) \\ \sigma_\epsilon^2 &\sim IG(a_0 = 1, b_0 = 1) \end{aligned}$$

Following Ghysels 2016 (Ghysels, 2016), a Gamma distribution is placed as a prior on the parameters of the polynomial terms with both shape and scale parameters being 1, which amounts to a flat weighting scheme that puts equal weight on the high frequency data:

$$\boldsymbol{\theta} \sim \Gamma(\mathbf{f}_0 = 1, \mathbf{F}_0 = 1) \tag{7}$$

Then, obtaining draws from the following conditionally conjugate distributions suffices. $\mathbf{\Phi}$ is directly block sampled from the conditional conjugate posterior distribution as follows:

$$\mathbf{\Phi}|\text{rest} \sim N(\mathbf{\Phi}^*, \sigma_\Phi^2)$$

$$\text{where } \mathbf{\Phi}^* = (\Sigma_0^{-1} + \frac{1}{\sigma^2}\mathbf{Z}'\mathbf{Z})^{-1}(\Sigma_0^{-1}\mathbf{\Phi_0} + \frac{1}{\sigma^2}\mathbf{Z}'\mathbf{y}), \quad \sigma_\Phi^2 = (\Sigma_0^{-1} + \frac{1}{\sigma^2}\mathbf{Z}'\mathbf{Z})^{-1}$$

while $\sigma_\epsilon^2$ is directly sampled from the conditional conjugate posterior:

$$\sigma_\epsilon^2|\text{rest} \sim IG(a_1/2, b_1/2)$$
$$\text{where } a_1 = a_0 + n, \quad b_1 = b_0 + (\mathbf{y} - \mathbf{Z\Phi})'(\mathbf{y} - \mathbf{Z\Phi})$$

while elements of $\boldsymbol{\theta}$ are sampled using a random walk Metropolis-in-Gibbs step. The Metropolis step is an accept-reject step which takes a candidate draw $\theta^*$ from some proposal distribution $q(\theta^*|\theta^{[i]})$ where $\theta^{[i]}$ denotes the last accepted draw. Acceptance for that draw is given by a probability that depends on the likelihood, parameter's prior distribution and the proposal density. In this case, we follow Ghysels 2016 (Ghysels, 2016), to use the Gamma proposal distribution as a proposal density, as it corresponds to the functional form of the MIDAS weighting polynomial.

Namely, for the $i + 1$ iteration of the MCMC sampler, we draw a candidate $\theta_v^* = (\gamma_1^*, \gamma_2^*)$ from

$$\gamma_j^* \sim \Gamma\left(c(\gamma_j^{[i]})^2, c\gamma_j^{[i]}\right), \tag{8}$$

where c is a tuning parameter chosen to acheive a reasonable acceptance rate. We used c=XX, which provided reasonable quick convergence and exploration of the posterior distribution, as shown in traceplots (S1). The candidate draw $\gamma_j^*$ is accepted with probability $a = \min\{\alpha, 1\}$, where

$$\alpha = \frac{L(\mathbf{y}|\mathbf{\Phi}, \sigma, \mathbf{Z}^*) \cdot d\Gamma(\gamma_j^*|\mathbf{f}_0, \mathbf{F}_0) \cdot d\Gamma(\gamma_j^{[i]}|c(\gamma_j^*)^2, c\gamma_j^*)}{L(\mathbf{y}|\mathbf{\Phi}, \sigma, \mathbf{Z}^{[i]}) \cdot d\Gamma(\gamma_j^{[i]}|\mathbf{f}_0, \mathbf{F}_0) \cdot d\Gamma(\gamma_j^*|c(\gamma_j^{[i]})^2, c\gamma_j^{[i]})} \tag{9}$$

where $L(\mathbf{y}|\mathbf{\Phi}, \sigma, \mathbf{Z}^*)$ is the conditional likelihood given the parameters $\mathbf{\Phi}, \mathbf{Z}^*$, $d\Gamma(\gamma_j^*|\mathbf{f}_0, \mathbf{F}_0)$ is the

gamma density function, and denotes the prior density of $\gamma$, while $d\Gamma(\gamma_j^{[i]}|c(\gamma_j^*)^2, c\gamma_j^*)$ denotes the proposal density function which $\gamma$ was sampled from. This step is repeated for each high frequency predictor's weighting scheme separately.

### 1.3 Shrinkage in the observation equation

While the polynomial $B(L^{1/m};\boldsymbol{\theta}_v)$ provides shrinkage of high frequency terms $x_{\tau,v}^{(m)}$, the MIDAS specification presented above takes into consideration a large number of potentially correlated variables, and estimation of parameters may be affected by over-parameterization, multicollinearity and over-fitting. Climate variables in particular have been found to influence vector breeding potential and the transmission of disease and are measured at a high frequency, may be highly correlated if jointly added as predictors in the MIDAS specification, along with lagged terms of disease case counts. Joint addition of these predictors may further increase the number of parameters requiring estimation and hence the dimension of $\mathbf{\Phi}$, leading to inefficient inference in sample and poor out-of-sample predictive performance.

Variable selection and alleviation of collinearity through inducing shrinkage in the MIDAS regression may alleviate this issue. However, parameter estimation under shrinkage using frequentist methods has no convenient measure of standard errors, making inference and characterizing of uncertainty inherently difficult. However, under the Bayesian approach, uncertainty and standard errors can be characterized using credible intervals even under shrinkage, and our MCMC strtaegy allows easy estimation of penalty parameters required for shrinkage as well as other parameters in the observation equation and the MIDAS polynomial.

This study considers extending estimation of (1) by inducing shrinkage structures in the observation equation through Bayesian LASSO (BL), with the priors, hierarchical representation and full computational strategies described below. We primarily extend the baseline MIDAS specification (1) to induce shrinkage in the observation equation (1). This is done by modifying the hierachical representation to allow for more probability weight to be placed on $\mathbf{0}$ on $\mathbf{\Phi}$ compared to the baseline specification in (1). Primarily, in BL of Park and Casella, 2008 equal penalty is placed on all terms of $\mathbf{\Phi}$ through the following hierarchical representation:

$$\mathbf{\Phi}|\sigma^2, \tau_1^2, \ldots, \tau_p^2 \sim \mathrm{N}_p\left(\mathbf{0}_p, \sigma^2\mathbf{D}_\tau\right) \tag{10}$$

$$\mathbf{D}_\tau = \mathrm{diag}\left(\tau_1^2, \ldots, \tau_p^2\right) \tag{11}$$

$$\sigma^2, \tau_1^2, \ldots, \tau_p^2 \sim \pi(\sigma^2)\prod_{j=1}^{p}\frac{\lambda}{2}e^{-\lambda^2\tau_j^2/2}d\tau_j^2 \tag{12}$$

which allows shrinkage of terms by representing $\mathbf{\Phi}$ as a scale mixture of normal distributions with an exponential mixing density (Park and Casella, 2008). Note that $\tau$ here represents the shrinkage parameter rather than time in the previous sections.

A simple modification to the BL hierarchical structure by replacing (12) using a more adaptive penalty incorporating element-wise shrinkage:

$$\sigma^2, \tau_1^2, \ldots, \tau_p^2 \sim \pi(\sigma^2)\prod_{j=1}^{p}\frac{\lambda_j}{2}e^{-\lambda_j^2\tau_j^2/2}d\tau_j^2 \tag{13}$$

by having different shrinkage hyper-parameters $\lambda_j$ for each $\tau_j$. This allows (10), (11) and (13) to complete the BAL hierarchical representation. This specification allows smaller shrinkage for parameters which are deemed important by the data, and larger shrinkage for parameters which are deemed unimportant.

### 1.4 Implementation of observation equation shrinkage through Bayesian LASSO

We now modify the sampling procedures detailed in (1.2) to incorporate shrinkage as proposed above. In brief, the conditional posterior distribution for $\mathbf{\Phi}$ was modified for the type of shrinkage which was imposed. The MCMC procedures also additionally sample from the conditional posterior distributions of other parameters which are required for each form of shrinkage. For the BL, the conditional posterior distributions to sample for $\mathbf{\Phi}$ and $\sigma^2_\epsilon$ were modified due to the choice of shrinkage priors:

$$\mathbf{\Phi}|\text{rest} \sim N(\mathbf{\Phi}^*, \sigma^2_\Phi) \quad \text{where} \quad \mathbf{\Phi}^* = (\boldsymbol{D}_\tau^{-1} + \mathbf{Z}'\mathbf{Z})^{-1}\mathbf{Z}'\mathbf{y}, \quad \sigma^2_\Phi = \sigma^2(\boldsymbol{D}_\tau^{-1} + \mathbf{Z}'\mathbf{Z})^{-1}$$

$$\sigma^2_\epsilon|\text{rest} \sim IG(a_1/2, b_1/2) \quad \text{where} \quad a_1 = p + n - 1, \quad b_1 = (\mathbf{y} - \mathbf{Z\Phi})'(\mathbf{y} - \mathbf{Z\Phi}) + \mathbf{\Phi}'\boldsymbol{D}_\tau^{-1}\mathbf{\Phi}$$

where $p$ denotes the number of parameters in the observation equation and $n$ denotes the number of observations. Whereas $\tau_1^2, \ldots \tau_p^2$ and $\lambda^2$ are directly sampled from the following inverse Gaussian and Gamma distributions respectively:

$$\tau_j^2|\text{rest} \sim \text{inverse-Gaussian}\left(\sqrt{\frac{\lambda^2\sigma^2}{\beta_j^2}}, \lambda^2\right) \tag{14}$$

$$\lambda^2|\text{rest} \sim \Gamma\left(p + r, \sum_{j=1}^{p} \frac{\tau_j^2}{2} + \delta\right) \tag{15}$$

# 2 Results using disease case counts normalized by population size

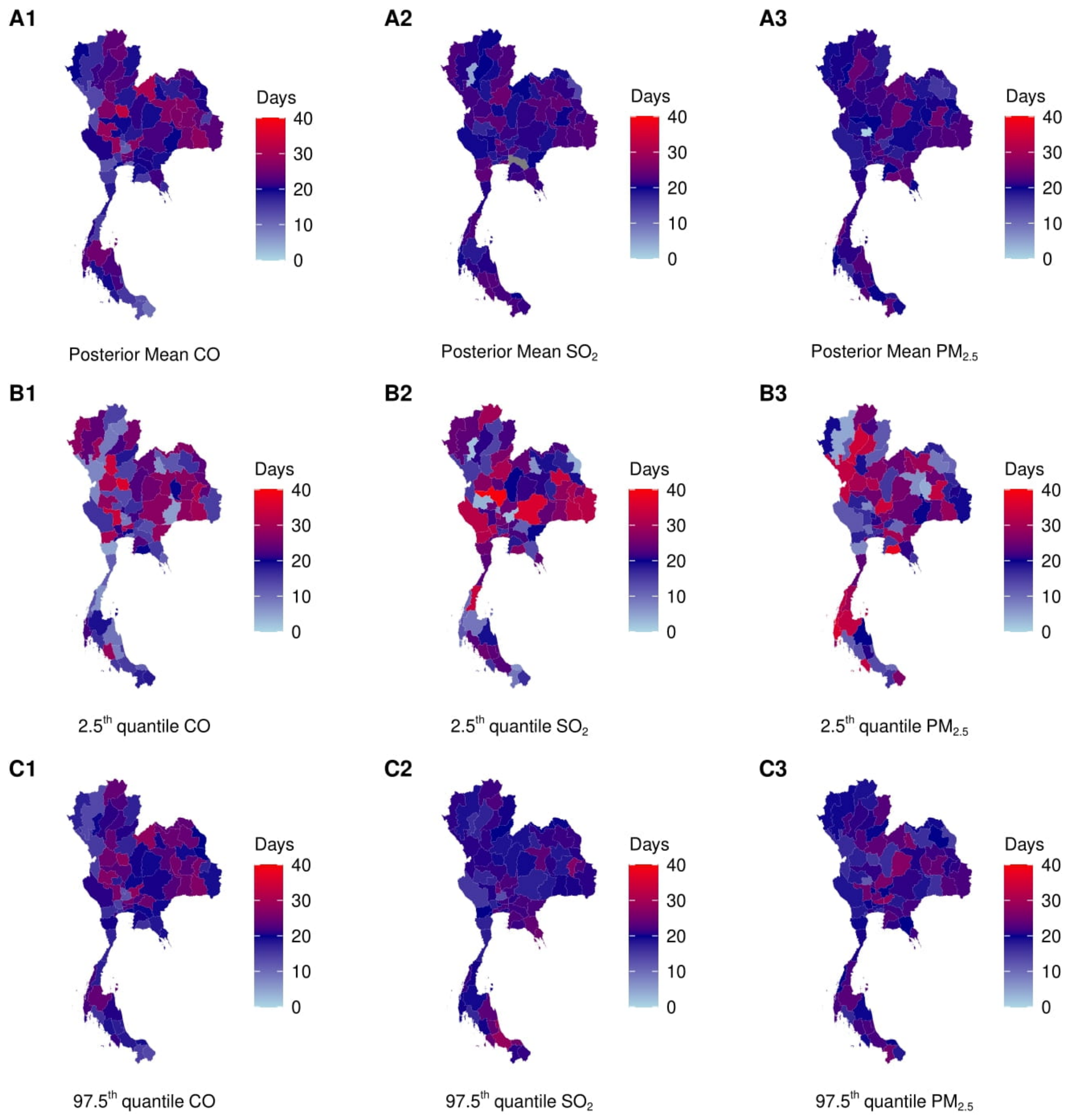


Figure 1: **A1 − A3** Posterior mean estimates of duration where the highest MIDAS weights were placed on the importance of respective ambient air pollutant measurements for each province on influencing URTI disease case counts per 100,000 individuals the following month. Darker red and blue shaded regions represent that ambient air pollutant measurements beyond 20 − 40 days are deemed more important in determining URTI disease case counts per 100,000 individuals . **B1 − B3** 2.5th quantile value for MIDAS weights drawn from MCMC samples **C1 − C3** 97.5th quantile value for MIDAS weights drawn from MCMC samples

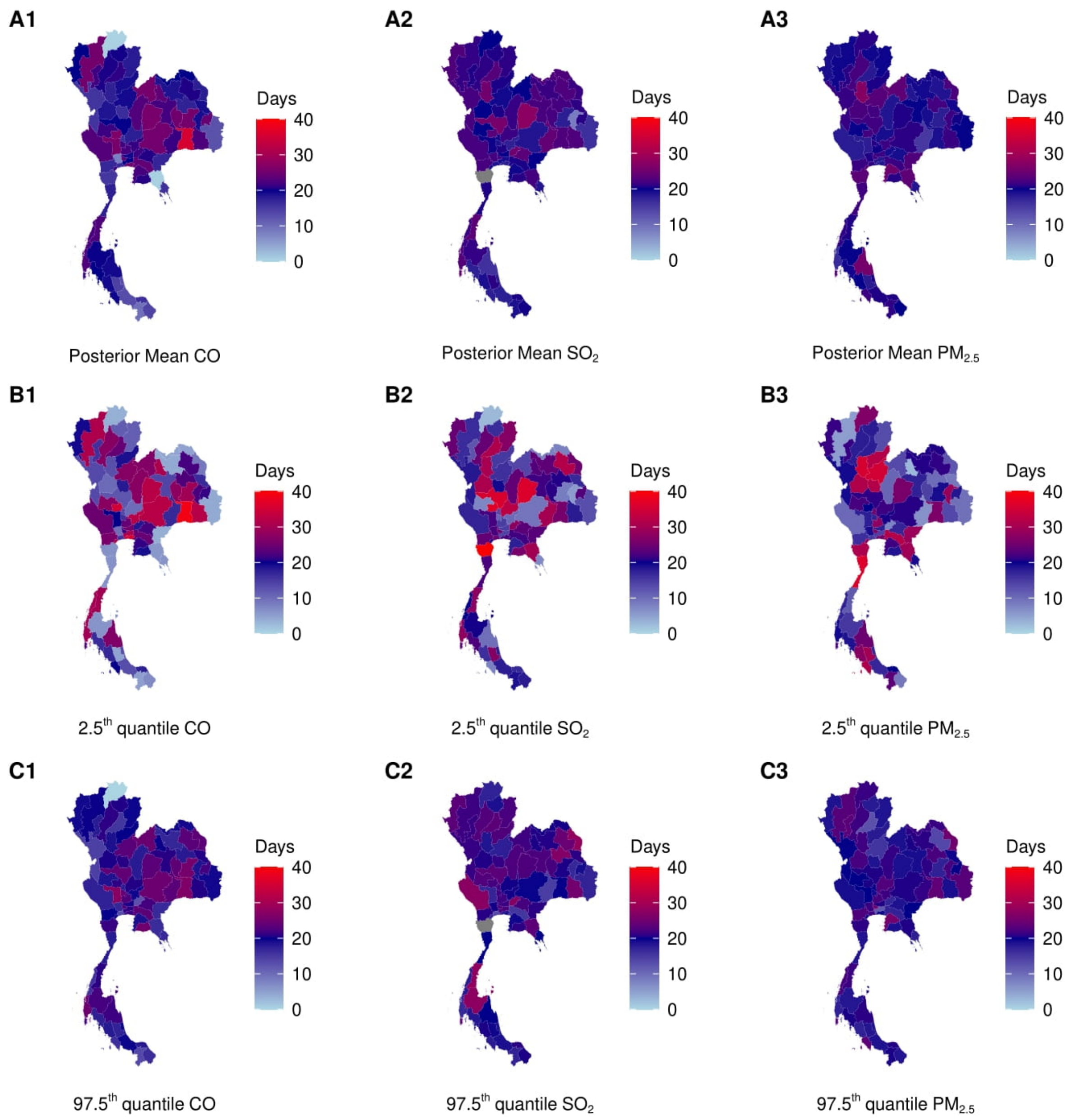


Figure 2: **A1 − A3** Posterior mean estimates of duration where the highest MIDAS weights were placed on the importance of respective ambient air pollutant measurements for each province on influencing pneumonia disease case counts per 100,000 individuals the following month. Darker red and blue shaded regions represent that ambient air pollutant measurements beyond 20 − 40 days are deemed more important in determining pneumonia disease case counts per 100,000 individuals . **B1 − B3** 2.5th quantile value for MIDAS weights drawn from MCMC samples **C1 − C3** 97.5th quantile value for MIDAS weights drawn from MCMC samples

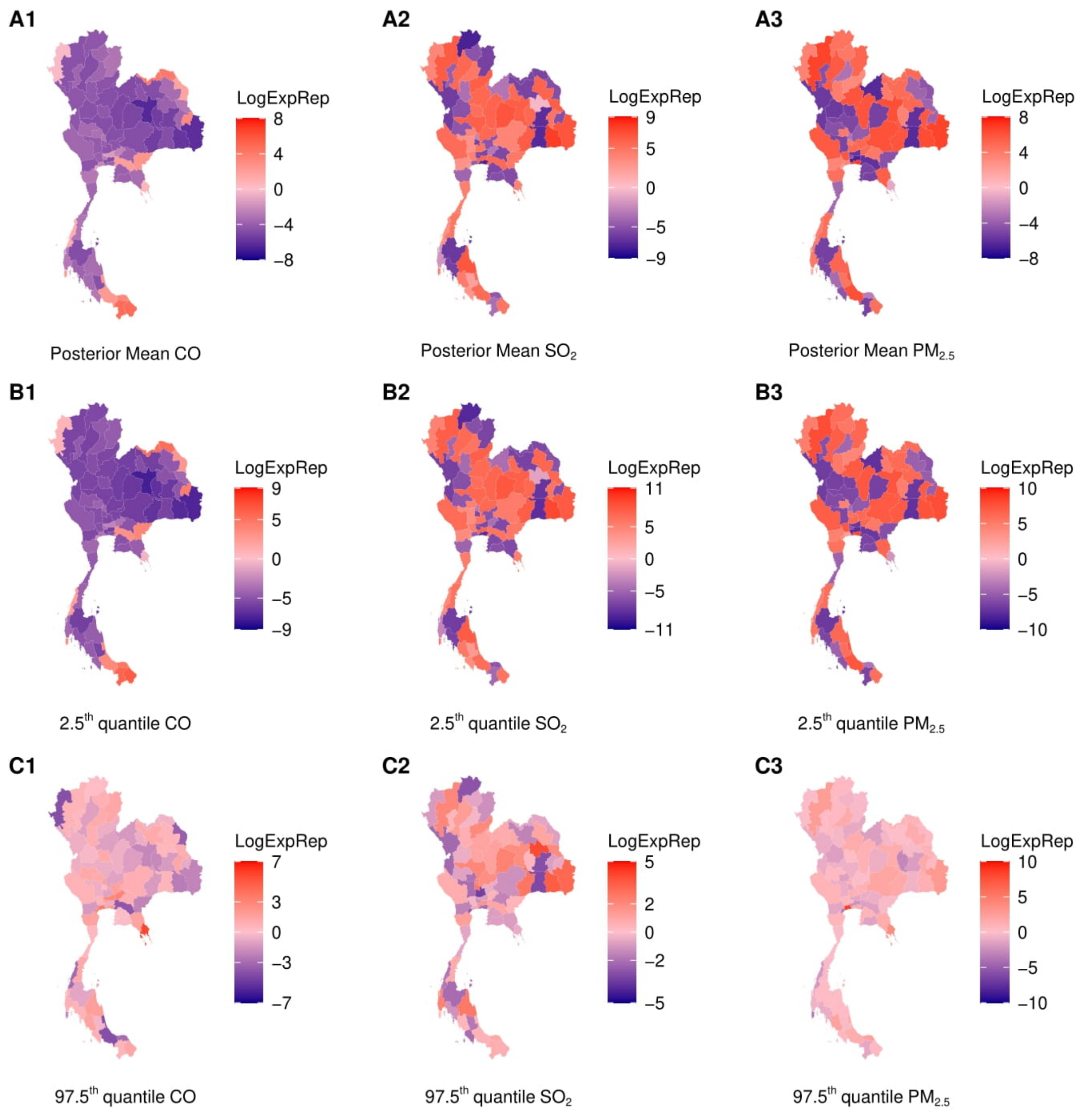


Figure 3: **A1 − A3** Posterior mean estimates of mean exposure-response of respective ambient air pollutants over the past 40 day period on contemporaneous URTI disease case counts per 100,000 individuals for a specific region. Red shades represent regions where a one unit increase in ambient air pollutant surface concentrations in the respective region for the past 40 days are associated to an increase in contemporaneous, monthly URTI disease case counts per 100,000 individuals . **B1 − B3** 2.5th quantile value for exposure response drawn from MCMC samples of MIDAS weights **C1 − C3** 97.5th quantile value for exposure response drawn from MCMC samples of MIDAS weights

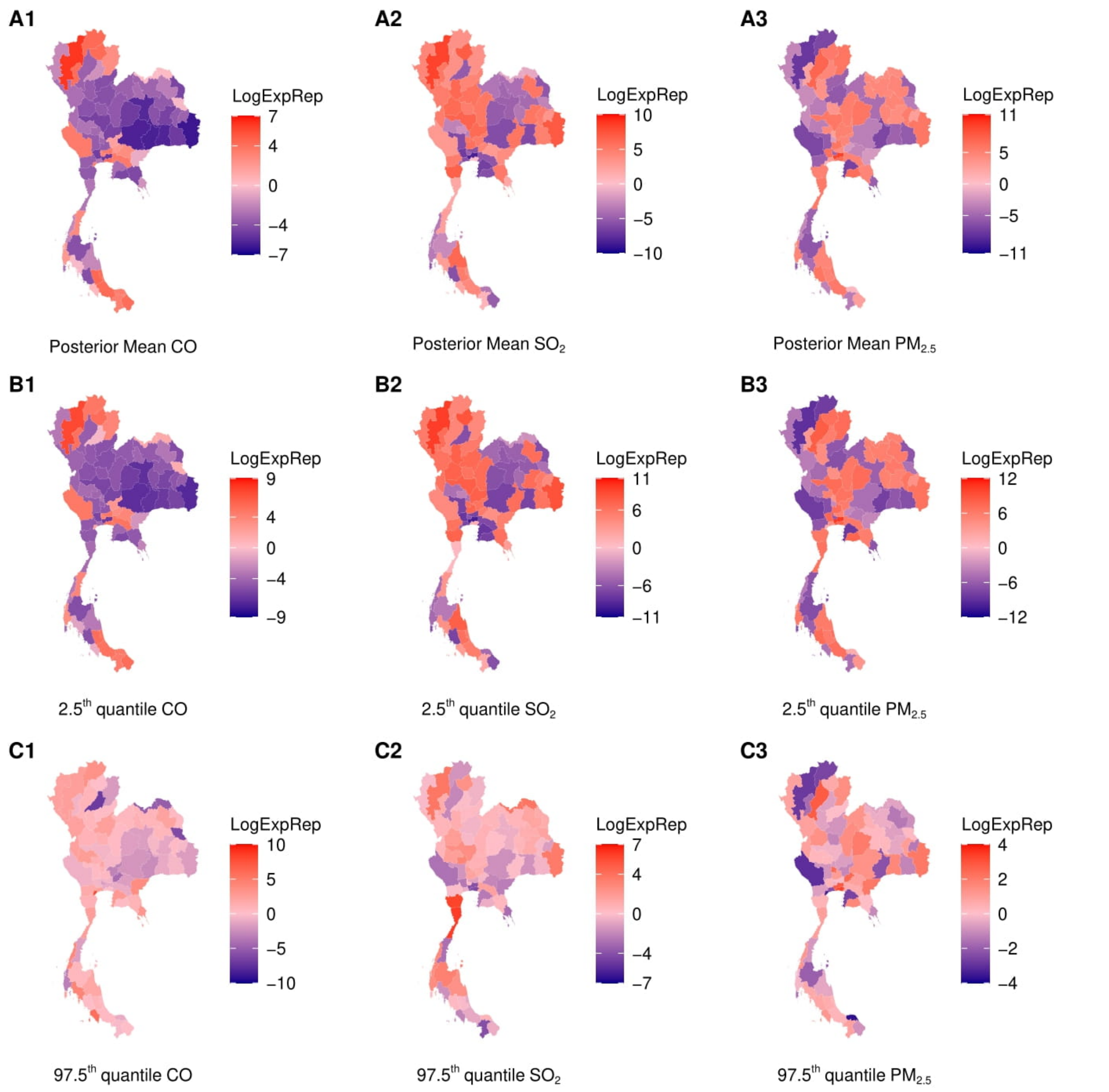


Figure 4: **A1 − A3** Posterior mean estimates of mean exposure-response of respective ambient air pollutants over the past 40 day period on contemporaneous pneumonia case counts per 100,000 individuals for a specific region. Red shades represent regions where a one unit increase in ambient air pollutant surface concentrations in the respective region for the past 40 days are associated to an increase in contemporaneous, monthly pneumonia case counts per 100,000 individuals . **B1 − B3** 2.5th quantile value for exposure response drawn from MCMC samples **C1 − C3** 97.5th quantile value for exposure response drawn from MCMC samples

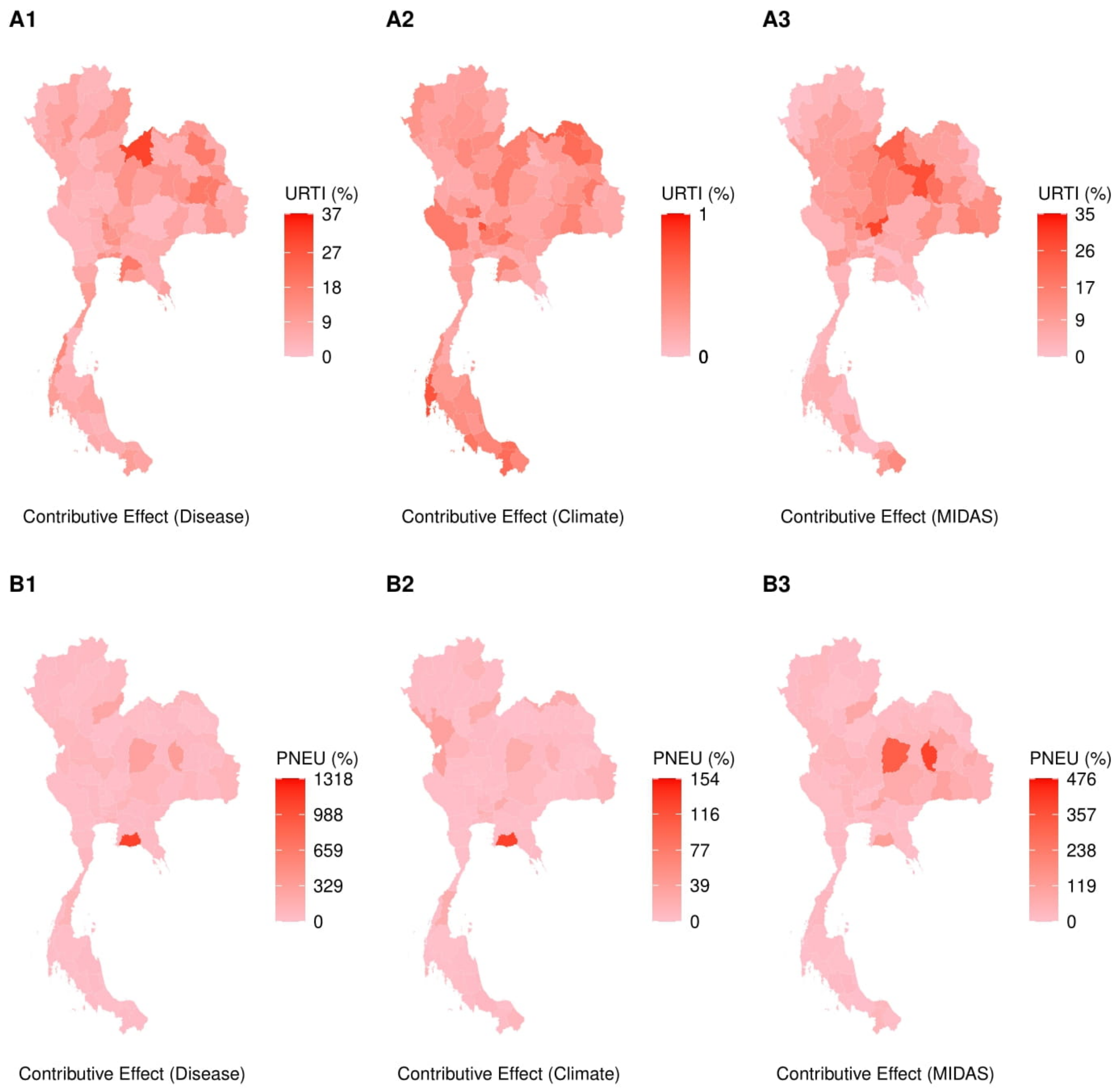


Figure 5: **A1** $-$ **A3** Average absolute contribution of disease case counts the past two months, climate measurements the past two months (i.e temperature, total precipitation, absolute humidity, relative humidity) as well as ambient air pollutants as weighted under MIDAS over the past 40 day period on predicted contemporaneous URTI case counts per 100,000 individuals **B1** $-$ **B3** Average absolute contribution of disease case counts per 100,000 individuals the past two months, climate measurements the past two months (i.e temperature, total precipitation, absolute humidity, relative humidity) as well as ambient air pollutants as weighted under MIDAS over the past 40 day period on predicted contemporaneous pneumonia case counts per 100,000 individuals

# 3 Model assessment metrics with unnormalized case counts as dependent variables

Table 1: Deviance Information Criterion for URTI case counts

| | AR | LASSO-MIDAS | | | AR-MIDAS | | |
|---|---|---|---|---|---|---|---|
| | | Free | Down | Hump | Free | Down | Hump |
| Amnat Charoen | 2399.89∗ | 2401.49 | 2401.14 | 2401.18 | 2437.38 | 2435.89 | 2437.09 |
| Ang Thong | 2263.58 | 2246.86∗ | 2247.19 | 2247.76 | 2390.36 | 2387.93 | 2389.8 |
| Bangkok | 3085.22 | 3067.83 | 3067.46∗ | 3067.54 | 3385.34 | 3384.83 | 3386.1 |
| Buri Ram | 2820.64 | 2792.79∗ | 2793.19 | 2793.18 | 3023.69 | 3023.41 | 3023.24 |
| Chachoengsao | 2612.95 | 2612.12 | 2611.94∗ | 2611.95 | 2757.52 | 2755.33 | 2757.16 |
| Chai Nat | 1819.24 | 1806.7 | 1805.59∗ | 1806.23 | 1857.54 | 1858.16 | 1858.02 |
| Chaiyaphum | 2653.58 | 2629.63∗ | 2631.1 | 2630.59 | 2865.26 | 2863.32 | 2865.99 |
| Chanthaburi | 2316.37∗ | 2317.87 | 2317.66 | 2317.41 | 2602.46 | 2598.5 | 2602.57 |
| Chiang Mai | 2880.09 | 2833.87∗ | 2833.87 | 2834.37 | 3300.07 | 3298.7 | 3300.04 |
| Chiang Rai | 2855.59 | 2818.63 | 2818.5 | 2818.43∗ | 3208.57 | 3206.5 | 3208.93 |
| Chon Buri | 2587.89 | 2576.34 | 2575.46∗ | 2576.78 | 2802 | 2800.59 | 2799.83 |
| Chumphon | 2222.94 | 2222.82 | 2222.1∗ | 2223.28 | 2377.9 | 2375.32 | 2365.56 |
| Kalasin | 2324.19 | 2311 | 2312.01 | 2310.04∗ | 2389.6 | 2387.53 | 2388.61 |
| Kamphaeng Phet | 2522.18 | 2508.87 | 2508.68 | 2507.51∗ | 2784.37 | 2783.09 | 2784.25 |
| Kanchanaburi | 2544.65 | 2530.08 | 2529.98∗ | 2530.38 | 2735.68 | 2734.12 | 2735.54 |
| Khon Kaen | 2973.21 | 2940.18∗ | 2942.16 | 2940.45 | 3275.87 | 3274.03 | 3275.67 |
| Krabi | 2306.46 | 2282.62 | 2282.14∗ | 2282.45 | 2483.62 | 2485.37 | 2483.38 |
| Lampang | 2481.32 | 2454.81∗ | 2455.06 | 2454.9 | 2734.6 | 2732.73 | 2734.11 |
| Lamphun | 2050.53 | 2037.74 | 2037.33∗ | 2037.62 | 2207.81 | 2205.57 | 2205.45 |
| Loei | 2444.47 | 2431.74 | 2432.34 | 2430.59∗ | 2694.1 | 2691.65 | 2693.73 |
| Lop Buri | 2492.09 | 2477.75∗ | 2478.6 | 2478.26 | 2647.92 | 2646.25 | 2648.19 |
| Mae Hong Son | 2336.13 | 2306.66 | 2306.39 | 2306.18∗ | 2486.22 | 2484.08 | 2486.55 |
| Maha Sarakham | 2765.72 | 2751.71 | 2754.17 | 2751.61∗ | 2992.3 | 2990.92 | 2992.36 |
| Mukdahan | 2291.83 | 2287.07∗ | 2287.79 | 2287.43 | 2342.28 | 2340.81 | 2342.54 |
| Nakhon Nayok | 1972.98 | 1970.56 | 1969.64∗ | 1970.67 | 1993.72 | 1993.52 | 1990.65 |
| Nakhon Pathom | 2450.51 | 2444.65∗ | 2444.7 | 2445.71 | 2694.82 | 2693.13 | 2693.81 |
| Nakhon Phanom | 2479.37 | 2457.6∗ | 2458.3 | 2458.18 | 2552.75 | 2550.73 | 2552.44 |
| Nakhon Ratchasima | 2796.78 | 2768.3∗ | 2769.15 | 2769.11 | 2961.3 | 2961.23 | 2961.55 |
| Nakhon Sawan | 2417.2 | 2399.25 | 2399.05∗ | 2399.21 | 2657.24 | 2655.46 | 2656.26 |
| Nakhon Si Thammarat | 2553.71 | 2532.37 | 2532.46 | 2531.4∗ | 2790.53 | 2793.13 | 2791.62 |
| Nan | 2323.46 | 2308.72∗ | 2309.4 | 2309.49 | 2553.6 | 2552.73 | 2553.57 |
| Narathiwat | 2453.78 | 2444.51∗ | 2445.62 | 2445.07 | 2559.63 | 2561.79 | 2560.57 |
| Nong Bua Lam Phu | 2143.92 | 2131.59 | 2132.04 | 2131.2∗ | 2235.7 | 2233.8 | 2235.46 |
| Nong Khai | 2437.2 | 2410.03∗ | 2410.03 | 2410.1 | 2540.24 | 2538.22 | 2539.47 |
| Nonthaburi | 2198.54∗ | 2203.54 | 2204.21 | 2203.71 | 2317.16 | 2315.73 | 2317.14 |
| Pathum Thani | 2628.49 | 2615.3∗ | 2615.73 | 2615.56 | 2848.58 | 2847.36 | 2848.97 |
| Pattani | 2268.14 | 2249.28 | 2249.48 | 2248.46∗ | 2332.44 | 2331.61 | 2332.2 |
| Phangnga | 2094.7 | 2074.89 | 2074.7 | 2074.67∗ | 2146.7 | 2146.87 | 2147.08 |
| Phatthalung | 2290.12 | 2269.74 | 2269.93 | 2269.32∗ | 2392.55 | 2390.66 | 2392.37 |
| Phayao | 2371.32 | 2356.12 | 2356.52 | 2356.05∗ | 2556.03 | 2553.5 | 2555.4 |

| | | | | | | | |
|---|---|---|---|---|---|---|---|
| Phetchabun | 2606.17 | 2589.75* | 2590.49 | 2590.39 | 2816.19 | 2814.59 | 2816.43 |
| Phetchaburi | 2208.7* | 2211.44 | 2211.15 | 2212 | 2407.26 | 2404.49 | 2392.04 |
| Phichit | 2266.97 | 2261.88 | 2262.37 | 2261.44* | 2367.3 | 2366.42 | 2368.07 |
| Phitsanulok | 2400.82 | 2383.5 | 2383.05* | 2383.69 | 2626.32 | 2624.33 | 2625.77 |
| Phra Nakhon Si Ayutthaya | 2630.33 | 2613.76 | 2613.65* | 2614.3 | 2802.8 | 2801.2 | 2802.47 |
| Phrae | 2155.98 | 2134.16 | 2134.13* | 2134.31 | 2358.03 | 2355.05 | 2354.51 |
| Phuket | 2185.08 | 2173.47* | 2173.68 | 2173.72 | 2280.7 | 2280.86 | 2280.42 |
| Prachin Buri | 2647.69 | 2646.23 | 2645.93* | 2646.16 | 2762.13 | 2761.62 | 2762.08 |
| Prachuap Khiri Khan | 2245.88 | 2246.09 | 2245.31* | 2246.23 | 2485.7 | 2482.57 | 2477.08 |
| Ranong | 1843.02* | 1849.02 | 1849.28 | 1849.31 | 1864.64 | 1864.97 | 1866.01 |
| Ratchaburi | 2367.85 | 2355.02 | 2354.67* | 2355.57 | 2588.12 | 2586.48 | 2582.05 |
| Rayong | 2416.4 | 2402.2 | 2401.69* | 2402.41 | 2746.6 | 2743.19 | 2742.06 |
| Roi Et | 2717.43 | 2694.43 | 2694.17 | 2694.11* | 3071.82 | 3071.06 | 3072.23 |
| Sa Kaeo | 1934.76 | 1930.55* | 1930.77 | 1930.6 | 1961.58 | 1960.36 | 1960.16 |
| Sakon Nakhon | 2359.65 | 2340.56* | 2341.52 | 2340.56 | 2515.43 | 2513.32 | 2515.48 |
| Samut Prakan | 2666.57* | 2671.25 | 2670.79 | 2671.43 | 3029.65 | 3029.17 | 3029.27 |
| Samut Sakhon | 2059.75 | 2055.54 | 2055.36* | 2055.69 | 2140.12 | 2138.72 | 2137.15 |
| Samut Songkhram | 1759.73* | 1765.09 | 1765.43 | 1765.07 | 1806.43 | 1806.01 | 1805.49 |
| Saraburi | 2486.45 | 2476.34 | 2476.85 | 2476.25* | 2605.03 | 2603.84 | 2605.45 |
| Satun | 1996.99 | 1980.35 | 1980.34* | 1980.92 | 2034.61 | 2032.68 | 2033.24 |
| Si Sa Ket | 2922.8 | 2887.43 | 2887.36 | 2885.77* | 3229.41 | 3229.92 | 3229.9 |
| Sing Buri | 1888.2 | 1888.32 | 1887.39* | 1888.03 | 1934.46 | 1934.79 | 1934.32 |
| Songkhla | 2634.55 | 2601.15 | 2601.74 | 2600.43* | 2838.51 | 2837.32 | 2838.52 |
| Sukhothai | 2193.32 | 2179.15 | 2178.67* | 2178.76 | 2356.51 | 2354.56 | 2355.84 |
| Suphan Buri | 2360.84 | 2358.29 | 2357.69 | 2357.23* | 2559.77 | 2558.72 | 2560.05 |
| Surat Thani | 2664.47 | 2632.29 | 2632.61 | 2632.14* | 2950.56 | 2954.76 | 2953.32 |
| Surin | 2851.09 | 2827.72 | 2827.75 | 2827.46* | 2972.32 | 2971.59 | 2972.25 |
| Tak | 2460.01 | 2423.51* | 2424.03 | 2424.62 | 2747.49 | 2745.06 | 2746.38 |
| Trang | 2336.91 | 2316.25 | 2316 | 2315.88* | 2487.8 | 2488.49 | 2488.05 |
| Trat | 1997.46* | 2005 | 2005.23 | 2005.02 | 2112.75 | 2112.88 | 2112.58 |
| Ubon Ratchathani | 3186.51 | 3163.21 | 3163.53 | 3162.92* | 3385.89 | 3385.54 | 3386.07 |
| Udon Thani | 2679.21 | 2652.92* | 2654.42 | 2653.62 | 2832.74 | 2830.96 | 2831.59 |
| Uthai Thani | 2109.99 | 2089.84 | 2089.56 | 2088.68* | 2276.92 | 2275.59 | 2276.24 |
| Uttaradit | 2409.78 | 2387.45* | 2389.47 | 2388.45 | 2560.49 | 2559.53 | 2560.06 |
| Yala | 2454.25 | 2439.57* | 2439.72 | 2440.03 | 2602.88 | 2601 | 2603.76 |
| Yasothon | 2441.6 | 2423.55 | 2423.84 | 2423.47* | 2600.59 | 2598.87 | 2600.48 |

Table 2: Deviance Information Criterion for pneumonia case counts

| | AR | LASSO-MIDAS | | | AR-MIDAS | | |
|---|---|---|---|---|---|---|---|
| | | Free | Down | Hump | Free | Down | Hump |
| Amnat Charoen | 1806.89 | 1804.03∗ | 1807.42 | 1804.54 | 1818.55 | 1818.63 | 1818.63 |
| Ang Thong | 2127.1∗ | 2127.47 | 2127.94 | 2127.43 | 2172.54 | 2172.5 | 2173.22 |
| Bangkok | 3986.22 | 3980.72∗ | 3981.01 | 3981.04 | 4221.03 | 4220.67 | 4221.5 |
| Buri Ram | 2985.53∗ | 2988.67 | 2988 | 2987.93 | 3176.99 | 3176.82 | 3177.44 |
| Chachoengsao | 2625.22∗ | 2632.28 | 2632.3 | 2632.23 | 2839.14 | 2839.12 | 2839.38 |
| Chai Nat | 1913.95∗ | 1916.37 | 1916.04 | 1915.48 | 1926.29 | 1926.36 | 1926.14 |
| Chaiyaphum | 2742.54∗ | 2745.94 | 2747.16 | 2745.68 | 2931.43 | 2931.31 | 2932.36 |
| Chanthaburi | 2747.17 | 2739.87 | 2739.65∗ | 2739.88 | 2973.25 | 2971.65 | 2972.6 |
| Chiang Mai | 3364.59 | 3345.12 | 3344.97 | 3344.96∗ | 3637.22 | 3635.97 | 3637.32 |
| Chiang Rai | 2963.2 | 2959.26 | 2959.14∗ | 2959.24 | 3133.68 | 3132.84 | 3133.57 |
| Chon Buri | 2870.55∗ | 2874.02 | 2873.85 | 2874.27 | 3089.16 | 3089.26 | 3089.25 |
| Chumphon | 2362.37∗ | 2364 | 2363.99 | 2363.85 | 2448.18 | 2447.82 | 2446.65 |
| Kalasin | 2269.8 | 2260.15 | 2260.22 | 2260.14∗ | 2307.13 | 2306.36 | 2306.96 |
| Kamphaeng Phet | 2699.72∗ | 2703.08 | 2702.95 | 2703.15 | 2826.33 | 2825.88 | 2826.46 |
| Kanchanaburi | 2762.47 | 2753.93 | 2754.26 | 2753.86∗ | 2906.91 | 2906.58 | 2906.55 |
| Khon Kaen | 2947.2∗ | 2948.63 | 2948.81 | 2948.32 | 3130.43 | 3129.85 | 3131.23 |
| Krabi | 2251.47 | 2250.34 | 2250.26 | 2250.14∗ | 2271.73 | 2271.66 | 2271.94 |
| Lampang | 2939.34 | 2933.23∗ | 2933.33 | 2933.7 | 3095.85 | 3094.79 | 3096.48 |
| Lamphun | 2529.77 | 2526.44 | 2526.16 | 2526.01∗ | 2663.12 | 2662.49 | 2663.41 |
| Loei | 2322.57 | 2320.01 | 2319.83 | 2318.82∗ | 2379.33 | 2378.74 | 2380.29 |
| Lop Buri | 2655.85 | 2654.53∗ | 2654.93 | 2655.14 | 2764.1 | 2763.38 | 2763.65 |
| Mae Hong Son | 2114.14 | 2107.9 | 2107.51∗ | 2108.16 | 2137.96 | 2137.98 | 2137.33 |
| Maha Sarakham | 2385.07∗ | 2386.92 | 2387.48 | 2386.55 | 2496.29 | 2495.68 | 2496.97 |
| Mukdahan | 2387.37 | 2376.93 | 2377 | 2376.9∗ | 2435.46 | 2435.85 | 2435.73 |
| Nakhon Nayok | 1924.12∗ | 1924.93 | 1924.56 | 1925.01 | 1924.86 | 1924.99 | 1924.85 |
| Nakhon Pathom | 2956.8∗ | 2958.69 | 2958.57 | 2959.28 | 3196.55 | 3195.26 | 3197.13 |
| Nakhon Phanom | 2472.32 | 2442.89∗ | 2442.9 | 2443.47 | 2589.24 | 2589.51 | 2590.16 |
| Nakhon Ratchasima | 3387.44∗ | 3390.6 | 3391.13 | 3390.61 | 3639.69 | 3639.15 | 3639.96 |
| Nakhon Sawan | 2873.84∗ | 2877.86 | 2877.32 | 2877.76 | 3122.58 | 3122.72 | 3123.17 |
| Nakhon Si Thammarat | 2667.93 | 2666.14 | 2666.12 | 2665.91∗ | 2921.22 | 2922.3 | 2921.91 |
| Nan | 2393.24 | 2392.64∗ | 2392.74 | 2392.67 | 2531.74 | 2531.97 | 2531.58 |
| Narathiwat | 2262.58 | 2254.82 | 2254.26∗ | 2254.67 | 2282.77 | 2281.57 | 2283.56 |
| Nong Bua Lam Phu | 2103.54∗ | 2109.24 | 2110.07 | 2109.39 | 2126.35 | 2126.1 | 2126.33 |
| Nong Khai | 2843.45 | 2843.13 | 2842.91 | 2842.89∗ | 2937.76 | 2937.24 | 2938.11 |
| Nonthaburi | 2616.78∗ | 2622.09 | 2621.98 | 2622.46 | 2743.28 | 2742.47 | 2743.29 |
| Pathum Thani | 2585.9∗ | 2591.65 | 2591.67 | 2591.81 | 2772.3 | 2772.11 | 2772.28 |
| Pattani | 1901.75∗ | 1903.06 | 1903.3 | 1903.39 | 1902.37 | 1902.81 | 1903.24 |
| Phangnga | 2047.34∗ | 2047.85 | 2047.93 | 2047.82 | 2094.22 | 2095.21 | 2093.72 |
| Phatthalung | 2361.73∗ | 2365.99 | 2365.69 | 2365.89 | 2417.05 | 2416.61 | 2417.55 |
| Phayao | 2716.37 | 2712.52 | 2712.45∗ | 2712.76 | 2908.61 | 2908.29 | 2909.44 |
| Phetchabun | 2477.52∗ | 2482.1 | 2482.39 | 2481.56 | 2624.53 | 2623.81 | 2625.01 |
| Phetchaburi | 2417.54∗ | 2424.12 | 2423.45 | 2424 | 2505.37 | 2505.83 | 2503.55 |
| Phichit | 2501.81 | 2499.76 | 2499.22∗ | 2499.84 | 2574.43 | 2574.08 | 2574.17 |

| | | | | | | | |
|---|---|---|---|---|---|---|---|
| Phitsanulok | 3069.94 | 3054.3 | 3053.44∗ | 3053.91 | 3187.55 | 3187.12 | 3187.96 |
| Phra Nakhon Si Ayutthaya | 2661.72∗ | 2661.96 | 2662.88 | 2662.38 | 2852.43 | 2851 | 2852.63 |
| Phrae | 2418.35∗ | 2423.97 | 2424.2 | 2423.92 | 2455.56 | 2455.45 | 2455.98 |
| Phuket | 2561.69 | 2559.93∗ | 2559.95 | 2560.06 | 2648.08 | 2648.13 | 2648.1 |
| Prachin Buri | 2392.34∗ | 2398.25 | 2398.01 | 2398.16 | 2484.61 | 2485.18 | 2485.55 |
| Prachuap Khiri Khan | 2419.19∗ | 2422.64 | 2422.34 | 2422.86 | 2565.31 | 2565.3 | 2563.05 |
| Ranong | 1611.96 | 1607.97 | 1607.93∗ | 1608.49 | 1609.01 | 1609.1 | 1609.23 |
| Ratchaburi | 2655.99∗ | 2661.21 | 2660.89 | 2662.07 | 2786.31 | 2786.15 | 2785.16 |
| Rayong | 2990.5 | 2988.71∗ | 2988.83 | 2988.75 | 3256.47 | 3256.18 | 3257.03 |
| Roi Et | 2412.66 | 2409.04 | 2409.46 | 2408.54∗ | 2569.42 | 2569.21 | 2569.86 |
| Sa Kaeo | 1849.66∗ | 1855.83 | 1855.54 | 1856.1 | 1872.85 | 1872.66 | 1871.92 |
| Sakon Nakhon | 2201.94 | 2185.44 | 2185.3 | 2185.29∗ | 2230.12 | 2229.87 | 2231.06 |
| Samut Prakan | 2914.52∗ | 2915.8 | 2915.75 | 2915.43 | 3142.97 | 3143.2 | 3143.38 |
| Samut Sakhon | 2733.92∗ | 2741.33 | 2741.36 | 2741.37 | 2842.03 | 2842.24 | 2841.89 |
| Samut Songkhram | 1782.62∗ | 1789.67 | 1789.32 | 1789.55 | 1797.53 | 1797 | 1796.99 |
| Saraburi | 2275.67∗ | 2282.38 | 2282.6 | 2282.2 | 2323.07 | 2322.62 | 2323.47 |
| Satun | 1651.59∗ | 1656.48 | 1656.98 | 1656.95 | 1660.5 | 1660.75 | 1660.24 |
| Si Sa Ket | 2454.01 | 2452.29 | 2451.64∗ | 2451.99 | 2622.44 | 2621.99 | 2622.21 |
| Sing Buri | 1959.6∗ | 1963.81 | 1964.29 | 1964.12 | 1971.73 | 1971.77 | 1971.85 |
| Songkhla | 2569.07 | 2568.48 | 2568.34∗ | 2568.55 | 2683.93 | 2683.27 | 2684.08 |
| Sukhothai | 2700.34∗ | 2703.01 | 2702.5 | 2703.55 | 2820.95 | 2821.63 | 2821.38 |
| Suphan Buri | 2498.49∗ | 2504.65 | 2504.95 | 2504.66 | 2620.59 | 2620.32 | 2621.02 |
| Surat Thani | 2765.11∗ | 2766.16 | 2766.75 | 2766.4 | 2991.54 | 2991.2 | 2990.98 |
| Surin | 2793.34 | 2793.36 | 2792.04∗ | 2792.52 | 3028.72 | 3028.36 | 3028.78 |
| Tak | 2401.83 | 2393.93∗ | 2393.96 | 2394.27 | 2503.88 | 2503.62 | 2502.63 |
| Trang | 2531.03 | 2526.27∗ | 2526.5 | 2526.73 | 2605.01 | 2604.29 | 2603.84 |
| Trat | 2119.5 | 2116.82∗ | 2117.27 | 2116.94 | 2185.27 | 2184.91 | 2184.4 |
| Ubon Ratchathani | 3214.37 | 3208.63 | 3207.81∗ | 3208.35 | 3456.31 | 3456.46 | 3456.7 |
| Udon Thani | 2633.08∗ | 2639.39 | 2638.87 | 2638.9 | 2792.3 | 2792.06 | 2792.6 |
| Uthai Thani | 2097.22 | 2093.03 | 2092.77∗ | 2093.42 | 2114.31 | 2114.4 | 2114.13 |
| Uttaradit | 2679.81 | 2669.25 | 2668.69∗ | 2669.06 | 2830.64 | 2830.54 | 2831.31 |
| Yala | 1867.84∗ | 1869.53 | 1870.41 | 1870.58 | 1869.34 | 1870.64 | 1870.37 |
| Yasothon | 2291.48 | 2286.43∗ | 2287.33 | 2287.15 | 2363.99 | 2363.7 | 2364.18 |

Table 3: Adjusted $R^2$ for URTI case counts

| | AR | LASSO-MIDAS | | | AR-MIDAS | | |
|---|---|---|---|---|---|---|---|
| | | Free | Down | Hump | Free | Down | Hump |
| Amnat Charoen | 0.278 | 0.292 | 0.293∗ | 0.292 | 0.137 | 0.142 | 0.14 |
| Ang Thong | 0.614 | 0.654∗ | 0.654 | 0.653 | 0.317 | 0.325 | 0.319 |
| Bangkok | 0.625 | 0.668 | 0.669∗ | 0.669 | -0.037 | -0.035 | -0.036 |
| Buri Ram | 0.555 | 0.622∗ | 0.622 | 0.622 | -0.035 | -0.033 | -0.034 |
| Chachoengsao | 0.493 | 0.513 | 0.514∗ | 0.513 | 0.045 | 0.047 | 0.044 |
| Chai Nat | 0.36 | 0.411 | 0.414∗ | 0.412 | 0.257 | 0.254 | 0.256 |
| Chaiyaphum | 0.613 | 0.664∗ | 0.662 | 0.663 | 0.009 | 0.011 | 0.008 |
| Chanthaburi | 0.539 | 0.551 | 0.551 | 0.552∗ | -0.034 | -0.033 | -0.035 |
| Chiang Mai | 0.704 | 0.769∗ | 0.769 | 0.769 | -0.062 | -0.062 | -0.062 |
| Chiang Rai | 0.514 | 0.606∗ | 0.606 | 0.606 | -0.058 | -0.057 | -0.056 |
| Chon Buri | 0.61 | 0.642 | 0.644∗ | 0.642 | 0.002 | 0.004 | 0.006 |
| Chumphon | 0.37 | 0.388 | 0.39∗ | 0.387 | -0.01 | -0.01 | 0.006 |
| Kalasin | 0.513 | 0.553 | 0.55 | 0.555∗ | 0.352 | 0.356 | 0.353 |
| Kamphaeng Phet | 0.495 | 0.54 | 0.54 | 0.543∗ | -0.066 | -0.066 | -0.066 |
| Kanchanaburi | 0.357 | 0.416∗ | 0.416 | 0.416 | -0.064 | -0.063 | -0.064 |
| Khon Kaen | 0.701 | 0.753∗ | 0.75 | 0.753 | -0.062 | -0.061 | -0.062 |
| Krabi | 0.55 | 0.608 | 0.609∗ | 0.608 | 0.057 | 0.049 | 0.057 |
| Lampang | 0.603 | 0.658 | 0.658 | 0.659∗ | 0.013 | 0.017 | 0.02 |
| Lamphun | 0.648 | 0.676 | 0.677∗ | 0.677 | 0.328 | 0.331 | 0.333 |
| Loei | 0.763 | 0.783 | 0.782 | 0.784∗ | 0.302 | 0.309 | 0.306 |
| Lop Buri | 0.56 | 0.601∗ | 0.6 | 0.6 | 0.098 | 0.101 | 0.093 |
| Mae Hong Son | 0.322 | 0.423∗ | 0.423 | 0.423 | 0.005 | 0.006 | 0.004 |
| Maha Sarakham | 0.648 | 0.681 | 0.678 | 0.682∗ | 0.007 | 0.011 | 0.008 |
| Mukdahan | 0.518 | 0.54∗ | 0.539 | 0.54 | 0.409 | 0.411 | 0.407 |
| Nakhon Nayok | 0.364 | 0.388 | 0.39∗ | 0.387 | 0.304 | 0.305 | 0.314 |
| Nakhon Pathom | 0.535 | 0.562∗ | 0.562 | 0.561 | -0.066 | -0.066 | -0.066 |
| Nakhon Phanom | 0.439 | 0.504∗ | 0.503 | 0.503 | 0.216 | 0.221 | 0.214 |
| Nakhon Ratchasima | 0.469 | 0.549∗ | 0.549 | 0.549 | 0.044 | 0.035 | 0.039 |
| Nakhon Sawan | 0.539 | 0.589∗ | 0.589 | 0.589 | -0.039 | -0.039 | -0.038 |
| Nakhon Si Thammarat | 0.641 | 0.685 | 0.686 | 0.687∗ | 0.002 | -0.004 | 0 |
| Nan | 0.72 | 0.745∗ | 0.744 | 0.745 | 0.234 | 0.234 | 0.235 |
| Narathiwat | 0.637 | 0.665∗ | 0.663 | 0.664 | 0.468 | 0.468 | 0.469 |
| Nong Bua Lam Phu | 0.501 | 0.542 | 0.541 | 0.544∗ | 0.25 | 0.254 | 0.251 |
| Nong Khai | 0.548 | 0.613∗ | 0.613 | 0.613 | 0.286 | 0.293 | 0.29 |
| Nonthaburi | 0.483 | 0.487∗ | 0.486 | 0.487 | 0.148 | 0.15 | 0.149 |
| Pathum Thani | 0.645 | 0.677∗ | 0.676 | 0.676 | 0.025 | 0.029 | 0.022 |
| Pattani | 0.608 | 0.649 | 0.65∗ | 0.649 | 0.504 | 0.504 | 0.504 |
| Phangnga | 0.493 | 0.549∗ | 0.549 | 0.549 | 0.367 | 0.366 | 0.367 |
| Phatthalung | 0.465 | 0.529 | 0.528 | 0.53∗ | 0.158 | 0.162 | 0.159 |
| Phayao | 0.526 | 0.572 | 0.571 | 0.573∗ | 0.064 | 0.067 | 0.066 |
| Phetchabun | 0.608 | 0.647∗ | 0.646 | 0.647 | -0.009 | -0.006 | -0.011 |
| Phetchaburi | 0.611 | 0.617 | 0.618∗ | 0.617 | 0.159 | 0.163 | 0.191 |
| Phichit | 0.387 | 0.418 | 0.417 | 0.422∗ | 0.08 | 0.081 | 0.077 |

| | | | | | | | |
|---|---|---|---|---|---|---|---|
| Phitsanulok | 0.657 | 0.692 | 0.693* | 0.692 | 0.08 | 0.085 | 0.085 |
| Phra Nakhon Si Ayutthaya | 0.547 | 0.594* | 0.594 | 0.594 | 0.02 | 0.023 | 0.02 |
| Phrae | 0.768 | 0.797* | 0.797 | 0.797 | 0.437 | 0.442 | 0.442 |
| Phuket | 0.728 | 0.749* | 0.749 | 0.749 | 0.603 | 0.601 | 0.602 |
| Prachin Buri | 0.398 | 0.424 | 0.425* | 0.424 | -0.022 | -0.021 | -0.023 |
| Prachuap Khiri Khan | 0.698 | 0.707 | 0.708* | 0.707 | 0.183 | 0.187 | 0.204 |
| Ranong | 0.529* | 0.527 | 0.527 | 0.527 | 0.489 | 0.487 | 0.487 |
| Ratchaburi | 0.378 | 0.432 | 0.434* | 0.432 | -0.056 | -0.056 | -0.057 |
| Rayong | 0.732 | 0.758* | 0.758 | 0.758 | -0.015 | -0.012 | -0.011 |
| Roi Et | 0.795 | 0.821 | 0.821 | 0.822* | -0.022 | -0.019 | -0.022 |
| Sa Kaeo | 0.428 | 0.455* | 0.455 | 0.455 | 0.364 | 0.366 | 0.367 |
| Sakon Nakhon | 0.733 | 0.761 | 0.761 | 0.762* | 0.552 | 0.555 | 0.552 |
| Samut Prakan | 0.76 | 0.764* | 0.764 | 0.764 | -0.063 | -0.062 | -0.062 |
| Samut Sakhon | 0.52 | 0.542* | 0.542 | 0.542 | 0.329 | 0.331 | 0.338 |
| Samut Songkhram | 0.369 | 0.371* | 0.371 | 0.371 | 0.26 | 0.26 | 0.261 |
| Saraburi | 0.492 | 0.531 | 0.529 | 0.532* | 0.149 | 0.153 | 0.146 |
| Satun | 0.484 | 0.535* | 0.535 | 0.534 | 0.396 | 0.4 | 0.4 |
| Si Sa Ket | 0.706 | 0.759 | 0.759 | 0.76* | -0.055 | -0.056 | -0.055 |
| Sing Buri | 0.56 | 0.574 | 0.575* | 0.575 | 0.465 | 0.464 | 0.465 |
| Songkhla | 0.573 | 0.644 | 0.644 | 0.645* | -0.032 | -0.031 | -0.032 |
| Sukhothai | 0.482 | 0.527 | 0.528* | 0.528 | 0.079 | 0.081 | 0.079 |
| Suphan Buri | 0.5 | 0.521 | 0.522 | 0.524* | -0.026 | -0.027 | -0.027 |
| Surat Thani | 0.513 | 0.595 | 0.594 | 0.596* | -0.066 | -0.065 | -0.066 |
| Surin | 0.292 | 0.384 | 0.384 | 0.385* | -0.054 | -0.054 | -0.053 |
| Tak | 0.598 | 0.669* | 0.668 | 0.669 | -0.046 | -0.047 | -0.048 |
| Trang | 0.498 | 0.558 | 0.559* | 0.559 | 0.05 | 0.045 | 0.049 |
| Trat | 0.539* | 0.535 | 0.535 | 0.535 | 0.322 | 0.317 | 0.32 |
| Ubon Ratchathani | 0.476 | 0.549 | 0.548 | 0.55* | -0.056 | -0.056 | -0.055 |
| Udon Thani | 0.475 | 0.549* | 0.546 | 0.547 | -0.022 | -0.019 | -0.02 |
| Uthai Thani | 0.377 | 0.447 | 0.448 | 0.45* | -0.006 | -0.01 | -0.009 |
| Uttaradit | 0.523 | 0.581* | 0.578 | 0.58 | 0.088 | 0.092 | 0.089 |
| Yala | 0.647 | 0.68 | 0.681* | 0.68 | 0.368 | 0.378 | 0.367 |
| Yasothon | 0.692 | 0.724 | 0.724 | 0.725* | 0.427 | 0.433 | 0.428 |

Table 4: Adjusted $R^2$ for pneumonia case counts

| | AR | LASSO-MIDAS | | | AR-MIDAS | | |
|---|---|---|---|---|---|---|---|
| | | Free | Down | Hump | Free | Down | Hump |
| Amnat Charoen | 0.55 | 0.566* | 0.56 | 0.566 | 0.531 | 0.531 | 0.53 |
| Ang Thong | 0.54 | 0.552 | 0.551 | 0.553* | 0.457 | 0.457 | 0.455 |
| Bangkok | 0.633 | 0.657* | 0.657 | 0.657 | -0.064 | -0.064 | -0.065 |
| Buri Ram | 0.557 | 0.567 | 0.568* | 0.568 | -0.065 | -0.065 | -0.065 |
| Chachoengsao | 0.68* | 0.68 | 0.68 | 0.68 | 0.393 | 0.397 | 0.397 |
| Chai Nat | 0.341 | 0.35 | 0.351 | 0.353* | 0.312 | 0.311 | 0.312 |
| Chaiyaphum | 0.603 | 0.61 | 0.609 | 0.611* | 0.165 | 0.168 | 0.162 |
| Chanthaburi | 0.604 | 0.632 | 0.633* | 0.632 | -0.048 | -0.047 | -0.047 |
| Chiang Mai | 0.667 | 0.709 | 0.71* | 0.709 | -0.062 | -0.063 | -0.062 |
| Chiang Rai | 0.498 | 0.526* | 0.526 | 0.526 | -0.065 | -0.065 | -0.065 |
| Chon Buri | 0.615 | 0.625* | 0.625 | 0.624 | -0.043 | -0.043 | -0.042 |
| Chumphon | 0.531 | 0.543 | 0.544* | 0.543 | 0.395 | 0.397 | 0.405 |
| Kalasin | 0.513 | 0.546* | 0.545 | 0.546 | 0.466 | 0.469 | 0.467 |
| Kamphaeng Phet | 0.445 | 0.454 | 0.455* | 0.454 | 0.009 | 0.01 | 0.009 |
| Kanchanaburi | 0.475 | 0.512* | 0.512 | 0.512 | -0.011 | -0.01 | -0.009 |
| Khon Kaen | 0.553 | 0.566 | 0.566 | 0.567* | -0.044 | -0.04 | -0.046 |
| Krabi | 0.34 | 0.362* | 0.362 | 0.362 | 0.293 | 0.292 | 0.292 |
| Lampang | 0.478 | 0.511* | 0.511 | 0.51 | -0.06 | -0.058 | -0.061 |
| Lamphun | 0.591 | 0.608* | 0.608 | 0.608 | 0.33 | 0.331 | 0.331 |
| Loei | 0.45 | 0.472 | 0.472 | 0.474* | 0.32 | 0.322 | 0.318 |
| Lop Buri | 0.454 | 0.476* | 0.475 | 0.475 | 0.17 | 0.178 | 0.175 |
| Mae Hong Son | 0.445 | 0.473* | 0.473 | 0.473 | 0.399 | 0.399 | 0.402 |
| Maha Sarakham | 0.6 | 0.608 | 0.607 | 0.609* | 0.433 | 0.435 | 0.433 |
| Mukdahan | 0.468 | 0.504 | 0.505* | 0.504 | 0.417 | 0.415 | 0.416 |
| Nakhon Nayok | 0.254 | 0.269* | 0.269 | 0.269 | 0.259 | 0.26 | 0.26 |
| Nakhon Pathom | 0.643 | 0.654* | 0.654 | 0.653 | -0.058 | -0.055 | -0.059 |
| Nakhon Phanom | 0.59 | 0.652 | 0.653* | 0.652 | 0.409 | 0.411 | 0.408 |
| Nakhon Ratchasima | 0.662 | 0.671* | 0.67 | 0.671 | -0.065 | -0.064 | -0.065 |
| Nakhon Sawan | 0.665 | 0.67 | 0.671* | 0.671 | -0.046 | -0.046 | -0.046 |
| Nakhon Si Thammarat | 0.7 | 0.713* | 0.713 | 0.713 | 0.073 | 0.064 | 0.072 |
| Nan | 0.667 | 0.677* | 0.677 | 0.677 | 0.473 | 0.472 | 0.478 |
| Narathiwat | 0.528 | 0.557* | 0.557 | 0.557 | 0.517 | 0.519 | 0.517 |
| Nong Bua Lam Phu | 0.416* | 0.415 | 0.413 | 0.416 | 0.372 | 0.372 | 0.371 |
| Nong Khai | 0.362 | 0.383* | 0.382 | 0.383 | 0.056 | 0.063 | 0.054 |
| Nonthaburi | 0.512 | 0.516* | 0.516 | 0.516 | 0.209 | 0.214 | 0.211 |
| Pathum Thani | 0.637 | 0.639* | 0.639 | 0.638 | 0.313 | 0.318 | 0.314 |
| Pattani | 0.275 | 0.287* | 0.287 | 0.287 | 0.28 | 0.279 | 0.279 |
| Phangnga | 0.705 | 0.712* | 0.712 | 0.712 | 0.65 | 0.649 | 0.652 |
| Phatthalung | 0.545 | 0.549 | 0.55* | 0.549 | 0.489 | 0.489 | 0.489 |
| Phayao | 0.611 | 0.633* | 0.633 | 0.633 | 0.111 | 0.112 | 0.111 |
| Phetchabun | 0.602 | 0.605 | 0.605 | 0.606* | 0.357 | 0.362 | 0.357 |
| Phetchaburi | 0.465 | 0.466 | 0.467* | 0.465 | 0.249 | 0.247 | 0.264 |
| Phichit | 0.41 | 0.432 | 0.434* | 0.432 | 0.235 | 0.234 | 0.235 |

| | | | | | | | |
|---|---|---|---|---|---|---|---|
| Phitsanulok | 0.363 | 0.43 | 0.431* | 0.429 | -0.065 | -0.065 | -0.064 |
| Phra Nakhon Si Ayutthaya | 0.621 | 0.634* | 0.632 | 0.633 | 0.151 | 0.158 | 0.149 |
| Phrae | 0.398* | 0.398 | 0.398 | 0.398 | 0.346 | 0.346 | 0.346 |
| Phuket | 0.441 | 0.465* | 0.465 | 0.465 | 0.263 | 0.26 | 0.263 |
| Prachin Buri | 0.58 | 0.581* | 0.581 | 0.581 | 0.463 | 0.461 | 0.462 |
| Prachuap Khiri Khan | 0.695 | 0.7 | 0.701* | 0.7 | 0.544 | 0.543 | 0.559 |
| Ranong | 0.324 | 0.352* | 0.352 | 0.352 | 0.344 | 0.345 | 0.344 |
| Ratchaburi | 0.449 | 0.453 | 0.454* | 0.452 | 0.007 | 0.007 | 0.014 |
| Rayong | 0.661 | 0.678* | 0.678 | 0.678 | -0.066 | -0.066 | -0.066 |
| Roi Et | 0.709 | 0.722 | 0.722 | 0.723* | 0.565 | 0.565 | 0.563 |
| Sa Kaeo | 0.643* | 0.642 | 0.643 | 0.642 | 0.615 | 0.615 | 0.616 |
| Sakon Nakhon | 0.526 | 0.571 | 0.572* | 0.572 | 0.49 | 0.492 | 0.491 |
| Samut Prakan | 0.627 | 0.64 | 0.64 | 0.641* | -0.054 | -0.054 | -0.053 |
| Samut Sakhon | 0.426 | 0.427* | 0.427 | 0.426 | 0.123 | 0.122 | 0.129 |
| Samut Songkhram | 0.526* | 0.523 | 0.523 | 0.523 | 0.503 | 0.503 | 0.503 |
| Saraburi | 0.516* | 0.513 | 0.513 | 0.514 | 0.445 | 0.445 | 0.443 |
| Satun | 0.485* | 0.485 | 0.485 | 0.484 | 0.472 | 0.472 | 0.472 |
| Si Sa Ket | 0.652 | 0.665 | 0.666* | 0.666 | 0.352 | 0.351 | 0.353 |
| Sing Buri | 0.357 | 0.362* | 0.361 | 0.362 | 0.327 | 0.328 | 0.327 |
| Songkhla | 0.507 | 0.526* | 0.526 | 0.526 | 0.316 | 0.32 | 0.313 |
| Sukhothai | 0.477 | 0.485 | 0.486* | 0.484 | 0.143 | 0.142 | 0.146 |
| Suphan Buri | 0.499 | 0.5* | 0.499 | 0.5 | 0.165 | 0.168 | 0.165 |
| Surat Thani | 0.616 | 0.629* | 0.628 | 0.629 | -0.05 | -0.048 | -0.047 |
| Surin | 0.663 | 0.674 | 0.676* | 0.675 | 0.089 | 0.091 | 0.095 |
| Tak | 0.581 | 0.607* | 0.607 | 0.606 | 0.398 | 0.399 | 0.404 |
| Trang | 0.354 | 0.389* | 0.389 | 0.389 | 0.134 | 0.138 | 0.141 |
| Trat | 0.593 | 0.613* | 0.612 | 0.612 | 0.488 | 0.488 | 0.489 |
| Ubon Ratchathani | 0.637 | 0.661 | 0.662* | 0.661 | -0.058 | -0.057 | -0.056 |
| Udon Thani | 0.57 | 0.572* | 0.572 | 0.572 | 0.261 | 0.267 | 0.262 |
| Uthai Thani | 0.368 | 0.395 | 0.396* | 0.395 | 0.328 | 0.328 | 0.329 |
| Uttaradit | 0.519 | 0.557 | 0.558* | 0.556 | 0.063 | 0.065 | 0.061 |
| Yala | 0.507 | 0.515* | 0.513 | 0.513 | 0.511 | 0.508 | 0.51 |
| Yasothon | 0.585 | 0.605* | 0.604 | 0.604 | 0.474 | 0.476 | 0.474 |

# 4 Model assessment metrics for case counts per 100,000

Table 5: Deviance Information Criterion for URTI case counts per 100,000

| | AR | LASSO-MIDAS | | | AR-MIDAS | | |
|---|---|---|---|---|---|---|---|
| | | Free | Down | Hump | Free | Down | Hump |
| Amnat Charoen | 2399.98∗ | 2401.44 | 2401.41 | 2401.28 | 2437.19 | 2435.32 | 2437.5 |
| Ang Thong | 2263.39 | 2246.86∗ | 2246.98 | 2247.89 | 2390.44 | 2388.09 | 2388.85 |
| Bangkok | 3085.42 | 3067.57 | 3067.51∗ | 3067.62 | 3385.31 | 3385.12 | 3385.94 |
| Buri Ram | 2820.78 | 2792.71∗ | 2793.13 | 2792.72 | 3024.04 | 3023.58 | 3023.94 |
| Chachoengsao | 2612.77 | 2611.85 | 2611.69∗ | 2612.17 | 2757.46 | 2755.94 | 2756.9 |
| Chai Nat | 1819.43 | 1806.75 | 1806.03 | 1805.92∗ | 1858.21 | 1858.36 | 1858.02 |
| Chaiyaphum | 2653.1 | 2629.52∗ | 2630.91 | 2630.66 | 2865.01 | 2863.63 | 2865.23 |
| Chanthaburi | 2316.33∗ | 2317.91 | 2317.85 | 2317.57 | 2601.75 | 2599.72 | 2601.64 |
| Chiang Mai | 2880.09 | 2833.58∗ | 2834.22 | 2834.37 | 3300.84 | 3297.29 | 3299.84 |
| Chiang Rai | 2855.53 | 2818.32∗ | 2818.89 | 2818.34 | 3208.35 | 3206.8 | 3207.81 |
| Chon Buri | 2587.8 | 2576.31 | 2575.73∗ | 2576.54 | 2801.84 | 2799.71 | 2799.92 |
| Chumphon | 2222.89 | 2222.57 | 2222.26∗ | 2223.05 | 2378.16 | 2375.06 | 2365.23 |
| Kalasin | 2324.24 | 2310.77 | 2312.08 | 2309.78∗ | 2389.48 | 2387.78 | 2389.61 |
| Kamphaeng Phet | 2521.93 | 2508.43 | 2508.75 | 2507.61∗ | 2784.28 | 2783.01 | 2784.14 |
| Kanchanaburi | 2544.53 | 2529.83∗ | 2530.02 | 2530.25 | 2736.19 | 2733.73 | 2735.62 |
| Khon Kaen | 2972.96 | 2940.35 | 2941.97 | 2939.97∗ | 3275.8 | 3273.96 | 3275.74 |
| Krabi | 2306.17 | 2282.45 | 2282.32∗ | 2282.63 | 2483.59 | 2485.28 | 2484.52 |
| Lampang | 2481.42 | 2454.8∗ | 2454.97 | 2455.01 | 2734.45 | 2732.48 | 2734.93 |
| Lamphun | 2050.33 | 2037.91 | 2037.05∗ | 2037.59 | 2207.12 | 2205.21 | 2205.38 |
| Loei | 2444.42 | 2432.04 | 2431.9 | 2430.48∗ | 2693.57 | 2692.47 | 2693.66 |
| Lop Buri | 2492.18 | 2477.43∗ | 2478.41 | 2478.24 | 2647.8 | 2646.88 | 2647.91 |
| Mae Hong Son | 2336.18 | 2306.68 | 2306.43 | 2306.39∗ | 2487.53 | 2483.48 | 2486.04 |
| Maha Sarakham | 2765.79 | 2751.3 | 2753.96 | 2751.16∗ | 2992.24 | 2990.99 | 2992.69 |
| Mukdahan | 2291.92 | 2287.38∗ | 2287.86 | 2287.85 | 2342.42 | 2340.81 | 2342.27 |
| Nakhon Nayok | 1972.86 | 1970.59 | 1969.89∗ | 1970.53 | 1994.05 | 1993.55 | 1991.15 |
| Nakhon Pathom | 2450.16 | 2444.51∗ | 2444.61 | 2445.13 | 2694.68 | 2693.16 | 2694.17 |
| Nakhon Phanom | 2479.37 | 2457.98∗ | 2458.3 | 2458.29 | 2552.69 | 2550.57 | 2552.42 |
| Nakhon Ratchasima | 2796.86 | 2768.67∗ | 2768.84 | 2768.8 | 2961.3 | 2961.12 | 2961.53 |
| Nakhon Sawan | 2417.23 | 2398.96 | 2398.9∗ | 2399.26 | 2656.88 | 2655.78 | 2656.88 |
| Nakhon Si Thammarat | 2553.78 | 2532.49 | 2532.33 | 2531.3∗ | 2790.62 | 2792.27 | 2791.56 |
| Nan | 2323.33 | 2309.23∗ | 2309.37 | 2309.29 | 2554.22 | 2551.88 | 2553.46 |
| Narathiwat | 2453.72 | 2444.51∗ | 2445.72 | 2444.98 | 2560.65 | 2561.32 | 2560.53 |
| Nong Bua Lam Phu | 2143.97 | 2131.53 | 2131.9 | 2131.34∗ | 2235.42 | 2233.67 | 2235.79 |
| Nong Khai | 2437.12 | 2410.18 | 2410.01 | 2409.94∗ | 2539.68 | 2538.71 | 2539.91 |
| Nonthaburi | 2198.52∗ | 2203.73 | 2204.33 | 2203.76 | 2317.07 | 2316 | 2317.27 |
| Pathum Thani | 2628.6 | 2615.29∗ | 2616.06 | 2615.59 | 2848.58 | 2847.28 | 2848.99 |
| Pattani | 2267.98 | 2249.66 | 2249.47 | 2248.28∗ | 2332.41 | 2332.14 | 2332.28 |
| Phangnga | 2094.51 | 2074.96 | 2074.74 | 2074.57∗ | 2146.95 | 2146.84 | 2147.26 |
| Phatthalung | 2290.18 | 2269.59 | 2269.48 | 2269.17∗ | 2391.65 | 2390.56 | 2393.23 |
| Phayao | 2371.37 | 2356.08 | 2356.69 | 2355.98∗ | 2556.16 | 2553.48 | 2555.56 |
| Phetchabun | 2606.2 | 2590∗ | 2590.37 | 2590.3 | 2816.84 | 2815.29 | 2816.25 |

| | | | | | | | |
|---|---|---|---|---|---|---|---|
| Phetchaburi | 2208.46∗ | 2211.72 | 2211.05 | 2211.9 | 2406.56 | 2404.57 | 2392.19 |
| Phichit | 2267.23 | 2262.06 | 2262.62 | 2261.07∗ | 2366.52 | 2366.58 | 2367.48 |
| Phitsanulok | 2401.11 | 2383.16 | 2382.9∗ | 2383.72 | 2625.71 | 2623.99 | 2624.7 |
| Phra Nakhon Si Ayutthaya | 2630.45 | 2613.64∗ | 2614.1 | 2614.46 | 2803.28 | 2801.41 | 2803.02 |
| Phrae | 2155.97 | 2134.22∗ | 2134.22 | 2134.4 | 2358.24 | 2355.1 | 2354.4 |
| Phuket | 2185.31 | 2173.9 | 2173.78∗ | 2173.93 | 2280.35 | 2280.79 | 2280.78 |
| Prachin Buri | 2647.67 | 2646.13 | 2645.86∗ | 2646.08 | 2762.45 | 2761.79 | 2761.82 |
| Prachuap Khiri Khan | 2245.95 | 2246.07 | 2245.28∗ | 2245.87 | 2485.4 | 2482.39 | 2477.02 |
| Ranong | 1842.95∗ | 1849.46 | 1849.33 | 1849.34 | 1864.79 | 1865.22 | 1865.54 |
| Ratchaburi | 2367.62 | 2355.58 | 2354.64∗ | 2355.78 | 2587.43 | 2585.82 | 2581.63 |
| Rayong | 2416.26 | 2402.16 | 2401.7∗ | 2402.67 | 2746.52 | 2742.91 | 2742.04 |
| Roi Et | 2717.51 | 2694.79 | 2693.95 | 2693.83∗ | 3071.97 | 3070.87 | 3072.05 |
| Sa Kaeo | 1934.79 | 1930.61 | 1930.53 | 1930.38∗ | 1961.62 | 1960.54 | 1960.45 |
| Sakon Nakhon | 2359.63 | 2340.95 | 2341.37 | 2340.25∗ | 2516.09 | 2513.41 | 2515.93 |
| Samut Prakan | 2666.55∗ | 2671.43 | 2670.7 | 2672.06 | 3029.44 | 3029.38 | 3029.61 |
| Samut Sakhon | 2059.69 | 2055.51 | 2055.02∗ | 2055.83 | 2139.41 | 2139.02 | 2137.12 |
| Samut Songkhram | 1760.02∗ | 1764.97 | 1765.2 | 1765.16 | 1806.3 | 1805.66 | 1804.95 |
| Saraburi | 2486.42 | 2476.36 | 2476.71 | 2476.19∗ | 2605.44 | 2603.86 | 2605.46 |
| Satun | 1997.06 | 1980.35 | 1980.14∗ | 1980.6 | 2034.49 | 2032.77 | 2033.46 |
| Si Sa Ket | 2922.76 | 2888.02 | 2887.5 | 2886.42∗ | 3229.17 | 3229.47 | 3229.76 |
| Sing Buri | 1888.31 | 1888.25 | 1887.9∗ | 1888.04 | 1934.52 | 1934.27 | 1934.63 |
| Songkhla | 2634.57 | 2601.44 | 2601.71 | 2601.09∗ | 2838.64 | 2837.09 | 2838.45 |
| Sukhothai | 2193.09 | 2179.28 | 2178.62 | 2178.41∗ | 2356 | 2354.37 | 2355.6 |
| Suphan Buri | 2360.89 | 2358.32 | 2357.88 | 2356.82∗ | 2560.35 | 2558.36 | 2560.35 |
| Surat Thani | 2664.38 | 2632.12 | 2632.38 | 2631.93∗ | 2951.26 | 2954.86 | 2952.22 |
| Surin | 2851.13 | 2827.82 | 2827.77 | 2826.96∗ | 2971.89 | 2971.52 | 2972.22 |
| Tak | 2460.03 | 2423.37∗ | 2424.3 | 2424.52 | 2747.59 | 2744.61 | 2746.18 |
| Trang | 2337.04 | 2315.85∗ | 2315.89 | 2316.06 | 2488.22 | 2488.79 | 2488.43 |
| Trat | 1997.38∗ | 2005.13 | 2005.45 | 2005.13 | 2112.89 | 2112.61 | 2112.6 |
| Ubon Ratchathani | 3186.3 | 3162.96∗ | 3163.33 | 3163 | 3385.94 | 3385.53 | 3386.51 |
| Udon Thani | 2679.03 | 2652.96∗ | 2654.23 | 2654.58 | 2832.72 | 2830.82 | 2832.98 |
| Uthai Thani | 2109.75 | 2090.11 | 2088.92 | 2088.68∗ | 2276.39 | 2274.94 | 2277.2 |
| Uttaradit | 2409.85 | 2387.63∗ | 2389.34 | 2388.4 | 2559.84 | 2558.58 | 2560.17 |
| Yala | 2453.98 | 2439.8 | 2439.4∗ | 2440.18 | 2603.22 | 2600.94 | 2602.65 |
| Yasothon | 2441.57 | 2423.18∗ | 2424.12 | 2423.35 | 2600.36 | 2599.17 | 2600.18 |

Table 6: Deviance Information Criterion for pneumonia case counts per 100,000

| | AR | LASSO-MIDAS | | | AR-MIDAS | | |
|---|---|---|---|---|---|---|---|
| | | Free | Down | Hump | Free | Down | Hump |
| Amnat Charoen | 1806.89 | 1804.22* | 1807.31 | 1804.89 | 1818.72 | 1818.58 | 1818.58 |
| Ang Thong | 2126.96* | 2127.49 | 2127.93 | 2127.58 | 2173.11 | 2172.56 | 2173.03 |
| Bangkok | 3986.24 | 3981.05 | 3980.83* | 3980.94 | 4221.03 | 4220.58 | 4221.51 |
| Buri Ram | 2985.18* | 2988.73 | 2988.18 | 2987.86 | 3177.24 | 3176.88 | 3177.41 |
| Chachoengsao | 2625.14* | 2632.43 | 2632.17 | 2631.97 | 2839 | 2839.19 | 2839.4 |
| Chai Nat | 1914.15* | 1916.36 | 1915.78 | 1915.69 | 1926.27 | 1926.11 | 1926.35 |
| Chaiyaphum | 2742.46* | 2746.12 | 2746.98 | 2745.66 | 2931.52 | 2931.06 | 2932.22 |
| Chanthaburi | 2747.28 | 2740.36 | 2739.58* | 2739.83 | 2972.94 | 2971.37 | 2972.19 |
| Chiang Mai | 3364.6 | 3345.32 | 3344.96* | 3345.02 | 3637.05 | 3635.94 | 3637.1 |
| Chiang Rai | 2963.27 | 2958.85* | 2958.85 | 2959.16 | 3133.73 | 3132.91 | 3133.83 |
| Chon Buri | 2870.39* | 2874.1 | 2873.6 | 2874.07 | 3089.19 | 3089.15 | 3089.35 |
| Chumphon | 2362.27* | 2363.95 | 2364.32 | 2363.86 | 2448.36 | 2448.32 | 2446.69 |
| Kalasin | 2270.08 | 2260.21 | 2260.37 | 2260.07* | 2307.03 | 2306.17 | 2306.96 |
| Kamphaeng Phet | 2699.58* | 2703.08 | 2702.9 | 2703.21 | 2826.39 | 2826.07 | 2826.67 |
| Kanchanaburi | 2762.53 | 2754.28 | 2753.97 | 2753.79* | 2906.84 | 2906.25 | 2906.68 |
| Khon Kaen | 2947.24* | 2948.51 | 2948.88 | 2948.77 | 3130.14 | 3129.89 | 3130.78 |
| Krabi | 2251.44 | 2250.36 | 2250.51 | 2250.2* | 2271.93 | 2271.89 | 2272.17 |
| Lampang | 2939.32 | 2933.17* | 2933.28 | 2933.45 | 3095.75 | 3095.08 | 3096.12 |
| Lamphun | 2529.5 | 2526.36 | 2526.03* | 2526.21 | 2663.03 | 2662.88 | 2663.59 |
| Loei | 2322.68 | 2319.66 | 2319.9 | 2318.82* | 2379.59 | 2379.16 | 2379.2 |
| Lop Buri | 2655.91 | 2654.04* | 2654.88 | 2655.24 | 2763.5 | 2763.26 | 2763.81 |
| Mae Hong Son | 2114.04 | 2107.84* | 2107.84 | 2108.45 | 2137.94 | 2137.94 | 2136.92 |
| Maha Sarakham | 2385.18* | 2387.04 | 2387.37 | 2386.43 | 2496.89 | 2495.76 | 2496.81 |
| Mukdahan | 2387.41 | 2377.25 | 2376.93 | 2376.85* | 2435.1 | 2435.31 | 2436 |
| Nakhon Nayok | 1924.3* | 1924.49 | 1924.61 | 1924.96 | 1925.13 | 1925.05 | 1924.73 |
| Nakhon Pathom | 2956.73* | 2959.04 | 2958.47 | 2959.07 | 3196.64 | 3195.45 | 3196.69 |
| Nakhon Phanom | 2472.4 | 2443.05* | 2443.26 | 2443.26 | 2589.35 | 2589.63 | 2590.14 |
| Nakhon Ratchasima | 3387.46* | 3390.42 | 3391.48 | 3390.47 | 3639.5 | 3639.16 | 3640.18 |
| Nakhon Sawan | 2873.81* | 2878.13 | 2877.98 | 2877.53 | 3122.55 | 3122.62 | 3123.31 |
| Nakhon Si Thammarat | 2668.02 | 2666.24 | 2666.19 | 2665.82* | 2921.73 | 2922.15 | 2922.52 |
| Nan | 2393.24 | 2392.42* | 2392.49 | 2392.69 | 2530.39 | 2531.76 | 2531.53 |
| Narathiwat | 2262.71 | 2254.76 | 2254.64* | 2254.85 | 2282.77 | 2281.93 | 2282.77 |
| Nong Bua Lam Phu | 2103.45* | 2109.1 | 2109.85 | 2109.26 | 2126.66 | 2125.52 | 2126.79 |
| Nong Khai | 2843.29 | 2843.15 | 2843.12 | 2843.07* | 2937.61 | 2937.33 | 2938.39 |
| Nonthaburi | 2616.73* | 2621.95 | 2622.13 | 2622.3 | 2742.74 | 2742.69 | 2743.54 |
| Pathum Thani | 2585.98* | 2591.42 | 2591.52 | 2591.97 | 2771.96 | 2771.91 | 2772.82 |
| Pattani | 1901.93* | 1902.89 | 1902.73 | 1903.46 | 1902.83 | 1903.03 | 1903.21 |
| Phangnga | 2047.39* | 2048.1 | 2047.68 | 2048.16 | 2094.24 | 2094.83 | 2093.42 |
| Phatthalung | 2361.71* | 2365.84 | 2365.6 | 2365.85 | 2417.22 | 2416.82 | 2417.67 |
| Phayao | 2716.57 | 2712.59 | 2712.41* | 2712.76 | 2908.69 | 2908.23 | 2909.33 |
| Phetchabun | 2477.65* | 2482.03 | 2482.3 | 2482.09 | 2624.47 | 2623.64 | 2624.71 |
| Phetchaburi | 2417.68* | 2423.89 | 2423.25 | 2424.16 | 2505.05 | 2505.98 | 2503.88 |
| Phichit | 2501.94 | 2499.71 | 2499.25* | 2499.75 | 2574.13 | 2573.83 | 2574.37 |

| | | | | | | | |
|---|---|---|---|---|---|---|---|
| Phitsanulok | 3069.9 | 3054.2 | 3053.11∗ | 3054.15 | 3187.51 | 3187.21 | 3187.87 |
| Phra Nakhon Si Ayutthaya | 2661.68∗ | 2662.17 | 2663.1 | 2662.38 | 2852.17 | 2851.44 | 2851.96 |
| Phrae | 2418.32∗ | 2424.03 | 2424.1 | 2424.11 | 2455.38 | 2455.32 | 2455.79 |
| Phuket | 2561.82 | 2560 | 2559.94 | 2559.82∗ | 2647.76 | 2648.01 | 2648.13 |
| Prachin Buri | 2392.25∗ | 2397.84 | 2397.94 | 2398.23 | 2484.95 | 2484.94 | 2485.02 |
| Prachuap Khiri Khan | 2419.17∗ | 2422.77 | 2422.3 | 2422.68 | 2565.22 | 2565.09 | 2564.23 |
| Ranong | 1612.2 | 1608.23 | 1607.71∗ | 1608.4 | 1609.05 | 1609.04 | 1609.34 |
| Ratchaburi | 2655.92∗ | 2661.55 | 2661.03 | 2661.9 | 2786.34 | 2786.07 | 2785.6 |
| Rayong | 2990.56 | 2988.78 | 2988.66∗ | 2988.95 | 3256.67 | 3255.97 | 3257.01 |
| Roi Et | 2412.9 | 2409.03 | 2408.95 | 2408.57∗ | 2569.28 | 2569.45 | 2570.37 |
| Sa Kaeo | 1849.51∗ | 1855.72 | 1855.56 | 1855.99 | 1873.06 | 1872.92 | 1871.85 |
| Sakon Nakhon | 2201.9 | 2185.63 | 2185.14∗ | 2185.16 | 2230.29 | 2230.29 | 2230.23 |
| Samut Prakan | 2914.53∗ | 2915.68 | 2915.69 | 2915.52 | 3143.19 | 3143.21 | 3143.49 |
| Samut Sakhon | 2733.93∗ | 2741.09 | 2741.17 | 2741.42 | 2842.46 | 2842.1 | 2842.13 |
| Samut Songkhram | 1782.5∗ | 1789.55 | 1789.68 | 1789.66 | 1797.04 | 1797.44 | 1797.22 |
| Saraburi | 2275.59∗ | 2282.76 | 2282.63 | 2282.45 | 2323.15 | 2322.74 | 2323.52 |
| Satun | 1651.69∗ | 1656.54 | 1656.77 | 1656.68 | 1660.72 | 1660.73 | 1660.15 |
| Si Sa Ket | 2454.23 | 2452.1 | 2451.54∗ | 2452.2 | 2621.96 | 2622.61 | 2622.6 |
| Sing Buri | 1959.33∗ | 1963.93 | 1963.7 | 1964.1 | 1971.94 | 1971.63 | 1971.85 |
| Songkhla | 2569.13 | 2568.01∗ | 2568.39 | 2568.24 | 2684.35 | 2682.96 | 2684.2 |
| Sukhothai | 2700.34∗ | 2703.01 | 2702.28 | 2703.24 | 2820.84 | 2821.53 | 2821.76 |
| Suphan Buri | 2498.2∗ | 2504.94 | 2505.14 | 2504.47 | 2621.1 | 2620.63 | 2620.65 |
| Surat Thani | 2764.99∗ | 2766.36 | 2766.74 | 2766.11 | 2991.15 | 2990.91 | 2991.61 |
| Surin | 2793.5 | 2792.87 | 2792.23∗ | 2792.65 | 3028.47 | 3028.58 | 3029.1 |
| Tak | 2401.64 | 2393.63∗ | 2393.83 | 2394.06 | 2503.31 | 2503.12 | 2502.58 |
| Trang | 2530.96 | 2526.28∗ | 2526.3 | 2526.67 | 2604.71 | 2604.53 | 2603.59 |
| Trat | 2119.67 | 2116.49∗ | 2116.54 | 2116.89 | 2185.12 | 2184.38 | 2184.61 |
| Ubon Ratchathani | 3214.37 | 3208.85 | 3207.81∗ | 3208.42 | 3456.37 | 3456.53 | 3456.61 |
| Udon Thani | 2633.13∗ | 2638.74 | 2639.2 | 2639.11 | 2791.93 | 2791.92 | 2792.91 |
| Uthai Thani | 2097.34 | 2092.84∗ | 2093.1 | 2093.11 | 2114.39 | 2114.25 | 2114.07 |
| Uttaradit | 2680.11 | 2669.27 | 2668.64∗ | 2669.13 | 2830.86 | 2830.22 | 2831.1 |
| Yala | 1867.63∗ | 1869.55 | 1870.62 | 1870.04 | 1869.51 | 1870.21 | 1870.22 |
| Yasothon | 2291.49 | 2286.36∗ | 2287.44 | 2287.29 | 2364.07 | 2363.55 | 2364.13 |

Table 7: Adjusted $R^2$ for URTI case counts per 100,000

| | AR | LASSO-MIDAS | | | AR-MIDAS | | |
|---|---|---|---|---|---|---|---|
| | | Free | Down | Hump | Free | Down | Hump |
| Amnat Charoen | 0.278 | 0.292 | 0.293* | 0.292 | 0.137 | 0.143 | 0.137 |
| Ang Thong | 0.614 | 0.653 | 0.654* | 0.653 | 0.318 | 0.324 | 0.323 |
| Bangkok | 0.625 | 0.669* | 0.669 | 0.669 | -0.036 | -0.034 | -0.037 |
| Buri Ram | 0.555 | 0.622* | 0.622 | 0.622 | -0.035 | -0.034 | -0.033 |
| Chachoengsao | 0.493 | 0.513 | 0.514* | 0.513 | 0.044 | 0.047 | 0.045 |
| Chai Nat | 0.36 | 0.411 | 0.413* | 0.412 | 0.255 | 0.254 | 0.256 |
| Chaiyaphum | 0.613 | 0.664* | 0.662 | 0.663 | 0.009 | 0.01 | 0.008 |
| Chanthaburi | 0.539 | 0.551* | 0.551 | 0.551 | -0.035 | -0.027 | -0.036 |
| Chiang Mai | 0.704 | 0.769* | 0.769 | 0.769 | -0.059 | -0.064 | -0.061 |
| Chiang Rai | 0.514 | 0.606* | 0.605 | 0.606 | -0.057 | -0.058 | -0.057 |
| Chon Buri | 0.61 | 0.642 | 0.643* | 0.642 | 0.002 | 0.006 | 0.006 |
| Chumphon | 0.37 | 0.388 | 0.39* | 0.388 | -0.01 | -0.01 | 0.006 |
| Kalasin | 0.513 | 0.553 | 0.55 | 0.555* | 0.353 | 0.356 | 0.353 |
| Kamphaeng Phet | 0.496 | 0.54 | 0.54 | 0.542* | -0.066 | -0.066 | -0.066 |
| Kanchanaburi | 0.357 | 0.416* | 0.416 | 0.416 | -0.063 | -0.063 | -0.064 |
| Khon Kaen | 0.701 | 0.752 | 0.751 | 0.753* | -0.062 | -0.061 | -0.062 |
| Krabi | 0.55 | 0.608 | 0.609* | 0.608 | 0.057 | 0.049 | 0.054 |
| Lampang | 0.603 | 0.658 | 0.658 | 0.659* | 0.018 | 0.016 | 0.015 |
| Lamphun | 0.648 | 0.676 | 0.677* | 0.677 | 0.329 | 0.331 | 0.333 |
| Loei | 0.763 | 0.783 | 0.783 | 0.785* | 0.304 | 0.306 | 0.304 |
| Lop Buri | 0.56 | 0.601* | 0.6 | 0.6 | 0.095 | 0.099 | 0.094 |
| Mae Hong Son | 0.322 | 0.423* | 0.423 | 0.423 | 0.005 | 0.006 | 0.003 |
| Maha Sarakham | 0.648 | 0.682* | 0.678 | 0.682 | 0.006 | 0.01 | 0.007 |
| Mukdahan | 0.518 | 0.54* | 0.538 | 0.539 | 0.407 | 0.41 | 0.407 |
| Nakhon Nayok | 0.364 | 0.388 | 0.39* | 0.387 | 0.304 | 0.305 | 0.313 |
| Nakhon Pathom | 0.535 | 0.562* | 0.562 | 0.561 | -0.066 | -0.066 | -0.066 |
| Nakhon Phanom | 0.439 | 0.504* | 0.503 | 0.503 | 0.213 | 0.222 | 0.213 |
| Nakhon Ratchasima | 0.469 | 0.549* | 0.549 | 0.549 | 0.04 | 0.036 | 0.043 |
| Nakhon Sawan | 0.539 | 0.589* | 0.589 | 0.589 | -0.039 | -0.039 | -0.039 |
| Nakhon Si Thammarat | 0.641 | 0.685 | 0.686* | 0.686 | 0.002 | -0.003 | 0.001 |
| Nan | 0.72 | 0.745* | 0.744 | 0.745 | 0.231 | 0.236 | 0.234 |
| Narathiwat | 0.637 | 0.664* | 0.663 | 0.664 | 0.466 | 0.47 | 0.468 |
| Nong Bua Lam Phu | 0.501 | 0.542 | 0.542 | 0.544* | 0.25 | 0.254 | 0.249 |
| Nong Khai | 0.548 | 0.613* | 0.612 | 0.612 | 0.289 | 0.292 | 0.289 |
| Nonthaburi | 0.483 | 0.487* | 0.486 | 0.487 | 0.149 | 0.149 | 0.148 |
| Pathum Thani | 0.645 | 0.677* | 0.676 | 0.676 | 0.027 | 0.028 | 0.024 |
| Pattani | 0.608 | 0.65* | 0.649 | 0.65 | 0.503 | 0.504 | 0.504 |
| Phangnga | 0.493 | 0.549* | 0.549 | 0.549 | 0.367 | 0.367 | 0.366 |
| Phatthalung | 0.465 | 0.529 | 0.528 | 0.53* | 0.16 | 0.162 | 0.158 |
| Phayao | 0.526 | 0.572* | 0.571 | 0.572 | 0.062 | 0.067 | 0.062 |
| Phetchabun | 0.608 | 0.647* | 0.646 | 0.647 | -0.012 | -0.009 | -0.012 |
| Phetchaburi | 0.611 | 0.617 | 0.618* | 0.617 | 0.16 | 0.161 | 0.191 |
| Phichit | 0.387 | 0.418 | 0.417 | 0.422* | 0.083 | 0.079 | 0.077 |

| | | | | | | | |
|---|---|---|---|---|---|---|---|
| Phitsanulok | 0.657 | 0.692 | 0.693* | 0.692 | 0.082 | 0.085 | 0.084 |
| Phra Nakhon Si Ayutthaya | 0.547 | 0.594* | 0.594 | 0.594 | 0.019 | 0.023 | 0.02 |
| Phrae | 0.768 | 0.797* | 0.797 | 0.797 | 0.438 | 0.443 | 0.444 |
| Phuket | 0.728 | 0.749* | 0.749 | 0.749 | 0.603 | 0.601 | 0.603 |
| Prachin Buri | 0.399 | 0.424 | 0.425* | 0.424 | -0.023 | -0.021 | -0.021 |
| Prachuap Khiri Khan | 0.698 | 0.707* | 0.707 | 0.707 | 0.183 | 0.19 | 0.203 |
| Ranong | 0.529* | 0.527 | 0.527 | 0.527 | 0.488 | 0.487 | 0.488 |
| Ratchaburi | 0.378 | 0.433 | 0.434* | 0.433 | -0.056 | -0.056 | -0.057 |
| Rayong | 0.732 | 0.758 | 0.759* | 0.758 | -0.014 | -0.012 | -0.01 |
| Roi Et | 0.795 | 0.821 | 0.821 | 0.822* | -0.022 | -0.02 | -0.022 |
| Sa Kaeo | 0.428 | 0.455* | 0.455 | 0.455 | 0.364 | 0.366 | 0.366 |
| Sakon Nakhon | 0.733 | 0.761 | 0.761 | 0.762* | 0.55 | 0.555 | 0.551 |
| Samut Prakan | 0.76 | 0.764 | 0.765* | 0.764 | -0.063 | -0.062 | -0.062 |
| Samut Sakhon | 0.52 | 0.542* | 0.542 | 0.542 | 0.331 | 0.331 | 0.337 |
| Samut Songkhram | 0.369 | 0.371* | 0.371 | 0.371 | 0.26 | 0.26 | 0.262 |
| Saraburi | 0.492 | 0.531 | 0.53 | 0.532* | 0.147 | 0.153 | 0.147 |
| Satun | 0.484 | 0.535* | 0.535 | 0.534 | 0.396 | 0.399 | 0.4 |
| Si Sa Ket | 0.706 | 0.758 | 0.759 | 0.76* | -0.055 | -0.056 | -0.055 |
| Sing Buri | 0.56 | 0.575* | 0.575 | 0.574 | 0.464 | 0.464 | 0.465 |
| Songkhla | 0.573 | 0.644* | 0.644 | 0.644 | -0.033 | -0.031 | -0.032 |
| Sukhothai | 0.482 | 0.527 | 0.528* | 0.528 | 0.081 | 0.081 | 0.079 |
| Suphan Buri | 0.5 | 0.521 | 0.522 | 0.524* | -0.028 | -0.026 | -0.027 |
| Surat Thani | 0.514 | 0.595 | 0.595 | 0.596* | -0.066 | -0.065 | -0.066 |
| Surin | 0.292 | 0.384 | 0.384 | 0.385* | -0.053 | -0.053 | -0.053 |
| Tak | 0.598 | 0.669* | 0.668 | 0.669 | -0.046 | -0.047 | -0.049 |
| Trang | 0.498 | 0.558 | 0.559* | 0.559 | 0.049 | 0.045 | 0.048 |
| Trat | 0.539* | 0.535 | 0.535 | 0.535 | 0.319 | 0.318 | 0.319 |
| Ubon Ratchathani | 0.476 | 0.549 | 0.548 | 0.55* | -0.056 | -0.056 | -0.055 |
| Udon Thani | 0.475 | 0.549* | 0.546 | 0.547 | -0.022 | -0.017 | -0.02 |
| Uthai Thani | 0.377 | 0.447 | 0.448 | 0.45* | -0.006 | -0.008 | -0.01 |
| Uttaradit | 0.523 | 0.58* | 0.578 | 0.58 | 0.091 | 0.092 | 0.09 |
| Yala | 0.647 | 0.68 | 0.681* | 0.68 | 0.368 | 0.377 | 0.37 |
| Yasothon | 0.692 | 0.725* | 0.724 | 0.725 | 0.427 | 0.432 | 0.43 |

Table 8: Adjusted $R^2$ for pneumonia case counts per 100,000

| | AR | LASSO-MIDAS | | | AR-MIDAS | | |
|---|---|---|---|---|---|---|---|
| | | Free | Down | Hump | Free | Down | Hump |
| Amnat Charoen | 0.57 | 0.593∗ | 0.588 | 0.593 | 0.559 | 0.56 | 0.56 |
| Ang Thong | 0.56 | 0.58 | 0.579 | 0.581∗ | 0.49 | 0.491 | 0.49 |
| Bangkok | 0.649 | 0.679∗ | 0.679 | 0.679 | 0.002 | 0.002 | 0.002 |
| Buri Ram | 0.577 | 0.594 | 0.595∗ | 0.595 | 0.001 | 0.002 | 0.001 |
| Chachoengsao | 0.694 | 0.7∗ | 0.7 | 0.7 | 0.429 | 0.439 | 0.439 |
| Chai Nat | 0.37 | 0.391 | 0.392 | 0.393∗ | 0.354 | 0.354 | 0.354 |
| Chaiyaphum | 0.621 | 0.634 | 0.633 | 0.636∗ | 0.217 | 0.222 | 0.215 |
| Chanthaburi | 0.622 | 0.655 | 0.656∗ | 0.656 | 0.019 | 0.02 | 0.018 |
| Chiang Mai | 0.682 | 0.727 | 0.728∗ | 0.727 | 0.004 | 0.003 | 0.004 |
| Chiang Rai | 0.521 | 0.556∗ | 0.556 | 0.556 | 0.001 | 0.001 | 0.001 |
| Chon Buri | 0.633 | 0.648 | 0.649∗ | 0.648 | 0.022 | 0.022 | 0.022 |
| Chumphon | 0.552 | 0.572∗ | 0.572 | 0.572 | 0.434 | 0.433 | 0.442 |
| Kalasin | 0.535 | 0.574∗ | 0.574 | 0.574 | 0.5 | 0.502 | 0.501 |
| Kamphaeng Phet | 0.47 | 0.488 | 0.489∗ | 0.488 | 0.07 | 0.071 | 0.07 |
| Kanchanaburi | 0.498 | 0.542∗ | 0.542 | 0.542 | 0.052 | 0.054 | 0.053 |
| Khon Kaen | 0.573 | 0.593 | 0.593 | 0.594∗ | 0.022 | 0.025 | 0.022 |
| Krabi | 0.369 | 0.401 | 0.401 | 0.402∗ | 0.336 | 0.336 | 0.336 |
| Lampang | 0.501 | 0.542∗ | 0.541 | 0.541 | 0.006 | 0.007 | 0.005 |
| Lamphun | 0.609 | 0.633∗ | 0.633 | 0.633 | 0.371 | 0.371 | 0.373 |
| Loei | 0.474 | 0.505 | 0.504 | 0.507∗ | 0.362 | 0.363 | 0.364 |
| Lop Buri | 0.478 | 0.509∗ | 0.507 | 0.508 | 0.224 | 0.229 | 0.226 |
| Mae Hong Son | 0.469 | 0.506∗ | 0.506 | 0.505 | 0.437 | 0.436 | 0.439 |
| Maha Sarakham | 0.617 | 0.632 | 0.632 | 0.633∗ | 0.466 | 0.47 | 0.47 |
| Mukdahan | 0.492 | 0.535∗ | 0.535 | 0.535 | 0.453 | 0.452 | 0.452 |
| Nakhon Nayok | 0.287 | 0.315∗ | 0.314 | 0.315 | 0.306 | 0.306 | 0.306 |
| Nakhon Pathom | 0.659 | 0.675∗ | 0.675 | 0.675 | 0.007 | 0.01 | 0.008 |
| Nakhon Phanom | 0.608 | 0.674∗ | 0.674 | 0.674 | 0.445 | 0.447 | 0.443 |
| Nakhon Ratchasima | 0.677 | 0.691 | 0.691 | 0.692∗ | 0.001 | 0.002 | 0.001 |
| Nakhon Sawan | 0.679 | 0.691∗ | 0.691 | 0.691 | 0.02 | 0.02 | 0.018 |
| Nakhon Si Thammarat | 0.713 | 0.731∗ | 0.731 | 0.731 | 0.126 | 0.123 | 0.127 |
| Nan | 0.682 | 0.697∗ | 0.697 | 0.697 | 0.508 | 0.506 | 0.511 |
| Narathiwat | 0.549 | 0.584∗ | 0.584 | 0.584 | 0.547 | 0.549 | 0.548 |
| Nong Bua Lam Phu | 0.442 | 0.452∗ | 0.45 | 0.452 | 0.41 | 0.412 | 0.41 |
| Nong Khai | 0.39 | 0.421∗ | 0.421 | 0.421 | 0.116 | 0.123 | 0.112 |
| Nonthaburi | 0.534 | 0.546∗ | 0.546 | 0.546 | 0.26 | 0.263 | 0.259 |
| Pathum Thani | 0.653 | 0.661∗ | 0.661 | 0.661 | 0.359 | 0.36 | 0.356 |
| Pattani | 0.307 | 0.332∗ | 0.331 | 0.331 | 0.325 | 0.324 | 0.324 |
| Phangnga | 0.718 | 0.73∗ | 0.73 | 0.73 | 0.672 | 0.671 | 0.674 |
| Phatthalung | 0.565 | 0.577 | 0.578∗ | 0.577 | 0.52 | 0.521 | 0.521 |
| Phayao | 0.629 | 0.655 | 0.656∗ | 0.655 | 0.166 | 0.167 | 0.165 |
| Phetchabun | 0.62 | 0.63∗ | 0.63 | 0.63 | 0.398 | 0.401 | 0.399 |
| Phetchaburi | 0.489 | 0.499 | 0.5∗ | 0.499 | 0.297 | 0.293 | 0.306 |
| Phichit | 0.436 | 0.468 | 0.469∗ | 0.468 | 0.281 | 0.282 | 0.282 |

| | | | | | | | |
|---|---|---|---|---|---|---|---|
| Phitsanulok | 0.392 | 0.465 | 0.466∗ | 0.465 | 0.001 | 0.001 | 0.002 |
| Phra Nakhon Si Ayutthaya | 0.638 | 0.657∗ | 0.655 | 0.656 | 0.203 | 0.209 | 0.202 |
| Phrae | 0.425 | 0.435∗ | 0.435 | 0.435 | 0.387 | 0.387 | 0.387 |
| Phuket | 0.466 | 0.498∗ | 0.498 | 0.498 | 0.31 | 0.307 | 0.31 |
| Prachin Buri | 0.599 | 0.607∗ | 0.607 | 0.607 | 0.496 | 0.496 | 0.496 |
| Prachuap Khiri Khan | 0.709 | 0.719∗ | 0.719 | 0.719 | 0.574 | 0.574 | 0.58 |
| Ranong | 0.354 | 0.392 | 0.393∗ | 0.392 | 0.385 | 0.385 | 0.385 |
| Ratchaburi | 0.473 | 0.487 | 0.488∗ | 0.486 | 0.069 | 0.069 | 0.072 |
| Rayong | 0.676 | 0.698∗ | 0.698 | 0.698 | 0 | 0 | 0 |
| Roi Et | 0.722 | 0.739 | 0.739 | 0.74∗ | 0.591 | 0.592 | 0.59 |
| Sa Kaeo | 0.659 | 0.665∗ | 0.665 | 0.665 | 0.639 | 0.639 | 0.641 |
| Sakon Nakhon | 0.547 | 0.598 | 0.599∗ | 0.598 | 0.523 | 0.523 | 0.524 |
| Samut Prakan | 0.644 | 0.663∗ | 0.663 | 0.663 | 0.012 | 0.012 | 0.012 |
| Samut Sakhon | 0.451 | 0.462∗ | 0.462 | 0.462 | 0.172 | 0.177 | 0.181 |
| Samut Songkhram | 0.547 | 0.553∗ | 0.553 | 0.553 | 0.534 | 0.534 | 0.534 |
| Saraburi | 0.537 | 0.543 | 0.543 | 0.544∗ | 0.478 | 0.479 | 0.478 |
| Satun | 0.508 | 0.517∗ | 0.517 | 0.517 | 0.504 | 0.504 | 0.505 |
| Si Sa Ket | 0.668 | 0.686 | 0.687∗ | 0.687 | 0.393 | 0.39 | 0.393 |
| Sing Buri | 0.386 | 0.401∗ | 0.401 | 0.401 | 0.369 | 0.369 | 0.369 |
| Songkhla | 0.529 | 0.555∗ | 0.555 | 0.555 | 0.354 | 0.365 | 0.354 |
| Sukhothai | 0.5 | 0.517 | 0.519∗ | 0.517 | 0.199 | 0.196 | 0.2 |
| Suphan Buri | 0.521 | 0.531 | 0.53 | 0.532∗ | 0.214 | 0.218 | 0.218 |
| Surat Thani | 0.633 | 0.652∗ | 0.651 | 0.652 | 0.018 | 0.017 | 0.017 |
| Surin | 0.678 | 0.695 | 0.696∗ | 0.696 | 0.15 | 0.146 | 0.148 |
| Tak | 0.599 | 0.631 | 0.632∗ | 0.631 | 0.436 | 0.437 | 0.442 |
| Trang | 0.383 | 0.427∗ | 0.427 | 0.427 | 0.189 | 0.19 | 0.196 |
| Trat | 0.611 | 0.637∗ | 0.637 | 0.636 | 0.519 | 0.52 | 0.52 |
| Ubon Ratchathani | 0.653 | 0.682 | 0.683∗ | 0.682 | 0.008 | 0.009 | 0.009 |
| Udon Thani | 0.59 | 0.598∗ | 0.598 | 0.598 | 0.309 | 0.312 | 0.306 |
| Uthai Thani | 0.396 | 0.433∗ | 0.433 | 0.433 | 0.37 | 0.37 | 0.37 |
| Uttaradit | 0.541 | 0.584 | 0.585∗ | 0.584 | 0.119 | 0.124 | 0.124 |
| Yala | 0.529 | 0.545∗ | 0.543 | 0.544 | 0.541 | 0.539 | 0.541 |
| Yasothon | 0.603 | 0.63∗ | 0.629 | 0.629 | 0.506 | 0.507 | 0.507 |

# 5 Coefficient output for Bayesian MIDAS-LASSO per province

## 5.1 Coefficient output for pneumonia case counts per 100,000 as the dependent variable

Table 9: Associations with monthly pneumonia case counts per 100,000 in Amnat Charoen

| | Posterior Mean | 95% CrI |
|---|---|---|
| Case Count Lag 1 | 0.315 | (0.184 ,0.444) |
| Case Count Lag 2 | -0.77 | (-0.902 ,-0.637) |
| Absolute Humidity Lag 1 | -8.957 | (-13.461 ,-4.655) |
| Absolute Humidity Lag 2 | -7.608 | (-11.118 ,-1.509) |
| Total Precipitation Lag 1 | 9524.473 | (-9048.677 ,29114.64) |
| Total Precipitation Lag 2 | -11335.536 | (-31215.829 ,7803.561) |
| Relative Humidity Lag 1 | -17.318 | (-83.905 ,47.019) |
| Relative Humidity Lag 2 | -10.288 | (-68.177 ,49.538) |
| Temperature Lag 1 | -136.454 | (-142.433 ,-130.147) |
| Temperature Lag 2 | -136.234 | (-144.663 ,-131.201) |

Table 10: Associations with monthly pneumonia case counts per 100,000 in Ang Thong

| | Posterior Mean | 95% CrI |
|---|---|---|
| Case Count Lag 1 | 0.255 | (0.124 ,0.384) |
| Case Count Lag 2 | -0.813 | (-0.941 ,-0.68) |
| Absolute Humidity Lag 1 | -13.447 | (-23.629 ,-4.224) |
| Absolute Humidity Lag 2 | -10.085 | (-18.884 ,1.379) |
| Total Precipitation Lag 1 | -29468.167 | (-83047.806 ,20229.133) |
| Total Precipitation Lag 2 | -8431.611 | (-57783.672 ,39474.522) |
| Relative Humidity Lag 1 | 53.603 | (-44.744 ,164.456) |
| Relative Humidity Lag 2 | -82.4 | (-175.528 ,13.991) |
| Temperature Lag 1 | -189.614 | (-205.051 ,-172.835) |
| Temperature Lag 2 | -187.61 | (-206.554 ,-173.135) |

Table 11: Associations with monthly pneumonia case counts per 100,000 in Bangkok

| | Posterior Mean | 95% CrI |
|---|---|---|
| Case Count Lag 1 | 0.349 | (0.219 ,0.475) |
| Case Count Lag 2 | -0.896 | (-1.02 ,-0.767) |
| Absolute Humidity Lag 1 | -85.316 | (-827.07 ,768.977) |
| Absolute Humidity Lag 2 | 204.312 | (-505.991 ,1012.487) |
| Total Precipitation Lag 1 | -3176973.65 | (-6471381.116 ,102512.363) |
| Total Precipitation Lag 2 | -1390526.432 | (-4390237.667 ,1336504.353) |
| Relative Humidity Lag 1 | 8961.353 | (142.179 ,18587.28) |
| Relative Humidity Lag 2 | -1047.727 | (-8736.867 ,6494.69) |
| Temperature Lag 1 | -464.866 | (-1817.987 ,665.2) |
| Temperature Lag 2 | -80.737 | (-1296.361 ,1078.616) |

Table 12: Associations with monthly pneumonia case counts per 100,000 in Buri Ram

| | Posterior Mean | 95% CrI |
|---|---|---|
| Case Count Lag 1 | 0.333 | (0.203 ,0.458) |
| Case Count Lag 2 | -0.902 | (-1.03 ,-0.77) |
| Absolute Humidity Lag 1 | -19.606 | (-90.864 ,34.867) |
| Absolute Humidity Lag 2 | 4.374 | (-46.297 ,83.018) |
| Total Precipitation Lag 1 | 245365.245 | (-106815.11 ,631056.751) |
| Total Precipitation Lag 2 | -54813.79 | (-422649.894 ,286429.862) |
| Relative Humidity Lag 1 | -446.025 | (-1402.285 ,410.388) |
| Relative Humidity Lag 2 | 105.193 | (-675.297 ,975.16) |
| Temperature Lag 1 | -150.431 | (-228.358 ,-46.797) |
| Temperature Lag 2 | -155.878 | (-272.71 ,-82.558) |

Table 13: Associations with monthly pneumonia case counts per 100,000 in Chachoengsao

| | Posterior Mean | 95% CrI |
|---|---|---|
| Case Count Lag 1 | 0.505 | (0.376 ,0.628) |
| Case Count Lag 2 | -0.779 | (-0.905 ,-0.641) |
| Absolute Humidity Lag 1 | -25.023 | (-84.223 ,11.762) |
| Absolute Humidity Lag 2 | -9.54 | (-51.437 ,38.465) |
| Total Precipitation Lag 1 | 62349.58 | (-72653.865 ,208157.014) |
| Total Precipitation Lag 2 | -50927.003 | (-195503.361 ,86390.962) |
| Relative Humidity Lag 1 | -40.206 | (-465.775 ,374.537) |
| Relative Humidity Lag 2 | -67.482 | (-444.43 ,288.905) |
| Temperature Lag 1 | -241.802 | (-298.151 ,-152.827) |
| Temperature Lag 2 | -245.012 | (-314.847 ,-181.069) |

Table 14: Associations with monthly pneumonia case counts per 100,000 in Chai Nat

| | Posterior Mean | 95% CrI |
|---|---|---|
| Case Count Lag 1 | 0.033 | (-0.094 ,0.168) |
| Case Count Lag 2 | -0.716 | (-0.85 ,-0.592) |
| Absolute Humidity Lag 1 | -10.19 | (-15.802 ,-6.097) |
| Absolute Humidity Lag 2 | -9.489 | (-14.011 ,-4.819) |
| Total Precipitation Lag 1 | -336.838 | (-31261.862 ,29907.824) |
| Total Precipitation Lag 2 | -1654.825 | (-31604.368 ,28940.232) |
| Relative Humidity Lag 1 | 3.641 | (-64.147 ,73.855) |
| Relative Humidity Lag 2 | -21.539 | (-87.698 ,42.131) |
| Temperature Lag 1 | -154.929 | (-161.364 ,-146.025) |
| Temperature Lag 2 | -154.597 | (-162.341 ,-147.436) |

Table 15: Associations with monthly pneumonia case counts per 100,000 in Chaiyaphum

| | Posterior Mean | 95% CrI |
|---|---|---|
| Case Count Lag 1 | 0.429 | (0.297 ,0.554) |
| Case Count Lag 2 | -0.726 | (-0.858 ,-0.595) |
| Absolute Humidity Lag 1 | -14.645 | (-55.469 ,23.765) |
| Absolute Humidity Lag 2 | -5.655 | (-42.012 ,33.341) |
| Total Precipitation Lag 1 | 36098.241 | (-140789.205 ,223625.734) |
| Total Precipitation Lag 2 | -39732.9 | (-223506.227 ,148590.862) |
| Relative Humidity Lag 1 | -16.357 | (-542.384 ,539.286) |
| Relative Humidity Lag 2 | -94.625 | (-628.988 ,433.368) |
| Temperature Lag 1 | -156.24 | (-207.735 ,-100.872) |
| Temperature Lag 2 | -147.901 | (-199.096 ,-99.092) |

Table 16: Associations with monthly pneumonia case counts per 100,000 in Chanthaburi

| | Posterior Mean | 95% CrI |
|---|---|---|
| Case Count Lag 1 | 0.183 | (0.058 ,0.304) |
| Case Count Lag 2 | -0.987 | (-1.104 ,-0.863) |
| Absolute Humidity Lag 1 | -22.088 | (-110.448 ,67.601) |
| Absolute Humidity Lag 2 | 52.743 | (-29.116 ,222.44) |
| Total Precipitation Lag 1 | 28794.9 | (-97526.033 ,165822.777) |
| Total Precipitation Lag 2 | 64308.202 | (-68066.388 ,210688.084) |
| Relative Humidity Lag 1 | -139.654 | (-713.991 ,397.576) |
| Relative Humidity Lag 2 | -453.634 | (-1033.73 ,89.273) |
| Temperature Lag 1 | -325.731 | (-457.394 ,-201.631) |
| Temperature Lag 2 | -370.786 | (-622.223 ,-250.818) |

Table 17: Associations with monthly pneumonia case counts per 100,000 in Chiang Mai

| | Posterior Mean | 95% CrI |
|---|---|---|
| Case Count Lag 1 | 0.466 | (0.341 ,0.589) |
| Case Count Lag 2 | -0.917 | (-1.039 ,-0.795) |
| Absolute Humidity Lag 1 | 120.553 | (-53.545 ,487.327) |
| Absolute Humidity Lag 2 | -10.577 | (-223.069 ,124.838) |
| Total Precipitation Lag 1 | 31559.7 | (-596880.9 ,701644.805) |
| Total Precipitation Lag 2 | -911550.211 | (-1747915.526 ,-77767.965) |
| Relative Humidity Lag 1 | 1477.494 | (-385.37 ,3612.382) |
| Relative Humidity Lag 2 | 1053.517 | (-733.847 ,2956.404) |
| Temperature Lag 1 | -314.265 | (-738.8 ,-103.642) |
| Temperature Lag 2 | -46.918 | (-211.28 ,208.08) |

Table 18: Associations with monthly pneumonia case counts per 100,000 in Chiang Rai

| | Posterior Mean | 95% CrI |
|---|---|---|
| Case Count Lag 1 | 0.115 | (-0.017 ,0.247) |
| Case Count Lag 2 | -0.653 | (-0.787 ,-0.525) |
| Absolute Humidity Lag 1 | 24.205 | (-27.759 ,138.395) |
| Absolute Humidity Lag 2 | -2.08 | (-57.526 ,42.61) |
| Total Precipitation Lag 1 | 62979.677 | (-165088.237 ,291668.872) |
| Total Precipitation Lag 2 | -153374.843 | (-444124.331 ,111351.966) |
| Relative Humidity Lag 1 | 399.133 | (-348.628 ,1283.845) |
| Relative Humidity Lag 2 | 84.762 | (-595.672 ,793.999) |
| Temperature Lag 1 | -164.793 | (-301.537 ,-100.2) |
| Temperature Lag 2 | -91.228 | (-146.374 ,-23.145) |

Table 19: Associations with monthly pneumonia case counts per 100,000 in Chon Buri

| | Posterior Mean | 95% CrI |
|---|---|---|
| Case Count Lag 1 | 0.261 | (0.134 ,0.392) |
| Case Count Lag 2 | -0.904 | (-1.035 ,-0.77) |
| Absolute Humidity Lag 1 | -37.425 | (-150.66 ,46.327) |
| Absolute Humidity Lag 2 | 13.756 | (-59.839 ,128.561) |
| Total Precipitation Lag 1 | -166286.293 | (-403943.139 ,63783.622) |
| Total Precipitation Lag 2 | -4856.765 | (-224770.392 ,202180.562) |
| Relative Humidity Lag 1 | 313.244 | (-373.769 ,1041.387) |
| Relative Humidity Lag 2 | -287.665 | (-919.559 ,310.86) |
| Temperature Lag 1 | -278.002 | (-403.504 ,-112.139) |
| Temperature Lag 2 | -286.916 | (-455.009 ,-172.998) |

Table 20: Associations with monthly pneumonia case counts per 100,000 in Chumphon

| | Posterior Mean | 95% CrI |
|---|---|---|
| Case Count Lag 1 | 0.319 | (0.195 ,0.446) |
| Case Count Lag 2 | -0.743 | (-0.869 ,-0.618) |
| Absolute Humidity Lag 1 | -19.814 | (-58.031 ,35.73) |
| Absolute Humidity Lag 2 | 2.19 | (-32.8 ,95.524) |
| Total Precipitation Lag 1 | -47011.92 | (-115909.804 ,15070.623) |
| Total Precipitation Lag 2 | 55907.388 | (-8657.533 ,124219.091) |
| Relative Humidity Lag 1 | 145.341 | (-216.768 ,541.955) |
| Relative Humidity Lag 2 | -252.92 | (-608.737 ,93.02) |
| Temperature Lag 1 | -377.17 | (-459.723 ,-321.48) |
| Temperature Lag 2 | -382.701 | (-522.472 ,-328.848) |

Table 21: Associations with monthly pneumonia case counts per 100,000 in Kalasin

| | Posterior Mean | 95% CrI |
|---|---|---|
| Case Count Lag 1 | 0.311 | (0.183 ,0.448) |
| Case Count Lag 2 | -0.548 | (-0.678 ,-0.423) |
| Absolute Humidity Lag 1 | -10.102 | (-23.619 ,-0.986) |
| Absolute Humidity Lag 2 | -7.445 | (-16.703 ,3.072) |
| Total Precipitation Lag 1 | 79754.532 | (14128.566 ,144964.114) |
| Total Precipitation Lag 2 | -64424.091 | (-134227.779 ,4135.237) |
| Relative Humidity Lag 1 | -15.347 | (-180.701 ,153.759) |
| Relative Humidity Lag 2 | -26.019 | (-175.479 ,120.896) |
| Temperature Lag 1 | -125.607 | (-138.491 ,-107.3) |
| Temperature Lag 2 | -125.368 | (-139.866 ,-112.403) |

Table 22: Associations with monthly pneumonia case counts per 100,000 in Kamphaeng Phet

| | Posterior Mean | 95% CrI |
|---|---|---|
| Case Count Lag 1 | 0.129 | (-0.002 ,0.259) |
| Case Count Lag 2 | -0.778 | (-0.908 ,-0.644) |
| Absolute Humidity Lag 1 | -8.31 | (-36.271 ,23.998) |
| Absolute Humidity Lag 2 | -3.075 | (-26.997 ,37.249) |
| Total Precipitation Lag 1 | 28605.234 | (-119252.087 ,184874.1) |
| Total Precipitation Lag 2 | -110561.043 | (-290594.337 ,52505.207) |
| Relative Humidity Lag 1 | 162.409 | (-298.733 ,658.805) |
| Relative Humidity Lag 2 | 79.913 | (-377.804 ,599.019) |
| Temperature Lag 1 | -142.471 | (-187.732 ,-105.093) |
| Temperature Lag 2 | -145.042 | (-201.03 ,-111.226) |

Table 23: Associations with monthly pneumonia case counts per 100,000 in Kanchanaburi

| | Posterior Mean | 95% CrI |
|---|---|---|
| Case Count Lag 1 | 0.044 | (-0.086 ,0.175) |
| Case Count Lag 2 | -0.713 | (-0.84 ,-0.592) |
| Absolute Humidity Lag 1 | -18.211 | (-73.359 ,23.315) |
| Absolute Humidity Lag 2 | -3.839 | (-42.574 ,53.514) |
| Total Precipitation Lag 1 | 252771.856 | (92758.16 ,419971.541) |
| Total Precipitation Lag 2 | -221420.714 | (-396362.547 ,-48664.783) |
| Relative Humidity Lag 1 | 13.08 | (-501.72 ,544.977) |
| Relative Humidity Lag 2 | 139.355 | (-362.873 ,685.533) |
| Temperature Lag 1 | -199.939 | (-258.097 ,-124.758) |
| Temperature Lag 2 | -203.549 | (-283.672 ,-152.637) |

Table 24: Associations with monthly pneumonia case counts per 100,000 in Khon Kaen

| | Posterior Mean | 95% CrI |
|---|---|---|
| Case Count Lag 1 | 0.33 | (0.196 ,0.46) |
| Case Count Lag 2 | -0.741 | (-0.877 ,-0.601) |
| Absolute Humidity Lag 1 | -12.463 | (-60.044 ,39.506) |
| Absolute Humidity Lag 2 | -2.08 | (-44.789 ,49.065) |
| Total Precipitation Lag 1 | 2422.962 | (-312860.105 ,315984.119) |
| Total Precipitation Lag 2 | -53345.395 | (-391113.97 ,280746.508) |
| Relative Humidity Lag 1 | 14.931 | (-746.032 ,797.352) |
| Relative Humidity Lag 2 | -188.907 | (-942.878 ,518.354) |
| Temperature Lag 1 | -139.043 | (-210.798 ,-74.296) |
| Temperature Lag 2 | -124.441 | (-195.339 ,-63.899) |

Table 25: Associations with monthly pneumonia case counts per 100,000 in Krabi

| | Posterior Mean | 95% CrI |
|---|---|---|
| Case Count Lag 1 | -0.308 | (-0.436 ,-0.18) |
| Case Count Lag 2 | -0.732 | (-0.854 ,-0.6) |
| Absolute Humidity Lag 1 | -22.94 | (-54.476 ,4.773) |
| Absolute Humidity Lag 2 | -15.884 | (-44.188 ,15.243) |
| Total Precipitation Lag 1 | -24050.184 | (-77636.361 ,26078.063) |
| Total Precipitation Lag 2 | -50921.481 | (-104311.35 ,-260.979) |
| Relative Humidity Lag 1 | -3.747 | (-257.88 ,247.708) |
| Relative Humidity Lag 2 | 258.659 | (17.972 ,499.787) |
| Temperature Lag 1 | -379.412 | (-424.173 ,-330.512) |
| Temperature Lag 2 | -373.787 | (-420.56 ,-329.233) |

Table 26: Associations with monthly pneumonia case counts per 100,000 in Lampang

| | Posterior Mean | 95% CrI |
|---|---|---|
| Case Count Lag 1 | 0.051 | (-0.081 ,0.181) |
| Case Count Lag 2 | -0.879 | (-1.008 ,-0.751) |
| Absolute Humidity Lag 1 | 28.544 | (-26.803 ,144.395) |
| Absolute Humidity Lag 2 | -5.145 | (-56.614 ,43.654) |
| Total Precipitation Lag 1 | 94670.879 | (-176438.989 ,392977.746) |
| Total Precipitation Lag 2 | -308257.426 | (-641018.302 ,8893.317) |
| Relative Humidity Lag 1 | 361.615 | (-377.793 ,1256.546) |
| Relative Humidity Lag 2 | 358.766 | (-297.89 ,1115.316) |
| Temperature Lag 1 | -185.839 | (-335.902 ,-112.659) |
| Temperature Lag 2 | -117.44 | (-182.165 ,-49.81) |

Table 27: Associations with monthly pneumonia case counts per 100,000 in Lamphun

| | Posterior Mean | 95% CrI |
|---|---|---|
| Case Count Lag 1 | 0.235 | (0.109 ,0.367) |
| Case Count Lag 2 | -0.638 | (-0.763 ,-0.515) |
| Absolute Humidity Lag 1 | -4.971 | (-20.841 ,22.682) |
| Absolute Humidity Lag 2 | -4.593 | (-26.593 ,14.582) |
| Total Precipitation Lag 1 | -9665.126 | (-132346.044 ,105673.786) |
| Total Precipitation Lag 2 | -186749.133 | (-335128.591 ,-38656.271) |
| Relative Humidity Lag 1 | 68.897 | (-204.476 ,384.872) |
| Relative Humidity Lag 2 | 331.818 | (47.932 ,627.373) |
| Temperature Lag 1 | -129.313 | (-166.06 ,-108.906) |
| Temperature Lag 2 | -113.971 | (-138.919 ,-84.048) |

Table 28: Associations with monthly pneumonia case counts per 100,000 in Loei

| | Posterior Mean | 95% CrI |
|---|---|---|
| Case Count Lag 1 | 0.003 | (-0.131 ,0.136) |
| Case Count Lag 2 | -0.906 | (-1.039 ,-0.775) |
| Absolute Humidity Lag 1 | -7.845 | (-20.181 ,7.286) |
| Absolute Humidity Lag 2 | -3.816 | (-14.653 ,16.346) |
| Total Precipitation Lag 1 | 56455.16 | (-21487.238 ,134782) |
| Total Precipitation Lag 2 | -31334.697 | (-115845.877 ,47729.43) |
| Relative Humidity Lag 1 | 47.735 | (-166.012 ,275.225) |
| Relative Humidity Lag 2 | -63.003 | (-275.121 ,141.948) |
| Temperature Lag 1 | -129.461 | (-149.156 ,-114.15) |
| Temperature Lag 2 | -130.347 | (-156.275 ,-116.646) |

Table 29: Associations with monthly pneumonia case counts per 100,000 in Lop Buri

| | Posterior Mean | 95% CrI |
|---|---|---|
| Case Count Lag 1 | 0.06 | (-0.069 ,0.19) |
| Case Count Lag 2 | -0.772 | (-0.903 ,-0.642) |
| Absolute Humidity Lag 1 | -15.765 | (-49.983 ,16.067) |
| Absolute Humidity Lag 2 | -10.734 | (-42.98 ,19.185) |
| Total Precipitation Lag 1 | -53205.141 | (-226991.117 ,112302.334) |
| Total Precipitation Lag 2 | -71625.287 | (-236922.512 ,92043.906) |
| Relative Humidity Lag 1 | 366.357 | (-7.039 ,768.046) |
| Relative Humidity Lag 2 | -224.993 | (-625.487 ,137.979) |
| Temperature Lag 1 | -181.923 | (-232.698 ,-130.07) |
| Temperature Lag 2 | -175.456 | (-222.126 ,-124.941) |

Table 30: Associations with monthly pneumonia case counts per 100,000 in Mae Hong Son

| | Posterior Mean | 95% CrI |
|---|---|---|
| Case Count Lag 1 | 0.134 | (0.003 ,0.268) |
| Case Count Lag 2 | -0.706 | (-0.839 ,-0.576) |
| Absolute Humidity Lag 1 | -7.774 | (-15.564 ,3.722) |
| Absolute Humidity Lag 2 | -6.036 | (-13.151 ,4.747) |
| Total Precipitation Lag 1 | 69536.629 | (29103.486 ,111538.1) |
| Total Precipitation Lag 2 | -26492.976 | (-78071.587 ,21632.979) |
| Relative Humidity Lag 1 | -81.559 | (-197.565 ,23.026) |
| Relative Humidity Lag 2 | 30.03 | (-61.336 ,138.365) |
| Temperature Lag 1 | -131.563 | (-144.797 ,-122.582) |
| Temperature Lag 2 | -127.574 | (-140.149 ,-118.624) |

Table 31: Associations with monthly pneumonia case counts per 100,000 in Maha Sarakham

| | Posterior Mean | 95% CrI |
|---|---|---|
| Case Count Lag 1 | 0.35 | (0.218 ,0.476) |
| Case Count Lag 2 | -0.822 | (-0.953 ,-0.689) |
| Absolute Humidity Lag 1 | -10.772 | (-26.688 ,2.371) |
| Absolute Humidity Lag 2 | -6.084 | (-18.22 ,10.159) |
| Total Precipitation Lag 1 | 67340.377 | (-16913.457 ,155767.471) |
| Total Precipitation Lag 2 | 30297.894 | (-54426.69 ,121235.844) |
| Relative Humidity Lag 1 | -77.905 | (-316.571 ,130.694) |
| Relative Humidity Lag 2 | -90.222 | (-299.51 ,102.997) |
| Temperature Lag 1 | -132.458 | (-151.766 ,-109.83) |
| Temperature Lag 2 | -131.831 | (-155.823 ,-114.085) |

Table 32: Associations with monthly pneumonia case counts per 100,000 in Mukdahan

| | Posterior Mean | 95% CrI |
|---|---|---|
| Case Count Lag 1 | 0.266 | (0.139 ,0.405) |
| Case Count Lag 2 | -0.531 | (-0.668 ,-0.41) |
| Absolute Humidity Lag 1 | -11.673 | (-30.146 ,-0.467) |
| Absolute Humidity Lag 2 | -5.726 | (-17.602 ,10.607) |
| Total Precipitation Lag 1 | 117597.977 | (47542.919 ,190031.589) |
| Total Precipitation Lag 2 | -51345.775 | (-126590.705 ,22581.003) |
| Relative Humidity Lag 1 | -59.878 | (-286.154 ,136.6) |
| Relative Humidity Lag 2 | -40.74 | (-237.385 ,145.59) |
| Temperature Lag 1 | -121.721 | (-136.731 ,-97.192) |
| Temperature Lag 2 | -122.341 | (-144.485 ,-106.456) |

Table 33: Associations with monthly pneumonia case counts per 100,000 in Nakhon Nayok

| | Posterior Mean | 95% CrI |
|---|---|---|
| Case Count Lag 1 | 0.139 | (0.009 ,0.264) |
| Case Count Lag 2 | -0.58 | (-0.708 ,-0.45) |
| Absolute Humidity Lag 1 | -13.843 | (-21.659 ,-5.48) |
| Absolute Humidity Lag 2 | -13.425 | (-21.553 ,-5.565) |
| Total Precipitation Lag 1 | -20560.754 | (-44929.567 ,4237.973) |
| Total Precipitation Lag 2 | -2711.173 | (-25522.867 ,19992.908) |
| Relative Humidity Lag 1 | 106.704 | (10.857 ,202.833) |
| Relative Humidity Lag 2 | -43.724 | (-130.778 ,37.873) |
| Temperature Lag 1 | -220.956 | (-233.226 ,-209.837) |
| Temperature Lag 2 | -217.994 | (-229.196 ,-206.418) |

Table 34: Associations with monthly pneumonia case counts per 100,000 in Nakhon Pathom

| | Posterior Mean | 95% CrI |
|---|---|---|
| Case Count Lag 1 | 0.369 | (0.237 ,0.503) |
| Case Count Lag 2 | -0.752 | (-0.885 ,-0.623) |
| Absolute Humidity Lag 1 | -26.069 | (-101.686 ,34.729) |
| Absolute Humidity Lag 2 | 1.291 | (-53.475 ,83.001) |
| Total Precipitation Lag 1 | -36188.516 | (-366403.176 ,275774.954) |
| Total Precipitation Lag 2 | -123977.526 | (-425161.589 ,176402.418) |
| Relative Humidity Lag 1 | 119.186 | (-542.861 ,806.952) |
| Relative Humidity Lag 2 | -144.525 | (-796.949 ,466.912) |
| Temperature Lag 1 | -203.68 | (-303.285 ,-83.997) |
| Temperature Lag 2 | -200.09 | (-331.289 ,-110.306) |

Table 35: Associations with monthly pneumonia case counts per 100,000 in Nakhon Phanom

| | Posterior Mean | 95% CrI |
|---|---|---|
| Case Count Lag 1 | 0.152 | (0.018 ,0.276) |
| Case Count Lag 2 | -0.885 | (-1.01 ,-0.751) |
| Absolute Humidity Lag 1 | -9.847 | (-26.794 ,8.457) |
| Absolute Humidity Lag 2 | 10.672 | (-8.461 ,42.567) |
| Total Precipitation Lag 1 | 148040.312 | (77402.251 ,218898.176) |
| Total Precipitation Lag 2 | -13182.879 | (-90826.165 ,60536.485) |
| Relative Humidity Lag 1 | -145.413 | (-393.845 ,97.091) |
| Relative Humidity Lag 2 | -181.786 | (-422.989 ,48.952) |
| Temperature Lag 1 | -120.881 | (-145.466 ,-98.953) |
| Temperature Lag 2 | -134.085 | (-175.752 ,-109.224) |

Table 36: Associations with monthly pneumonia case counts per 100,000 in Nakhon Ratchasima

| | Posterior Mean | 95% CrI |
|---|---|---|
| Case Count Lag 1 | 0.478 | (0.351 ,0.608) |
| Case Count Lag 2 | -0.742 | (-0.872 ,-0.615) |
| Absolute Humidity Lag 1 | -29.275 | (-190.281 ,121.4) |
| Absolute Humidity Lag 2 | 5.398 | (-139.099 ,163.522) |
| Total Precipitation Lag 1 | -20652.594 | (-907955.488 ,816039.176) |
| Total Precipitation Lag 2 | -262573.337 | (-1167673.267 ,591412.965) |
| Relative Humidity Lag 1 | 275.163 | (-1767.015 ,2551.734) |
| Relative Humidity Lag 2 | -44.001 | (-2074.892 ,2038.46) |
| Temperature Lag 1 | -186.461 | (-404.095 ,32.875) |
| Temperature Lag 2 | -135.41 | (-354.292 ,73.388) |

Table 37: Associations with monthly pneumonia case counts per 100,000 in Nakhon Sawan

| | Posterior Mean | 95% CrI |
|---|---|---|
| Case Count Lag 1 | 0.425 | (0.294 ,0.557) |
| Case Count Lag 2 | -0.814 | (-0.941 ,-0.682) |
| Absolute Humidity Lag 1 | -11.751 | (-53.231 ,35.805) |
| Absolute Humidity Lag 2 | -0.705 | (-37.62 ,52.036) |
| Total Precipitation Lag 1 | -18741.522 | (-280863.986 ,228406.849) |
| Total Precipitation Lag 2 | -159927.837 | (-428348.869 ,104722.914) |
| Relative Humidity Lag 1 | 276.615 | (-355.539 ,965.967) |
| Relative Humidity Lag 2 | -76.867 | (-706.237 ,567.308) |
| Temperature Lag 1 | -157.213 | (-229.603 ,-94.065) |
| Temperature Lag 2 | -153.184 | (-235.823 ,-97.516) |

Table 38: Associations with monthly pneumonia case counts per 100,000 in Nakhon Si Thammarat

| | Posterior Mean | 95% CrI |
|---|---|---|
| Case Count Lag 1 | 0.028 | (-0.096 ,0.154) |
| Case Count Lag 2 | -1.108 | (-1.231 ,-0.981) |
| Absolute Humidity Lag 1 | -16.463 | (-80.731 ,92.2) |
| Absolute Humidity Lag 2 | 14.852 | (-53.916 ,142.073) |
| Total Precipitation Lag 1 | -40051.245 | (-156804.735 ,80770.111) |
| Total Precipitation Lag 2 | 58460.508 | (-56789.972 ,176931.313) |
| Relative Humidity Lag 1 | -486.956 | (-1348.243 ,285.927) |
| Relative Humidity Lag 2 | 385.91 | (-310.295 ,1164.212) |
| Temperature Lag 1 | -391.382 | (-565.14 ,-291.746) |
| Temperature Lag 2 | -361.558 | (-557.365 ,-252.89) |

Table 39: Associations with monthly pneumonia case counts per 100,000 in Nan

| | Posterior Mean | 95% CrI |
|---|---|---|
| Case Count Lag 1 | 0.271 | (0.142 ,0.397) |
| Case Count Lag 2 | -0.863 | (-0.988 ,-0.73) |
| Absolute Humidity Lag 1 | -11.099 | (-30.186 ,4.459) |
| Absolute Humidity Lag 2 | -5.281 | (-19.85 ,13.593) |
| Total Precipitation Lag 1 | 91718.264 | (26319.176 ,158625.263) |
| Total Precipitation Lag 2 | -43435.503 | (-125519.71 ,30026.44) |
| Relative Humidity Lag 1 | -42.081 | (-289.426 ,197.113) |
| Relative Humidity Lag 2 | 21.922 | (-189.374 ,260.202) |
| Temperature Lag 1 | -129.104 | (-148.43 ,-105.603) |
| Temperature Lag 2 | -125.766 | (-148.259 ,-107.15) |

Table 40: Associations with monthly pneumonia case counts per 100,000 in Narathiwat

| | Posterior Mean | 95% CrI |
|---|---|---|
| Case Count Lag 1 | -0.341 | (-0.463 ,-0.209) |
| Case Count Lag 2 | -0.954 | (-1.077 ,-0.829) |
| Absolute Humidity Lag 1 | -19.233 | (-52.827 ,6.003) |
| Absolute Humidity Lag 2 | -9.546 | (-33.109 ,32.718) |
| Total Precipitation Lag 1 | -11100.06 | (-51309.809 ,26962.922) |
| Total Precipitation Lag 2 | 52773.083 | (11205.321 ,93751.264) |
| Relative Humidity Lag 1 | 180.631 | (-142.387 ,511.176) |
| Relative Humidity Lag 2 | -7.233 | (-318.201 ,319.944) |
| Temperature Lag 1 | -313.453 | (-349.296 ,-264.147) |
| Temperature Lag 2 | -315.05 | (-377.192 ,-280.758) |

Table 41: Associations with monthly pneumonia case counts per 100,000 in Nong Bua Lam Phu

| | Posterior Mean | 95% CrI |
|---|---|---|
| Case Count Lag 1 | 0.35 | (0.221 ,0.479) |
| Case Count Lag 2 | -0.614 | (-0.742 ,-0.481) |
| Absolute Humidity Lag 1 | -7.699 | (-13.735 ,-0.139) |
| Absolute Humidity Lag 2 | -7.212 | (-14.001 ,-0.713) |
| Total Precipitation Lag 1 | -14075.562 | (-62047.232 ,29470.977) |
| Total Precipitation Lag 2 | 13081.873 | (-31973.466 ,63445.486) |
| Relative Humidity Lag 1 | 0.135 | (-118.517 ,122.16) |
| Relative Humidity Lag 2 | -59.721 | (-179.437 ,51.046) |
| Temperature Lag 1 | -119.19 | (-129.423 ,-111.27) |
| Temperature Lag 2 | -115.553 | (-124.357 ,-106.256) |

Table 42: Associations with monthly pneumonia case counts per 100,000 in Nong Khai

| | Posterior Mean | 95% CrI |
|---|---|---|
| Case Count Lag 1 | 0.265 | (0.136 ,0.399) |
| Case Count Lag 2 | -0.576 | (-0.704 ,-0.444) |
| Absolute Humidity Lag 1 | -9.56 | (-40.649 ,29.776) |
| Absolute Humidity Lag 2 | -7.438 | (-47.618 ,23.634) |
| Total Precipitation Lag 1 | 205533.759 | (31576.198 ,380782.202) |
| Total Precipitation Lag 2 | 48263.252 | (-131417.979 ,249713.358) |
| Relative Humidity Lag 1 | -275.899 | (-872.308 ,255.613) |
| Relative Humidity Lag 2 | -321.44 | (-882.466 ,189.59) |
| Temperature Lag 1 | -128.555 | (-181.75 ,-86.606) |
| Temperature Lag 2 | -110.401 | (-150.653 ,-57.035) |

Table 43: Associations with monthly pneumonia case counts per 100,000 in Nonthaburi

| | Posterior Mean | 95% CrI |
|---|---|---|
| Case Count Lag 1 | 0.159 | (0.03 ,0.287) |
| Case Count Lag 2 | -0.94 | (-1.07 ,-0.813) |
| Absolute Humidity Lag 1 | -15.949 | (-48.494 ,15.826) |
| Absolute Humidity Lag 2 | -5.48 | (-35.055 ,28.499) |
| Total Precipitation Lag 1 | -83187.027 | (-239682.039 ,70674.298) |
| Total Precipitation Lag 2 | -690.372 | (-139345.481 ,142338.197) |
| Relative Humidity Lag 1 | 271.975 | (-59.261 ,619.566) |
| Relative Humidity Lag 2 | -152.067 | (-463.828 ,149.798) |
| Temperature Lag 1 | -205.314 | (-258.446 ,-153.41) |
| Temperature Lag 2 | -196.933 | (-249.87 ,-149.337) |

Table 44: Associations with monthly pneumonia case counts per 100,000 in Pathum Thani

| | Posterior Mean | 95% CrI |
|---|---|---|
| Case Count Lag 1 | 0.463 | (0.338 ,0.585) |
| Case Count Lag 2 | -0.971 | (-1.097 ,-0.847) |
| Absolute Humidity Lag 1 | -15.07 | (-45.497 ,19.183) |
| Absolute Humidity Lag 2 | -4.143 | (-33.642 ,30.927) |
| Total Precipitation Lag 1 | -17333.018 | (-162542.904 ,112422.014) |
| Total Precipitation Lag 2 | -46988.484 | (-180974.676 ,79757.766) |
| Relative Humidity Lag 1 | 47.169 | (-302.996 ,438.617) |
| Relative Humidity Lag 2 | 6.391 | (-324.804 ,356.388) |
| Temperature Lag 1 | -213.336 | (-267.498 ,-165.256) |
| Temperature Lag 2 | -199.019 | (-252.055 ,-152.655) |

Table 45: Associations with monthly pneumonia case counts per 100,000 in Pattani

| | Posterior Mean | 95% CrI |
|---|---|---|
| Case Count Lag 1 | -0.313 | (-0.443 ,-0.185) |
| Case Count Lag 2 | -0.921 | (-1.049 ,-0.803) |
| Absolute Humidity Lag 1 | -17.667 | (-28.318 ,-2.862) |
| Absolute Humidity Lag 2 | -17.403 | (-30.104 ,-5.495) |
| Total Precipitation Lag 1 | 11144.137 | (-10372.414 ,34184.738) |
| Total Precipitation Lag 2 | -6927.578 | (-30874.018 ,14777.545) |
| Relative Humidity Lag 1 | -120.253 | (-239.865 ,-1.794) |
| Relative Humidity Lag 2 | 86.003 | (-23.762 ,211.301) |
| Temperature Lag 1 | -327.998 | (-350.967 ,-311.697) |
| Temperature Lag 2 | -324.252 | (-342.014 ,-303.915) |

Table 46: Associations with monthly pneumonia case counts per 100,000 in Phangnga

| | Posterior Mean | 95% CrI |
|---|---|---|
| Case Count Lag 1 | 0.099 | (-0.033 ,0.229) |
| Case Count Lag 2 | -0.993 | (-1.12 ,-0.865) |
| Absolute Humidity Lag 1 | -26.052 | (-53.887 ,-1.358) |
| Absolute Humidity Lag 2 | -21.536 | (-46.245 ,8.101) |
| Total Precipitation Lag 1 | -12862.863 | (-44230.345 ,15717.029) |
| Total Precipitation Lag 2 | -25922.856 | (-55758.888 ,3119.732) |
| Relative Humidity Lag 1 | 58.862 | (-102.948 ,230.966) |
| Relative Humidity Lag 2 | 140.366 | (-22.443 ,302.756) |
| Temperature Lag 1 | -456.903 | (-495.242 ,-413.606) |
| Temperature Lag 2 | -458.163 | (-503.274 ,-419.672) |

Table 47: Associations with monthly pneumonia case counts per 100,000 in Phatthalung

| | Posterior Mean | 95% CrI |
|---|---|---|
| Case Count Lag 1 | -0.124 | (-0.248 ,0.005) |
| Case Count Lag 2 | -0.788 | (-0.907 ,-0.661) |
| Absolute Humidity Lag 1 | -25.632 | (-69.291 ,11.681) |
| Absolute Humidity Lag 2 | -8.146 | (-42.882 ,45.527) |
| Total Precipitation Lag 1 | 20075.218 | (-37367.547 ,79304.641) |
| Total Precipitation Lag 2 | 17096.278 | (-38288.728 ,73716.046) |
| Relative Humidity Lag 1 | -88.386 | (-546.304 ,335.943) |
| Relative Humidity Lag 2 | -90.347 | (-529.368 ,316.122) |
| Temperature Lag 1 | -383.236 | (-443.249 ,-314.506) |
| Temperature Lag 2 | -381.263 | (-463.877 ,-325.698) |

Table 48: Associations with monthly pneumonia case counts per 100,000 in Phayao

| | Posterior Mean | 95% CrI |
|---|---|---|
| Case Count Lag 1 | 0.312 | (0.179 ,0.443) |
| Case Count Lag 2 | -0.692 | (-0.823 ,-0.558) |
| Absolute Humidity Lag 1 | 34.7 | (-15.551 ,117.535) |
| Absolute Humidity Lag 2 | -7.617 | (-51.442 ,21.357) |
| Total Precipitation Lag 1 | 114152.809 | (-32008.724 ,270827.542) |
| Total Precipitation Lag 2 | -68038.615 | (-257866.494 ,97501.306) |
| Relative Humidity Lag 1 | 16.526 | (-479.949 ,549.693) |
| Relative Humidity Lag 2 | 263.256 | (-141.214 ,754.999) |
| Temperature Lag 1 | -187.568 | (-293.045 ,-121.076) |
| Temperature Lag 2 | -100.944 | (-137.889 ,-45.093) |

Table 49: Associations with monthly pneumonia case counts per 100,000 in Phetchabun

| | Posterior Mean | 95% CrI |
|---|---|---|
| Case Count Lag 1 | 0.413 | (0.285 ,0.535) |
| Case Count Lag 2 | -0.856 | (-0.983 ,-0.732) |
| Absolute Humidity Lag 1 | -11.471 | (-34.011 ,13.74) |
| Absolute Humidity Lag 2 | -8.505 | (-29.91 ,15.35) |
| Total Precipitation Lag 1 | 39805.481 | (-68305.676 ,151767.825) |
| Total Precipitation Lag 2 | -13549.789 | (-123730.131 ,99978.948) |
| Relative Humidity Lag 1 | 17.506 | (-289.972 ,331.362) |
| Relative Humidity Lag 2 | -114.976 | (-401.812 ,159.377) |
| Temperature Lag 1 | -168.76 | (-203.789 ,-137.41) |
| Temperature Lag 2 | -162.299 | (-195.912 ,-132.441) |

Table 50: Associations with monthly pneumonia case counts per 100,000 in Phetchaburi

| | Posterior Mean | 95% CrI |
|---|---|---|
| Case Count Lag 1 | 0.029 | (-0.103 ,0.163) |
| Case Count Lag 2 | -0.818 | (-0.946 ,-0.691) |
| Absolute Humidity Lag 1 | -17.896 | (-46.909 ,5.93) |
| Absolute Humidity Lag 2 | -7.828 | (-29.173 ,25.998) |
| Total Precipitation Lag 1 | -58228.214 | (-173926.93 ,48026.767) |
| Total Precipitation Lag 2 | 20315.244 | (-84245.185 ,131661.095) |
| Relative Humidity Lag 1 | 13.256 | (-253.463 ,306.773) |
| Relative Humidity Lag 2 | -47.85 | (-324.726 ,199.207) |
| Temperature Lag 1 | -228.097 | (-261.997 ,-186.278) |
| Temperature Lag 2 | -231.288 | (-278.755 ,-200.76) |

Table 51: Associations with monthly pneumonia case counts per 100,000 in Phichit

| | Posterior Mean | 95% CrI |
|---|---|---|
| Case Count Lag 1 | 0.14 | (0.005 ,0.27) |
| Case Count Lag 2 | -0.706 | (-0.834 ,-0.575) |
| Absolute Humidity Lag 1 | -9.419 | (-27.038 ,11) |
| Absolute Humidity Lag 2 | -8.759 | (-25.736 ,9.2) |
| Total Precipitation Lag 1 | 96468.62 | (10612.078 ,187035.824) |
| Total Precipitation Lag 2 | -49784.341 | (-146046.886 ,36542.666) |
| Relative Humidity Lag 1 | -100.651 | (-401.726 ,167.894) |
| Relative Humidity Lag 2 | 82.859 | (-167.978 ,364.842) |
| Temperature Lag 1 | -154.611 | (-184.429 ,-129.679) |
| Temperature Lag 2 | -147.95 | (-175.292 ,-122.859) |

Table 52: Associations with monthly pneumonia case counts per 100,000 in Phitsanulok

| | Posterior Mean | 95% CrI |
|---|---|---|
| Case Count Lag 1 | 0.007 | (-0.123 ,0.135) |
| Case Count Lag 2 | -0.769 | (-0.899 ,-0.642) |
| Absolute Humidity Lag 1 | -3.706 | (-61.915 ,94.188) |
| Absolute Humidity Lag 2 | 7.226 | (-49.368 ,102.155) |
| Total Precipitation Lag 1 | 408607.306 | (83604.633 ,721038.339) |
| Total Precipitation Lag 2 | -493279.752 | (-853701.912 ,-156416.594) |
| Relative Humidity Lag 1 | 189.566 | (-819.858 ,1288.945) |
| Relative Humidity Lag 2 | 675.084 | (-279.957 ,1837.485) |
| Temperature Lag 1 | -170.056 | (-299.902 ,-91.007) |
| Temperature Lag 2 | -157.653 | (-290.651 ,-79.351) |

Table 53: Associations with monthly pneumonia case counts per 100,000 in Phra Nakhon Si Ayutthaya

| | Posterior Mean | 95% CrI |
|---|---|---|
| Case Count Lag 1 | 0.174 | (0.05 ,0.304) |
| Case Count Lag 2 | -1.104 | (-1.232 ,-0.974) |
| Absolute Humidity Lag 1 | -16.772 | (-53.304 ,21.058) |
| Absolute Humidity Lag 2 | -3.445 | (-38.777 ,36.605) |
| Total Precipitation Lag 1 | -212677.167 | (-386572.608 ,-38557.432) |
| Total Precipitation Lag 2 | 917.076 | (-152989.064 ,156417.596) |
| Relative Humidity Lag 1 | 333.055 | (-36.077 ,716.346) |
| Relative Humidity Lag 2 | -123.586 | (-463.071 ,189.794) |
| Temperature Lag 1 | -213.011 | (-275.856 ,-152.584) |
| Temperature Lag 2 | -198.098 | (-263.371 ,-139.884) |

Table 54: Associations with monthly pneumonia case counts per 100,000 in Phrae

| | Posterior Mean | 95% CrI |
|---|---|---|
| Case Count Lag 1 | 0.085 | (-0.042 ,0.219) |
| Case Count Lag 2 | -0.279 | (-0.414 ,-0.14) |
| Absolute Humidity Lag 1 | -6.071 | (-17.819 ,15.579) |
| Absolute Humidity Lag 2 | -9.236 | (-27.639 ,3.057) |
| Total Precipitation Lag 1 | 3082.979 | (-72210.844 ,81526.208) |
| Total Precipitation Lag 2 | -51951.796 | (-143524.045 ,35764.442) |
| Relative Humidity Lag 1 | 94.648 | (-130.04 ,353.847) |
| Relative Humidity Lag 2 | 37.185 | (-180.204 ,258.807) |
| Temperature Lag 1 | -135.459 | (-163.655 ,-119.918) |
| Temperature Lag 2 | -126.083 | (-143.027 ,-100.477) |

Table 55: Associations with monthly pneumonia case counts per 100,000 in Phuket

| | Posterior Mean | 95% CrI |
|---|---|---|
| Case Count Lag 1 | -0.121 | (-0.249 ,0.002) |
| Case Count Lag 2 | -0.719 | (-0.839 ,-0.598) |
| Absolute Humidity Lag 1 | -20.513 | (-105.513 ,81.168) |
| Absolute Humidity Lag 2 | -29.814 | (-119.081 ,65.755) |
| Total Precipitation Lag 1 | -60365.508 | (-171958.271 ,39765.436) |
| Total Precipitation Lag 2 | -19278.569 | (-124205.135 ,77022.051) |
| Relative Humidity Lag 1 | 254.064 | (-416.635 ,1000.93) |
| Relative Humidity Lag 2 | 560.695 | (-77.269 ,1221.037) |
| Temperature Lag 1 | -523.998 | (-677.61 ,-387.243) |
| Temperature Lag 2 | -527.77 | (-674.549 ,-388.017) |

Table 56: Associations with monthly pneumonia case counts per 100,000 in Prachin Buri

| | Posterior Mean | 95% CrI |
|---|---|---|
| Case Count Lag 1 | 0.332 | (0.207 ,0.465) |
| Case Count Lag 2 | -0.754 | (-0.881 ,-0.625) |
| Absolute Humidity Lag 1 | -15.434 | (-39.252 ,9.606) |
| Absolute Humidity Lag 2 | -12.329 | (-39.443 ,9.814) |
| Total Precipitation Lag 1 | 4142.01 | (-69751.449 ,79057.22) |
| Total Precipitation Lag 2 | -12282.3 | (-91232.135 ,62644.227) |
| Relative Humidity Lag 1 | 135.481 | (-92.3 ,390.002) |
| Relative Humidity Lag 2 | -137.403 | (-371.929 ,84.871) |
| Temperature Lag 1 | -225.269 | (-263.058 ,-191.099) |
| Temperature Lag 2 | -216.837 | (-249.418 ,-177.448) |

Table 57: Associations with monthly pneumonia case counts per 100,000 in Prachuap Khiri Khan

| | Posterior Mean | 95% CrI |
|---|---|---|
| Case Count Lag 1 | 0.165 | (0.033 ,0.294) |
| Case Count Lag 2 | -0.544 | (-0.669 ,-0.416) |
| Absolute Humidity Lag 1 | -17.098 | (-49.339 ,22.189) |
| Absolute Humidity Lag 2 | -9.307 | (-40.728 ,33.639) |
| Total Precipitation Lag 1 | -48725.592 | (-162624.129 ,60404.006) |
| Total Precipitation Lag 2 | 91320.103 | (-12537.651 ,206820.897) |
| Relative Humidity Lag 1 | 125.077 | (-178.732 ,450.389) |
| Relative Humidity Lag 2 | -307.932 | (-609.925 ,-31.926) |
| Temperature Lag 1 | -296.523 | (-355.656 ,-249.499) |
| Temperature Lag 2 | -286.877 | (-348.878 ,-239.965) |

Table 58: Associations with monthly pneumonia case counts per 100,000 in Ranong

| | Posterior Mean | 95% CrI |
|---|---|---|
| Case Count Lag 1 | -0.063 | (-0.19 ,0.077) |
| Case Count Lag 2 | -0.703 | (-0.833 ,-0.58) |
| Absolute Humidity Lag 1 | -22.8 | (-32.518 ,-16.105) |
| Absolute Humidity Lag 2 | -21.797 | (-32.066 ,-14.968) |
| Total Precipitation Lag 1 | -13626.503 | (-25019.54 ,-2149.509) |
| Total Precipitation Lag 2 | 9519.282 | (-1574.804 ,21252.477) |
| Relative Humidity Lag 1 | 9.534 | (-49.565 ,76.979) |
| Relative Humidity Lag 2 | -34.417 | (-94.457 ,18.215) |
| Temperature Lag 1 | -390.39 | (-400.586 ,-376.017) |
| Temperature Lag 2 | -389.044 | (-399.423 ,-374.041) |

Table 59: Associations with monthly pneumonia case counts per 100,000 in Ratchaburi

| | Posterior Mean | 95% CrI |
|---|---|---|
| Case Count Lag 1 | -0.084 | (-0.212 ,0.047) |
| Case Count Lag 2 | -0.822 | (-0.954 ,-0.699) |
| Absolute Humidity Lag 1 | -20.575 | (-64.251 ,9.944) |
| Absolute Humidity Lag 2 | -5.656 | (-36.533 ,35.753) |
| Total Precipitation Lag 1 | -29732.92 | (-228127.66 ,153877.634) |
| Total Precipitation Lag 2 | -31109.661 | (-214484.054 ,148551.412) |
| Relative Humidity Lag 1 | 106.909 | (-285.743 ,546.258) |
| Relative Humidity Lag 2 | -122.405 | (-530.391 ,290.7) |
| Temperature Lag 1 | -196.843 | (-243.168 ,-132.684) |
| Temperature Lag 2 | -197.96 | (-259.082 ,-151.156) |

Table 60: Associations with monthly pneumonia case counts per 100,000 in Rayong

| | Posterior Mean | 95% CrI |
|---|---|---|
| Case Count Lag 1 | 0.411 | (0.283 ,0.537) |
| Case Count Lag 2 | -0.968 | (-1.089 ,-0.839) |
| Absolute Humidity Lag 1 | 4.202 | (-121.841 ,225.459) |
| Absolute Humidity Lag 2 | 52.519 | (-68.602 ,297.917) |
| Total Precipitation Lag 1 | -41088.921 | (-323356.349 ,236002.122) |
| Total Precipitation Lag 2 | 73185.388 | (-180847.579 ,337369.55) |
| Relative Humidity Lag 1 | -276.257 | (-1279.65 ,677.549) |
| Relative Humidity Lag 2 | -383.186 | (-1290.566 ,478.111) |
| Temperature Lag 1 | -377.556 | (-706.483 ,-189.26) |
| Temperature Lag 2 | -352.467 | (-712.168 ,-169.675) |

Table 61: Associations with monthly pneumonia case counts per 100,000 in Roi Et

| | Posterior Mean | 95% CrI |
|---|---|---|
| Case Count Lag 1 | 0.451 | (0.321 ,0.583) |
| Case Count Lag 2 | -0.641 | (-0.776 ,-0.511) |
| Absolute Humidity Lag 1 | -9.049 | (-25.091 ,8.201) |
| Absolute Humidity Lag 2 | -7.029 | (-21.647 ,9.444) |
| Total Precipitation Lag 1 | 75588.774 | (-8267.288 ,166018.495) |
| Total Precipitation Lag 2 | -11587.712 | (-96115.924 ,78909.376) |
| Relative Humidity Lag 1 | -150.345 | (-416.162 ,83.959) |
| Relative Humidity Lag 2 | -63.972 | (-282.714 ,149.239) |
| Temperature Lag 1 | -136.04 | (-160.855 ,-113.615) |
| Temperature Lag 2 | -131.296 | (-154.991 ,-110.814) |

Table 62: Associations with monthly pneumonia case counts per 100,000 in Sa Kaeo

| | Posterior Mean | 95% CrI |
|---|---|---|
| Case Count Lag 1 | 0.404 | (0.272 ,0.529) |
| Case Count Lag 2 | -0.957 | (-1.081 ,-0.828) |
| Absolute Humidity Lag 1 | -15.417 | (-26.594 ,-9.706) |
| Absolute Humidity Lag 2 | -11.817 | (-18.121 ,-3.649) |
| Total Precipitation Lag 1 | -9698.177 | (-38650.006 ,16285.122) |
| Total Precipitation Lag 2 | -10960.117 | (-39112.001 ,16270.123) |
| Relative Humidity Lag 1 | 28.808 | (-41.533 ,104.533) |
| Relative Humidity Lag 2 | -19.234 | (-81.97 ,43.238) |
| Temperature Lag 1 | -211.253 | (-219.632 ,-194.727) |
| Temperature Lag 2 | -212.282 | (-224.223 ,-202.909) |

Table 63: Associations with monthly pneumonia case counts per 100,000 in Sakon Nakhon

| | Posterior Mean | 95% CrI |
|---|---|---|
| Case Count Lag 1 | 0.005 | (-0.127 ,0.137) |
| Case Count Lag 2 | -0.916 | (-1.053 ,-0.784) |
| Absolute Humidity Lag 1 | -9.809 | (-19.446 ,-1.407) |
| Absolute Humidity Lag 2 | -7.112 | (-15.929 ,1.649) |
| Total Precipitation Lag 1 | 89641.829 | (44717.779 ,137101.905) |
| Total Precipitation Lag 2 | -19640.117 | (-71113.406 ,29366.267) |
| Relative Humidity Lag 1 | -37.82 | (-183.13 ,97.957) |
| Relative Humidity Lag 2 | -86.106 | (-226.769 ,39.235) |
| Temperature Lag 1 | -119.733 | (-131.117 ,-106.787) |
| Temperature Lag 2 | -116.65 | (-128.267 ,-105.001) |

Table 64: Associations with monthly pneumonia case counts per 100,000 in Samut Prakan

| | Posterior Mean | 95% CrI |
|---|---|---|
| Case Count Lag 1 | 0.376 | (0.252 ,0.498) |
| Case Count Lag 2 | -1.034 | (-1.158 ,-0.913) |
| Absolute Humidity Lag 1 | -14.861 | (-84.358 ,82.932) |
| Absolute Humidity Lag 2 | 30.06 | (-38.49 ,152.941) |
| Total Precipitation Lag 1 | -291342.284 | (-616277.846 ,30302.952) |
| Total Precipitation Lag 2 | 38398.428 | (-231994.704 ,321868.439) |
| Relative Humidity Lag 1 | 654.906 | (-165.536 ,1506.683) |
| Relative Humidity Lag 2 | -237.373 | (-991.187 ,518.689) |
| Temperature Lag 1 | -248.762 | (-404.644 ,-142.389) |
| Temperature Lag 2 | -247.74 | (-437.443 ,-140.316) |

Table 65: Associations with monthly pneumonia case counts per 100,000 in Samut Sakhon

| | Posterior Mean | 95% CrI |
|---|---|---|
| Case Count Lag 1 | 0.337 | (0.205 ,0.472) |
| Case Count Lag 2 | -0.443 | (-0.576 ,-0.319) |
| Absolute Humidity Lag 1 | -19.032 | (-68.284 ,20.126) |
| Absolute Humidity Lag 2 | -11.995 | (-56.665 ,33.466) |
| Total Precipitation Lag 1 | -104817.225 | (-309333.951 ,90918.092) |
| Total Precipitation Lag 2 | -4641.412 | (-186349.846 ,182311.806) |
| Relative Humidity Lag 1 | 356.517 | (-152.497 ,885.17) |
| Relative Humidity Lag 2 | -174.894 | (-645.442 ,277.841) |
| Temperature Lag 1 | -218.252 | (-280.636 ,-141.684) |
| Temperature Lag 2 | -215.49 | (-285.681 ,-144.888) |

Table 66: Associations with monthly pneumonia case counts per 100,000 in Samut Songkhram

| | Posterior Mean | 95% CrI |
|---|---|---|
| Case Count Lag 1 | -0.169 | (-0.296 ,-0.043) |
| Case Count Lag 2 | -0.913 | (-1.039 ,-0.788) |
| Absolute Humidity Lag 1 | -14.184 | (-21.967 ,-9.68) |
| Absolute Humidity Lag 2 | -11.187 | (-15.807 ,-3.544) |
| Total Precipitation Lag 1 | 2217.501 | (-23441.905 ,28850.674) |
| Total Precipitation Lag 2 | 3874.635 | (-20568.495 ,29647.906) |
| Relative Humidity Lag 1 | -8.227 | (-71.226 ,55.834) |
| Relative Humidity Lag 2 | -51.604 | (-110.955 ,5.282) |
| Temperature Lag 1 | -209.226 | (-216.384 ,-197.189) |
| Temperature Lag 2 | -209.289 | (-220.748 ,-202.195) |

Table 67: Associations with monthly pneumonia case counts per 100,000 in Saraburi

| | Posterior Mean | 95% CrI |
|---|---|---|
| Case Count Lag 1 | 0.144 | (0.012 ,0.28) |
| Case Count Lag 2 | -0.741 | (-0.88 ,-0.616) |
| Absolute Humidity Lag 1 | -15.917 | (-35.445 ,-2.087) |
| Absolute Humidity Lag 2 | -8.382 | (-20.925 ,11.268) |
| Total Precipitation Lag 1 | -5417.585 | (-73270.551 ,59756.411) |
| Total Precipitation Lag 2 | -34494.611 | (-104744.817 ,25978.743) |
| Relative Humidity Lag 1 | 53.504 | (-106.628 ,231.263) |
| Relative Humidity Lag 2 | -10.947 | (-169.991 ,151.23) |
| Temperature Lag 1 | -200.972 | (-222.73 ,-171.31) |
| Temperature Lag 2 | -201.122 | (-230.897 ,-181.748) |

Table 68: Associations with monthly pneumonia case counts per 100,000 in Satun

| | Posterior Mean | 95% CrI |
|---|---|---|
| Case Count Lag 1 | -0.296 | (-0.423 ,-0.165) |
| Case Count Lag 2 | -1.024 | (-1.15 ,-0.901) |
| Absolute Humidity Lag 1 | -26.172 | (-37.897 ,-16.98) |
| Absolute Humidity Lag 2 | -24.786 | (-35.081 ,-14.414) |
| Total Precipitation Lag 1 | -9175.085 | (-23452.348 ,4031.683) |
| Total Precipitation Lag 2 | 3256.315 | (-9738.827 ,16999.89) |
| Relative Humidity Lag 1 | 11.78 | (-66.138 ,97.682) |
| Relative Humidity Lag 2 | -21.612 | (-90.544 ,45.824) |
| Temperature Lag 1 | -459.93 | (-474.905 ,-441.878) |
| Temperature Lag 2 | -459.53 | (-474.905 ,-443.171) |

Table 69: Associations with monthly pneumonia case counts per 100,000 in Si Sa Ket

| | Posterior Mean | 95% CrI |
|---|---|---|
| Case Count Lag 1 | 0.252 | (0.121 ,0.377) |
| Case Count Lag 2 | -1.064 | (-1.192 ,-0.935) |
| Absolute Humidity Lag 1 | -13.493 | (-38.513 ,6.225) |
| Absolute Humidity Lag 2 | -4.435 | (-20.986 ,23.69) |
| Total Precipitation Lag 1 | 12320.353 | (-79699.315 ,109481.15) |
| Total Precipitation Lag 2 | -80830.059 | (-181126.403 ,16616.26) |
| Relative Humidity Lag 1 | 6.969 | (-265.688 ,279.714) |
| Relative Humidity Lag 2 | -5.253 | (-239.852 ,246.692) |
| Temperature Lag 1 | -151.582 | (-179.893 ,-116.16) |
| Temperature Lag 2 | -153.989 | (-193.886 ,-130.056) |

Table 70: Associations with monthly pneumonia case counts per 100,000 in Sing Buri

| | Posterior Mean | 95% CrI |
|---|---|---|
| Case Count Lag 1 | 0.048 | (-0.083 ,0.184) |
| Case Count Lag 2 | -0.685 | (-0.817 ,-0.56) |
| Absolute Humidity Lag 1 | -12.03 | (-20.127 ,-7.328) |
| Absolute Humidity Lag 2 | -10.678 | (-16.795 ,-5.174) |
| Total Precipitation Lag 1 | -25988.561 | (-61793.728 ,7913.82) |
| Total Precipitation Lag 2 | -808.567 | (-33775.805 ,31466.357) |
| Relative Humidity Lag 1 | 39.206 | (-32.234 ,119.412) |
| Relative Humidity Lag 2 | -43.213 | (-111.471 ,17.888) |
| Temperature Lag 1 | -172.554 | (-180.263 ,-159.014) |
| Temperature Lag 2 | -172.767 | (-181.933 ,-162.724) |

Table 71: Associations with monthly pneumonia case counts per 100,000 in Songkhla

| | Posterior Mean | 95% CrI |
|---|---|---|
| Case Count Lag 1 | 0.129 | (0.008 ,0.259) |
| Case Count Lag 2 | -0.957 | (-1.083 ,-0.83) |
| Absolute Humidity Lag 1 | -15.175 | (-87.409 ,59.101) |
| Absolute Humidity Lag 2 | -28.607 | (-122.613 ,28.312) |
| Total Precipitation Lag 1 | 85030.869 | (-17587.49 ,196255.864) |
| Total Precipitation Lag 2 | 31212.183 | (-70985.808 ,134476.364) |
| Relative Humidity Lag 1 | -340.356 | (-941.875 ,210.117) |
| Relative Humidity Lag 2 | 466.885 | (-60.021 ,991.765) |
| Temperature Lag 1 | -385.661 | (-500.497 ,-269.117) |
| Temperature Lag 2 | -383.991 | (-472.61 ,-237.656) |

Table 72: Associations with monthly pneumonia case counts per 100,000 in Sukhothai

| | Posterior Mean | 95% CrI |
|---|---|---|
| Case Count Lag 1 | 0.108 | (-0.025 ,0.238) |
| Case Count Lag 2 | -0.552 | (-0.677 ,-0.423) |
| Absolute Humidity Lag 1 | -8.388 | (-32.698 ,14.732) |
| Absolute Humidity Lag 2 | -8.2 | (-32.069 ,14.091) |
| Total Precipitation Lag 1 | 61353.285 | (-88074.002 ,219657.47) |
| Total Precipitation Lag 2 | -80707.315 | (-247725.854 ,84802.064) |
| Relative Humidity Lag 1 | 164.96 | (-212.798 ,591.018) |
| Relative Humidity Lag 2 | -25.546 | (-405.728 ,342.207) |
| Temperature Lag 1 | -125.944 | (-159.467 ,-90.24) |
| Temperature Lag 2 | -127.631 | (-161.548 ,-93.498) |

Table 73: Associations with monthly pneumonia case counts per 100,000 in Suphan Buri

| | Posterior Mean | 95% CrI |
|---|---|---|
| Case Count Lag 1 | 0.242 | (0.118 ,0.367) |
| Case Count Lag 2 | -0.936 | (-1.061 ,-0.811) |
| Absolute Humidity Lag 1 | -10.326 | (-28.174 ,14.609) |
| Absolute Humidity Lag 2 | -8.474 | (-28.081 ,12.757) |
| Total Precipitation Lag 1 | -58694.655 | (-200608.675 ,71966.958) |
| Total Precipitation Lag 2 | 29885.008 | (-93682.986 ,162265.326) |
| Relative Humidity Lag 1 | 89.343 | (-181.841 ,384.393) |
| Relative Humidity Lag 2 | -152.446 | (-416.99 ,93.157) |
| Temperature Lag 1 | -180.597 | (-220.435 ,-153.442) |
| Temperature Lag 2 | -168.163 | (-201.255 ,-138.105) |

Table 74: Associations with monthly pneumonia case counts per 100,000 in Surat Thani

| | Posterior Mean | 95% CrI |
|---|---|---|
| Case Count Lag 1 | 0.106 | (-0.023 ,0.228) |
| Case Count Lag 2 | -1.141 | (-1.265 ,-1.018) |
| Absolute Humidity Lag 1 | -28.618 | (-128.485 ,60.958) |
| Absolute Humidity Lag 2 | -9.991 | (-106.706 ,78.218) |
| Total Precipitation Lag 1 | -75940.8 | (-220733.596 ,61409.111) |
| Total Precipitation Lag 2 | 17504.335 | (-124498.514 ,157788.748) |
| Relative Humidity Lag 1 | -203.98 | (-1106.606 ,597.66) |
| Relative Humidity Lag 2 | 339.682 | (-412.029 ,1124.218) |
| Temperature Lag 1 | -357.466 | (-505.461 ,-206.772) |
| Temperature Lag 2 | -317.573 | (-450.837 ,-165.306) |

Table 75: Associations with monthly pneumonia case counts per 100,000 in Surin

| | Posterior Mean | 95% CrI |
|---|---|---|
| Case Count Lag 1 | 0.508 | (0.382 ,0.633) |
| Case Count Lag 2 | -0.788 | (-0.917 ,-0.659) |
| Absolute Humidity Lag 1 | -16.694 | (-71.226 ,17.916) |
| Absolute Humidity Lag 2 | -3.588 | (-37.772 ,45.651) |
| Total Precipitation Lag 1 | 138029.455 | (-54695.43 ,356207.063) |
| Total Precipitation Lag 2 | -132895.82 | (-336928.599 ,68510.809) |
| Relative Humidity Lag 1 | -199.571 | (-832.625 ,320.916) |
| Relative Humidity Lag 2 | 28.027 | (-450.884 ,549.683) |
| Temperature Lag 1 | -144.721 | (-194.25 ,-68.585) |
| Temperature Lag 2 | -148.132 | (-220.949 ,-98.151) |

Table 76: Associations with monthly pneumonia case counts per 100,000 in Tak

| | Posterior Mean | 95% CrI |
|---|---|---|
| Case Count Lag 1 | 0.07 | (-0.061 ,0.205) |
| Case Count Lag 2 | -0.702 | (-0.83 ,-0.576) |
| Absolute Humidity Lag 1 | -11.067 | (-32.537 ,10.89) |
| Absolute Humidity Lag 2 | -1.383 | (-18.036 ,32.114) |
| Total Precipitation Lag 1 | 112228.702 | (36744.876 ,190290.868) |
| Total Precipitation Lag 2 | -78895.401 | (-168074.375 ,6225.647) |
| Relative Humidity Lag 1 | -77.185 | (-318.987 ,152.737) |
| Relative Humidity Lag 2 | 82.281 | (-134.18 ,360.422) |
| Temperature Lag 1 | -163.863 | (-191.606 ,-138.47) |
| Temperature Lag 2 | -168.886 | (-210.542 ,-147.131) |

Table 77: Associations with monthly pneumonia case counts per 100,000 in Trang

| | Posterior Mean | 95% CrI |
|---|---|---|
| Case Count Lag 1 | -0.367 | (-0.495 ,-0.246) |
| Case Count Lag 2 | -0.995 | (-1.119 ,-0.879) |
| Absolute Humidity Lag 1 | -53.289 | (-174.684 ,-0.704) |
| Absolute Humidity Lag 2 | -18.917 | (-94.052 ,47.427) |
| Total Precipitation Lag 1 | -23965.47 | (-125083.62 ,71911.148) |
| Total Precipitation Lag 2 | -45350.658 | (-148556.888 ,56995.873) |
| Relative Humidity Lag 1 | -112.29 | (-703.991 ,437.118) |
| Relative Humidity Lag 2 | 231.733 | (-258.892 ,750.955) |
| Temperature Lag 1 | -412.411 | (-498.623 ,-218.266) |
| Temperature Lag 2 | -416.531 | (-517.536 ,-301.239) |

Table 78: Associations with monthly pneumonia case counts per 100,000 in Trat

| | Posterior Mean | 95% CrI |
|---|---|---|
| Case Count Lag 1 | 0.244 | (0.126 ,0.368) |
| Case Count Lag 2 | -0.942 | (-1.059 ,-0.821) |
| Absolute Humidity Lag 1 | -23.061 | (-54.113 ,11.148) |
| Absolute Humidity Lag 2 | 23.971 | (-21.79 ,108.358) |
| Total Precipitation Lag 1 | 3251.132 | (-25955.722 ,30887.594) |
| Total Precipitation Lag 2 | 8542.239 | (-19671.465 ,38904.309) |
| Relative Humidity Lag 1 | 10.204 | (-126.779 ,150.758) |
| Relative Humidity Lag 2 | -60.286 | (-213.785 ,103.758) |
| Temperature Lag 1 | -392.689 | (-442.108 ,-348.729) |
| Temperature Lag 2 | -442.029 | (-564.546 ,-375.772) |

Table 79: Associations with monthly pneumonia case counts per 100,000 in Ubon Ratchathani

| | Posterior Mean | 95% CrI |
|---|---|---|
| Case Count Lag 1 | 0.435 | (0.298 ,0.565) |
| Case Count Lag 2 | -0.786 | (-0.921 ,-0.65) |
| Absolute Humidity Lag 1 | -13.343 | (-133.381 ,109.018) |
| Absolute Humidity Lag 2 | 17.942 | (-75.567 ,185.14) |
| Total Precipitation Lag 1 | -331664.708 | (-843317.713 ,127092.738) |
| Total Precipitation Lag 2 | -528567.949 | (-1056474.497 ,-26085.954) |
| Relative Humidity Lag 1 | 1416.037 | (-93.186 ,3048.207) |
| Relative Humidity Lag 2 | -165.969 | (-1460.076 ,1128.907) |
| Temperature Lag 1 | -163.683 | (-334.75 ,8.665) |
| Temperature Lag 2 | -182.889 | (-417.551 ,-53.178) |

Table 80: Associations with monthly pneumonia case counts per 100,000 in Udon Thani

| | Posterior Mean | 95% CrI |
|---|---|---|
| Case Count Lag 1 | 0.46 | (0.333 ,0.587) |
| Case Count Lag 2 | -0.831 | (-0.961 ,-0.704) |
| Absolute Humidity Lag 1 | -9.185 | (-30.753 ,14.194) |
| Absolute Humidity Lag 2 | -8.677 | (-36.473 ,8.898) |
| Total Precipitation Lag 1 | 20428.995 | (-113785.219 ,162523.874) |
| Total Precipitation Lag 2 | 62756.189 | (-86430.48 ,219957.224) |
| Relative Humidity Lag 1 | 78.742 | (-311.094 ,465.457) |
| Relative Humidity Lag 2 | -246.592 | (-620.137 ,96.489) |
| Temperature Lag 1 | -121.624 | (-154.531 ,-92.755) |
| Temperature Lag 2 | -113.169 | (-136.822 ,-76.407) |

Table 81: Associations with monthly pneumonia case counts per 100,000 in Uthai Thani

| | Posterior Mean | 95% CrI |
|---|---|---|
| Case Count Lag 1 | -0.027 | (-0.155 ,0.103) |
| Case Count Lag 2 | -0.732 | (-0.86 ,-0.615) |
| Absolute Humidity Lag 1 | -9.833 | (-20.194 ,-2.422) |
| Absolute Humidity Lag 2 | -7.655 | (-15.193 ,3.762) |
| Total Precipitation Lag 1 | 2336.143 | (-41509.788 ,45536.585) |
| Total Precipitation Lag 2 | -62164.95 | (-109008.053 ,-15875.403) |
| Relative Humidity Lag 1 | 56.2 | (-55.745 ,170.348) |
| Relative Humidity Lag 2 | 52.906 | (-58.364 ,182.519) |
| Temperature Lag 1 | -149.394 | (-159.425 ,-135.195) |
| Temperature Lag 2 | -152.456 | (-167.761 ,-142.529) |

Table 82: Associations with monthly pneumonia case counts per 100,000 in Uttaradit

| | Posterior Mean | 95% CrI |
|---|---|---|
| Case Count Lag 1 | 0.172 | (0.045 ,0.302) |
| Case Count Lag 2 | -0.907 | (-1.034 ,-0.777) |
| Absolute Humidity Lag 1 | -3.659 | (-28.377 ,42.782) |
| Absolute Humidity Lag 2 | -9.232 | (-43.558 ,17.366) |
| Total Precipitation Lag 1 | 123116.565 | (-18420.788 ,269787.379) |
| Total Precipitation Lag 2 | -242358.072 | (-405735.732 ,-81549.894) |
| Relative Humidity Lag 1 | 217.983 | (-241.588 ,711.587) |
| Relative Humidity Lag 2 | 123.635 | (-283.354 ,565.99) |
| Temperature Lag 1 | -146.789 | (-207.443 ,-114.288) |
| Temperature Lag 2 | -127.771 | (-162.502 ,-80.934) |

Table 83: Associations with monthly pneumonia case counts per 100,000 in Yala

| | Posterior Mean | 95% CrI |
|---|---|---|
| Case Count Lag 1 | -0.109 | (-0.237 ,0.019) |
| Case Count Lag 2 | -0.72 | (-0.84 ,-0.59) |
| Absolute Humidity Lag 1 | -18.514 | (-28.572 ,-6.817) |
| Absolute Humidity Lag 2 | -16.947 | (-29.369 ,-6.432) |
| Total Precipitation Lag 1 | -9240.933 | (-33575.809 ,14612.772) |
| Total Precipitation Lag 2 | -1229.532 | (-24934.411 ,22353.015) |
| Relative Humidity Lag 1 | 2.147 | (-109.365 ,128.03) |
| Relative Humidity Lag 2 | 41.895 | (-61.561 ,153.486) |
| Temperature Lag 1 | -335.204 | (-352.672 ,-320.334) |
| Temperature Lag 2 | -329.262 | (-343.786 ,-311.67) |

Table 84: Associations with monthly pneumonia case counts per 100,000 in Yasothon

| | Posterior Mean | 95% CrI |
|---|---|---|
| Case Count Lag 1 | 0.24 | (0.109 ,0.363) |
| Case Count Lag 2 | -0.851 | (-0.975 ,-0.717) |
| Absolute Humidity Lag 1 | -8.748 | (-21.639 ,4.035) |
| Absolute Humidity Lag 2 | -4.685 | (-14.512 ,14.784) |
| Total Precipitation Lag 1 | 40559.541 | (-18891.879 ,104302.857) |
| Total Precipitation Lag 2 | -50883.228 | (-116303.03 ,13341.183) |
| Relative Humidity Lag 1 | 22.543 | (-156.403 ,222.707) |
| Relative Humidity Lag 2 | -36.178 | (-215.33 ,139.31) |
| Temperature Lag 1 | -136.761 | (-155.005 ,-119.01) |
| Temperature Lag 2 | -138.006 | (-165.014 ,-124.086) |

## 5.2 Coefficient output for URTI case counts per 100,000 as the dependent variable

Table 85: Associations with monthly URTI case counts per 100,000 in Amnat Charoen

| | Posterior Mean | 95% CrI |
|---|---|---|
| Case Count Lag 1 | 1.046 | (0.654 ,1.44) |
| Case Count Lag 2 | -0.562 | (-0.951 ,-0.178) |
| Absolute Humidity Lag 1 | -8.52 | (-22.378 ,12.64) |
| Absolute Humidity Lag 2 | -3.137 | (-15.908 ,21.516) |
| Total Precipitation Lag 1 | 79238.989 | (6170.272 ,152718.454) |
| Total Precipitation Lag 2 | -27829.98 | (-106605.501 ,44510.248) |
| Relative Humidity Lag 1 | -62.242 | (-319.79 ,171.817) |
| Relative Humidity Lag 2 | 35.972 | (-185.549 ,281.482) |
| Temperature Lag 1 | -142.991 | (-172.522 ,-122.878) |
| Temperature Lag 2 | -139.301 | (-173.804 ,-121.362) |

Table 86: Associations with monthly URTI case counts per 100,000 in Ang Thong

| | Posterior Mean | 95% CrI |
|---|---|---|
| Case Count Lag 1 | 0.559 | (0.375 ,0.747) |
| Case Count Lag 2 | -0.636 | (-0.834 ,-0.451) |
| Absolute Humidity Lag 1 | -19.117 | (-39.715 ,-6.949) |
| Absolute Humidity Lag 2 | -6.237 | (-18.142 ,12.353) |
| Total Precipitation Lag 1 | -103686.1 | (-174517.888 ,-34154.682) |
| Total Precipitation Lag 2 | -72800.228 | (-139972.863 ,-5616.399) |
| Relative Humidity Lag 1 | 115.882 | (-29.723 ,266.102) |
| Relative Humidity Lag 2 | -29.62 | (-159.271 ,100.386) |
| Temperature Lag 1 | -186.213 | (-205.893 ,-153.129) |
| Temperature Lag 2 | -188.359 | (-218.19 ,-168.792) |

Table 87: Associations with monthly URTI case counts per 100,000 in Bangkok

| | Posterior Mean | 95% CrI |
|---|---|---|
| Case Count Lag 1 | -0.516 | (-0.532 ,-0.498) |
| Case Count Lag 2 | -0.674 | (-0.691 ,-0.657) |
| Absolute Humidity Lag 1 | -41.851 | (-175.848 ,63.4) |
| Absolute Humidity Lag 2 | 61.356 | (-46.036 ,237.278) |
| Total Precipitation Lag 1 | -563466.53 | (-1024870.168 ,-107865.699) |
| Total Precipitation Lag 2 | -30118.482 | (-399903.383 ,346428.247) |
| Relative Humidity Lag 1 | 942.964 | (-252.345 ,2214.542) |
| Relative Humidity Lag 2 | -393.562 | (-1525.87 ,695.784) |
| Temperature Lag 1 | -215.342 | (-386.031 ,-8.033) |
| Temperature Lag 2 | -230.097 | (-504.559 ,-62.107) |

Table 88: Associations with monthly URTI case counts per 100,000 in Buri Ram

| | Posterior Mean | 95% CrI |
|---|---|---|
| Case Count Lag 1 | -0.032 | (-0.119 ,0.052) |
| Case Count Lag 2 | -0.644 | (-0.732 ,-0.562) |
| Absolute Humidity Lag 1 | -18.893 | (-64.945 ,28.927) |
| Absolute Humidity Lag 2 | 5.302 | (-31.706 ,67.087) |
| Total Precipitation Lag 1 | -24863.793 | (-279384.214 ,205968.38) |
| Total Precipitation Lag 2 | -425919.853 | (-675356.952 ,-183848.621) |
| Relative Humidity Lag 1 | 431.67 | (-168.392 ,1045.703) |
| Relative Humidity Lag 2 | 39.23 | (-507.449 ,636.142) |
| Temperature Lag 1 | -164.504 | (-233.482 ,-98.92) |
| Temperature Lag 2 | -159.153 | (-248.654 ,-106.602) |

Table 89: Associations with monthly URTI case counts per 100,000 in Chachoengsao

| | Posterior Mean | 95% CrI |
|---|---|---|
| Case Count Lag 1 | 0.109 | (0.009 ,0.212) |
| Case Count Lag 2 | -0.688 | (-0.788 ,-0.587) |
| Absolute Humidity Lag 1 | -44.369 | (-124.296 ,-0.419) |
| Absolute Humidity Lag 2 | -6.114 | (-52.63 ,45.367) |
| Total Precipitation Lag 1 | -15902.261 | (-159634.273 ,130361.401) |
| Total Precipitation Lag 2 | -67635.075 | (-209726.265 ,70131.403) |
| Relative Humidity Lag 1 | 228.395 | (-170.894 ,660.928) |
| Relative Humidity Lag 2 | -114.047 | (-487.51 ,258.547) |
| Temperature Lag 1 | -230.951 | (-297.072 ,-109.953) |
| Temperature Lag 2 | -236.022 | (-311.442 ,-162.169) |

Table 90: Associations with monthly URTI case counts per 100,000 in Chai Nat

| | Posterior Mean | 95% CrI |
|---|---|---|
| Case Count Lag 1 | -0.116 | (-0.225 ,-0.014) |
| Case Count Lag 2 | -0.566 | (-0.671 ,-0.463) |
| Absolute Humidity Lag 1 | -11.901 | (-17.958 ,-8.009) |
| Absolute Humidity Lag 2 | -9.546 | (-14.379 ,-6.021) |
| Total Precipitation Lag 1 | -11592.214 | (-36309.401 ,12514.991) |
| Total Precipitation Lag 2 | -24747.119 | (-49652.204 ,446.532) |
| Relative Humidity Lag 1 | 22.587 | (-35.829 ,81.68) |
| Relative Humidity Lag 2 | -44.726 | (-100.6 ,8.091) |
| Temperature Lag 1 | -154.698 | (-160.707 ,-145.231) |
| Temperature Lag 2 | -153.144 | (-158.94 ,-145.283) |

Table 91: Associations with monthly URTI case counts per 100,000 in Chaiyaphum

| | Posterior Mean | 95% CrI |
|---|---|---|
| Case Count Lag 1 | 0.136 | (0.029 ,0.242) |
| Case Count Lag 2 | -0.485 | (-0.585 ,-0.378) |
| Absolute Humidity Lag 1 | -17.062 | (-55.238 ,17.315) |
| Absolute Humidity Lag 2 | -1.367 | (-29.753 ,43.186) |
| Total Precipitation Lag 1 | 11103.141 | (-139806.923 ,158166.184) |
| Total Precipitation Lag 2 | -180651.991 | (-339367.252 ,-25736.124) |
| Relative Humidity Lag 1 | 366.236 | (-62.223 ,831.766) |
| Relative Humidity Lag 2 | 45.357 | (-391.648 ,499.791) |
| Temperature Lag 1 | -157.559 | (-203.122 ,-107.915) |
| Temperature Lag 2 | -158.219 | (-216.999 ,-119.953) |

Table 92: Associations with monthly URTI case counts per 100,000 in Chanthaburi

| | Posterior Mean | 95% CrI |
|---|---|---|
| Case Count Lag 1 | -0.452 | (-0.498 ,-0.403) |
| Case Count Lag 2 | -0.784 | (-0.832 ,-0.738) |
| Absolute Humidity Lag 1 | -34.657 | (-91.229 ,-4.382) |
| Absolute Humidity Lag 2 | -5.941 | (-36.225 ,47.291) |
| Total Precipitation Lag 1 | 5845.769 | (-51006.674 ,64350.097) |
| Total Precipitation Lag 2 | -15842.463 | (-72983.37 ,39476.121) |
| Relative Humidity Lag 1 | -60.907 | (-295.61 ,152.646) |
| Relative Humidity Lag 2 | 29.971 | (-167.778 ,241.896) |
| Temperature Lag 1 | -306.38 | (-349.971 ,-223.278) |
| Temperature Lag 2 | -317.995 | (-395.205 ,-274.747) |

Table 93: Associations with monthly URTI case counts per 100,000 in Chiang Mai

| | Posterior Mean | 95% CrI |
|---|---|---|
| Case Count Lag 1 | -0.275 | (-0.322 ,-0.228) |
| Case Count Lag 2 | -0.606 | (-0.653 ,-0.561) |
| Absolute Humidity Lag 1 | 46.207 | (-30.835 ,164.085) |
| Absolute Humidity Lag 2 | -9.811 | (-84.57 ,40.925) |
| Total Precipitation Lag 1 | 577626.293 | (351701.699 ,811247.143) |
| Total Precipitation Lag 2 | -313553.71 | (-588448.889 ,-33850.818) |
| Relative Humidity Lag 1 | -298.132 | (-972.819 ,379.042) |
| Relative Humidity Lag 2 | 412.027 | (-181.599 ,1038.516) |
| Temperature Lag 1 | -226.207 | (-364.221 ,-130.99) |
| Temperature Lag 2 | -102.585 | (-166.051 ,-11.873) |

Table 94: Associations with monthly URTI case counts per 100,000 in Chiang Rai

| | Posterior Mean | 95% CrI |
|---|---|---|
| Case Count Lag 1 | -0.053 | (-0.164 ,0.057) |
| Case Count Lag 2 | -0.659 | (-0.763 ,-0.558) |
| Absolute Humidity Lag 1 | 70.959 | (-9.034 ,162.918) |
| Absolute Humidity Lag 2 | -8.092 | (-60.767 ,36.583) |
| Total Precipitation Lag 1 | 415290.019 | (228751.78 ,599430.982) |
| Total Precipitation Lag 2 | -131392.839 | (-336004.344 ,76892.238) |
| Relative Humidity Lag 1 | -63.05 | (-722.252 ,583.862) |
| Relative Humidity Lag 2 | 554.595 | (1.791 ,1165.464) |
| Temperature Lag 1 | -219.906 | (-332.68 ,-121.717) |
| Temperature Lag 2 | -101.828 | (-156.813 ,-38.272) |

Table 95: Associations with monthly URTI case counts per 100,000 in Chon Buri

| | Posterior Mean | 95% CrI |
|---|---|---|
| Case Count Lag 1 | -0.281 | (-0.35 ,-0.213) |
| Case Count Lag 2 | -0.667 | (-0.734 ,-0.6) |
| Absolute Humidity Lag 1 | -46.359 | (-137.049 ,-1.964) |
| Absolute Humidity Lag 2 | 2.674 | (-42.69 ,76.814) |
| Total Precipitation Lag 1 | -36650.56 | (-159341.521 ,82448.655) |
| Total Precipitation Lag 2 | -106611.332 | (-223458.888 ,9056.47) |
| Relative Humidity Lag 1 | -68.51 | (-448.765 ,289.452) |
| Relative Humidity Lag 2 | -14.495 | (-339.131 ,330.089) |
| Temperature Lag 1 | -262.248 | (-329.547 ,-127.45) |
| Temperature Lag 2 | -281.202 | (-393.929 ,-212.645) |

Table 96: Associations with monthly URTI case counts per 100,000 in Chumphon

| | Posterior Mean | 95% CrI |
|---|---|---|
| Case Count Lag 1 | -0.17 | (-0.256 ,-0.088) |
| Case Count Lag 2 | -0.561 | (-0.643 ,-0.483) |
| Absolute Humidity Lag 1 | -19.988 | (-50.301 ,31.768) |
| Absolute Humidity Lag 2 | -10.918 | (-41.72 ,35.818) |
| Total Precipitation Lag 1 | -52931.631 | (-104759.458 ,-5730.976) |
| Total Precipitation Lag 2 | 3689.788 | (-41454.822 ,49877.731) |
| Relative Humidity Lag 1 | 140.034 | (-145.036 ,460.687) |
| Relative Humidity Lag 2 | -19.524 | (-258.647 ,230.049) |
| Temperature Lag 1 | -385.836 | (-464.347 ,-340.642) |
| Temperature Lag 2 | -365.778 | (-435.47 ,-318.795) |

Table 97: Associations with monthly URTI case counts per 100,000 in Kalasin

| | Posterior Mean | 95% CrI |
|---|---|---|
| Case Count Lag 1 | 0.292 | (0.143 ,0.438) |
| Case Count Lag 2 | -0.397 | (-0.544 ,-0.248) |
| Absolute Humidity Lag 1 | -10.091 | (-23.017 ,3.042) |
| Absolute Humidity Lag 2 | -7.302 | (-20.284 ,5.444) |
| Total Precipitation Lag 1 | 99771.264 | (29103.493 ,173096.63) |
| Total Precipitation Lag 2 | -53094.201 | (-133301.331 ,21395.038) |
| Relative Humidity Lag 1 | 32.638 | (-159.607 ,224.798) |
| Relative Humidity Lag 2 | -51.872 | (-223.942 ,120.357) |
| Temperature Lag 1 | -130.441 | (-149.293 ,-112.896) |
| Temperature Lag 2 | -124.984 | (-142.671 ,-106.646) |

Table 98: Associations with monthly URTI case counts per 100,000 in Kamphaeng Phet

| | Posterior Mean | 95% CrI |
|---|---|---|
| Case Count Lag 1 | -0.14 | (-0.231 ,-0.049) |
| Case Count Lag 2 | -0.621 | (-0.708 ,-0.53) |
| Absolute Humidity Lag 1 | -7.912 | (-29.516 ,20.824) |
| Absolute Humidity Lag 2 | -3.47 | (-21.711 ,23.099) |
| Total Precipitation Lag 1 | 95479.947 | (-4479.751 ,202519.848) |
| Total Precipitation Lag 2 | -83049.297 | (-195649.795 ,25578.093) |
| Relative Humidity Lag 1 | -22.074 | (-351.191 ,288.791) |
| Relative Humidity Lag 2 | -6.423 | (-326.489 ,328.095) |
| Temperature Lag 1 | -153.922 | (-194.606 ,-124.159) |
| Temperature Lag 2 | -142.471 | (-180.118 ,-116.704) |

Table 99: Associations with monthly URTI case counts per 100,000 in Kanchanaburi

| | Posterior Mean | 95% CrI |
|---|---|---|
| Case Count Lag 1 | -0.382 | (-0.455 ,-0.31) |
| Case Count Lag 2 | -0.753 | (-0.821 ,-0.685) |
| Absolute Humidity Lag 1 | -19.755 | (-53.413 ,13.606) |
| Absolute Humidity Lag 2 | -17.056 | (-60.151 ,8.914) |
| Total Precipitation Lag 1 | 142337.126 | (39827.17 ,247770.146) |
| Total Precipitation Lag 2 | -87898.458 | (-192929.928 ,18511.062) |
| Relative Humidity Lag 1 | -18.465 | (-332.248 ,320.241) |
| Relative Humidity Lag 2 | -54.219 | (-400.828 ,258.527) |
| Temperature Lag 1 | -210.624 | (-255.819 ,-164.443) |
| Temperature Lag 2 | -187.351 | (-223.386 ,-128.403) |

Table 100: Associations with monthly URTI case counts per 100,000 in Khon Kaen

| | Posterior Mean | 95% CrI |
|---|---|---|
| Case Count Lag 1 | 0.544 | (0.371 ,0.721) |
| Case Count Lag 2 | -0.518 | (-0.689 ,-0.345) |
| Absolute Humidity Lag 1 | -12.779 | (-67.23 ,51.493) |
| Absolute Humidity Lag 2 | 6.34 | (-39.342 ,75.004) |
| Total Precipitation Lag 1 | -49695.511 | (-389917.41 ,275935.211) |
| Total Precipitation Lag 2 | -129427.904 | (-458077.762 ,180154.06) |
| Relative Humidity Lag 1 | 304.011 | (-464.598 ,1120.093) |
| Relative Humidity Lag 2 | -225.038 | (-1011.347 ,547.643) |
| Temperature Lag 1 | -155.049 | (-244.843 ,-76.189) |
| Temperature Lag 2 | -140.556 | (-239.596 ,-75.807) |

Table 101: Associations with monthly URTI case counts per 100,000 in Krabi

| | Posterior Mean | 95% CrI |
|---|---|---|
| Case Count Lag 1 | 0.227 | (0.054 ,0.393) |
| Case Count Lag 2 | -0.991 | (-1.159 ,-0.824) |
| Absolute Humidity Lag 1 | -27.856 | (-76.08 ,11.444) |
| Absolute Humidity Lag 2 | -1.583 | (-38.905 ,54.884) |
| Total Precipitation Lag 1 | -53322.183 | (-111541.966 ,3037.364) |
| Total Precipitation Lag 2 | -39332.416 | (-95476.672 ,13889.727) |
| Relative Humidity Lag 1 | -170.342 | (-492.41 ,106.78) |
| Relative Humidity Lag 2 | 529.477 | (240.753 ,821.633) |
| Temperature Lag 1 | -385.024 | (-447.451 ,-311.24) |
| Temperature Lag 2 | -375.73 | (-458.987 ,-317.683) |

Table 102: Associations with monthly URTI case counts per 100,000 in Lampang

| | Posterior Mean | 95% CrI |
|---|---|---|
| Case Count Lag 1 | -0.397 | (-0.449 ,-0.343) |
| Case Count Lag 2 | -0.732 | (-0.783 ,-0.68) |
| Absolute Humidity Lag 1 | -9.158 | (-31.11 ,14.85) |
| Absolute Humidity Lag 2 | -5.759 | (-24.453 ,16.298) |
| Total Precipitation Lag 1 | 194133.059 | (83874.226 ,302970.682) |
| Total Precipitation Lag 2 | -106908.545 | (-227582.208 ,5913.408) |
| Relative Humidity Lag 1 | -239.632 | (-537.457 ,44.278) |
| Relative Humidity Lag 2 | 221.552 | (-22.989 ,475.359) |
| Temperature Lag 1 | -134.701 | (-166.78 ,-107.001) |
| Temperature Lag 2 | -123.675 | (-152.746 ,-98.313) |

Table 103: Associations with monthly URTI case counts per 100,000 in Lamphun

| | Posterior Mean | 95% CrI |
|---|---|---|
| Case Count Lag 1 | -0.248 | (-0.297 ,-0.2) |
| Case Count Lag 2 | -0.562 | (-0.61 ,-0.516) |
| Absolute Humidity Lag 1 | -6.204 | (-12.873 ,5.186) |
| Absolute Humidity Lag 2 | -9.624 | (-20.879 ,-3.341) |
| Total Precipitation Lag 1 | 28823.534 | (-13960.749 ,70110.291) |
| Total Precipitation Lag 2 | -59836.323 | (-108622.281 ,-13032.989) |
| Relative Humidity Lag 1 | 11.266 | (-87.712 ,120.359) |
| Relative Humidity Lag 2 | 26.525 | (-73.437 ,122.038) |
| Temperature Lag 1 | -126.585 | (-141.67 ,-117.857) |
| Temperature Lag 2 | -118.868 | (-127.141 ,-103.343) |

Table 104: Associations with monthly URTI case counts per 100,000 in Loei

| | Posterior Mean | 95% CrI |
|---|---|---|
| Case Count Lag 1 | 0.689 | (0.427 ,0.957) |
| Case Count Lag 2 | -0.428 | (-0.693 ,-0.161) |
| Absolute Humidity Lag 1 | -8.846 | (-28.767 ,14.352) |
| Absolute Humidity Lag 2 | -4.049 | (-19.5 ,20.496) |
| Total Precipitation Lag 1 | 67190.343 | (-35250.089 ,171773.347) |
| Total Precipitation Lag 2 | -53204.155 | (-160688.366 ,45540.807) |
| Relative Humidity Lag 1 | 19.645 | (-265.4 ,316.949) |
| Relative Humidity Lag 2 | -69.461 | (-346.924 ,215.29) |
| Temperature Lag 1 | -136.118 | (-165.92 ,-111.791) |
| Temperature Lag 2 | -126.815 | (-157.557 ,-106.558) |

Table 105: Associations with monthly URTI case counts per 100,000 in Lop Buri

| | Posterior Mean | 95% CrI |
|---|---|---|
| Case Count Lag 1 | -0.098 | (-0.198 ,-0.002) |
| Case Count Lag 2 | -0.66 | (-0.751 ,-0.564) |
| Absolute Humidity Lag 1 | -14.866 | (-38.283 ,10.637) |
| Absolute Humidity Lag 2 | -7.358 | (-29.779 ,18.013) |
| Total Precipitation Lag 1 | 8966.165 | (-103436.764 ,121085.156) |
| Total Precipitation Lag 2 | -95894.433 | (-210788.87 ,22513.676) |
| Relative Humidity Lag 1 | 153.287 | (-100.083 ,428.864) |
| Relative Humidity Lag 2 | -180.449 | (-450.828 ,74.045) |
| Temperature Lag 1 | -186.556 | (-226.437 ,-149.623) |
| Temperature Lag 2 | -175.011 | (-214.738 ,-139.987) |

Table 106: Associations with monthly URTI case counts per 100,000 in Mae Hong Son

| | Posterior Mean | 95% CrI |
|---|---|---|
| Case Count Lag 1 | 0.137 | (-0.057 ,0.339) |
| Case Count Lag 2 | -0.488 | (-0.666 ,-0.307) |
| Absolute Humidity Lag 1 | -6.205 | (-20.114 ,16.389) |
| Absolute Humidity Lag 2 | -10.142 | (-31.493 ,2.155) |
| Total Precipitation Lag 1 | 168985.179 | (102873.42 ,233644.015) |
| Total Precipitation Lag 2 | 36248.279 | (-41008.021 ,121456.863) |
| Relative Humidity Lag 1 | -146.881 | (-311.563 ,23.393) |
| Relative Humidity Lag 2 | -56.108 | (-228.712 ,93.561) |
| Temperature Lag 1 | -138.633 | (-165.85 ,-122.542) |
| Temperature Lag 2 | -122.797 | (-138.178 ,-97.056) |

Table 107: Associations with monthly URTI case counts per 100,000 in Maha Sarakham

| | Posterior Mean | 95% CrI |
|---|---|---|
| Case Count Lag 1 | 1.511 | (1.192 ,1.836) |
| Case Count Lag 2 | -0.609 | (-0.945 ,-0.306) |
| Absolute Humidity Lag 1 | -12.585 | (-47.95 ,26.632) |
| Absolute Humidity Lag 2 | -3.119 | (-35.201 ,36.236) |
| Total Precipitation Lag 1 | 119280.883 | (-77151.691 ,321445.385) |
| Total Precipitation Lag 2 | -174273.326 | (-379000.983 ,23834.48) |
| Relative Humidity Lag 1 | 128.392 | (-370.492 ,642.113) |
| Relative Humidity Lag 2 | -27.864 | (-508.88 ,447.527) |
| Temperature Lag 1 | -142.902 | (-198.352 ,-92.601) |
| Temperature Lag 2 | -131.493 | (-189.471 ,-85.749) |

Table 108: Associations with monthly URTI case counts per 100,000 in Mukdahan

| | Posterior Mean | 95% CrI |
|---|---|---|
| Case Count Lag 1 | 0.098 | (-0.021 ,0.212) |
| Case Count Lag 2 | -0.405 | (-0.517 ,-0.284) |
| Absolute Humidity Lag 1 | -7.755 | (-18.221 ,7.745) |
| Absolute Humidity Lag 2 | -6.795 | (-18.718 ,5.544) |
| Total Precipitation Lag 1 | 78504.693 | (18371.131 ,137559.051) |
| Total Precipitation Lag 2 | -1601.835 | (-61751.777 ,57503.152) |
| Relative Humidity Lag 1 | -1.418 | (-181.226 ,179.826) |
| Relative Humidity Lag 2 | -94.878 | (-262.988 ,65.014) |
| Temperature Lag 1 | -128.727 | (-149.385 ,-114.854) |
| Temperature Lag 2 | -120.92 | (-137.031 ,-104.825) |

Table 109: Associations with monthly URTI case counts per 100,000 in Nakhon Nayok

| | Posterior Mean | 95% CrI |
|---|---|---|
| Case Count Lag 1 | 0.238 | (0.075 ,0.394) |
| Case Count Lag 2 | -0.411 | (-0.561 ,-0.255) |
| Absolute Humidity Lag 1 | -15.854 | (-26.455 ,-6.107) |
| Absolute Humidity Lag 2 | -13.766 | (-24.961 ,-4.828) |
| Total Precipitation Lag 1 | -8540.322 | (-36352.086 ,18120.268) |
| Total Precipitation Lag 2 | -22440.339 | (-49161.651 ,4560.255) |
| Relative Humidity Lag 1 | 112.331 | (9.284 ,220.333) |
| Relative Humidity Lag 2 | -28.626 | (-127.098 ,65.818) |
| Temperature Lag 1 | -221.921 | (-236.172 ,-206.981) |
| Temperature Lag 2 | -215.769 | (-228.871 ,-199.464) |

Table 110: Associations with monthly URTI case counts per 100,000 in Nakhon Pathom

| | Posterior Mean | 95% CrI |
|---|---|---|
| Case Count Lag 1 | -0.346 | (-0.382 ,-0.312) |
| Case Count Lag 2 | -0.634 | (-0.67 ,-0.599) |
| Absolute Humidity Lag 1 | -24.706 | (-57.927 ,-2.764) |
| Absolute Humidity Lag 2 | -6.085 | (-30.363 ,18.725) |
| Total Precipitation Lag 1 | -137965.215 | (-244924.984 ,-23319.01) |
| Total Precipitation Lag 2 | -3431.51 | (-101500.791 ,94418.98) |
| Relative Humidity Lag 1 | 45.106 | (-188.738 ,267.907) |
| Relative Humidity Lag 2 | -75.043 | (-283.106 ,131.485) |
| Temperature Lag 1 | -195.203 | (-230.388 ,-140.476) |
| Temperature Lag 2 | -190.388 | (-229.831 ,-151.276) |

Table 111: Associations with monthly URTI case counts per 100,000 in Nakhon Phanom

| | Posterior Mean | 95% CrI |
|---|---|---|
| Case Count Lag 1 | -0.143 | (-0.262 ,-0.022) |
| Case Count Lag 2 | -0.794 | (-0.909 ,-0.687) |
| Absolute Humidity Lag 1 | -6.771 | (-21.848 ,15.203) |
| Absolute Humidity Lag 2 | -6.969 | (-22.95 ,9.693) |
| Total Precipitation Lag 1 | 135009.686 | (61464.536 ,207538.56) |
| Total Precipitation Lag 2 | -6409.609 | (-83531.032 ,66817.331) |
| Relative Humidity Lag 1 | 68.56 | (-173.071 ,327.504) |
| Relative Humidity Lag 2 | -184.503 | (-419.319 ,43.26) |
| Temperature Lag 1 | -124.861 | (-154.44 ,-104.942) |
| Temperature Lag 2 | -115.725 | (-137.427 ,-95.46) |

Table 112: Associations with monthly URTI case counts per 100,000 in Nakhon Ratchasima

| | Posterior Mean | 95% CrI |
|---|---|---|
| Case Count Lag 1 | -0.361 | (-0.388 ,-0.333) |
| Case Count Lag 2 | -0.548 | (-0.574 ,-0.521) |
| Absolute Humidity Lag 1 | -29.893 | (-87.68 ,17.072) |
| Absolute Humidity Lag 2 | 3.535 | (-38.754 ,57.2) |
| Total Precipitation Lag 1 | -33637.04 | (-268878.078 ,194795.01) |
| Total Precipitation Lag 2 | -467114.803 | (-698707.988 ,-230839.238) |
| Relative Humidity Lag 1 | 383.368 | (-200.314 ,972.315) |
| Relative Humidity Lag 2 | 308.373 | (-278.956 ,901.213) |
| Temperature Lag 1 | -166.122 | (-232.974 ,-84.717) |
| Temperature Lag 2 | -155.482 | (-233.435 ,-94.758) |

Table 113: Associations with monthly URTI case counts per 100,000 in Nakhon Sawan

| | Posterior Mean | 95% CrI |
|---|---|---|
| Case Count Lag 1 | -0.332 | (-0.37 ,-0.294) |
| Case Count Lag 2 | -0.606 | (-0.645 ,-0.568) |
| Absolute Humidity Lag 1 | -17.272 | (-40.346 ,-0.86) |
| Absolute Humidity Lag 2 | -8.46 | (-26.151 ,8.875) |
| Total Precipitation Lag 1 | 26084.375 | (-62508.732 ,119289.467) |
| Total Precipitation Lag 2 | -148358.691 | (-246399.291 ,-55017.453) |
| Relative Humidity Lag 1 | -4.799 | (-240.121 ,226.867) |
| Relative Humidity Lag 2 | 109.517 | (-115.714 ,346.697) |
| Temperature Lag 1 | -148.218 | (-172.745 ,-113.21) |
| Temperature Lag 2 | -145.887 | (-171.852 ,-117.803) |

Table 114: Associations with monthly URTI case counts per 100,000 in Nakhon Si Thammarat

| | Posterior Mean | 95% CrI |
|---|---|---|
| Case Count Lag 1 | -0.443 | (-0.538 ,-0.353) |
| Case Count Lag 2 | -0.863 | (-0.948 ,-0.773) |
| Absolute Humidity Lag 1 | -21.108 | (-85.863 ,69.977) |
| Absolute Humidity Lag 2 | 23.147 | (-37.86 ,129.26) |
| Total Precipitation Lag 1 | -69368.305 | (-164525.271 ,28335.413) |
| Total Precipitation Lag 2 | 35095.794 | (-56135.631 ,126961.748) |
| Relative Humidity Lag 1 | -356.332 | (-1013.116 ,269.563) |
| Relative Humidity Lag 2 | 483.404 | (-86.403 ,1076.567) |
| Temperature Lag 1 | -402.274 | (-544.075 ,-303.829) |
| Temperature Lag 2 | -361.722 | (-520.416 ,-267.03) |

Table 115: Associations with monthly URTI case counts per 100,000 in Nan

| | Posterior Mean | 95% CrI |
|---|---|---|
| Case Count Lag 1 | 0.143 | (0.025 ,0.262) |
| Case Count Lag 2 | -0.71 | (-0.83 ,-0.591) |
| Absolute Humidity Lag 1 | -7.628 | (-21.733 ,14.716) |
| Absolute Humidity Lag 2 | -11.034 | (-31.925 ,1.009) |
| Total Precipitation Lag 1 | 83160.839 | (26591.11 ,136648.169) |
| Total Precipitation Lag 2 | -33.946 | (-65502.455 ,67983.454) |
| Relative Humidity Lag 1 | 15.564 | (-198.325 ,242.596) |
| Relative Humidity Lag 2 | -95.411 | (-296.289 ,88.171) |
| Temperature Lag 1 | -134.288 | (-161.225 ,-116.988) |
| Temperature Lag 2 | -122.763 | (-137.84 ,-97.25) |

Table 116: Associations with monthly URTI case counts per 100,000 in Narathiwat

| | Posterior Mean | 95% CrI |
|---|---|---|
| Case Count Lag 1 | 0.236 | (0.006 ,0.467) |
| Case Count Lag 2 | -1.003 | (-1.222 ,-0.785) |
| Absolute Humidity Lag 1 | -15.112 | (-54.914 ,51.595) |
| Absolute Humidity Lag 2 | 1.456 | (-45.135 ,71.43) |
| Total Precipitation Lag 1 | -27710.913 | (-90579.09 ,30034.428) |
| Total Precipitation Lag 2 | 3765.688 | (-59033.377 ,67783.362) |
| Relative Humidity Lag 1 | 123.92 | (-369.916 ,679.529) |
| Relative Humidity Lag 2 | 464.639 | (-51.736 ,998.4) |
| Temperature Lag 1 | -325.611 | (-425.573 ,-267.796) |
| Temperature Lag 2 | -309.035 | (-412.068 ,-240.313) |

Table 117: Associations with monthly URTI case counts per 100,000 in Nong Bua Lam Phu

| | Posterior Mean | 95% CrI |
|---|---|---|
| Case Count Lag 1 | 0.432 | (0.279 ,0.58) |
| Case Count Lag 2 | -0.371 | (-0.516 ,-0.211) |
| Absolute Humidity Lag 1 | -8.805 | (-17.631 ,-0.168) |
| Absolute Humidity Lag 2 | -0.371 | (-9.158 ,14.214) |
| Total Precipitation Lag 1 | -5836.278 | (-56346.456 ,43362.133) |
| Total Precipitation Lag 2 | -26273.111 | (-78058.071 ,23146.991) |
| Relative Humidity Lag 1 | 77.63 | (-51.165 ,220.974) |
| Relative Humidity Lag 2 | -39.066 | (-172.199 ,102.716) |
| Temperature Lag 1 | -118.513 | (-130.687 ,-106.986) |
| Temperature Lag 2 | -126.487 | (-146.283 ,-114.105) |

Table 118: Associations with monthly URTI case counts per 100,000 in Nong Khai

| | Posterior Mean | 95% CrI |
|---|---|---|
| Case Count Lag 1 | -0.117 | (-0.183 ,-0.053) |
| Case Count Lag 2 | -0.35 | (-0.413 ,-0.285) |
| Absolute Humidity Lag 1 | -1.548 | (-18.157 ,26.793) |
| Absolute Humidity Lag 2 | -7.604 | (-26.135 ,7.27) |
| Total Precipitation Lag 1 | 127241.355 | (58204.407 ,197145.023) |
| Total Precipitation Lag 2 | 44206.7 | (-30568.685 ,125088.472) |
| Relative Humidity Lag 1 | 29.808 | (-195.951 ,273.5) |
| Relative Humidity Lag 2 | -280.483 | (-507.198 ,-56.415) |
| Temperature Lag 1 | -141.272 | (-178.379 ,-119.129) |
| Temperature Lag 2 | -112.951 | (-132.07 ,-88.129) |

Table 119: Associations with monthly URTI case counts per 100,000 in Nonthaburi

| | Posterior Mean | 95% CrI |
|---|---|---|
| Case Count Lag 1 | -0.435 | (-0.483 ,-0.387) |
| Case Count Lag 2 | -0.732 | (-0.78 ,-0.684) |
| Absolute Humidity Lag 1 | -18.316 | (-37.341 ,-4.835) |
| Absolute Humidity Lag 2 | -4.524 | (-18.037 ,16.897) |
| Total Precipitation Lag 1 | -51042.113 | (-115315.94 ,12700.14) |
| Total Precipitation Lag 2 | -13671.397 | (-71757.511 ,41943.243) |
| Relative Humidity Lag 1 | 63.316 | (-70.16 ,208.043) |
| Relative Humidity Lag 2 | -11.548 | (-145.541 ,127.23) |
| Temperature Lag 1 | -201.981 | (-224.244 ,-171.734) |
| Temperature Lag 2 | -200.757 | (-233.715 ,-179.16) |

Table 120: Associations with monthly URTI case counts per 100,000 in Pathum Thani

| | Posterior Mean | 95% CrI |
|---|---|---|
| Case Count Lag 1 | 0.483 | (0.343 ,0.622) |
| Case Count Lag 2 | -0.814 | (-0.96 ,-0.667) |
| Absolute Humidity Lag 1 | -31.042 | (-92.1 ,1.873) |
| Absolute Humidity Lag 2 | 7.103 | (-31.09 ,55.33) |
| Total Precipitation Lag 1 | -188759.39 | (-341494.12 ,-24619.482) |
| Total Precipitation Lag 2 | 43146.273 | (-84445.38 ,185209.258) |
| Relative Humidity Lag 1 | -1.541 | (-432.534 ,408.513) |
| Relative Humidity Lag 2 | -277.898 | (-670.555 ,93.597) |
| Temperature Lag 1 | -194.432 | (-246.86 ,-95.827) |
| Temperature Lag 2 | -200.191 | (-274.963 ,-140.702) |

Table 121: Associations with monthly URTI case counts per 100,000 in Pattani

| | Posterior Mean | 95% CrI |
|---|---|---|
| Case Count Lag 1 | 1.499 | (1.128 ,1.882) |
| Case Count Lag 2 | -1.135 | (-1.504 ,-0.761) |
| Absolute Humidity Lag 1 | -18.879 | (-50.487 ,19.587) |
| Absolute Humidity Lag 2 | -2.764 | (-32.816 ,49.464) |
| Total Precipitation Lag 1 | -25069.527 | (-76189.407 ,22188.176) |
| Total Precipitation Lag 2 | -13773.645 | (-64035.593 ,35246.13) |
| Relative Humidity Lag 1 | -28.034 | (-293.359 ,233.174) |
| Relative Humidity Lag 2 | 498.404 | (231.274 ,778.88) |
| Temperature Lag 1 | -331.924 | (-391.616 ,-280.931) |
| Temperature Lag 2 | -324.216 | (-405.603 ,-276.233) |

Table 122: Associations with monthly URTI case counts per 100,000 in Phangnga

| | Posterior Mean | 95% CrI |
|---|---|---|
| Case Count Lag 1 | -0.288 | (-0.395 ,-0.188) |
| Case Count Lag 2 | -0.799 | (-0.901 ,-0.692) |
| Absolute Humidity Lag 1 | -32.497 | (-90.461 ,-8.177) |
| Absolute Humidity Lag 2 | -26.251 | (-63.537 ,0.381) |
| Total Precipitation Lag 1 | 4156.016 | (-27354.357 ,35107.56) |
| Total Precipitation Lag 2 | -47959.129 | (-80008.452 ,-16039.851) |
| Relative Humidity Lag 1 | -121.145 | (-312.594 ,60.929) |
| Relative Humidity Lag 2 | 353.66 | (178.742 ,521.806) |
| Temperature Lag 1 | -447.696 | (-483.825 ,-359.389) |
| Temperature Lag 2 | -453.396 | (-494.315 ,-395.627) |

Table 123: Associations with monthly URTI case counts per 100,000 in Phatthalung

| | Posterior Mean | 95% CrI |
|---|---|---|
| Case Count Lag 1 | -0.349 | (-0.449 ,-0.248) |
| Case Count Lag 2 | -0.762 | (-0.861 ,-0.662) |
| Absolute Humidity Lag 1 | -36.21 | (-96.632 ,-0.435) |
| Absolute Humidity Lag 2 | -11.518 | (-54.283 ,35.205) |
| Total Precipitation Lag 1 | -3092.799 | (-54628.748 ,47306.45) |
| Total Precipitation Lag 2 | 39574.16 | (-7182.274 ,89650.349) |
| Relative Humidity Lag 1 | -495.868 | (-885.74 ,-88.16) |
| Relative Humidity Lag 2 | 373.239 | (0.037 ,762.757) |
| Temperature Lag 1 | -381.133 | (-437.283 ,-287.337) |
| Temperature Lag 2 | -366.225 | (-437.693 ,-298.762) |

Table 124: Associations with monthly URTI case counts per 100,000 in Phayao

| | Posterior Mean | 95% CrI |
|---|---|---|
| Case Count Lag 1 | -0.28 | (-0.337 ,-0.226) |
| Case Count Lag 2 | -0.536 | (-0.587 ,-0.482) |
| Absolute Humidity Lag 1 | 1.793 | (-15.272 ,33.323) |
| Absolute Humidity Lag 2 | -9.111 | (-28.404 ,3.839) |
| Total Precipitation Lag 1 | 120311.995 | (51506.739 ,189405.598) |
| Total Precipitation Lag 2 | -33710.489 | (-119359.434 ,42160.343) |
| Relative Humidity Lag 1 | -64.108 | (-291.477 ,152.185) |
| Relative Humidity Lag 2 | 113.454 | (-76.075 ,322.406) |
| Temperature Lag 1 | -138.238 | (-177.748 ,-116.647) |
| Temperature Lag 2 | -113.606 | (-130.175 ,-88.438) |

Table 125: Associations with monthly URTI case counts per 100,000 in Phetchabun

| | Posterior Mean | 95% CrI |
|---|---|---|
| Case Count Lag 1 | 0.51 | (0.331 ,0.685) |
| Case Count Lag 2 | -0.58 | (-0.748 ,-0.41) |
| Absolute Humidity Lag 1 | -12.891 | (-44.649 ,22.527) |
| Absolute Humidity Lag 2 | -7.454 | (-39.897 ,25.63) |
| Total Precipitation Lag 1 | 55699.198 | (-79371.779 ,204284.77) |
| Total Precipitation Lag 2 | -228546.731 | (-386469.002 ,-74583.663) |
| Relative Humidity Lag 1 | 150.183 | (-247.804 ,562.857) |
| Relative Humidity Lag 2 | 132.345 | (-247.313 ,546.232) |
| Temperature Lag 1 | -177.469 | (-225.471 ,-134.863) |
| Temperature Lag 2 | -159.87 | (-205.607 ,-115.626) |

Table 126: Associations with monthly URTI case counts per 100,000 in Phetchaburi

| | Posterior Mean | 95% CrI |
|---|---|---|
| Case Count Lag 1 | -0.177 | (-0.276 ,-0.083) |
| Case Count Lag 2 | -0.697 | (-0.793 ,-0.602) |
| Absolute Humidity Lag 1 | -16.518 | (-35.12 ,3.942) |
| Absolute Humidity Lag 2 | -4.452 | (-20.983 ,20.412) |
| Total Precipitation Lag 1 | -40962.286 | (-113959.708 ,28183.069) |
| Total Precipitation Lag 2 | -31249.882 | (-103670.223 ,37027.945) |
| Relative Humidity Lag 1 | 21.705 | (-162.912 ,209.194) |
| Relative Humidity Lag 2 | 35.59 | (-132.936 ,225.062) |
| Temperature Lag 1 | -238.57 | (-268.767 ,-212.162) |
| Temperature Lag 2 | -228.945 | (-265.09 ,-205.257) |

Table 127: Associations with monthly URTI case counts per 100,000 in Phichit

| | Posterior Mean | 95% CrI |
|---|---|---|
| Case Count Lag 1 | -0.261 | (-0.339 ,-0.184) |
| Case Count Lag 2 | -0.517 | (-0.593 ,-0.444) |
| Absolute Humidity Lag 1 | -9.007 | (-21.225 ,7.801) |
| Absolute Humidity Lag 2 | -10.823 | (-25.965 ,-0.194) |
| Total Precipitation Lag 1 | 29725.86 | (-18326.531 ,83176.107) |
| Total Precipitation Lag 2 | -41913.252 | (-97857.803 ,10496.178) |
| Relative Humidity Lag 1 | -13.149 | (-184.033 ,159.986) |
| Relative Humidity Lag 2 | -5.416 | (-161.159 ,161.463) |
| Temperature Lag 1 | -159.485 | (-184.159 ,-142.595) |
| Temperature Lag 2 | -142.944 | (-159.311 ,-119.586) |

Table 128: Associations with monthly URTI case counts per 100,000 in Phitsanulok

| | Posterior Mean | 95% CrI |
|---|---|---|
| Case Count Lag 1 | -0.399 | (-0.438 ,-0.357) |
| Case Count Lag 2 | -0.691 | (-0.732 ,-0.653) |
| Absolute Humidity Lag 1 | -10.388 | (-29.633 ,12.078) |
| Absolute Humidity Lag 2 | -7.222 | (-24.591 ,14.253) |
| Total Precipitation Lag 1 | 55076.117 | (-16759.167 ,126703.91) |
| Total Precipitation Lag 2 | -68543.406 | (-145181.712 ,1707.139) |
| Relative Humidity Lag 1 | 59.898 | (-177.871 ,320.366) |
| Relative Humidity Lag 2 | -9.72 | (-241.868 ,239.369) |
| Temperature Lag 1 | -150.911 | (-181.416 ,-125.524) |
| Temperature Lag 2 | -144.623 | (-174.759 ,-120.495) |

Table 129: Associations with monthly URTI case counts per 100,000 in Phra Nakhon Si Ayutthaya

| | Posterior Mean | 95% CrI |
|---|---|---|
| Case Count Lag 1 | -0.125 | (-0.223 ,-0.017) |
| Case Count Lag 2 | -0.848 | (-0.958 ,-0.745) |
| Absolute Humidity Lag 1 | -26.613 | (-68.186 ,7.457) |
| Absolute Humidity Lag 2 | -2.541 | (-39.55 ,33.551) |
| Total Precipitation Lag 1 | -191548.345 | (-349003.183 ,-33620.872) |
| Total Precipitation Lag 2 | -66825.552 | (-206144.363 ,66871.419) |
| Relative Humidity Lag 1 | 83.838 | (-250.355 ,431.305) |
| Relative Humidity Lag 2 | -106.57 | (-417.104 ,203.185) |
| Temperature Lag 1 | -210.056 | (-266.197 ,-143.074) |
| Temperature Lag 2 | -188.246 | (-244.612 ,-127.7) |

Table 130: Associations with monthly URTI case counts per 100,000 in Phrae

| | Posterior Mean | 95% CrI |
|---|---|---|
| Case Count Lag 1 | 0.244 | (0.13 ,0.358) |
| Case Count Lag 2 | -0.324 | (-0.44 ,-0.21) |
| Absolute Humidity Lag 1 | -3.27 | (-14.156 ,14.764) |
| Absolute Humidity Lag 2 | -11.383 | (-27.532 ,-1.702) |
| Total Precipitation Lag 1 | 70405.189 | (25585.703 ,115584.363) |
| Total Precipitation Lag 2 | -69542.036 | (-122160.256 ,-18509.172) |
| Relative Humidity Lag 1 | -62.49 | (-214.34 ,81.931) |
| Relative Humidity Lag 2 | 119.929 | (-22.418 ,260.23) |
| Temperature Lag 1 | -143.776 | (-168.048 ,-129.317) |
| Temperature Lag 2 | -123.386 | (-136.155 ,-101.216) |

Table 131: Associations with monthly URTI case counts per 100,000 in Phuket

| | Posterior Mean | 95% CrI |
|---|---|---|
| Case Count Lag 1 | -0.254 | (-0.337 ,-0.178) |
| Case Count Lag 2 | -0.72 | (-0.793 ,-0.643) |
| Absolute Humidity Lag 1 | -27.47 | (-70.015 ,31.557) |
| Absolute Humidity Lag 2 | -21.047 | (-64.696 ,35.357) |
| Total Precipitation Lag 1 | 37154.929 | (-8243.798 ,82116.376) |
| Total Precipitation Lag 2 | -17341.323 | (-61129.148 ,27018.719) |
| Relative Humidity Lag 1 | -383.758 | (-703.219 ,-59.419) |
| Relative Humidity Lag 2 | 488.699 | (204.553 ,768.029) |
| Temperature Lag 1 | -529.423 | (-619.834 ,-464.213) |
| Temperature Lag 2 | -524.816 | (-613.386 ,-458.047) |

Table 132: Associations with monthly URTI case counts per 100,000 in Prachin Buri

| | Posterior Mean | 95% CrI |
|---|---|---|
| Case Count Lag 1 | 0.513 | (0.327 ,0.698) |
| Case Count Lag 2 | -0.841 | (-1.035 ,-0.647) |
| Absolute Humidity Lag 1 | -14.722 | (-53.31 ,40.705) |
| Absolute Humidity Lag 2 | -8.035 | (-52.047 ,36.345) |
| Total Precipitation Lag 1 | -25875.104 | (-163822.826 ,110415.775) |
| Total Precipitation Lag 2 | -123775.401 | (-264848.879 ,16726.227) |
| Relative Humidity Lag 1 | 366.704 | (-58.769 ,812.478) |
| Relative Humidity Lag 2 | -31.368 | (-450.117 ,365.79) |
| Temperature Lag 1 | -242.123 | (-322.754 ,-186.959) |
| Temperature Lag 2 | -214.188 | (-280.324 ,-150.106) |

Table 133: Associations with monthly URTI case counts per 100,000 in Prachuap Khiri Khan

| | Posterior Mean | 95% CrI |
|---|---|---|
| Case Count Lag 1 | -0.064 | (-0.152 ,0.023) |
| Case Count Lag 2 | -0.598 | (-0.683 ,-0.506) |
| Absolute Humidity Lag 1 | -22.294 | (-50.402 ,5.423) |
| Absolute Humidity Lag 2 | -6.343 | (-31.669 ,25.739) |
| Total Precipitation Lag 1 | -71756.795 | (-147676.757 ,6519.88) |
| Total Precipitation Lag 2 | 15951.089 | (-53193.909 ,87891.099) |
| Relative Humidity Lag 1 | 6.963 | (-229.793 ,230.38) |
| Relative Humidity Lag 2 | -35.459 | (-229.762 ,158.509) |
| Temperature Lag 1 | -298.427 | (-339.816 ,-256.435) |
| Temperature Lag 2 | -283.414 | (-330.206 ,-245.291) |

Table 134: Associations with monthly URTI case counts per 100,000 in Ranong

| | Posterior Mean | 95% CrI |
|---|---|---|
| Case Count Lag 1 | 0.624 | (0.374 ,0.869) |
| Case Count Lag 2 | -0.413 | (-0.658 ,-0.161) |
| Absolute Humidity Lag 1 | -24.322 | (-40.333 ,-11.529) |
| Absolute Humidity Lag 2 | -21.266 | (-38.331 ,-8.643) |
| Total Precipitation Lag 1 | -2132.142 | (-21018.731 ,16839.684) |
| Total Precipitation Lag 2 | -2130.968 | (-21170.344 ,15333.649) |
| Relative Humidity Lag 1 | -48.167 | (-166.464 ,55.524) |
| Relative Humidity Lag 2 | 11.405 | (-75.427 ,110.643) |
| Temperature Lag 1 | -392.659 | (-412.291 ,-368.822) |
| Temperature Lag 2 | -384.147 | (-402.228 ,-358.054) |

Table 135: Associations with monthly URTI case counts per 100,000 in Ratchaburi

| | Posterior Mean | 95% CrI |
|---|---|---|
| Case Count Lag 1 | -0.484 | (-0.548 ,-0.419) |
| Case Count Lag 2 | -0.887 | (-0.949 ,-0.827) |
| Absolute Humidity Lag 1 | -36.132 | (-72.842 ,-11.117) |
| Absolute Humidity Lag 2 | -17.101 | (-50.836 ,2.878) |
| Total Precipitation Lag 1 | -68634.942 | (-172334.463 ,30329.436) |
| Total Precipitation Lag 2 | -68468.12 | (-166789.739 ,27097.491) |
| Relative Humidity Lag 1 | 4.008 | (-222.106 ,230.929) |
| Relative Humidity Lag 2 | -43.683 | (-266.237 ,169.405) |
| Temperature Lag 1 | -178.862 | (-215 ,-123.781) |
| Temperature Lag 2 | -181.837 | (-211.201 ,-132.609) |

Table 136: Associations with monthly URTI case counts per 100,000 in Rayong

| | Posterior Mean | 95% CrI |
|---|---|---|
| Case Count Lag 1 | -0.336 | (-0.375 ,-0.299) |
| Case Count Lag 2 | -0.689 | (-0.727 ,-0.651) |
| Absolute Humidity Lag 1 | -27.998 | (-82.392 ,19.076) |
| Absolute Humidity Lag 2 | 36.579 | (-16.417 ,131.409) |
| Total Precipitation Lag 1 | -99501.908 | (-181800.321 ,-16450.321) |
| Total Precipitation Lag 2 | 46265.426 | (-26307.443 ,121207.909) |
| Relative Humidity Lag 1 | 42.95 | (-226.413 ,330.374) |
| Relative Humidity Lag 2 | -166.482 | (-429.651 ,105.251) |
| Temperature Lag 1 | -317.02 | (-387.802 ,-239.681) |
| Temperature Lag 2 | -355.756 | (-495.64 ,-277.934) |

Table 137: Associations with monthly URTI case counts per 100,000 in Roi Et

| | Posterior Mean | 95% CrI |
|---|---|---|
| Case Count Lag 1 | 1.593 | (1.29 ,1.887) |
| Case Count Lag 2 | -0.459 | (-0.745 ,-0.148) |
| Absolute Humidity Lag 1 | -15.308 | (-53.15 ,19.954) |
| Absolute Humidity Lag 2 | 7.247 | (-21.741 ,58.809) |
| Total Precipitation Lag 1 | 191521.484 | (24700.213 ,356604.326) |
| Total Precipitation Lag 2 | -213268.189 | (-383243.725 ,-44010.178) |
| Relative Humidity Lag 1 | 25.102 | (-434.956 ,486.1) |
| Relative Humidity Lag 2 | -12.691 | (-458.498 ,459.933) |
| Temperature Lag 1 | -142.856 | (-194.226 ,-91.05) |
| Temperature Lag 2 | -142.499 | (-214.566 ,-100.201) |

Table 138: Associations with monthly URTI case counts per 100,000 in Sa Kaeo

| | Posterior Mean | 95% CrI |
|---|---|---|
| Case Count Lag 1 | -0.04 | (-0.16 ,0.081) |
| Case Count Lag 2 | -0.554 | (-0.674 ,-0.434) |
| Absolute Humidity Lag 1 | -16.207 | (-26.947 ,-8.865) |
| Absolute Humidity Lag 2 | -13.257 | (-23.872 ,-5.907) |
| Total Precipitation Lag 1 | -22324.329 | (-57507.711 ,10553.32) |
| Total Precipitation Lag 2 | -46067.21 | (-81652.002 ,-11061.459) |
| Relative Humidity Lag 1 | 76.46 | (-9.701 ,167.644) |
| Relative Humidity Lag 2 | -12.886 | (-91.423 ,60.152) |
| Temperature Lag 1 | -213.432 | (-224.594 ,-197.946) |
| Temperature Lag 2 | -208.022 | (-218.86 ,-192.275) |

Table 139: Associations with monthly URTI case counts per 100,000 in Sakon Nakhon

| | Posterior Mean | 95% CrI |
|---|---|---|
| Case Count Lag 1 | 0.886 | (0.654 ,1.128) |
| Case Count Lag 2 | -0.886 | (-1.127 ,-0.661) |
| Absolute Humidity Lag 1 | -12.632 | (-28.218 ,0.844) |
| Absolute Humidity Lag 2 | -6.271 | (-20.814 ,6.639) |
| Total Precipitation Lag 1 | 143040.48 | (75912.276 ,211461.835) |
| Total Precipitation Lag 2 | -47322.188 | (-122321.849 ,24091.851) |
| Relative Humidity Lag 1 | -133.431 | (-349.37 ,63.404) |
| Relative Humidity Lag 2 | -55.326 | (-248.039 ,135.082) |
| Temperature Lag 1 | -120.233 | (-138.965 ,-99.616) |
| Temperature Lag 2 | -115.043 | (-132.294 ,-95.664) |

Table 140: Associations with monthly URTI case counts per 100,000 in Samut Prakan

| | Posterior Mean | 95% CrI |
|---|---|---|
| Case Count Lag 1 | 0.181 | (0.095 ,0.267) |
| Case Count Lag 2 | -0.988 | (-1.074 ,-0.898) |
| Absolute Humidity Lag 1 | -4.482 | (-47.673 ,71.223) |
| Absolute Humidity Lag 2 | 11.648 | (-33.509 ,104.122) |
| Total Precipitation Lag 1 | -55848.744 | (-248002.372 ,129180.446) |
| Total Precipitation Lag 2 | 17509.427 | (-150097.379 ,178193.573) |
| Relative Humidity Lag 1 | 12.748 | (-469.489 ,509.473) |
| Relative Humidity Lag 2 | 170.134 | (-264.252 ,648.393) |
| Temperature Lag 1 | -249.338 | (-367.511 ,-182.258) |
| Temperature Lag 2 | -251.614 | (-396.219 ,-181.02) |

Table 141: Associations with monthly URTI case counts per 100,000 in Samut Sakhon

| | Posterior Mean | 95% CrI |
|---|---|---|
| Case Count Lag 1 | -0.187 | (-0.22 ,-0.155) |
| Case Count Lag 2 | -0.375 | (-0.407 ,-0.342) |
| Absolute Humidity Lag 1 | -17.077 | (-31.771 ,-6.892) |
| Absolute Humidity Lag 2 | -9.026 | (-19.331 ,8.46) |
| Total Precipitation Lag 1 | -64717.893 | (-113016.433 ,-17680.893) |
| Total Precipitation Lag 2 | 17273.529 | (-22752.039 ,60972.233) |
| Relative Humidity Lag 1 | 24.749 | (-88.946 ,142.742) |
| Relative Humidity Lag 2 | -81.742 | (-190.679 ,23.523) |
| Temperature Lag 1 | -218.632 | (-234.103 ,-195.248) |
| Temperature Lag 2 | -218.508 | (-245.874 ,-202.522) |

Table 142: Associations with monthly URTI case counts per 100,000 in Samut Songkhram

| | Posterior Mean | 95% CrI |
|---|---|---|
| Case Count Lag 1 | -0.532 | (-0.637 ,-0.427) |
| Case Count Lag 2 | -0.742 | (-0.855 ,-0.632) |
| Absolute Humidity Lag 1 | -14.925 | (-21.818 ,-9.504) |
| Absolute Humidity Lag 2 | -12.865 | (-20.695 ,-7.834) |
| Total Precipitation Lag 1 | -4941.606 | (-29750.846 ,20442.204) |
| Total Precipitation Lag 2 | -1300.065 | (-25519.926 ,23632.73) |
| Relative Humidity Lag 1 | -44.227 | (-105.102 ,15.509) |
| Relative Humidity Lag 2 | -18.313 | (-75.41 ,39.008) |
| Temperature Lag 1 | -210.308 | (-218.831 ,-199.745) |
| Temperature Lag 2 | -205.454 | (-213.064 ,-193.411) |

Table 143: Associations with monthly URTI case counts per 100,000 in Saraburi

| | Posterior Mean | 95% CrI |
|---|---|---|
| Case Count Lag 1 | 0.644 | (0.452 ,0.838) |
| Case Count Lag 2 | -0.954 | (-1.153 ,-0.756) |
| Absolute Humidity Lag 1 | -26.205 | (-68.963 ,-4.052) |
| Absolute Humidity Lag 2 | -7.707 | (-33.839 ,22.044) |
| Total Precipitation Lag 1 | -7138.151 | (-114986.302 ,98441.183) |
| Total Precipitation Lag 2 | -150395.956 | (-258882.288 ,-45968.902) |
| Relative Humidity Lag 1 | 1.092 | (-276.396 ,276.586) |
| Relative Humidity Lag 2 | 51.247 | (-201.924 ,321.988) |
| Temperature Lag 1 | -190.952 | (-225.772 ,-125.221) |
| Temperature Lag 2 | -197.47 | (-243.009 ,-158.197) |

Table 144: Associations with monthly URTI case counts per 100,000 in Satun

| | Posterior Mean | 95% CrI |
|---|---|---|
| Case Count Lag 1 | 0.54 | (0.275 ,0.813) |
| Case Count Lag 2 | -1.229 | (-1.508 ,-0.962) |
| Absolute Humidity Lag 1 | -26.127 | (-52.153 ,-0.861) |
| Absolute Humidity Lag 2 | -25.249 | (-52.699 ,-2.465) |
| Total Precipitation Lag 1 | 8080.452 | (-19130.683 ,38616.165) |
| Total Precipitation Lag 2 | -17747.695 | (-47752.237 ,10008.985) |
| Relative Humidity Lag 1 | -119.953 | (-299.628 ,46.649) |
| Relative Humidity Lag 2 | 258.031 | (99.855 ,410.445) |
| Temperature Lag 1 | -459.012 | (-496.598 ,-418.328) |
| Temperature Lag 2 | -454.858 | (-489.201 ,-412.232) |

Table 145: Associations with monthly URTI case counts per 100,000 in Si Sa Ket

| | Posterior Mean | 95% CrI |
|---|---|---|
| Case Count Lag 1 | 1.795 | (1.413 ,2.175) |
| Case Count Lag 2 | -0.62 | (-0.992 ,-0.227) |
| Absolute Humidity Lag 1 | -28.68 | (-97.201 ,32.206) |
| Absolute Humidity Lag 2 | 9.91 | (-42.858 ,91.798) |
| Total Precipitation Lag 1 | 75578.204 | (-167019.811 ,321533.26) |
| Total Precipitation Lag 2 | -414284.13 | (-672092.717 ,-151646.64) |
| Relative Humidity Lag 1 | 257.353 | (-495.865 ,1018.963) |
| Relative Humidity Lag 2 | 61.34 | (-604.824 ,746.591) |
| Temperature Lag 1 | -164.43 | (-251.545 ,-68.759) |
| Temperature Lag 2 | -168.082 | (-286.014 ,-93.3) |

Table 146: Associations with monthly URTI case counts per 100,000 in Sing Buri

| | Posterior Mean | 95% CrI |
|---|---|---|
| Case Count Lag 1 | 0.004 | (-0.129 ,0.133) |
| Case Count Lag 2 | -0.4 | (-0.532 ,-0.271) |
| Absolute Humidity Lag 1 | -14.962 | (-25.256 ,-9.506) |
| Absolute Humidity Lag 2 | -8.415 | (-12.984 ,0.247) |
| Total Precipitation Lag 1 | -5501.26 | (-34417.604 ,23826.322) |
| Total Precipitation Lag 2 | -19470.246 | (-48189.208 ,7467.907) |
| Relative Humidity Lag 1 | 6.914 | (-59.889 ,74.573) |
| Relative Humidity Lag 2 | -8.822 | (-64.402 ,50.868) |
| Temperature Lag 1 | -168.749 | (-177.88 ,-152.057) |
| Temperature Lag 2 | -176.339 | (-190.808 ,-168.861) |

Table 147: Associations with monthly URTI case counts per 100,000 in Songkhla

| | Posterior Mean | 95% CrI |
|---|---|---|
| Case Count Lag 1 | 0.121 | (-0.044 ,0.287) |
| Case Count Lag 2 | -0.705 | (-0.86 ,-0.545) |
| Absolute Humidity Lag 1 | -29.91 | (-133.909 ,51.961) |
| Absolute Humidity Lag 2 | 20.158 | (-58.443 ,154.252) |
| Total Precipitation Lag 1 | -91676.414 | (-213957.589 ,29489.587) |
| Total Precipitation Lag 2 | 14047.833 | (-103017.381 ,129108.12) |
| Relative Humidity Lag 1 | -30.467 | (-670.155 ,604.836) |
| Relative Humidity Lag 2 | 959.714 | (341.343 ,1598.8) |
| Temperature Lag 1 | -403.504 | (-535.203 ,-240.58) |
| Temperature Lag 2 | -400.494 | (-611.331 ,-276.609) |

Table 148: Associations with monthly URTI case counts per 100,000 in Sukhothai

| | Posterior Mean | 95% CrI |
|---|---|---|
| Case Count Lag 1 | -0.367 | (-0.41 ,-0.323) |
| Case Count Lag 2 | -0.575 | (-0.615 ,-0.534) |
| Absolute Humidity Lag 1 | -10.62 | (-20.628 ,-1.666) |
| Absolute Humidity Lag 2 | -7.917 | (-17.011 ,0.984) |
| Total Precipitation Lag 1 | 52623.846 | (-101.831 ,105617.813) |
| Total Precipitation Lag 2 | -27035.701 | (-81787.096 ,28357.569) |
| Relative Humidity Lag 1 | -20.282 | (-151.285 ,108.957) |
| Relative Humidity Lag 2 | 0.116 | (-131.646 ,128.19) |
| Temperature Lag 1 | -129.374 | (-142.732 ,-114.965) |
| Temperature Lag 2 | -127.498 | (-140.39 ,-114.302) |

Table 149: Associations with monthly URTI case counts per 100,000 in Suphan Buri

| | Posterior Mean | 95% CrI |
|---|---|---|
| Case Count Lag 1 | -0.076 | (-0.173 ,0.021) |
| Case Count Lag 2 | -0.697 | (-0.795 ,-0.603) |
| Absolute Humidity Lag 1 | -16.894 | (-37.158 ,-0.177) |
| Absolute Humidity Lag 2 | -8.801 | (-27.103 ,7.275) |
| Total Precipitation Lag 1 | -85277.364 | (-187193.504 ,15283.168) |
| Total Precipitation Lag 2 | -33565.588 | (-129232.545 ,59322.601) |
| Relative Humidity Lag 1 | 29.111 | (-173.755 ,241.833) |
| Relative Humidity Lag 2 | -63.939 | (-259.748 ,118.802) |
| Temperature Lag 1 | -174.195 | (-200.585 ,-142.388) |
| Temperature Lag 2 | -164.953 | (-189.518 ,-136.397) |

Table 150: Associations with monthly URTI case counts per 100,000 in Surat Thani

| | Posterior Mean | 95% CrI |
|---|---|---|
| Case Count Lag 1 | -0.349 | (-0.437 ,-0.26) |
| Case Count Lag 2 | -0.898 | (-0.984 ,-0.813) |
| Absolute Humidity Lag 1 | -22.847 | (-99.728 ,79.048) |
| Absolute Humidity Lag 2 | 10.831 | (-64.431 ,101.963) |
| Total Precipitation Lag 1 | -170827.046 | (-280828.361 ,-55500.75) |
| Total Precipitation Lag 2 | 30098.743 | (-75862.566 ,144967.782) |
| Relative Humidity Lag 1 | -218.275 | (-914.021 ,436.229) |
| Relative Humidity Lag 2 | 567.033 | (-64.285 ,1187.346) |
| Temperature Lag 1 | -397.628 | (-560.057 ,-280.891) |
| Temperature Lag 2 | -327.888 | (-466.674 ,-211.595) |

Table 151: Associations with monthly URTI case counts per 100,000 in Surin

| | Posterior Mean | 95% CrI |
|---|---|---|
| Case Count Lag 1 | -0.027 | (-0.127 ,0.071) |
| Case Count Lag 2 | -0.622 | (-0.721 ,-0.524) |
| Absolute Humidity Lag 1 | -19.393 | (-68.286 ,25.423) |
| Absolute Humidity Lag 2 | -8.315 | (-60.559 ,33.502) |
| Total Precipitation Lag 1 | 158775.383 | (-53119.624 ,370538.773) |
| Total Precipitation Lag 2 | -431258.426 | (-663424.112 ,-201790.444) |
| Relative Humidity Lag 1 | 314.948 | (-271.603 ,951.244) |
| Relative Humidity Lag 2 | 26.581 | (-523.754 ,571.18) |
| Temperature Lag 1 | -158.213 | (-225.688 ,-87.895) |
| Temperature Lag 2 | -138.138 | (-198.083 ,-63.292) |

Table 152: Associations with monthly URTI case counts per 100,000 in Tak

| | Posterior Mean | 95% CrI |
|---|---|---|
| Case Count Lag 1 | 0.175 | (0.022 ,0.332) |
| Case Count Lag 2 | -0.63 | (-0.783 ,-0.476) |
| Absolute Humidity Lag 1 | -9.085 | (-31.733 ,23.868) |
| Absolute Humidity Lag 2 | -13.257 | (-40.408 ,10.494) |
| Total Precipitation Lag 1 | 142642.577 | (60379.573 ,225727.337) |
| Total Precipitation Lag 2 | 44371.984 | (-46344.383 ,142384.937) |
| Relative Humidity Lag 1 | -154.08 | (-414.445 ,99.743) |
| Relative Humidity Lag 2 | -134.251 | (-408.368 ,116.426) |
| Temperature Lag 1 | -174.491 | (-215.449 ,-145.936) |
| Temperature Lag 2 | -161.809 | (-192.55 ,-127.812) |

Table 153: Associations with monthly URTI case counts per 100,000 in Trang

| | Posterior Mean | 95% CrI |
|---|---|---|
| Case Count Lag 1 | -0.404 | (-0.494 ,-0.311) |
| Case Count Lag 2 | -1.043 | (-1.135 ,-0.951) |
| Absolute Humidity Lag 1 | -40.168 | (-105.643 ,0.5) |
| Absolute Humidity Lag 2 | -1.986 | (-46.747 ,64.247) |
| Total Precipitation Lag 1 | 1758.577 | (-66431.098 ,69031.075) |
| Total Precipitation Lag 2 | -42909.296 | (-109527.539 ,20983.573) |
| Relative Humidity Lag 1 | -381.11 | (-790.842 ,-1.102) |
| Relative Humidity Lag 2 | 653.821 | (307.605 ,998.536) |
| Temperature Lag 1 | -435.724 | (-500.489 ,-333.595) |
| Temperature Lag 2 | -440 | (-543.12 ,-371.329) |

Table 154: Associations with monthly URTI case counts per 100,000 in Trat

| | Posterior Mean | 95% CrI |
|---|---|---|
| Case Count Lag 1 | -0.139 | (-0.237 ,-0.044) |
| Case Count Lag 2 | -0.647 | (-0.739 ,-0.55) |
| Absolute Humidity Lag 1 | -24.011 | (-46.592 ,-3.748) |
| Absolute Humidity Lag 2 | -19.786 | (-38.085 ,6.137) |
| Total Precipitation Lag 1 | 9348.658 | (-12543.006 ,32928.619) |
| Total Precipitation Lag 2 | 4596.852 | (-17025.726 ,26816.055) |
| Relative Humidity Lag 1 | -64.945 | (-183.195 ,36.97) |
| Relative Humidity Lag 2 | -37.183 | (-144.809 ,63.362) |
| Temperature Lag 1 | -388.387 | (-418.515 ,-356.834) |
| Temperature Lag 2 | -387.512 | (-425.663 ,-360.685) |

Table 155: Associations with monthly URTI case counts per 100,000 in Ubon Ratchathani

| | Posterior Mean | 95% CrI |
|---|---|---|
| Case Count Lag 1 | 0.092 | (-0.009 ,0.189) |
| Case Count Lag 2 | -0.705 | (-0.806 ,-0.608) |
| Absolute Humidity Lag 1 | -3.339 | (-109.228 ,149.203) |
| Absolute Humidity Lag 2 | 24.299 | (-68.628 ,185.658) |
| Total Precipitation Lag 1 | 630390.521 | (232048.367 ,1056379.105) |
| Total Precipitation Lag 2 | -577870.397 | (-1027714.203 ,-116287.496) |
| Relative Humidity Lag 1 | -589.465 | (-2069.396 ,726.567) |
| Relative Humidity Lag 2 | 697.772 | (-453.472 ,2033.107) |
| Temperature Lag 1 | -206.757 | (-420.32 ,-59.861) |
| Temperature Lag 2 | -187.012 | (-422.743 ,-57.175) |

Table 156: Associations with monthly URTI case counts per 100,000 in Udon Thani

| | Posterior Mean | 95% CrI |
|---|---|---|
| Case Count Lag 1 | 0.259 | (0.13 ,0.394) |
| Case Count Lag 2 | -0.573 | (-0.699 ,-0.443) |
| Absolute Humidity Lag 1 | -7.105 | (-31.797 ,26.229) |
| Absolute Humidity Lag 2 | -4.824 | (-30.337 ,23.327) |
| Total Precipitation Lag 1 | 258302.241 | (96197.657 ,416461.833) |
| Total Precipitation Lag 2 | -229179.177 | (-402834.955 ,-64949.083) |
| Relative Humidity Lag 1 | -38.037 | (-473.67 ,398.046) |
| Relative Humidity Lag 2 | 137.775 | (-239.43 ,549.873) |
| Temperature Lag 1 | -134.562 | (-180.451 ,-100.736) |
| Temperature Lag 2 | -116.663 | (-154.542 ,-81.33) |

Table 157: Associations with monthly URTI case counts per 100,000 in Uthai Thani

| | Posterior Mean | 95% CrI |
|---|---|---|
| Case Count Lag 1 | -0.048 | (-0.18 ,0.082) |
| Case Count Lag 2 | -0.691 | (-0.816 ,-0.564) |
| Absolute Humidity Lag 1 | -14.527 | (-28.705 ,-6.157) |
| Absolute Humidity Lag 2 | -7.937 | (-16.829 ,3.095) |
| Total Precipitation Lag 1 | -26.701 | (-44351.881 ,44853.92) |
| Total Precipitation Lag 2 | -87999.068 | (-135844.792 ,-41827.78) |
| Relative Humidity Lag 1 | 33.463 | (-89.443 ,158.537) |
| Relative Humidity Lag 2 | 39.034 | (-85.109 ,167.61) |
| Temperature Lag 1 | -148.914 | (-160.243 ,-129.768) |
| Temperature Lag 2 | -150.195 | (-164.914 ,-138.019) |

Table 158: Associations with monthly URTI case counts per 100,000 in Uttaradit

| | Posterior Mean | 95% CrI |
|---|---|---|
| Case Count Lag 1 | -0.301 | (-0.372 ,-0.228) |
| Case Count Lag 2 | -0.678 | (-0.746 ,-0.608) |
| Absolute Humidity Lag 1 | -11.702 | (-30.055 ,6.249) |
| Absolute Humidity Lag 2 | -8.476 | (-26.322 ,7.617) |
| Total Precipitation Lag 1 | 93671.33 | (14414.147 ,174273.489) |
| Total Precipitation Lag 2 | -81069.626 | (-168618.884 ,5997.486) |
| Relative Humidity Lag 1 | 52.554 | (-197.413 ,304.452) |
| Relative Humidity Lag 2 | -48.72 | (-283.142 ,175.931) |
| Temperature Lag 1 | -139.643 | (-163.393 ,-115.378) |
| Temperature Lag 2 | -132.66 | (-154.596 ,-108.554) |

Table 159: Associations with monthly URTI case counts per 100,000 in Yala

| | Posterior Mean | 95% CrI |
|---|---|---|
| Case Count Lag 1 | 3.014 | (2.481 ,3.569) |
| Case Count Lag 2 | -1.393 | (-1.944 ,-0.842) |
| Absolute Humidity Lag 1 | -26.973 | (-82.94 ,15.24) |
| Absolute Humidity Lag 2 | -19.265 | (-87.197 ,23.07) |
| Total Precipitation Lag 1 | -91368.816 | (-182358.232 ,-3239.261) |
| Total Precipitation Lag 2 | 38695.086 | (-45293.505 ,126792.131) |
| Relative Humidity Lag 1 | 39.231 | (-405.902 ,511.123) |
| Relative Humidity Lag 2 | 234.61 | (-187.387 ,645.148) |
| Temperature Lag 1 | -332.746 | (-394.212 ,-251.028) |
| Temperature Lag 2 | -293.042 | (-355.023 ,-191.967) |

Table 160: Associations with monthly URTI case counts per 100,000 in Yasothon

| | Posterior Mean | 95% CrI |
|---|---|---|
| Case Count Lag 1 | 0.618 | (0.407 ,0.831) |
| Case Count Lag 2 | -0.541 | (-0.747 ,-0.318) |
| Absolute Humidity Lag 1 | -10.737 | (-29.429 ,9.644) |
| Absolute Humidity Lag 2 | -6.131 | (-22.215 ,15.539) |
| Total Precipitation Lag 1 | 114387.699 | (32208.645 ,200962.026) |
| Total Precipitation Lag 2 | -96800.432 | (-188905.857 ,-3268.845) |
| Relative Humidity Lag 1 | 65.697 | (-179.135 ,331.292) |
| Relative Humidity Lag 2 | -68.192 | (-326.134 ,164.147) |
| Temperature Lag 1 | -139.841 | (-168.865 ,-113.834) |
| Temperature Lag 2 | -137.172 | (-167.004 ,-113.915) |

## 5.3 Coefficient output for pneumonia case counts as the dependent variable

Table 161: Associations with monthly pneumonia case counts in Amnat Charoen

| | Posterior Mean | 95% CrI |
|---|---|---|
| Case Count Lag 1 | 0.312 | (0.185 ,0.438) |
| Case Count Lag 2 | -0.768 | (-0.9 ,-0.641) |
| Absolute Humidity Lag 1 | -8.905 | (-13.38 ,-4.501) |
| Absolute Humidity Lag 2 | -7.613 | (-11.237 ,-1.648) |
| Total Precipitation Lag 1 | 9567.083 | (-8720.045 ,29177.789) |
| Total Precipitation Lag 2 | -11178.674 | (-31153.273 ,8201.542) |
| Relative Humidity Lag 1 | -16.931 | (-83.714 ,43.851) |
| Relative Humidity Lag 2 | -10.52 | (-68.135 ,50.085) |
| Temperature Lag 1 | -136.457 | (-142.642 ,-130.34) |
| Temperature Lag 2 | -136.252 | (-144.604 ,-131.179) |

Table 162: Associations with monthly pneumonia case counts in Ang Thong

| | Posterior Mean | 95% CrI |
|---|---|---|
| Case Count Lag 1 | 0.258 | (0.134 ,0.386) |
| Case Count Lag 2 | -0.813 | (-0.945 ,-0.686) |
| Absolute Humidity Lag 1 | -13.414 | (-24.635 ,-4.274) |
| Absolute Humidity Lag 2 | -10.197 | (-18.881 ,0.405) |
| Total Precipitation Lag 1 | -29233.835 | (-81837.135 ,19853.157) |
| Total Precipitation Lag 2 | -8483.024 | (-58029.459 ,38294.027) |
| Relative Humidity Lag 1 | 51.965 | (-49.78 ,160.454) |
| Relative Humidity Lag 2 | -81.187 | (-175.41 ,10.464) |
| Temperature Lag 1 | -189.708 | (-205.031 ,-171.343) |
| Temperature Lag 2 | -187.583 | (-204.97 ,-172.973) |

Table 163: Associations with monthly pneumonia case counts in Bangkok

| | Posterior Mean | 95% CrI |
|---|---|---|
| Case Count Lag 1 | 0.349 | (0.22 ,0.476) |
| Case Count Lag 2 | -0.897 | (-1.024 ,-0.768) |
| Absolute Humidity Lag 1 | -94.928 | (-802.419 ,772.615) |
| Absolute Humidity Lag 2 | 202.887 | (-492.386 ,1054.599) |
| Total Precipitation Lag 1 | -3101750.45 | (-6559615.89 ,90607.142) |
| Total Precipitation Lag 2 | -1388162.717 | (-4288530.885 ,1416902.602) |
| Relative Humidity Lag 1 | 9042.889 | (-110.911 ,18345.976) |
| Relative Humidity Lag 2 | -1012.976 | (-9075.454 ,6690.99) |
| Temperature Lag 1 | -446.44 | (-1844.589 ,663.307) |
| Temperature Lag 2 | -86.148 | (-1389.657 ,1003.856) |

Table 164: Associations with monthly pneumonia case counts in Buri Ram

| | Posterior Mean | 95% CrI |
|---|---|---|
| Case Count Lag 1 | 0.332 | (0.205 ,0.465) |
| Case Count Lag 2 | -0.901 | (-1.029 ,-0.774) |
| Absolute Humidity Lag 1 | -19.7 | (-90.747 ,35.42) |
| Absolute Humidity Lag 2 | 4.054 | (-43.331 ,79.662) |
| Total Precipitation Lag 1 | 238280.323 | (-92445.801 ,627271.134) |
| Total Precipitation Lag 2 | -58185.407 | (-411693.098 ,297172.583) |
| Relative Humidity Lag 1 | -451.195 | (-1398.699 ,427.119) |
| Relative Humidity Lag 2 | 112.507 | (-689.604 ,951.625) |
| Temperature Lag 1 | -149.081 | (-229.461 ,-47.262) |
| Temperature Lag 2 | -156.558 | (-264.94 ,-85.911) |

Table 165: Associations with monthly pneumonia case counts in Chachoengsao

| | Posterior Mean | 95% CrI |
|---|---|---|
| Case Count Lag 1 | 0.504 | (0.378 ,0.635) |
| Case Count Lag 2 | -0.776 | (-0.908 ,-0.648) |
| Absolute Humidity Lag 1 | -24.733 | (-81.943 ,11.069) |
| Absolute Humidity Lag 2 | -9.701 | (-49.936 ,35.81) |
| Total Precipitation Lag 1 | 62457.105 | (-75102.419 ,214064.264) |
| Total Precipitation Lag 2 | -51428.932 | (-199370.086 ,82910.806) |
| Relative Humidity Lag 1 | -31.697 | (-467.888 ,367.691) |
| Relative Humidity Lag 2 | -74.479 | (-429.319 ,280.1) |
| Temperature Lag 1 | -242.969 | (-297.811 ,-155.427) |
| Temperature Lag 2 | -244.365 | (-311.289 ,-184.086) |

Table 166: Associations with monthly pneumonia case counts in Chai Nat

| | Posterior Mean | 95% CrI |
|---|---|---|
| Case Count Lag 1 | 0.035 | (-0.097 ,0.163) |
| Case Count Lag 2 | -0.72 | (-0.847 ,-0.589) |
| Absolute Humidity Lag 1 | -10.192 | (-15.764 ,-6.006) |
| Absolute Humidity Lag 2 | -9.543 | (-14.236 ,-5.028) |
| Total Precipitation Lag 1 | -989.947 | (-31463.833 ,30025.122) |
| Total Precipitation Lag 2 | -1935.345 | (-30856.963 ,28435.737) |
| Relative Humidity Lag 1 | 3.912 | (-66.143 ,74.388) |
| Relative Humidity Lag 2 | -20.738 | (-87.664 ,38.685) |
| Temperature Lag 1 | -154.91 | (-161.366 ,-146.031) |
| Temperature Lag 2 | -154.485 | (-161.783 ,-147.137) |

Table 167: Associations with monthly pneumonia case counts in Chaiyaphum

| | Posterior Mean | 95% CrI |
|---|---|---|
| Case Count Lag 1 | 0.425 | (0.302 ,0.557) |
| Case Count Lag 2 | -0.723 | (-0.854 ,-0.601) |
| Absolute Humidity Lag 1 | -14.458 | (-56.189 ,27.049) |
| Absolute Humidity Lag 2 | -4.974 | (-40.191 ,35.139) |
| Total Precipitation Lag 1 | 36134.389 | (-141373.069 ,218059.584) |
| Total Precipitation Lag 2 | -36395.953 | (-224688.865 ,145872.869) |
| Relative Humidity Lag 1 | -7.821 | (-527.572 ,535.898) |
| Relative Humidity Lag 2 | -98.567 | (-612.753 ,411.015) |
| Temperature Lag 1 | -156.261 | (-211.669 ,-102.758) |
| Temperature Lag 2 | -148.548 | (-201.469 ,-101.253) |

Table 168: Associations with monthly pneumonia case counts in Chanthaburi

| | Posterior Mean | 95% CrI |
|---|---|---|
| Case Count Lag 1 | 0.183 | (0.06 ,0.307) |
| Case Count Lag 2 | -0.986 | (-1.11 ,-0.862) |
| Absolute Humidity Lag 1 | -23.089 | (-108.239 ,62.715) |
| Absolute Humidity Lag 2 | 52.786 | (-32.071 ,227.916) |
| Total Precipitation Lag 1 | 28648.394 | (-100691.937 ,167539.972) |
| Total Precipitation Lag 2 | 63793.3 | (-71731.574 ,208279.184) |
| Relative Humidity Lag 1 | -134.864 | (-721.448 ,376.921) |
| Relative Humidity Lag 2 | -454.827 | (-1023.194 ,72.869) |
| Temperature Lag 1 | -324.635 | (-448.417 ,-201.034) |
| Temperature Lag 2 | -371.514 | (-625.314 ,-250.402) |

Table 169: Associations with monthly pneumonia case counts in Chiang Mai

| | Posterior Mean | 95% CrI |
|---|---|---|
| Case Count Lag 1 | 0.467 | (0.349 ,0.59) |
| Case Count Lag 2 | -0.916 | (-1.046 ,-0.794) |
| Absolute Humidity Lag 1 | 112.759 | (-62.389 ,461.688) |
| Absolute Humidity Lag 2 | -9.149 | (-223.133 ,129.375) |
| Total Precipitation Lag 1 | 39714.823 | (-581707.154 ,672540.179) |
| Total Precipitation Lag 2 | -905611.274 | (-1728199.811 ,-84331.175) |
| Relative Humidity Lag 1 | 1425.85 | (-412.135 ,3615.355) |
| Relative Humidity Lag 2 | 1056.334 | (-652.233 ,2917.133) |
| Temperature Lag 1 | -306.598 | (-718.151 ,-95.144) |
| Temperature Lag 2 | -48.343 | (-221.065 ,207.922) |

Table 170: Associations with monthly pneumonia case counts in Chiang Rai

| | Posterior Mean | 95% CrI |
|---|---|---|
| Case Count Lag 1 | 0.115 | (-0.015 ,0.25) |
| Case Count Lag 2 | -0.652 | (-0.782 ,-0.522) |
| Absolute Humidity Lag 1 | 21.033 | (-30.505 ,140.04) |
| Absolute Humidity Lag 2 | -2.053 | (-58.582 ,46.589) |
| Total Precipitation Lag 1 | 64376.462 | (-160006.636 ,288883.507) |
| Total Precipitation Lag 2 | -153758.271 | (-440815.823 ,110759.89) |
| Relative Humidity Lag 1 | 404.523 | (-359.358 ,1271.891) |
| Relative Humidity Lag 2 | 94.233 | (-551.6 ,804.762) |
| Temperature Lag 1 | -160.052 | (-300.421 ,-97.025) |
| Temperature Lag 2 | -91.352 | (-151.214 ,-21.316) |

Table 171: Associations with monthly pneumonia case counts in Chon Buri

| | Posterior Mean | 95% CrI |
|---|---|---|
| Case Count Lag 1 | 0.261 | (0.134 ,0.389) |
| Case Count Lag 2 | -0.904 | (-1.034 ,-0.775) |
| Absolute Humidity Lag 1 | -34.706 | (-146.396 ,38.131) |
| Absolute Humidity Lag 2 | 13.382 | (-61.969 ,136.377) |
| Total Precipitation Lag 1 | -167270.063 | (-419796.875 ,65194.456) |
| Total Precipitation Lag 2 | -4661.469 | (-215621.404 ,210285.649) |
| Relative Humidity Lag 1 | 308.23 | (-340.354 ,1017.717) |
| Relative Humidity Lag 2 | -298.573 | (-907.347 ,284.168) |
| Temperature Lag 1 | -280.55 | (-392.432 ,-115.157) |
| Temperature Lag 2 | -286.707 | (-466.17 ,-173.361) |

Table 172: Associations with monthly pneumonia case counts in Chumphon

| | Posterior Mean | 95% CrI |
|---|---|---|
| Case Count Lag 1 | 0.318 | (0.193 ,0.447) |
| Case Count Lag 2 | -0.744 | (-0.867 ,-0.619) |
| Absolute Humidity Lag 1 | -19.794 | (-58.157 ,32.397) |
| Absolute Humidity Lag 2 | 0.964 | (-33.497 ,85.779) |
| Total Precipitation Lag 1 | -48203.235 | (-116735.851 ,15489.816) |
| Total Precipitation Lag 2 | 56019.539 | (-6999.003 ,123071.913) |
| Relative Humidity Lag 1 | 146.817 | (-242.377 ,546.836) |
| Relative Humidity Lag 2 | -255.802 | (-599.78 ,80.755) |
| Temperature Lag 1 | -377.311 | (-455.496 ,-319.858) |
| Temperature Lag 2 | -380.901 | (-509.308 ,-328.869) |

Table 173: Associations with monthly pneumonia case counts in Kalasin

| | Posterior Mean | 95% CrI |
|---|---|---|
| Case Count Lag 1 | 0.309 | (0.185 ,0.444) |
| Case Count Lag 2 | -0.548 | (-0.678 ,-0.426) |
| Absolute Humidity Lag 1 | -9.972 | (-22.758 ,-1.124) |
| Absolute Humidity Lag 2 | -7.366 | (-16.486 ,3.218) |
| Total Precipitation Lag 1 | 79158.043 | (12254.015 ,146083.904) |
| Total Precipitation Lag 2 | -65477.018 | (-132923.814 ,3046.643) |
| Relative Humidity Lag 1 | -14.044 | (-190.963 ,144.93) |
| Relative Humidity Lag 2 | -24.896 | (-175.97 ,122.641) |
| Temperature Lag 1 | -125.627 | (-137.924 ,-107.907) |
| Temperature Lag 2 | -125.46 | (-140.281 ,-112.702) |

Table 174: Associations with monthly pneumonia case counts in Kamphaeng Phet

| | Posterior Mean | 95% CrI |
|---|---|---|
| Case Count Lag 1 | 0.128 | (-0.007 ,0.261) |
| Case Count Lag 2 | -0.776 | (-0.909 ,-0.639) |
| Absolute Humidity Lag 1 | -8.371 | (-37.949 ,23.985) |
| Absolute Humidity Lag 2 | -3.321 | (-27.675 ,34.392) |
| Total Precipitation Lag 1 | 28311.692 | (-119324.307 ,181119.369) |
| Total Precipitation Lag 2 | -111578.173 | (-289718.771 ,48943.257) |
| Relative Humidity Lag 1 | 162.282 | (-305.783 ,662.47) |
| Relative Humidity Lag 2 | 82.642 | (-357.074 ,585.625) |
| Temperature Lag 1 | -142.329 | (-187.131 ,-102.555) |
| Temperature Lag 2 | -144.62 | (-199.218 ,-110.957) |

Table 175: Associations with monthly pneumonia case counts in Kanchanaburi

| | Posterior Mean | 95% CrI |
|---|---|---|
| Case Count Lag 1 | 0.04 | (-0.081 ,0.168) |
| Case Count Lag 2 | -0.713 | (-0.839 ,-0.593) |
| Absolute Humidity Lag 1 | -18.737 | (-73.904 ,24.637) |
| Absolute Humidity Lag 2 | -3.096 | (-42.086 ,56.771) |
| Total Precipitation Lag 1 | 250937.344 | (83139.77 ,416949.306) |
| Total Precipitation Lag 2 | -218708.289 | (-396762.539 ,-46763.17) |
| Relative Humidity Lag 1 | 8.866 | (-501.737 ,535.914) |
| Relative Humidity Lag 2 | 143.463 | (-349.46 ,695.95) |
| Temperature Lag 1 | -199.4 | (-259.549 ,-126.34) |
| Temperature Lag 2 | -204.858 | (-286.404 ,-151.018) |

Table 176: Associations with monthly pneumonia case counts in Khon Kaen

| | Posterior Mean | 95% CrI |
|---|---|---|
| Case Count Lag 1 | 0.329 | (0.198 ,0.462) |
| Case Count Lag 2 | -0.738 | (-0.874 ,-0.594) |
| Absolute Humidity Lag 1 | -12.032 | (-58.616 ,40.905) |
| Absolute Humidity Lag 2 | -1.542 | (-44.488 ,48.032) |
| Total Precipitation Lag 1 | 2089.613 | (-304814.03 ,300457.635) |
| Total Precipitation Lag 2 | -54945.582 | (-390458.405 ,247252.158) |
| Relative Humidity Lag 1 | 29.701 | (-711.758 ,781.36) |
| Relative Humidity Lag 2 | -183.136 | (-881.596 ,508.727) |
| Temperature Lag 1 | -139.602 | (-215.704 ,-73.541) |
| Temperature Lag 2 | -125.19 | (-194.175 ,-62.575) |

Table 177: Associations with monthly pneumonia case counts in Krabi

| | Posterior Mean | 95% CrI |
|---|---|---|
| Case Count Lag 1 | -0.308 | (-0.441 ,-0.179) |
| Case Count Lag 2 | -0.732 | (-0.855 ,-0.604) |
| Absolute Humidity Lag 1 | -22.979 | (-54.663 ,4.245) |
| Absolute Humidity Lag 2 | -16.124 | (-46.095 ,15.664) |
| Total Precipitation Lag 1 | -24094.231 | (-77538.023 ,24814.869) |
| Total Precipitation Lag 2 | -50613.477 | (-103567.346 ,2599.298) |
| Relative Humidity Lag 1 | -2.272 | (-255.931 ,245.046) |
| Relative Humidity Lag 2 | 254.573 | (15.794 ,497.796) |
| Temperature Lag 1 | -379.522 | (-419.685 ,-332.319) |
| Temperature Lag 2 | -373.998 | (-420.633 ,-325.224) |

Table 178: Associations with monthly pneumonia case counts in Lampang

| | Posterior Mean | 95% CrI |
|---|---|---|
| Case Count Lag 1 | 0.052 | (-0.077 ,0.183) |
| Case Count Lag 2 | -0.88 | (-1.008 ,-0.751) |
| Absolute Humidity Lag 1 | 22.442 | (-27.41 ,129.435) |
| Absolute Humidity Lag 2 | -4.188 | (-57.783 ,45.109) |
| Total Precipitation Lag 1 | 90674.195 | (-177392.435 ,386093.66) |
| Total Precipitation Lag 2 | -301402.902 | (-624669.129 ,16889.318) |
| Relative Humidity Lag 1 | 334.045 | (-368.933 ,1195.659) |
| Relative Humidity Lag 2 | 363.344 | (-301.697 ,1105.106) |
| Temperature Lag 1 | -177.344 | (-318.216 ,-111.463) |
| Temperature Lag 2 | -118.752 | (-182.112 ,-47.998) |

Table 179: Associations with monthly pneumonia case counts in Lamphun

| | Posterior Mean | 95% CrI |
|---|---|---|
| Case Count Lag 1 | 0.235 | (0.11 ,0.363) |
| Case Count Lag 2 | -0.636 | (-0.766 ,-0.511) |
| Absolute Humidity Lag 1 | -5.166 | (-20.437 ,21.413) |
| Absolute Humidity Lag 2 | -4.8 | (-25.867 ,14.612) |
| Total Precipitation Lag 1 | -13150.752 | (-130557.162 ,105105.09) |
| Total Precipitation Lag 2 | -186125.437 | (-328415.522 ,-48052.051) |
| Relative Humidity Lag 1 | 74.281 | (-197.746 ,392.676) |
| Relative Humidity Lag 2 | 331.031 | (48.494 ,622.18) |
| Temperature Lag 1 | -128.85 | (-162.302 ,-108.894) |
| Temperature Lag 2 | -114.231 | (-140.271 ,-84.933) |

Table 180: Associations with monthly pneumonia case counts in Loei

| | Posterior Mean | 95% CrI |
|---|---|---|
| Case Count Lag 1 | 0.003 | (-0.124 ,0.133) |
| Case Count Lag 2 | -0.908 | (-1.038 ,-0.779) |
| Absolute Humidity Lag 1 | -8.02 | (-20.138 ,7.981) |
| Absolute Humidity Lag 2 | -3.686 | (-14.026 ,16.693) |
| Total Precipitation Lag 1 | 56143.529 | (-22276.261 ,136031.24) |
| Total Precipitation Lag 2 | -31602.832 | (-118884.712 ,46947.94) |
| Relative Humidity Lag 1 | 48.978 | (-161.946 ,279.225) |
| Relative Humidity Lag 2 | -60.432 | (-273.929 ,149.558) |
| Temperature Lag 1 | -129.151 | (-149.99 ,-114.141) |
| Temperature Lag 2 | -130.563 | (-156.932 ,-117.226) |

Table 181: Associations with monthly pneumonia case counts in Lop Buri

| | Posterior Mean | 95% CrI |
|---|---|---|
| Case Count Lag 1 | 0.06 | (-0.071 ,0.19) |
| Case Count Lag 2 | -0.772 | (-0.906 ,-0.649) |
| Absolute Humidity Lag 1 | -16.058 | (-49.789 ,15.372) |
| Absolute Humidity Lag 2 | -10.533 | (-41.847 ,20.311) |
| Total Precipitation Lag 1 | -52713.902 | (-231967.113 ,112469.504) |
| Total Precipitation Lag 2 | -68935.149 | (-235953.258 ,92328.681) |
| Relative Humidity Lag 1 | 361.152 | (-34.823 ,780.941) |
| Relative Humidity Lag 2 | -225.424 | (-615.209 ,134.575) |
| Temperature Lag 1 | -181.611 | (-230.835 ,-130.065) |
| Temperature Lag 2 | -176.011 | (-223.355 ,-126.058) |

Table 182: Associations with monthly pneumonia case counts in Mae Hong Son

| | Posterior Mean | 95% CrI |
|---|---|---|
| Case Count Lag 1 | 0.135 | (0.003 ,0.264) |
| Case Count Lag 2 | -0.706 | (-0.833 ,-0.577) |
| Absolute Humidity Lag 1 | -7.742 | (-15.728 ,3.514) |
| Absolute Humidity Lag 2 | -6.007 | (-13.196 ,5.275) |
| Total Precipitation Lag 1 | 70407.477 | (29059.243 ,112860.825) |
| Total Precipitation Lag 2 | -26534.166 | (-80456.957 ,21635.033) |
| Relative Humidity Lag 1 | -82.063 | (-199.698 ,21.667) |
| Relative Humidity Lag 2 | 31.846 | (-60.544 ,140.027) |
| Temperature Lag 1 | -131.62 | (-145.336 ,-122.17) |
| Temperature Lag 2 | -127.487 | (-140.722 ,-118.423) |

Table 183: Associations with monthly pneumonia case counts in Maha Sarakham

| | Posterior Mean | 95% CrI |
|---|---|---|
| Case Count Lag 1 | 0.353 | (0.223 ,0.482) |
| Case Count Lag 2 | -0.822 | (-0.949 ,-0.691) |
| Absolute Humidity Lag 1 | -11.123 | (-27.832 ,2.051) |
| Absolute Humidity Lag 2 | -6.005 | (-18.15 ,11.176) |
| Total Precipitation Lag 1 | 67245.272 | (-17779.648 ,157266.824) |
| Total Precipitation Lag 2 | 29013.058 | (-53391.706 ,117954.536) |
| Relative Humidity Lag 1 | -81.893 | (-309.445 ,137.199) |
| Relative Humidity Lag 2 | -91.147 | (-310.203 ,109.164) |
| Temperature Lag 1 | -131.941 | (-151.401 ,-108.772) |
| Temperature Lag 2 | -131.933 | (-156.701 ,-114.48) |

Table 184: Associations with monthly pneumonia case counts in Mukdahan

| | Posterior Mean | 95% CrI |
|---|---|---|
| Case Count Lag 1 | 0.267 | (0.136 ,0.407) |
| Case Count Lag 2 | -0.532 | (-0.666 ,-0.405) |
| Absolute Humidity Lag 1 | -11.528 | (-29.173 ,0.183) |
| Absolute Humidity Lag 2 | -5.527 | (-17.893 ,9.684) |
| Total Precipitation Lag 1 | 116315.303 | (43771.192 ,189142.663) |
| Total Precipitation Lag 2 | -51278.715 | (-126535.379 ,19262.531) |
| Relative Humidity Lag 1 | -55.442 | (-277.778 ,149.697) |
| Relative Humidity Lag 2 | -41.123 | (-237.549 ,143.711) |
| Temperature Lag 1 | -121.895 | (-137.839 ,-97.851) |
| Temperature Lag 2 | -122.659 | (-143.051 ,-106.507) |

Table 185: Associations with monthly pneumonia case counts in Nakhon Nayok

| | Posterior Mean | 95% CrI |
|---|---|---|
| Case Count Lag 1 | 0.139 | (0.01 ,0.272) |
| Case Count Lag 2 | -0.581 | (-0.708 ,-0.455) |
| Absolute Humidity Lag 1 | -13.918 | (-21.793 ,-5.348) |
| Absolute Humidity Lag 2 | -13.622 | (-21.485 ,-6.086) |
| Total Precipitation Lag 1 | -20598.435 | (-44958.721 ,3481.325) |
| Total Precipitation Lag 2 | -2591.135 | (-25400.237 ,20613.921) |
| Relative Humidity Lag 1 | 106.806 | (17.158 ,199.022) |
| Relative Humidity Lag 2 | -45.983 | (-134.519 ,36.503) |
| Temperature Lag 1 | -220.818 | (-233.357 ,-209.933) |
| Temperature Lag 2 | -217.673 | (-228.619 ,-205.675) |

Table 186: Associations with monthly pneumonia case counts in Nakhon Pathom

| | Posterior Mean | 95% CrI |
|---|---|---|
| Case Count Lag 1 | 0.371 | (0.239 ,0.503) |
| Case Count Lag 2 | -0.754 | (-0.89 ,-0.624) |
| Absolute Humidity Lag 1 | -26.967 | (-102.174 ,32.893) |
| Absolute Humidity Lag 2 | 1.284 | (-53.981 ,76.953) |
| Total Precipitation Lag 1 | -40494.456 | (-366960.218 ,284600.265) |
| Total Precipitation Lag 2 | -129467.952 | (-440170.638 ,183605.447) |
| Relative Humidity Lag 1 | 136.404 | (-548.012 ,816.755) |
| Relative Humidity Lag 2 | -142.919 | (-810.187 ,487.526) |
| Temperature Lag 1 | -203.045 | (-299.52 ,-82.622) |
| Temperature Lag 2 | -198.979 | (-321.705 ,-112.205) |

Table 187: Associations with monthly pneumonia case counts in Nakhon Phanom

| | Posterior Mean | 95% CrI |
|---|---|---|
| Case Count Lag 1 | 0.152 | (0.028 ,0.277) |
| Case Count Lag 2 | -0.885 | (-1.007 ,-0.757) |
| Absolute Humidity Lag 1 | -9.735 | (-26.963 ,8.3) |
| Absolute Humidity Lag 2 | 9.016 | (-9.173 ,42.821) |
| Total Precipitation Lag 1 | 148602.327 | (76596.766 ,220914.438) |
| Total Precipitation Lag 2 | -14688.159 | (-93372.4 ,58304.279) |
| Relative Humidity Lag 1 | -142.771 | (-391.63 ,108.155) |
| Relative Humidity Lag 2 | -184.295 | (-427.797 ,55.894) |
| Temperature Lag 1 | -120.995 | (-145.165 ,-98.511) |
| Temperature Lag 2 | -131.794 | (-175.622 ,-108.163) |

Table 188: Associations with monthly pneumonia case counts in Nakhon Ratchasima

| | Posterior Mean | 95% CrI |
|---|---|---|
| Case Count Lag 1 | 0.483 | (0.352 ,0.615) |
| Case Count Lag 2 | -0.747 | (-0.884 ,-0.613) |
| Absolute Humidity Lag 1 | -30.971 | (-188.004 ,124.555) |
| Absolute Humidity Lag 2 | 6.897 | (-142.494 ,161.948) |
| Total Precipitation Lag 1 | -19252.003 | (-876433.395 ,863207.009) |
| Total Precipitation Lag 2 | -260630.311 | (-1145793.884 ,621859.389) |
| Relative Humidity Lag 1 | 281.021 | (-1889.207 ,2479.297) |
| Relative Humidity Lag 2 | -44.891 | (-2132.918 ,2040.723) |
| Temperature Lag 1 | -187.561 | (-413.974 ,36.439) |
| Temperature Lag 2 | -138.383 | (-359.038 ,76.261) |

Table 189: Associations with monthly pneumonia case counts in Nakhon Sawan

| | Posterior Mean | 95% CrI |
|---|---|---|
| Case Count Lag 1 | 0.427 | (0.298 ,0.557) |
| Case Count Lag 2 | -0.813 | (-0.939 ,-0.686) |
| Absolute Humidity Lag 1 | -10.633 | (-54.602 ,37.319) |
| Absolute Humidity Lag 2 | -1.002 | (-38.08 ,50.642) |
| Total Precipitation Lag 1 | -26104.549 | (-280148.851 ,221900.985) |
| Total Precipitation Lag 2 | -158313.854 | (-427125.275 ,98681.978) |
| Relative Humidity Lag 1 | 295.892 | (-359.415 ,978.835) |
| Relative Humidity Lag 2 | -82.962 | (-724.276 ,557.421) |
| Temperature Lag 1 | -158.309 | (-233.962 ,-93.254) |
| Temperature Lag 2 | -152.829 | (-234.449 ,-94.183) |

Table 190: Associations with monthly pneumonia case counts in Nakhon Si Thammarat

| | Posterior Mean | 95% CrI |
|---|---|---|
| Case Count Lag 1 | 0.029 | (-0.097 ,0.155) |
| Case Count Lag 2 | -1.11 | (-1.233 ,-0.978) |
| Absolute Humidity Lag 1 | -17.138 | (-85.647 ,87.381) |
| Absolute Humidity Lag 2 | 14.743 | (-53.136 ,142.716) |
| Total Precipitation Lag 1 | -37467.496 | (-156990.039 ,79311.398) |
| Total Precipitation Lag 2 | 55759.696 | (-58526.353 ,177103.913) |
| Relative Humidity Lag 1 | -500.615 | (-1332.55 ,311.379) |
| Relative Humidity Lag 2 | 389.291 | (-292.234 ,1135.773) |
| Temperature Lag 1 | -389.92 | (-557.825 ,-283.654) |
| Temperature Lag 2 | -361.979 | (-555.891 ,-259.109) |

Table 191: Associations with monthly pneumonia case counts in Nan

| | Posterior Mean | 95% CrI |
|---|---|---|
| Case Count Lag 1 | 0.268 | (0.135 ,0.397) |
| Case Count Lag 2 | -0.86 | (-0.996 ,-0.727) |
| Absolute Humidity Lag 1 | -11.231 | (-30.112 ,4.82) |
| Absolute Humidity Lag 2 | -5.056 | (-19.515 ,13.614) |
| Total Precipitation Lag 1 | 90630.888 | (26018.343 ,156657.64) |
| Total Precipitation Lag 2 | -42710.241 | (-124330.394 ,29625.07) |
| Relative Humidity Lag 1 | -38.666 | (-290.889 ,190.693) |
| Relative Humidity Lag 2 | 21.972 | (-191.028 ,256.149) |
| Temperature Lag 1 | -128.954 | (-149.407 ,-106.387) |
| Temperature Lag 2 | -125.904 | (-148.735 ,-107.977) |

Table 192: Associations with monthly pneumonia case counts in Narathiwat

| | Posterior Mean | 95% CrI |
|---|---|---|
| Case Count Lag 1 | -0.34 | (-0.465 ,-0.214) |
| Case Count Lag 2 | -0.953 | (-1.076 ,-0.829) |
| Absolute Humidity Lag 1 | -19.221 | (-52.359 ,6.76) |
| Absolute Humidity Lag 2 | -9.239 | (-33.248 ,31.302) |
| Total Precipitation Lag 1 | -12327.629 | (-52211.63 ,24132.516) |
| Total Precipitation Lag 2 | 53301.224 | (11566.524 ,94551.947) |
| Relative Humidity Lag 1 | 184.364 | (-143.866 ,523.422) |
| Relative Humidity Lag 2 | -7.113 | (-331.596 ,309.015) |
| Temperature Lag 1 | -313.636 | (-350.968 ,-265.197) |
| Temperature Lag 2 | -315.294 | (-374.092 ,-279.694) |

Table 193: Associations with monthly pneumonia case counts in Nong Bua Lam Phu

| | Posterior Mean | 95% CrI |
|---|---|---|
| Case Count Lag 1 | 0.353 | (0.222 ,0.484) |
| Case Count Lag 2 | -0.617 | (-0.744 ,-0.491) |
| Absolute Humidity Lag 1 | -7.572 | (-14.098 ,0.372) |
| Absolute Humidity Lag 2 | -7.253 | (-14.663 ,-1.223) |
| Total Precipitation Lag 1 | -13672.79 | (-61820.278 ,30204.492) |
| Total Precipitation Lag 2 | 13589.556 | (-32636.959 ,63043.962) |
| Relative Humidity Lag 1 | -0.384 | (-120.136 ,124.864) |
| Relative Humidity Lag 2 | -59.798 | (-184.479 ,47.085) |
| Temperature Lag 1 | -119.357 | (-130.318 ,-110.881) |
| Temperature Lag 2 | -115.535 | (-123.623 ,-105.672) |

Table 194: Associations with monthly pneumonia case counts in Nong Khai

| | Posterior Mean | 95% CrI |
|---|---|---|
| Case Count Lag 1 | 0.267 | (0.134 ,0.395) |
| Case Count Lag 2 | -0.573 | (-0.703 ,-0.447) |
| Absolute Humidity Lag 1 | -9.811 | (-43.316 ,27.115) |
| Absolute Humidity Lag 2 | -7.514 | (-46.734 ,24.534) |
| Total Precipitation Lag 1 | 203074.979 | (35152.862 ,386305.986) |
| Total Precipitation Lag 2 | 47104.45 | (-128294.769 ,239344.564) |
| Relative Humidity Lag 1 | -278.085 | (-927.191 ,266.548) |
| Relative Humidity Lag 2 | -327.071 | (-867.65 ,164.319) |
| Temperature Lag 1 | -127.839 | (-178.645 ,-85.435) |
| Temperature Lag 2 | -110.234 | (-151.986 ,-57.888) |

Table 195: Associations with monthly pneumonia case counts in Nonthaburi

| | Posterior Mean | 95% CrI |
|---|---|---|
| Case Count Lag 1 | 0.157 | (0.027 ,0.284) |
| Case Count Lag 2 | -0.94 | (-1.07 ,-0.808) |
| Absolute Humidity Lag 1 | -15.15 | (-46.92 ,15.573) |
| Absolute Humidity Lag 2 | -5.603 | (-34.26 ,30.946) |
| Total Precipitation Lag 1 | -84471.176 | (-251676.894 ,67267.242) |
| Total Precipitation Lag 2 | 77.764 | (-138349.967 ,138726.573) |
| Relative Humidity Lag 1 | 275.617 | (-52.999 ,633.901) |
| Relative Humidity Lag 2 | -148.952 | (-469.314 ,156.967) |
| Temperature Lag 1 | -206.685 | (-257.367 ,-155.291) |
| Temperature Lag 2 | -197.063 | (-255.484 ,-151.736) |

Table 196: Associations with monthly pneumonia case counts in Pathum Thani

| | Posterior Mean | 95% CrI |
|---|---|---|
| Case Count Lag 1 | 0.46 | (0.338 ,0.585) |
| Case Count Lag 2 | -0.971 | (-1.096 ,-0.845) |
| Absolute Humidity Lag 1 | -15.287 | (-46.676 ,18.521) |
| Absolute Humidity Lag 2 | -3.723 | (-32.786 ,32.004) |
| Total Precipitation Lag 1 | -15520.319 | (-154990.109 ,119496.054) |
| Total Precipitation Lag 2 | -43678.393 | (-175975.067 ,79061.954) |
| Relative Humidity Lag 1 | 36.799 | (-312.493 ,430.415) |
| Relative Humidity Lag 2 | 2.524 | (-319.006 ,334.18) |
| Temperature Lag 1 | -212.891 | (-267.314 ,-163.942) |
| Temperature Lag 2 | -199.686 | (-255.07 ,-152.569) |

Table 197: Associations with monthly pneumonia case counts in Pattani

| | Posterior Mean | 95% CrI |
|---|---|---|
| Case Count Lag 1 | -0.316 | (-0.441 ,-0.182) |
| Case Count Lag 2 | -0.921 | (-1.048 ,-0.799) |
| Absolute Humidity Lag 1 | -17.655 | (-28.503 ,-4.729) |
| Absolute Humidity Lag 2 | -17.423 | (-29.779 ,-5.434) |
| Total Precipitation Lag 1 | 11707.854 | (-10378.757 ,34451.763) |
| Total Precipitation Lag 2 | -6953.065 | (-30595.768 ,14885.482) |
| Relative Humidity Lag 1 | -119.226 | (-244.43 ,-4.526) |
| Relative Humidity Lag 2 | 86.806 | (-25.347 ,210.187) |
| Temperature Lag 1 | -327.864 | (-348.315 ,-311.012) |
| Temperature Lag 2 | -324.266 | (-342.465 ,-304.58) |

Table 198: Associations with monthly pneumonia case counts in Phangnga

| | Posterior Mean | 95% CrI |
|---|---|---|
| Case Count Lag 1 | 0.098 | (-0.032 ,0.224) |
| Case Count Lag 2 | -0.991 | (-1.117 ,-0.86) |
| Absolute Humidity Lag 1 | -26.214 | (-54.861 ,0.229) |
| Absolute Humidity Lag 2 | -21.43 | (-45.067 ,9.538) |
| Total Precipitation Lag 1 | -13049.977 | (-44387.524 ,15113.859) |
| Total Precipitation Lag 2 | -25927.669 | (-56207.331 ,3027.43) |
| Relative Humidity Lag 1 | 54.351 | (-98.856 ,227.86) |
| Relative Humidity Lag 2 | 141.184 | (-18.531 ,300.185) |
| Temperature Lag 1 | -456.775 | (-497.428 ,-412.058) |
| Temperature Lag 2 | -458.341 | (-503.825 ,-421.424) |

Table 199: Associations with monthly pneumonia case counts in Phatthalung

| | Posterior Mean | 95% CrI |
|---|---|---|
| Case Count Lag 1 | -0.121 | (-0.259 ,0.008) |
| Case Count Lag 2 | -0.786 | (-0.906 ,-0.662) |
| Absolute Humidity Lag 1 | -25.304 | (-70.534 ,13.281) |
| Absolute Humidity Lag 2 | -8.577 | (-42.848 ,46.411) |
| Total Precipitation Lag 1 | 20939.482 | (-33181.262 ,81540.549) |
| Total Precipitation Lag 2 | 17490.519 | (-40226.29 ,72790.374) |
| Relative Humidity Lag 1 | -81.469 | (-536.397 ,343.886) |
| Relative Humidity Lag 2 | -94.863 | (-524.13 ,327.892) |
| Temperature Lag 1 | -383.744 | (-444.228 ,-312.903) |
| Temperature Lag 2 | -380.619 | (-463.865 ,-327.133) |

Table 200: Associations with monthly pneumonia case counts in Phayao

| | Posterior Mean | 95% CrI |
|---|---|---|
| Case Count Lag 1 | 0.311 | (0.184 ,0.44) |
| Case Count Lag 2 | -0.691 | (-0.82 ,-0.561) |
| Absolute Humidity Lag 1 | 34.462 | (-14.306 ,116.944) |
| Absolute Humidity Lag 2 | -7.589 | (-51.759 ,21.826) |
| Total Precipitation Lag 1 | 112296.287 | (-38236.923 ,269156.179) |
| Total Precipitation Lag 2 | -66233.658 | (-246945.473 ,101745.965) |
| Relative Humidity Lag 1 | 27.036 | (-493.206 ,583.032) |
| Relative Humidity Lag 2 | 265.047 | (-159.461 ,717.428) |
| Temperature Lag 1 | -186.992 | (-292.731 ,-122.93) |
| Temperature Lag 2 | -101.043 | (-139.637 ,-43.173) |

Table 201: Associations with monthly pneumonia case counts in Phetchabun

| | Posterior Mean | 95% CrI |
|---|---|---|
| Case Count Lag 1 | 0.411 | (0.287 ,0.54) |
| Case Count Lag 2 | -0.856 | (-0.98 ,-0.731) |
| Absolute Humidity Lag 1 | -11.656 | (-35.21 ,13.156) |
| Absolute Humidity Lag 2 | -8.496 | (-29.418 ,15.421) |
| Total Precipitation Lag 1 | 36086.532 | (-67132.409 ,141405.753) |
| Total Precipitation Lag 2 | -12539.116 | (-124560.026 ,92676.979) |
| Relative Humidity Lag 1 | 15.335 | (-282.558 ,329.049) |
| Relative Humidity Lag 2 | -114.77 | (-394.775 ,152.513) |
| Temperature Lag 1 | -168.4 | (-201.824 ,-136.358) |
| Temperature Lag 2 | -162.444 | (-194.926 ,-133.599) |

Table 202: Associations with monthly pneumonia case counts in Phetchaburi

| | Posterior Mean | 95% CrI |
|---|---|---|
| Case Count Lag 1 | 0.03 | (-0.099 ,0.163) |
| Case Count Lag 2 | -0.817 | (-0.954 ,-0.69) |
| Absolute Humidity Lag 1 | -17.997 | (-49.153 ,3.964) |
| Absolute Humidity Lag 2 | -7.61 | (-29.319 ,26.408) |
| Total Precipitation Lag 1 | -57411.758 | (-171373.898 ,50223.873) |
| Total Precipitation Lag 2 | 22121.559 | (-79204.736 ,130067.594) |
| Relative Humidity Lag 1 | 16.371 | (-271.821 ,310.404) |
| Relative Humidity Lag 2 | -53.436 | (-305.404 ,192.401) |
| Temperature Lag 1 | -228.246 | (-259.621 ,-185.047) |
| Temperature Lag 2 | -231.384 | (-280.872 ,-201.243) |

Table 203: Associations with monthly pneumonia case counts in Phichit

| | Posterior Mean | 95% CrI |
|---|---|---|
| Case Count Lag 1 | 0.135 | (0.006 ,0.267) |
| Case Count Lag 2 | -0.704 | (-0.835 ,-0.573) |
| Absolute Humidity Lag 1 | -9.324 | (-26.76 ,11.154) |
| Absolute Humidity Lag 2 | -8.887 | (-27.95 ,9.933) |
| Total Precipitation Lag 1 | 99369.705 | (14470.188 ,186373.417) |
| Total Precipitation Lag 2 | -51667.297 | (-142804.084 ,35326.687) |
| Relative Humidity Lag 1 | -96.417 | (-417.002 ,170.012) |
| Relative Humidity Lag 2 | 79.125 | (-161.456 ,357.61) |
| Temperature Lag 1 | -154.899 | (-185.113 ,-129.319) |
| Temperature Lag 2 | -148.012 | (-175.524 ,-119.171) |

Table 204: Associations with monthly pneumonia case counts in Phitsanulok

| | Posterior Mean | 95% CrI |
|---|---|---|
| Case Count Lag 1 | 0.007 | (-0.125 ,0.137) |
| Case Count Lag 2 | -0.77 | (-0.9 ,-0.646) |
| Absolute Humidity Lag 1 | -4.737 | (-62.541 ,91.698) |
| Absolute Humidity Lag 2 | 5.522 | (-52.739 ,103.699) |
| Total Precipitation Lag 1 | 409479.168 | (100685.016 ,710934.803) |
| Total Precipitation Lag 2 | -494559.165 | (-827788.003 ,-151495.12) |
| Relative Humidity Lag 1 | 165.706 | (-826.747 ,1269.812) |
| Relative Humidity Lag 2 | 668.068 | (-285.135 ,1807.7) |
| Temperature Lag 1 | -167.696 | (-298.629 ,-88.561) |
| Temperature Lag 2 | -155.877 | (-289.65 ,-77.029) |

Table 205: Associations with monthly pneumonia case counts in Phra Nakhon Si Ayutthaya

| | Posterior Mean | 95% CrI |
|---|---|---|
| Case Count Lag 1 | 0.174 | (0.051 ,0.299) |
| Case Count Lag 2 | -1.105 | (-1.232 ,-0.974) |
| Absolute Humidity Lag 1 | -16.383 | (-52.336 ,22.832) |
| Absolute Humidity Lag 2 | -3.706 | (-38.521 ,35.717) |
| Total Precipitation Lag 1 | -213136.284 | (-385576.914 ,-48436.155) |
| Total Precipitation Lag 2 | 2315.025 | (-146516.084 ,158273.108) |
| Relative Humidity Lag 1 | 336.129 | (-27.262 ,709.727) |
| Relative Humidity Lag 2 | -119.551 | (-458.05 ,190.827) |
| Temperature Lag 1 | -213.578 | (-277.955 ,-153.845) |
| Temperature Lag 2 | -197.068 | (-261.014 ,-138.988) |

Table 206: Associations with monthly pneumonia case counts in Phrae

| | Posterior Mean | 95% CrI |
|---|---|---|
| Case Count Lag 1 | 0.083 | (-0.048 ,0.212) |
| Case Count Lag 2 | -0.276 | (-0.411 ,-0.145) |
| Absolute Humidity Lag 1 | -6.437 | (-18.368 ,13.128) |
| Absolute Humidity Lag 2 | -8.867 | (-27.495 ,3.473) |
| Total Precipitation Lag 1 | 4042.74 | (-76798.554 ,83113.165) |
| Total Precipitation Lag 2 | -51385.853 | (-144377.972 ,33733.186) |
| Relative Humidity Lag 1 | 88.403 | (-132.16 ,344.672) |
| Relative Humidity Lag 2 | 40.639 | (-166.687 ,264.245) |
| Temperature Lag 1 | -135.084 | (-161.363 ,-118.645) |
| Temperature Lag 2 | -126.526 | (-143.523 ,-101.845) |

Table 207: Associations with monthly pneumonia case counts in Phuket

| | Posterior Mean | 95% CrI |
|---|---|---|
| Case Count Lag 1 | -0.121 | (-0.253 ,0.008) |
| Case Count Lag 2 | -0.722 | (-0.845 ,-0.594) |
| Absolute Humidity Lag 1 | -20.376 | (-111.313 ,88.021) |
| Absolute Humidity Lag 2 | -28.861 | (-123.911 ,64.805) |
| Total Precipitation Lag 1 | -63575.68 | (-173162.475 ,35821.369) |
| Total Precipitation Lag 2 | -18714.75 | (-121143.485 ,82206.498) |
| Relative Humidity Lag 1 | 255.752 | (-417.217 ,967.585) |
| Relative Humidity Lag 2 | 560.341 | (-95.852 ,1218.494) |
| Temperature Lag 1 | -524.44 | (-693.64 ,-381.174) |
| Temperature Lag 2 | -528.668 | (-672.846 ,-380.488) |

Table 208: Associations with monthly pneumonia case counts in Prachin Buri

| | Posterior Mean | 95% CrI |
|---|---|---|
| Case Count Lag 1 | 0.331 | (0.2 ,0.463) |
| Case Count Lag 2 | -0.75 | (-0.882 ,-0.622) |
| Absolute Humidity Lag 1 | -15.535 | (-38.648 ,6.897) |
| Absolute Humidity Lag 2 | -12.127 | (-35.519 ,10.266) |
| Total Precipitation Lag 1 | 4441 | (-69342.372 ,79949.108) |
| Total Precipitation Lag 2 | -14258.828 | (-94770.866 ,62675.372) |
| Relative Humidity Lag 1 | 134.152 | (-95.032 ,389.245) |
| Relative Humidity Lag 2 | -129.021 | (-361.425 ,87.263) |
| Temperature Lag 1 | -224.885 | (-258.326 ,-190.456) |
| Temperature Lag 2 | -217.254 | (-249.462 ,-182.051) |

Table 209: Associations with monthly pneumonia case counts in Prachuap Khiri Khan

| | Posterior Mean | 95% CrI |
|---|---|---|
| Case Count Lag 1 | 0.165 | (0.043 ,0.298) |
| Case Count Lag 2 | -0.545 | (-0.675 ,-0.416) |
| Absolute Humidity Lag 1 | -16.673 | (-48.937 ,22.956) |
| Absolute Humidity Lag 2 | -8.985 | (-41.218 ,34.712) |
| Total Precipitation Lag 1 | -50302.926 | (-166010.469 ,58030.86) |
| Total Precipitation Lag 2 | 92102.513 | (-13824.507 ,204792.199) |
| Relative Humidity Lag 1 | 130.231 | (-186.159 ,461.467) |
| Relative Humidity Lag 2 | -302.41 | (-603.063 ,-18.994) |
| Temperature Lag 1 | -297.25 | (-357.428 ,-249.713) |
| Temperature Lag 2 | -287.523 | (-351.52 ,-240.824) |

Table 210: Associations with monthly pneumonia case counts in Ranong

| | Posterior Mean | 95% CrI |
|---|---|---|
| Case Count Lag 1 | -0.061 | (-0.193 ,0.067) |
| Case Count Lag 2 | -0.704 | (-0.836 ,-0.577) |
| Absolute Humidity Lag 1 | -22.842 | (-32.263 ,-16.003) |
| Absolute Humidity Lag 2 | -21.846 | (-31.256 ,-15.297) |
| Total Precipitation Lag 1 | -13832.248 | (-25882.742 ,-2449.58) |
| Total Precipitation Lag 2 | 9747.811 | (-1104.528 ,21500.013) |
| Relative Humidity Lag 1 | 11.753 | (-49.963 ,79.067) |
| Relative Humidity Lag 2 | -35.76 | (-97.549 ,17.865) |
| Temperature Lag 1 | -390.367 | (-400.759 ,-376.038) |
| Temperature Lag 2 | -389.037 | (-398.754 ,-374.943) |

Table 211: Associations with monthly pneumonia case counts in Ratchaburi

| | Posterior Mean | 95% CrI |
|---|---|---|
| Case Count Lag 1 | -0.083 | (-0.21 ,0.052) |
| Case Count Lag 2 | -0.82 | (-0.948 ,-0.698) |
| Absolute Humidity Lag 1 | -20.516 | (-65.369 ,10.343) |
| Absolute Humidity Lag 2 | -5.537 | (-37.171 ,38.399) |
| Total Precipitation Lag 1 | -28346.722 | (-224415.584 ,159266.84) |
| Total Precipitation Lag 2 | -31523.928 | (-225102.756 ,144229.091) |
| Relative Humidity Lag 1 | 106.005 | (-294.406 ,549.348) |
| Relative Humidity Lag 2 | -116.375 | (-515.65 ,258.61) |
| Temperature Lag 1 | -197.45 | (-244.161 ,-131.627) |
| Temperature Lag 2 | -198.383 | (-261.97 ,-150.609) |

Table 212: Associations with monthly pneumonia case counts in Rayong

| | Posterior Mean | 95% CrI |
|---|---|---|
| Case Count Lag 1 | 0.413 | (0.29 ,0.535) |
| Case Count Lag 2 | -0.968 | (-1.092 ,-0.841) |
| Absolute Humidity Lag 1 | 6.334 | (-117.328 ,217.454) |
| Absolute Humidity Lag 2 | 46.345 | (-74.323 ,293) |
| Total Precipitation Lag 1 | -46694.438 | (-332569.106 ,233798.708) |
| Total Precipitation Lag 2 | 75197.698 | (-174373.762 ,355229.257) |
| Relative Humidity Lag 1 | -265.323 | (-1244.284 ,721.703) |
| Relative Humidity Lag 2 | -390.912 | (-1318.126 ,453.511) |
| Temperature Lag 1 | -382.496 | (-698.268 ,-201.848) |
| Temperature Lag 2 | -344.132 | (-704.537 ,-163.477) |

Table 213: Associations with monthly pneumonia case counts in Roi Et

| | Posterior Mean | 95% CrI |
|---|---|---|
| Case Count Lag 1 | 0.447 | (0.322 ,0.583) |
| Case Count Lag 2 | -0.64 | (-0.782 ,-0.512) |
| Absolute Humidity Lag 1 | -9.314 | (-25.009 ,7.097) |
| Absolute Humidity Lag 2 | -6.754 | (-21.813 ,9.375) |
| Total Precipitation Lag 1 | 78237.076 | (-9537.66 ,169620.237) |
| Total Precipitation Lag 2 | -8461.969 | (-94338.688 ,78203.153) |
| Relative Humidity Lag 1 | -161.408 | (-413.913 ,72.576) |
| Relative Humidity Lag 2 | -61.992 | (-282.828 ,139.415) |
| Temperature Lag 1 | -135.827 | (-159.658 ,-113.588) |
| Temperature Lag 2 | -131.869 | (-154.075 ,-110.08) |

Table 214: Associations with monthly pneumonia case counts in Sa Kaeo

| | Posterior Mean | 95% CrI |
|---|---|---|
| Case Count Lag 1 | 0.401 | (0.275 ,0.524) |
| Case Count Lag 2 | -0.957 | (-1.083 ,-0.834) |
| Absolute Humidity Lag 1 | -15.212 | (-26.2 ,-9.639) |
| Absolute Humidity Lag 2 | -11.873 | (-18.064 ,-3.774) |
| Total Precipitation Lag 1 | -9575.944 | (-39317.683 ,16710.018) |
| Total Precipitation Lag 2 | -10598.459 | (-39109.644 ,16213.797) |
| Relative Humidity Lag 1 | 28.846 | (-39.882 ,104.788) |
| Relative Humidity Lag 2 | -18.913 | (-83.293 ,40.798) |
| Temperature Lag 1 | -211.538 | (-219.792 ,-195.357) |
| Temperature Lag 2 | -212.238 | (-224.164 ,-202.797) |

Table 215: Associations with monthly pneumonia case counts in Sakon Nakhon

| | Posterior Mean | 95% CrI |
|---|---|---|
| Case Count Lag 1 | 0.007 | (-0.122 ,0.14) |
| Case Count Lag 2 | -0.915 | (-1.048 ,-0.786) |
| Absolute Humidity Lag 1 | -9.677 | (-19.137 ,-1.251) |
| Absolute Humidity Lag 2 | -7.211 | (-15.527 ,1.127) |
| Total Precipitation Lag 1 | 89905.398 | (41548.546 ,137320.913) |
| Total Precipitation Lag 2 | -20692.154 | (-74686.016 ,27740.207) |
| Relative Humidity Lag 1 | -39.239 | (-183.144 ,96.394) |
| Relative Humidity Lag 2 | -85.216 | (-221.336 ,43.723) |
| Temperature Lag 1 | -119.828 | (-131.5 ,-107.255) |
| Temperature Lag 2 | -116.566 | (-127.785 ,-105.15) |

Table 216: Associations with monthly pneumonia case counts in Samut Prakan

| | Posterior Mean | 95% CrI |
|---|---|---|
| Case Count Lag 1 | 0.376 | (0.256 ,0.502) |
| Case Count Lag 2 | -1.035 | (-1.158 ,-0.907) |
| Absolute Humidity Lag 1 | -14.127 | (-83.154 ,79.315) |
| Absolute Humidity Lag 2 | 30.387 | (-38.521 ,164.071) |
| Total Precipitation Lag 1 | -289013.609 | (-625319.871 ,23695.244) |
| Total Precipitation Lag 2 | 37944.743 | (-236966.26 ,320850.898) |
| Relative Humidity Lag 1 | 655.137 | (-150.247 ,1513.167) |
| Relative Humidity Lag 2 | -234.089 | (-983.653 ,502.273) |
| Temperature Lag 1 | -250.643 | (-396.943 ,-148.376) |
| Temperature Lag 2 | -246.974 | (-457.737 ,-137.803) |

Table 217: Associations with monthly pneumonia case counts in Samut Sakhon

| | Posterior Mean | 95% CrI |
|---|---|---|
| Case Count Lag 1 | 0.336 | (0.204 ,0.47) |
| Case Count Lag 2 | -0.444 | (-0.576 ,-0.315) |
| Absolute Humidity Lag 1 | -19.368 | (-72.949 ,21.843) |
| Absolute Humidity Lag 2 | -12.128 | (-58.005 ,34.453) |
| Total Precipitation Lag 1 | -108008.307 | (-317152.753 ,82636.958) |
| Total Precipitation Lag 2 | -4761.44 | (-186474.251 ,178858.735) |
| Relative Humidity Lag 1 | 365.424 | (-134.808 ,893.795) |
| Relative Humidity Lag 2 | -177.318 | (-655.359 ,253.776) |
| Temperature Lag 1 | -218.432 | (-283.028 ,-135.96) |
| Temperature Lag 2 | -215.328 | (-286.095 ,-142.173) |

Table 218: Associations with monthly pneumonia case counts in Samut Songkhram

| | Posterior Mean | 95% CrI |
|---|---|---|
| Case Count Lag 1 | -0.169 | (-0.293 ,-0.04) |
| Case Count Lag 2 | -0.913 | (-1.037 ,-0.789) |
| Absolute Humidity Lag 1 | -14.273 | (-21.877 ,-9.614) |
| Absolute Humidity Lag 2 | -11.201 | (-15.76 ,-3.221) |
| Total Precipitation Lag 1 | 2494.451 | (-24737.357 ,27726.449) |
| Total Precipitation Lag 2 | 3885.202 | (-21559.562 ,28319.124) |
| Relative Humidity Lag 1 | -7.315 | (-69.03 ,56.337) |
| Relative Humidity Lag 2 | -51.806 | (-110.806 ,6.531) |
| Temperature Lag 1 | -209.155 | (-216.314 ,-197.432) |
| Temperature Lag 2 | -209.221 | (-221.66 ,-202.118) |

Table 219: Associations with monthly pneumonia case counts in Saraburi

| | Posterior Mean | 95% CrI |
|---|---|---|
| Case Count Lag 1 | 0.141 | (0.013 ,0.271) |
| Case Count Lag 2 | -0.739 | (-0.879 ,-0.608) |
| Absolute Humidity Lag 1 | -16.447 | (-35.375 ,-2.5) |
| Absolute Humidity Lag 2 | -7.752 | (-20.602 ,11.434) |
| Total Precipitation Lag 1 | -7125.083 | (-77023.264 ,58909.479) |
| Total Precipitation Lag 2 | -35890.826 | (-104586.112 ,28381.347) |
| Relative Humidity Lag 1 | 57.291 | (-110.651 ,234.232) |
| Relative Humidity Lag 2 | -10.551 | (-171.216 ,155.341) |
| Temperature Lag 1 | -200.476 | (-222.045 ,-171.597) |
| Temperature Lag 2 | -202.078 | (-231.962 ,-182.495) |

Table 220: Associations with monthly pneumonia case counts in Satun

| | Posterior Mean | 95% CrI |
|---|---|---|
| Case Count Lag 1 | -0.3 | (-0.424 ,-0.171) |
| Case Count Lag 2 | -1.021 | (-1.146 ,-0.894) |
| Absolute Humidity Lag 1 | -26.265 | (-37.28 ,-16.859) |
| Absolute Humidity Lag 2 | -24.771 | (-35.352 ,-14.597) |
| Total Precipitation Lag 1 | -9446.001 | (-23656.154 ,4119.581) |
| Total Precipitation Lag 2 | 3258.819 | (-9614.577 ,17129.953) |
| Relative Humidity Lag 1 | 13.498 | (-63.202 ,97.293) |
| Relative Humidity Lag 2 | -21.393 | (-89.081 ,45.067) |
| Temperature Lag 1 | -459.756 | (-474.04 ,-442.329) |
| Temperature Lag 2 | -459.596 | (-474.533 ,-443.003) |

Table 221: Associations with monthly pneumonia case counts in Si Sa Ket

| | Posterior Mean | 95% CrI |
|---|---|---|
| Case Count Lag 1 | 0.253 | (0.124 ,0.38) |
| Case Count Lag 2 | -1.066 | (-1.194 ,-0.936) |
| Absolute Humidity Lag 1 | -13.198 | (-36.945 ,5.654) |
| Absolute Humidity Lag 2 | -4.265 | (-20.593 ,22.69) |
| Total Precipitation Lag 1 | 12164.881 | (-78859.64 ,106296.009) |
| Total Precipitation Lag 2 | -80276.983 | (-181743.04 ,13818.973) |
| Relative Humidity Lag 1 | 6.977 | (-272.964 ,263.76) |
| Relative Humidity Lag 2 | -2.612 | (-239.826 ,245.753) |
| Temperature Lag 1 | -152.007 | (-178.93 ,-119.176) |
| Temperature Lag 2 | -154.373 | (-192.764 ,-130.624) |

Table 222: Associations with monthly pneumonia case counts in Sing Buri

| | Posterior Mean | 95% CrI |
|---|---|---|
| Case Count Lag 1 | 0.046 | (-0.084 ,0.176) |
| Case Count Lag 2 | -0.682 | (-0.812 ,-0.561) |
| Absolute Humidity Lag 1 | -11.961 | (-20.153 ,-7.352) |
| Absolute Humidity Lag 2 | -10.6 | (-16.892 ,-4.818) |
| Total Precipitation Lag 1 | -26497.313 | (-61566.351 ,6342.646) |
| Total Precipitation Lag 2 | -777.372 | (-34050.82 ,31713.004) |
| Relative Humidity Lag 1 | 39.443 | (-32.017 ,118.281) |
| Relative Humidity Lag 2 | -44.385 | (-111.017 ,18.738) |
| Temperature Lag 1 | -172.591 | (-180.164 ,-159.272) |
| Temperature Lag 2 | -172.81 | (-181.995 ,-162.38) |

Table 223: Associations with monthly pneumonia case counts in Songkhla

| | Posterior Mean | 95% CrI |
|---|---|---|
| Case Count Lag 1 | 0.132 | (0.006 ,0.255) |
| Case Count Lag 2 | -0.957 | (-1.091 ,-0.831) |
| Absolute Humidity Lag 1 | -16.187 | (-87.95 ,52.142) |
| Absolute Humidity Lag 2 | -28.703 | (-123.199 ,29.502) |
| Total Precipitation Lag 1 | 84063.369 | (-17670.141 ,197455.657) |
| Total Precipitation Lag 2 | 31259.86 | (-67733.643 ,135347.295) |
| Relative Humidity Lag 1 | -340.111 | (-968.227 ,259.585) |
| Relative Humidity Lag 2 | 454.493 | (-80.713 ,1015.538) |
| Temperature Lag 1 | -384.855 | (-487.764 ,-275.636) |
| Temperature Lag 2 | -385.255 | (-474.026 ,-235.813) |

Table 224: Associations with monthly pneumonia case counts in Sukhothai

| | Posterior Mean | 95% CrI |
|---|---|---|
| Case Count Lag 1 | 0.107 | (-0.02 ,0.234) |
| Case Count Lag 2 | -0.549 | (-0.676 ,-0.419) |
| Absolute Humidity Lag 1 | -8.328 | (-34.034 ,13.917) |
| Absolute Humidity Lag 2 | -8.22 | (-32.014 ,14.66) |
| Total Precipitation Lag 1 | 62724.049 | (-84693.652 ,217907.336) |
| Total Precipitation Lag 2 | -78655.59 | (-250684.493 ,81239.785) |
| Relative Humidity Lag 1 | 168.979 | (-208.199 ,584.786) |
| Relative Humidity Lag 2 | -27.703 | (-423.333 ,342.443) |
| Temperature Lag 1 | -126.368 | (-157.918 ,-88.602) |
| Temperature Lag 2 | -127.245 | (-162.066 ,-92.616) |

Table 225: Associations with monthly pneumonia case counts in Suphan Buri

| | Posterior Mean | 95% CrI |
|---|---|---|
| Case Count Lag 1 | 0.243 | (0.119 ,0.371) |
| Case Count Lag 2 | -0.937 | (-1.066 ,-0.809) |
| Absolute Humidity Lag 1 | -10.337 | (-29.646 ,15.449) |
| Absolute Humidity Lag 2 | -8.366 | (-28.96 ,12.064) |
| Total Precipitation Lag 1 | -58465.247 | (-200554.766 ,72538.782) |
| Total Precipitation Lag 2 | 32443.878 | (-94186.464 ,165706.218) |
| Relative Humidity Lag 1 | 87.829 | (-174.401 ,358.193) |
| Relative Humidity Lag 2 | -151.544 | (-411.849 ,78.116) |
| Temperature Lag 1 | -180.704 | (-220.464 ,-151.502) |
| Temperature Lag 2 | -168.252 | (-200.004 ,-136.098) |

Table 226: Associations with monthly pneumonia case counts in Surat Thani

| | Posterior Mean | 95% CrI |
|---|---|---|
| Case Count Lag 1 | 0.107 | (-0.022 ,0.233) |
| Case Count Lag 2 | -1.141 | (-1.266 ,-1.01) |
| Absolute Humidity Lag 1 | -30.549 | (-123.188 ,60.986) |
| Absolute Humidity Lag 2 | -9.171 | (-109.435 ,74.596) |
| Total Precipitation Lag 1 | -77965.85 | (-229503.288 ,63476.518) |
| Total Precipitation Lag 2 | 17321.763 | (-121675.543 ,159355.65) |
| Relative Humidity Lag 1 | -212.408 | (-1087.195 ,628.787) |
| Relative Humidity Lag 2 | 349.273 | (-389.733 ,1184.662) |
| Temperature Lag 1 | -356.673 | (-497.385 ,-213.368) |
| Temperature Lag 2 | -318.141 | (-444.573 ,-160.875) |

Table 227: Associations with monthly pneumonia case counts in Surin

| | Posterior Mean | 95% CrI |
|---|---|---|
| Case Count Lag 1 | 0.507 | (0.381 ,0.639) |
| Case Count Lag 2 | -0.787 | (-0.924 ,-0.659) |
| Absolute Humidity Lag 1 | -16.188 | (-68.089 ,16.605) |
| Absolute Humidity Lag 2 | -3.815 | (-36.84 ,40.401) |
| Total Precipitation Lag 1 | 134017.916 | (-56011.074 ,345567.929) |
| Total Precipitation Lag 2 | -131182.579 | (-337115.639 ,65134.603) |
| Relative Humidity Lag 1 | -168.13 | (-766.942 ,346.774) |
| Relative Humidity Lag 2 | 21.925 | (-466.211 ,532.904) |
| Temperature Lag 1 | -145.025 | (-192.936 ,-70.098) |
| Temperature Lag 2 | -148.212 | (-214.434 ,-97.888) |

Table 228: Associations with monthly pneumonia case counts in Tak

| | Posterior Mean | 95% CrI |
|---|---|---|
| Case Count Lag 1 | 0.072 | (-0.059 ,0.208) |
| Case Count Lag 2 | -0.705 | (-0.833 ,-0.575) |
| Absolute Humidity Lag 1 | -11.088 | (-32.247 ,11.153) |
| Absolute Humidity Lag 2 | -1.932 | (-18.078 ,30.172) |
| Total Precipitation Lag 1 | 113035.46 | (36540.228 ,190625.047) |
| Total Precipitation Lag 2 | -80315.815 | (-169173.057 ,4673.324) |
| Relative Humidity Lag 1 | -74.872 | (-322.998 ,138.132) |
| Relative Humidity Lag 2 | 80.827 | (-132.728 ,341.667) |
| Temperature Lag 1 | -163.853 | (-192.062 ,-137.437) |
| Temperature Lag 2 | -167.602 | (-207.006 ,-147.336) |

Table 229: Associations with monthly pneumonia case counts in Trang

| | Posterior Mean | 95% CrI |
|---|---|---|
| Case Count Lag 1 | -0.368 | (-0.49 ,-0.24) |
| Case Count Lag 2 | -0.997 | (-1.122 ,-0.877) |
| Absolute Humidity Lag 1 | -52.336 | (-174.386 ,-1.505) |
| Absolute Humidity Lag 2 | -19.656 | (-93.745 ,40.031) |
| Total Precipitation Lag 1 | -24074.447 | (-122993.893 ,72205.085) |
| Total Precipitation Lag 2 | -44629.019 | (-153351.004 ,54968.491) |
| Relative Humidity Lag 1 | -116.503 | (-726.639 ,433.112) |
| Relative Humidity Lag 2 | 236.726 | (-245.481 ,750.108) |
| Temperature Lag 1 | -415.29 | (-494.218 ,-218.695) |
| Temperature Lag 2 | -414.488 | (-506.928 ,-297.223) |

Table 230: Associations with monthly pneumonia case counts in Trat

| | Posterior Mean | 95% CrI |
|---|---|---|
| Case Count Lag 1 | 0.242 | (0.12 ,0.363) |
| Case Count Lag 2 | -0.938 | (-1.058 ,-0.818) |
| Absolute Humidity Lag 1 | -23.267 | (-55.459 ,11.745) |
| Absolute Humidity Lag 2 | 27.563 | (-20.631 ,104.995) |
| Total Precipitation Lag 1 | 3218.116 | (-26471.676 ,32083.678) |
| Total Precipitation Lag 2 | 8415.773 | (-19591.87 ,38508.805) |
| Relative Humidity Lag 1 | 7.786 | (-134.592 ,154.382) |
| Relative Humidity Lag 2 | -55.819 | (-208.865 ,102.82) |
| Temperature Lag 1 | -392.424 | (-443.717 ,-346.185) |
| Temperature Lag 2 | -446.844 | (-558.226 ,-377.673) |

Table 231: Associations with monthly pneumonia case counts in Ubon Ratchathani

| | Posterior Mean | 95% CrI |
|---|---|---|
| Case Count Lag 1 | 0.434 | (0.297 ,0.572) |
| Case Count Lag 2 | -0.786 | (-0.923 ,-0.653) |
| Absolute Humidity Lag 1 | -12.149 | (-127.714 ,110.258) |
| Absolute Humidity Lag 2 | 21.787 | (-76.037 ,181.259) |
| Total Precipitation Lag 1 | -347147.531 | (-852049.78 ,140468.497) |
| Total Precipitation Lag 2 | -532677.191 | (-1051452.172 ,-36763.221) |
| Relative Humidity Lag 1 | 1451.652 | (-56.943 ,3115.516) |
| Relative Humidity Lag 2 | -161.784 | (-1485.131 ,1093.122) |
| Temperature Lag 1 | -164.626 | (-344.673 ,-0.554) |
| Temperature Lag 2 | -184.631 | (-413.962 ,-47.996) |

Table 232: Associations with monthly pneumonia case counts in Udon Thani

| | Posterior Mean | 95% CrI |
|---|---|---|
| Case Count Lag 1 | 0.464 | (0.338 ,0.59) |
| Case Count Lag 2 | -0.835 | (-0.964 ,-0.709) |
| Absolute Humidity Lag 1 | -9.142 | (-31.994 ,14.132) |
| Absolute Humidity Lag 2 | -8.826 | (-35.153 ,10.049) |
| Total Precipitation Lag 1 | 22829.158 | (-111393.898 ,160743.036) |
| Total Precipitation Lag 2 | 66222.562 | (-80805.189 ,228330.743) |
| Relative Humidity Lag 1 | 72.352 | (-296.391 ,455.704) |
| Relative Humidity Lag 2 | -248.564 | (-632.758 ,95.973) |
| Temperature Lag 1 | -121.412 | (-153.239 ,-90.576) |
| Temperature Lag 2 | -113.222 | (-139.403 ,-77.572) |

Table 233: Associations with monthly pneumonia case counts in Uthai Thani

| | Posterior Mean | 95% CrI |
|---|---|---|
| Case Count Lag 1 | -0.027 | (-0.158 ,0.107) |
| Case Count Lag 2 | -0.732 | (-0.862 ,-0.609) |
| Absolute Humidity Lag 1 | -9.833 | (-20.49 ,-2.665) |
| Absolute Humidity Lag 2 | -7.942 | (-14.508 ,3.178) |
| Total Precipitation Lag 1 | 2506.75 | (-40804.04 ,45153.749) |
| Total Precipitation Lag 2 | -62105.693 | (-110776.949 ,-15601.566) |
| Relative Humidity Lag 1 | 54.64 | (-52.48 ,171.668) |
| Relative Humidity Lag 2 | 52.431 | (-64.846 ,184.026) |
| Temperature Lag 1 | -149.323 | (-158.776 ,-135.231) |
| Temperature Lag 2 | -151.997 | (-167.099 ,-142.945) |

Table 234: Associations with monthly pneumonia case counts in Uttaradit

| | Posterior Mean | 95% CrI |
|---|---|---|
| Case Count Lag 1 | 0.171 | (0.048 ,0.3) |
| Case Count Lag 2 | -0.907 | (-1.042 ,-0.776) |
| Absolute Humidity Lag 1 | -3.978 | (-29.43 ,40.905) |
| Absolute Humidity Lag 2 | -8.92 | (-41.287 ,16.212) |
| Total Precipitation Lag 1 | 123943.873 | (-13451.739 ,267918.929) |
| Total Precipitation Lag 2 | -237491.614 | (-400261.729 ,-80091.541) |
| Relative Humidity Lag 1 | 221.454 | (-216.253 ,722.288) |
| Relative Humidity Lag 2 | 112.112 | (-291.253 ,549.954) |
| Temperature Lag 1 | -146.719 | (-205.058 ,-114.4) |
| Temperature Lag 2 | -128.114 | (-161.488 ,-84.848) |

Table 235: Associations with monthly pneumonia case counts in Yala

| | Posterior Mean | 95% CrI |
|---|---|---|
| Case Count Lag 1 | -0.108 | (-0.236 ,0.022) |
| Case Count Lag 2 | -0.72 | (-0.844 ,-0.593) |
| Absolute Humidity Lag 1 | -18.44 | (-28.042 ,-6.01) |
| Absolute Humidity Lag 2 | -16.773 | (-29.141 ,-5.947) |
| Total Precipitation Lag 1 | -9841.443 | (-34600.904 ,12647.441) |
| Total Precipitation Lag 2 | -1101.302 | (-23595.078 ,22159.679) |
| Relative Humidity Lag 1 | 3.064 | (-111.057 ,125.807) |
| Relative Humidity Lag 2 | 43.757 | (-65.675 ,152.806) |
| Temperature Lag 1 | -335.39 | (-354.211 ,-320.905) |
| Temperature Lag 2 | -329.729 | (-344.956 ,-311.879) |

Table 236: Associations with monthly pneumonia case counts in Yasothon

| | Posterior Mean | 95% CrI |
|---|---|---|
| Case Count Lag 1 | 0.236 | (0.109 ,0.365) |
| Case Count Lag 2 | -0.848 | (-0.979 ,-0.721) |
| Absolute Humidity Lag 1 | -8.786 | (-20.807 ,4.085) |
| Absolute Humidity Lag 2 | -4.355 | (-14.024 ,14.245) |
| Total Precipitation Lag 1 | 40717.22 | (-19426.584 ,102872.74) |
| Total Precipitation Lag 2 | -51533.236 | (-117309.062 ,10073.504) |
| Relative Humidity Lag 1 | 24.623 | (-153.648 ,212.486) |
| Relative Humidity Lag 2 | -36.741 | (-207.148 ,134.695) |
| Temperature Lag 1 | -136.726 | (-155.182 ,-119.989) |
| Temperature Lag 2 | -138.318 | (-164.645 ,-124.471) |

### 5.4 Coefficient output for URTI case counts as the dependent variable

Table 237: Associations with monthly URTI case counts in Amnat Charoen

| | Posterior Mean | 95% CrI |
|---|---|---|
| Case Count Lag 1 | 1.044 | (0.662 ,1.434) |
| Case Count Lag 2 | -0.56 | (-0.952 ,-0.18) |
| Absolute Humidity Lag 1 | -8.456 | (-22.173 ,11.522) |
| Absolute Humidity Lag 2 | -3.388 | (-15.542 ,20.297) |
| Total Precipitation Lag 1 | 78911.938 | (9465.199 ,154739.834) |
| Total Precipitation Lag 2 | -27687.173 | (-103676.978 ,44357.554) |
| Relative Humidity Lag 1 | -62.239 | (-315.043 ,168.028) |
| Relative Humidity Lag 2 | 28.63 | (-179.222 ,269.419) |
| Temperature Lag 1 | -142.844 | (-170.49 ,-123.455) |
| Temperature Lag 2 | -139.198 | (-171.389 ,-122.182) |

Table 238: Associations with monthly URTI case counts in Ang Thong

| | Posterior Mean | 95% CrI |
|---|---|---|
| Case Count Lag 1 | 0.56 | (0.369 ,0.742) |
| Case Count Lag 2 | -0.636 | (-0.828 ,-0.445) |
| Absolute Humidity Lag 1 | -19.49 | (-39.749 ,-6.845) |
| Absolute Humidity Lag 2 | -5.899 | (-17.598 ,12.437) |
| Total Precipitation Lag 1 | -103661.381 | (-175666.195 ,-34790.759) |
| Total Precipitation Lag 2 | -73101.386 | (-138523.669 ,-6653.129) |
| Relative Humidity Lag 1 | 114.563 | (-27.356 ,258.597) |
| Relative Humidity Lag 2 | -28.718 | (-159.214 ,101.551) |
| Temperature Lag 1 | -185.62 | (-206.182 ,-152.146) |
| Temperature Lag 2 | -188.79 | (-218.973 ,-169.953) |

Table 239: Associations with monthly URTI case counts in Bangkok

| | Posterior Mean | 95% CrI |
|---|---|---|
| Case Count Lag 1 | -0.516 | (-0.532 ,-0.499) |
| Case Count Lag 2 | -0.674 | (-0.692 ,-0.657) |
| Absolute Humidity Lag 1 | -40.746 | (-171.675 ,68.75) |
| Absolute Humidity Lag 2 | 62.481 | (-46.384 ,232.727) |
| Total Precipitation Lag 1 | -564930.445 | (-1027702.095 ,-107292.569) |
| Total Precipitation Lag 2 | -23721.299 | (-395384.025 ,368024.837) |
| Relative Humidity Lag 1 | 979.194 | (-222.026 ,2233.811) |
| Relative Humidity Lag 2 | -396.62 | (-1473.813 ,709.9) |
| Temperature Lag 1 | -219.851 | (-391.335 ,-18.738) |
| Temperature Lag 2 | -230.647 | (-497.185 ,-60.776) |

Table 240: Associations with monthly URTI case counts in Buri Ram

| | Posterior Mean | 95% CrI |
|---|---|---|
| Case Count Lag 1 | -0.033 | (-0.124 ,0.052) |
| Case Count Lag 2 | -0.645 | (-0.729 ,-0.56) |
| Absolute Humidity Lag 1 | -17.98 | (-67.006 ,30.799) |
| Absolute Humidity Lag 2 | 5.677 | (-32.28 ,66.665) |
| Total Precipitation Lag 1 | -21884.031 | (-270489.531 ,215538.276) |
| Total Precipitation Lag 2 | -423526.413 | (-672703.345 ,-180551.938) |
| Relative Humidity Lag 1 | 431.462 | (-151.993 ,1016.784) |
| Relative Humidity Lag 2 | 42.707 | (-521.967 ,629.951) |
| Temperature Lag 1 | -164.799 | (-237.041 ,-96.496) |
| Temperature Lag 2 | -159.554 | (-248.462 ,-104.313) |

Table 241: Associations with monthly URTI case counts in Chachoengsao

| | Posterior Mean | 95% CrI |
|---|---|---|
| Case Count Lag 1 | 0.111 | (0.007 ,0.215) |
| Case Count Lag 2 | -0.689 | (-0.79 ,-0.587) |
| Absolute Humidity Lag 1 | -43.117 | (-121.471 ,0.531) |
| Absolute Humidity Lag 2 | -7.06 | (-54.993 ,42.379) |
| Total Precipitation Lag 1 | -14160.348 | (-159266.703 ,123298.413) |
| Total Precipitation Lag 2 | -63044.225 | (-206939.947 ,72199.917) |
| Relative Humidity Lag 1 | 223.309 | (-188.739 ,646.047) |
| Relative Humidity Lag 2 | -114.8 | (-479.621 ,240.86) |
| Temperature Lag 1 | -232.798 | (-298.585 ,-110.511) |
| Temperature Lag 2 | -233.904 | (-306.322 ,-160.601) |

Table 242: Associations with monthly URTI case counts in Chai Nat

| | Posterior Mean | 95% CrI |
|---|---|---|
| Case Count Lag 1 | -0.118 | (-0.221 ,-0.006) |
| Case Count Lag 2 | -0.567 | (-0.668 ,-0.457) |
| Absolute Humidity Lag 1 | -11.925 | (-18.224 ,-8.031) |
| Absolute Humidity Lag 2 | -9.493 | (-14.545 ,-5.61) |
| Total Precipitation Lag 1 | -11290.135 | (-37508.892 ,13568.464) |
| Total Precipitation Lag 2 | -24673.768 | (-50418.277 ,-618.702) |
| Relative Humidity Lag 1 | 21.646 | (-35.593 ,84.037) |
| Relative Humidity Lag 2 | -44.38 | (-102.501 ,7.355) |
| Temperature Lag 1 | -154.641 | (-160.907 ,-144.625) |
| Temperature Lag 2 | -153.28 | (-159.526 ,-145.206) |

Table 243: Associations with monthly URTI case counts in Chaiyaphum

| | Posterior Mean | 95% CrI |
|---|---|---|
| Case Count Lag 1 | 0.139 | (0.031 ,0.243) |
| Case Count Lag 2 | -0.487 | (-0.588 ,-0.375) |
| Absolute Humidity Lag 1 | -16.802 | (-52.224 ,18.067) |
| Absolute Humidity Lag 2 | -1.133 | (-28.466 ,41.735) |
| Total Precipitation Lag 1 | 9730.209 | (-139328.139 ,163630.617) |
| Total Precipitation Lag 2 | -183493.828 | (-335475.355 ,-24574.688) |
| Relative Humidity Lag 1 | 367.427 | (-62.809 ,818.336) |
| Relative Humidity Lag 2 | 44.134 | (-375.109 ,496.739) |
| Temperature Lag 1 | -158.22 | (-204.193 ,-111.228) |
| Temperature Lag 2 | -158.287 | (-215.116 ,-121.68) |

Table 244: Associations with monthly URTI case counts in Chanthaburi

| | Posterior Mean | 95% CrI |
|---|---|---|
| Case Count Lag 1 | -0.451 | (-0.5 ,-0.402) |
| Case Count Lag 2 | -0.784 | (-0.831 ,-0.739) |
| Absolute Humidity Lag 1 | -35.1 | (-94.646 ,-5.079) |
| Absolute Humidity Lag 2 | -5.258 | (-33.838 ,45.774) |
| Total Precipitation Lag 1 | 5456.364 | (-50424.879 ,60545.481) |
| Total Precipitation Lag 2 | -15986.635 | (-72797.988 ,41473.245) |
| Relative Humidity Lag 1 | -63.585 | (-284.382 ,144.082) |
| Relative Humidity Lag 2 | 28.838 | (-173.388 ,239.868) |
| Temperature Lag 1 | -306.102 | (-350.103 ,-221.008) |
| Temperature Lag 2 | -318.64 | (-392.498 ,-277.267) |

Table 245: Associations with monthly URTI case counts in Chiang Mai

| | Posterior Mean | 95% CrI |
|---|---|---|
| Case Count Lag 1 | -0.275 | (-0.32 ,-0.228) |
| Case Count Lag 2 | -0.606 | (-0.652 ,-0.56) |
| Absolute Humidity Lag 1 | 45.511 | (-32.228 ,156.752) |
| Absolute Humidity Lag 2 | -11.437 | (-83.714 ,41.214) |
| Total Precipitation Lag 1 | 584780.491 | (360607.579 ,810076.641) |
| Total Precipitation Lag 2 | -320394.247 | (-587255.197 ,-47874.483) |
| Relative Humidity Lag 1 | -296.156 | (-959.798 ,350.215) |
| Relative Humidity Lag 2 | 414.671 | (-190.966 ,1044.414) |
| Temperature Lag 1 | -225.371 | (-359.032 ,-132.697) |
| Temperature Lag 2 | -100.766 | (-163.879 ,-14.181) |

Table 246: Associations with monthly URTI case counts in Chiang Rai

| | Posterior Mean | 95% CrI |
|---|---|---|
| Case Count Lag 1 | -0.053 | (-0.159 ,0.055) |
| Case Count Lag 2 | -0.66 | (-0.765 ,-0.555) |
| Absolute Humidity Lag 1 | 73.004 | (-8.632 ,166.171) |
| Absolute Humidity Lag 2 | -8.909 | (-60.469 ,36.071) |
| Total Precipitation Lag 1 | 414050.129 | (243654.369 ,607268.346) |
| Total Precipitation Lag 2 | -133253.288 | (-352705.84 ,68383.011) |
| Relative Humidity Lag 1 | -55.848 | (-704.432 ,585.604) |
| Relative Humidity Lag 2 | 564.556 | (0.045 ,1148.448) |
| Temperature Lag 1 | -220.971 | (-335.596 ,-121.351) |
| Temperature Lag 2 | -101.305 | (-155.713 ,-37.529) |

Table 247: Associations with monthly URTI case counts in Chon Buri

| | Posterior Mean | 95% CrI |
|---|---|---|
| Case Count Lag 1 | -0.281 | (-0.347 ,-0.216) |
| Case Count Lag 2 | -0.668 | (-0.734 ,-0.599) |
| Absolute Humidity Lag 1 | -44.538 | (-134.41 ,-0.946) |
| Absolute Humidity Lag 2 | 2.467 | (-42.017 ,75.554) |
| Total Precipitation Lag 1 | -36174.542 | (-159925.325 ,85340.964) |
| Total Precipitation Lag 2 | -104962.542 | (-221128.105 ,8798.577) |
| Relative Humidity Lag 1 | -61.692 | (-446.703 ,292.069) |
| Relative Humidity Lag 2 | -13.451 | (-334.686 ,313.915) |
| Temperature Lag 1 | -264.657 | (-330.257 ,-129.722) |
| Temperature Lag 2 | -280.616 | (-391.515 ,-214.674) |

Table 248: Associations with monthly URTI case counts in Chumphon

| | Posterior Mean | 95% CrI |
|---|---|---|
| Case Count Lag 1 | -0.169 | (-0.255 ,-0.085) |
| Case Count Lag 2 | -0.561 | (-0.642 ,-0.48) |
| Absolute Humidity Lag 1 | -19.832 | (-49.64 ,34.867) |
| Absolute Humidity Lag 2 | -11.279 | (-39.919 ,32.128) |
| Total Precipitation Lag 1 | -53367.237 | (-103518.425 ,-4757.812) |
| Total Precipitation Lag 2 | 4093.413 | (-40546.198 ,50941.877) |
| Relative Humidity Lag 1 | 149.646 | (-146.311 ,444.693) |
| Relative Humidity Lag 2 | -23.792 | (-265.308 ,224.921) |
| Temperature Lag 1 | -385.85 | (-468.6 ,-341.594) |
| Temperature Lag 2 | -365.14 | (-430.087 ,-320.124) |

Table 249: Associations with monthly URTI case counts in Kalasin

| | Posterior Mean | 95% CrI |
|---|---|---|
| Case Count Lag 1 | 0.292 | (0.143 ,0.442) |
| Case Count Lag 2 | -0.401 | (-0.546 ,-0.253) |
| Absolute Humidity Lag 1 | -10.107 | (-23.946 ,4.475) |
| Absolute Humidity Lag 2 | -7.361 | (-20.129 ,6.654) |
| Total Precipitation Lag 1 | 99300.381 | (25847.871 ,173961.085) |
| Total Precipitation Lag 2 | -53983.792 | (-133347.436 ,22355.076) |
| Relative Humidity Lag 1 | 33.124 | (-159.519 ,233.021) |
| Relative Humidity Lag 2 | -49.838 | (-224.205 ,124.996) |
| Temperature Lag 1 | -130.35 | (-151.02 ,-111.14) |
| Temperature Lag 2 | -124.924 | (-143.679 ,-107.185) |

Table 250: Associations with monthly URTI case counts in Kamphaeng Phet

| | Posterior Mean | 95% CrI |
|---|---|---|
| Case Count Lag 1 | -0.138 | (-0.231 ,-0.047) |
| Case Count Lag 2 | -0.622 | (-0.714 ,-0.533) |
| Absolute Humidity Lag 1 | -8.623 | (-29.819 ,19.114) |
| Absolute Humidity Lag 2 | -3.864 | (-21.852 ,24.799) |
| Total Precipitation Lag 1 | 95472.785 | (-7776.547 ,203482.988) |
| Total Precipitation Lag 2 | -84183.164 | (-201481.545 ,27064.936) |
| Relative Humidity Lag 1 | -24.258 | (-350.839 ,289.308) |
| Relative Humidity Lag 2 | -8.257 | (-324.662 ,338.395) |
| Temperature Lag 1 | -152.979 | (-192.15 ,-124.526) |
| Temperature Lag 2 | -142.207 | (-181.99 ,-116.219) |

Table 251: Associations with monthly URTI case counts in Kanchanaburi

| | Posterior Mean | 95% CrI |
|---|---|---|
| Case Count Lag 1 | -0.381 | (-0.453 ,-0.308) |
| Case Count Lag 2 | -0.754 | (-0.824 ,-0.685) |
| Absolute Humidity Lag 1 | -20.092 | (-54.034 ,15.879) |
| Absolute Humidity Lag 2 | -16.165 | (-58.227 ,10.604) |
| Total Precipitation Lag 1 | 142263.349 | (40796.528 ,242762.199) |
| Total Precipitation Lag 2 | -87420.92 | (-195530.001 ,17543.705) |
| Relative Humidity Lag 1 | -15.117 | (-339.316 ,309.737) |
| Relative Humidity Lag 2 | -50.407 | (-383.377 ,275.324) |
| Temperature Lag 1 | -209.837 | (-257.903 ,-163.828) |
| Temperature Lag 2 | -188.547 | (-224.887 ,-131.569) |

Table 252: Associations with monthly URTI case counts in Khon Kaen

| | Posterior Mean | 95% CrI |
|---|---|---|
| Case Count Lag 1 | 0.546 | (0.37 ,0.719) |
| Case Count Lag 2 | -0.519 | (-0.696 ,-0.346) |
| Absolute Humidity Lag 1 | -13.649 | (-67.323 ,51.749) |
| Absolute Humidity Lag 2 | 7.06 | (-37.504 ,78.06) |
| Total Precipitation Lag 1 | -52114.767 | (-387868.623 ,261084.371) |
| Total Precipitation Lag 2 | -133398.382 | (-466500.215 ,201112.009) |
| Relative Humidity Lag 1 | 285.839 | (-463.839 ,1107.147) |
| Relative Humidity Lag 2 | -215.138 | (-1007.115 ,562.099) |
| Temperature Lag 1 | -153.988 | (-248.716 ,-77.591) |
| Temperature Lag 2 | -141.964 | (-243.468 ,-77.497) |

Table 253: Associations with monthly URTI case counts in Krabi

| | Posterior Mean | 95% CrI |
|---|---|---|
| Case Count Lag 1 | 0.227 | (0.068 ,0.399) |
| Case Count Lag 2 | -0.991 | (-1.154 ,-0.829) |
| Absolute Humidity Lag 1 | -28.064 | (-75.75 ,10.589) |
| Absolute Humidity Lag 2 | -1.901 | (-39.779 ,53.937) |
| Total Precipitation Lag 1 | -53337.189 | (-112855.469 ,3186.103) |
| Total Precipitation Lag 2 | -40084.634 | (-97386.665 ,14900.643) |
| Relative Humidity Lag 1 | -166.921 | (-482.668 ,109.892) |
| Relative Humidity Lag 2 | 521.153 | (258.146 ,810.698) |
| Temperature Lag 1 | -384.617 | (-447.274 ,-314.083) |
| Temperature Lag 2 | -375.471 | (-460.753 ,-315.249) |

Table 254: Associations with monthly URTI case counts in Lampang

| | Posterior Mean | 95% CrI |
|---|---|---|
| Case Count Lag 1 | -0.396 | (-0.449 ,-0.344) |
| Case Count Lag 2 | -0.731 | (-0.784 ,-0.68) |
| Absolute Humidity Lag 1 | -9.021 | (-29.532 ,14.722) |
| Absolute Humidity Lag 2 | -5.559 | (-24.329 ,17.049) |
| Total Precipitation Lag 1 | 193967.804 | (84373.743 ,304075.547) |
| Total Precipitation Lag 2 | -108286.35 | (-227538.776 ,5963.3) |
| Relative Humidity Lag 1 | -237.312 | (-526.602 ,27.955) |
| Relative Humidity Lag 2 | 222.455 | (-26.241 ,490.701) |
| Temperature Lag 1 | -134.871 | (-166.484 ,-108.717) |
| Temperature Lag 2 | -123.679 | (-154.093 ,-99.008) |

Table 255: Associations with monthly URTI case counts in Lamphun

| | Posterior Mean | 95% CrI |
|---|---|---|
| Case Count Lag 1 | -0.247 | (-0.295 ,-0.201) |
| Case Count Lag 2 | -0.564 | (-0.61 ,-0.514) |
| Absolute Humidity Lag 1 | -6.371 | (-12.952 ,4.746) |
| Absolute Humidity Lag 2 | -9.9 | (-21.485 ,-3.053) |
| Total Precipitation Lag 1 | 29441.1 | (-11628.102 ,71343.036) |
| Total Precipitation Lag 2 | -60356.147 | (-109198.55 ,-9393.742) |
| Relative Humidity Lag 1 | 10.179 | (-88.313 ,114.721) |
| Relative Humidity Lag 2 | 25.026 | (-77.918 ,124.931) |
| Temperature Lag 1 | -126.419 | (-141.387 ,-117.718) |
| Temperature Lag 2 | -118.608 | (-127.443 ,-102.928) |

Table 256: Associations with monthly URTI case counts in Loei

| | Posterior Mean | 95% CrI |
|---|---|---|
| Case Count Lag 1 | 0.693 | (0.435 ,0.948) |
| Case Count Lag 2 | -0.43 | (-0.692 ,-0.173) |
| Absolute Humidity Lag 1 | -8.805 | (-27.128 ,13.372) |
| Absolute Humidity Lag 2 | -3.764 | (-20.329 ,19.301) |
| Total Precipitation Lag 1 | 64438.328 | (-35933.975 ,171091.672) |
| Total Precipitation Lag 2 | -53131.706 | (-161402.198 ,48578.991) |
| Relative Humidity Lag 1 | 23.694 | (-268.074 ,316.543) |
| Relative Humidity Lag 2 | -69.904 | (-349.42 ,214.256) |
| Temperature Lag 1 | -135.978 | (-165.099 ,-113.338) |
| Temperature Lag 2 | -127.266 | (-157.181 ,-106.292) |

Table 257: Associations with monthly URTI case counts in Lop Buri

| | Posterior Mean | 95% CrI |
|---|---|---|
| Case Count Lag 1 | -0.098 | (-0.197 ,-0.005) |
| Case Count Lag 2 | -0.661 | (-0.754 ,-0.562) |
| Absolute Humidity Lag 1 | -14.854 | (-39.014 ,10.788) |
| Absolute Humidity Lag 2 | -7.064 | (-28.627 ,19.308) |
| Total Precipitation Lag 1 | 10878.702 | (-103072.213 ,128215.463) |
| Total Precipitation Lag 2 | -99012.526 | (-216089.757 ,18399.324) |
| Relative Humidity Lag 1 | 151.568 | (-101.361 ,432.618) |
| Relative Humidity Lag 2 | -173.115 | (-447.315 ,90.245) |
| Temperature Lag 1 | -186.554 | (-225.893 ,-149.821) |
| Temperature Lag 2 | -176.01 | (-216.865 ,-141.879) |

Table 258: Associations with monthly URTI case counts in Mae Hong Son

| | Posterior Mean | 95% CrI |
|---|---|---|
| Case Count Lag 1 | 0.136 | (-0.058 ,0.327) |
| Case Count Lag 2 | -0.487 | (-0.663 ,-0.306) |
| Absolute Humidity Lag 1 | -6.025 | (-20.773 ,18.194) |
| Absolute Humidity Lag 2 | -10.67 | (-31.37 ,2.057) |
| Total Precipitation Lag 1 | 169375.307 | (103475.809 ,236787.804) |
| Total Precipitation Lag 2 | 35312.988 | (-41214.366 ,121719.003) |
| Relative Humidity Lag 1 | -145.668 | (-325.872 ,23.672) |
| Relative Humidity Lag 2 | -57.712 | (-232.666 ,97.871) |
| Temperature Lag 1 | -138.947 | (-167.943 ,-121.264) |
| Temperature Lag 2 | -122.393 | (-137.063 ,-97.264) |

Table 259: Associations with monthly URTI case counts in Maha Sarakham

| | Posterior Mean | 95% CrI |
|---|---|---|
| Case Count Lag 1 | 1.519 | (1.193 ,1.839) |
| Case Count Lag 2 | -0.619 | (-0.947 ,-0.312) |
| Absolute Humidity Lag 1 | -12.564 | (-47.115 ,27.234) |
| Absolute Humidity Lag 2 | -3.07 | (-34.739 ,35.489) |
| Total Precipitation Lag 1 | 118542.466 | (-69770.314 ,323296.707) |
| Total Precipitation Lag 2 | -175207.934 | (-372473.234 ,23558.2) |
| Relative Humidity Lag 1 | 126.55 | (-367.765 ,657.285) |
| Relative Humidity Lag 2 | -29.269 | (-514.368 ,445.059) |
| Temperature Lag 1 | -142.196 | (-199.159 ,-92.632) |
| Temperature Lag 2 | -131.507 | (-187.685 ,-86.784) |

Table 260: Associations with monthly URTI case counts in Mukdahan

| | Posterior Mean | 95% CrI |
|---|---|---|
| Case Count Lag 1 | 0.095 | (-0.019 ,0.206) |
| Case Count Lag 2 | -0.403 | (-0.517 ,-0.286) |
| Absolute Humidity Lag 1 | -7.508 | (-18.247 ,9.152) |
| Absolute Humidity Lag 2 | -6.943 | (-18.636 ,4.963) |
| Total Precipitation Lag 1 | 78589.887 | (21558.368 ,136695.636) |
| Total Precipitation Lag 2 | -1926.996 | (-59598.103 ,60230.708) |
| Relative Humidity Lag 1 | 1.019 | (-176.219 ,179.731) |
| Relative Humidity Lag 2 | -94.099 | (-262.679 ,68.645) |
| Temperature Lag 1 | -129.026 | (-150.896 ,-114.691) |
| Temperature Lag 2 | -120.728 | (-136.24 ,-104.92) |

Table 261: Associations with monthly URTI case counts in Nakhon Nayok

| | Posterior Mean | 95% CrI |
|---|---|---|
| Case Count Lag 1 | 0.238 | (0.078 ,0.394) |
| Case Count Lag 2 | -0.409 | (-0.56 ,-0.255) |
| Absolute Humidity Lag 1 | -15.812 | (-26.585 ,-5.881) |
| Absolute Humidity Lag 2 | -13.743 | (-24.523 ,-4.717) |
| Total Precipitation Lag 1 | -8911.458 | (-36373.454 ,17666.683) |
| Total Precipitation Lag 2 | -21837.262 | (-48260.858 ,4391.218) |
| Relative Humidity Lag 1 | 112.332 | (9.126 ,216.974) |
| Relative Humidity Lag 2 | -27.844 | (-125.36 ,66.873) |
| Temperature Lag 1 | -221.923 | (-236.491 ,-206.757) |
| Temperature Lag 2 | -215.804 | (-228.592 ,-199.859) |

Table 262: Associations with monthly URTI case counts in Nakhon Pathom

| | Posterior Mean | 95% CrI |
|---|---|---|
| Case Count Lag 1 | -0.346 | (-0.381 ,-0.311) |
| Case Count Lag 2 | -0.635 | (-0.672 ,-0.599) |
| Absolute Humidity Lag 1 | -24.888 | (-60.709 ,-3.773) |
| Absolute Humidity Lag 2 | -6.545 | (-30.505 ,18.871) |
| Total Precipitation Lag 1 | -136386.048 | (-249680.397 ,-24350.57) |
| Total Precipitation Lag 2 | -2361.099 | (-100688.532 ,98641.235) |
| Relative Humidity Lag 1 | 37.935 | (-189.657 ,270.204) |
| Relative Humidity Lag 2 | -76.35 | (-292.586 ,127.397) |
| Temperature Lag 1 | -194.259 | (-228.911 ,-136.839) |
| Temperature Lag 2 | -189.785 | (-230.04 ,-151.397) |

Table 263: Associations with monthly URTI case counts in Nakhon Phanom

| | Posterior Mean | 95% CrI |
|---|---|---|
| Case Count Lag 1 | -0.146 | (-0.261 ,-0.029) |
| Case Count Lag 2 | -0.796 | (-0.912 ,-0.681) |
| Absolute Humidity Lag 1 | -7.163 | (-21.153 ,14.231) |
| Absolute Humidity Lag 2 | -6.867 | (-22.69 ,10.71) |
| Total Precipitation Lag 1 | 135121.532 | (60927.471 ,210059.784) |
| Total Precipitation Lag 2 | -6625.598 | (-84340.36 ,69132.059) |
| Relative Humidity Lag 1 | 70.358 | (-168.547 ,324.561) |
| Relative Humidity Lag 2 | -185.023 | (-416.717 ,44.039) |
| Temperature Lag 1 | -124.344 | (-152.411 ,-106.059) |
| Temperature Lag 2 | -115.95 | (-138.638 ,-95.683) |

Table 264: Associations with monthly URTI case counts in Nakhon Ratchasima

| | Posterior Mean | 95% CrI |
|---|---|---|
| Case Count Lag 1 | -0.361 | (-0.389 ,-0.333) |
| Case Count Lag 2 | -0.547 | (-0.575 ,-0.521) |
| Absolute Humidity Lag 1 | -29.932 | (-85.85 ,17.039) |
| Absolute Humidity Lag 2 | 3.899 | (-37.641 ,60.98) |
| Total Precipitation Lag 1 | -36074.698 | (-269517.916 ,196787.065) |
| Total Precipitation Lag 2 | -464059.965 | (-708436.165 ,-232650.205) |
| Relative Humidity Lag 1 | 387.124 | (-197.791 ,992.722) |
| Relative Humidity Lag 2 | 306.211 | (-281.434 ,945.505) |
| Temperature Lag 1 | -165.296 | (-232.935 ,-87.554) |
| Temperature Lag 2 | -156.289 | (-237.917 ,-95.952) |

Table 265: Associations with monthly URTI case counts in Nakhon Sawan

| | Posterior Mean | 95% CrI |
|---|---|---|
| Case Count Lag 1 | -0.332 | (-0.372 ,-0.292) |
| Case Count Lag 2 | -0.606 | (-0.646 ,-0.568) |
| Absolute Humidity Lag 1 | -17.498 | (-40.148 ,-0.8) |
| Absolute Humidity Lag 2 | -8.271 | (-26.436 ,8.148) |
| Total Precipitation Lag 1 | 24050.208 | (-59058.642 ,119154.613) |
| Total Precipitation Lag 2 | -147871.379 | (-250195.043 ,-57296.882) |
| Relative Humidity Lag 1 | -6.842 | (-248.698 ,233.075) |
| Relative Humidity Lag 2 | 111.005 | (-122.713 ,347.225) |
| Temperature Lag 1 | -148.156 | (-172.994 ,-114.073) |
| Temperature Lag 2 | -146.379 | (-171.381 ,-117.92) |

Table 266: Associations with monthly URTI case counts in Nakhon Si Thammarat

| | Posterior Mean | 95% CrI |
|---|---|---|
| Case Count Lag 1 | -0.444 | (-0.535 ,-0.357) |
| Case Count Lag 2 | -0.862 | (-0.948 ,-0.77) |
| Absolute Humidity Lag 1 | -19.25 | (-80.824 ,71.535) |
| Absolute Humidity Lag 2 | 23.27 | (-38.778 ,122.049) |
| Total Precipitation Lag 1 | -66536.503 | (-160654.925 ,20649.707) |
| Total Precipitation Lag 2 | 35189.196 | (-55460.842 ,126457.032) |
| Relative Humidity Lag 1 | -372.339 | (-991.509 ,257.109) |
| Relative Humidity Lag 2 | 484.659 | (-79.213 ,1088.73) |
| Temperature Lag 1 | -405.492 | (-544.565 ,-310.999) |
| Temperature Lag 2 | -361.761 | (-509.533 ,-268.176) |

Table 267: Associations with monthly URTI case counts in Nan

| | Posterior Mean | 95% CrI |
|---|---|---|
| Case Count Lag 1 | 0.144 | (0.021 ,0.263) |
| Case Count Lag 2 | -0.709 | (-0.832 ,-0.59) |
| Absolute Humidity Lag 1 | -7.506 | (-22.288 ,13.189) |
| Absolute Humidity Lag 2 | -10.866 | (-32.451 ,1.71) |
| Total Precipitation Lag 1 | 82966.367 | (29198.074 ,138030.185) |
| Total Precipitation Lag 2 | 0.019 | (-68100.581 ,63604.423) |
| Relative Humidity Lag 1 | 14.029 | (-189.565 ,225.168) |
| Relative Humidity Lag 2 | -93.796 | (-300.032 ,96.909) |
| Temperature Lag 1 | -134.467 | (-160.086 ,-116.491) |
| Temperature Lag 2 | -122.946 | (-138.928 ,-96.81) |

Table 268: Associations with monthly URTI case counts in Narathiwat

| | Posterior Mean | 95% CrI |
|---|---|---|
| Case Count Lag 1 | 0.23 | (0.008 ,0.464) |
| Case Count Lag 2 | -1.001 | (-1.211 ,-0.788) |
| Absolute Humidity Lag 1 | -14.594 | (-54.162 ,50.853) |
| Absolute Humidity Lag 2 | 1.45 | (-42.649 ,68.571) |
| Total Precipitation Lag 1 | -27972.489 | (-89625.335 ,29516.127) |
| Total Precipitation Lag 2 | 3163.396 | (-58710.257 ,65430.704) |
| Relative Humidity Lag 1 | 139.731 | (-361.76 ,692.315) |
| Relative Humidity Lag 2 | 458.332 | (-49.27 ,992.387) |
| Temperature Lag 1 | -325.891 | (-423.231 ,-267.92) |
| Temperature Lag 2 | -309.4 | (-404.365 ,-243.198) |

Table 269: Associations with monthly URTI case counts in Nong Bua Lam Phu

| | Posterior Mean | 95% CrI |
|---|---|---|
| Case Count Lag 1 | 0.429 | (0.282 ,0.581) |
| Case Count Lag 2 | -0.366 | (-0.523 ,-0.207) |
| Absolute Humidity Lag 1 | -8.925 | (-18.804 ,-0.138) |
| Absolute Humidity Lag 2 | -0.308 | (-9.306 ,14.269) |
| Total Precipitation Lag 1 | -6214.548 | (-57531.848 ,43792.299) |
| Total Precipitation Lag 2 | -26167.288 | (-78991.486 ,24957.548) |
| Relative Humidity Lag 1 | 73.192 | (-58.617 ,215.545) |
| Relative Humidity Lag 2 | -36.081 | (-173.184 ,102.517) |
| Temperature Lag 1 | -118.473 | (-130.268 ,-105.44) |
| Temperature Lag 2 | -126.306 | (-146.388 ,-114.011) |

Table 270: Associations with monthly URTI case counts in Nong Khai

| | Posterior Mean | 95% CrI |
|---|---|---|
| Case Count Lag 1 | -0.118 | (-0.181 ,-0.056) |
| Case Count Lag 2 | -0.35 | (-0.411 ,-0.287) |
| Absolute Humidity Lag 1 | -1.107 | (-18.177 ,26.692) |
| Absolute Humidity Lag 2 | -7.559 | (-26.903 ,6.16) |
| Total Precipitation Lag 1 | 126744.368 | (60236.835 ,195773.126) |
| Total Precipitation Lag 2 | 43524.05 | (-30966.156 ,121390.909) |
| Relative Humidity Lag 1 | 33.132 | (-200.944 ,261.391) |
| Relative Humidity Lag 2 | -274.047 | (-507.557 ,-50.681) |
| Temperature Lag 1 | -141.674 | (-178.794 ,-118.281) |
| Temperature Lag 2 | -113.04 | (-131.081 ,-87.812) |

Table 271: Associations with monthly URTI case counts in Nonthaburi

| | Posterior Mean | 95% CrI |
|---|---|---|
| Case Count Lag 1 | -0.435 | (-0.483 ,-0.386) |
| Case Count Lag 2 | -0.731 | (-0.781 ,-0.685) |
| Absolute Humidity Lag 1 | -18.448 | (-36.263 ,-5.849) |
| Absolute Humidity Lag 2 | -4.102 | (-17.763 ,14.658) |
| Total Precipitation Lag 1 | -52753.24 | (-115403.962 ,10761.814) |
| Total Precipitation Lag 2 | -13310.108 | (-71059.972 ,41998.582) |
| Relative Humidity Lag 1 | 66.82 | (-72.303 ,203.866) |
| Relative Humidity Lag 2 | -10.515 | (-141.756 ,127.78) |
| Temperature Lag 1 | -201.844 | (-222.476 ,-173.015) |
| Temperature Lag 2 | -201.359 | (-231.387 ,-179.258) |

Table 272: Associations with monthly URTI case counts in Pathum Thani

| | Posterior Mean | 95% CrI |
|---|---|---|
| Case Count Lag 1 | 0.482 | (0.342 ,0.622) |
| Case Count Lag 2 | -0.814 | (-0.962 ,-0.667) |
| Absolute Humidity Lag 1 | -30.606 | (-91.508 ,2.092) |
| Absolute Humidity Lag 2 | 6.536 | (-28.999 ,57.752) |
| Total Precipitation Lag 1 | -188772.519 | (-343626.012 ,-29066.076) |
| Total Precipitation Lag 2 | 45346.171 | (-91421.626 ,186064.062) |
| Relative Humidity Lag 1 | 1.355 | (-437.501 ,421.949) |
| Relative Humidity Lag 2 | -278.852 | (-667.511 ,102.607) |
| Temperature Lag 1 | -195.295 | (-246.466 ,-99.56) |
| Temperature Lag 2 | -199.871 | (-279.445 ,-142.373) |

Table 273: Associations with monthly URTI case counts in Pattani

| | Posterior Mean | 95% CrI |
|---|---|---|
| Case Count Lag 1 | 1.501 | (1.125 ,1.868) |
| Case Count Lag 2 | -1.131 | (-1.517 ,-0.754) |
| Absolute Humidity Lag 1 | -18.802 | (-48.997 ,18.609) |
| Absolute Humidity Lag 2 | -2.973 | (-34.399 ,45.804) |
| Total Precipitation Lag 1 | -25868.011 | (-76983.838 ,22668.468) |
| Total Precipitation Lag 2 | -13584.321 | (-63163.411 ,37037.177) |
| Relative Humidity Lag 1 | -25.998 | (-284.506 ,230.828) |
| Relative Humidity Lag 2 | 497.614 | (222.161 ,762.761) |
| Temperature Lag 1 | -332.032 | (-391.977 ,-284.154) |
| Temperature Lag 2 | -323.43 | (-397.958 ,-274.631) |

Table 274: Associations with monthly URTI case counts in Phangnga

| | Posterior Mean | 95% CrI |
|---|---|---|
| Case Count Lag 1 | -0.287 | (-0.396 ,-0.18) |
| Case Count Lag 2 | -0.799 | (-0.901 ,-0.689) |
| Absolute Humidity Lag 1 | -32.472 | (-94.651 ,-8.603) |
| Absolute Humidity Lag 2 | -25.601 | (-63.031 ,3.868) |
| Total Precipitation Lag 1 | 3826.587 | (-26713.287 ,35508.114) |
| Total Precipitation Lag 2 | -48106.373 | (-79988.656 ,-16300.581) |
| Relative Humidity Lag 1 | -117.444 | (-318.555 ,57.191) |
| Relative Humidity Lag 2 | 355.119 | (185.294 ,532.031) |
| Temperature Lag 1 | -447.831 | (-483.604 ,-354.593) |
| Temperature Lag 2 | -454.093 | (-497.471 ,-398.059) |

Table 275: Associations with monthly URTI case counts in Phatthalung

| | Posterior Mean | 95% CrI |
|---|---|---|
| Case Count Lag 1 | -0.35 | (-0.449 ,-0.251) |
| Case Count Lag 2 | -0.761 | (-0.86 ,-0.662) |
| Absolute Humidity Lag 1 | -36.007 | (-99.244 ,1.966) |
| Absolute Humidity Lag 2 | -12.235 | (-56.573 ,34.114) |
| Total Precipitation Lag 1 | -3919.426 | (-54234.766 ,47680.626) |
| Total Precipitation Lag 2 | 38800.95 | (-8766.284 ,87323.515) |
| Relative Humidity Lag 1 | -477.761 | (-907.053 ,-82.337) |
| Relative Humidity Lag 2 | 384.58 | (-9.134 ,778.95) |
| Temperature Lag 1 | -380.717 | (-440.356 ,-283.345) |
| Temperature Lag 2 | -364.874 | (-436.198 ,-295.921) |

Table 276: Associations with monthly URTI case counts in Phayao

| | Posterior Mean | 95% CrI |
|---|---|---|
| Case Count Lag 1 | -0.28 | (-0.339 ,-0.225) |
| Case Count Lag 2 | -0.536 | (-0.587 ,-0.482) |
| Absolute Humidity Lag 1 | 1.953 | (-15.091 ,33.632) |
| Absolute Humidity Lag 2 | -9.267 | (-29.158 ,4.037) |
| Total Precipitation Lag 1 | 119600.206 | (50749.526 ,192890.756) |
| Total Precipitation Lag 2 | -34601.137 | (-115066.332 ,43551.667) |
| Relative Humidity Lag 1 | -64.849 | (-287.61 ,151.271) |
| Relative Humidity Lag 2 | 111.822 | (-81.789 ,320.163) |
| Temperature Lag 1 | -138.452 | (-178.874 ,-117.189) |
| Temperature Lag 2 | -113.389 | (-130.369 ,-87.863) |

Table 277: Associations with monthly URTI case counts in Phetchabun

| | Posterior Mean | 95% CrI |
|---|---|---|
| Case Count Lag 1 | 0.512 | (0.338 ,0.685) |
| Case Count Lag 2 | -0.584 | (-0.749 ,-0.414) |
| Absolute Humidity Lag 1 | -14.182 | (-46.063 ,25) |
| Absolute Humidity Lag 2 | -7.228 | (-38.651 ,25.604) |
| Total Precipitation Lag 1 | 58076.62 | (-82909.999 ,201627.818) |
| Total Precipitation Lag 2 | -228885.954 | (-388399.679 ,-77112.686) |
| Relative Humidity Lag 1 | 148.511 | (-242.109 ,571.958) |
| Relative Humidity Lag 2 | 132.202 | (-260.342 ,534.929) |
| Temperature Lag 1 | -175.81 | (-229.624 ,-131.691) |
| Temperature Lag 2 | -160.419 | (-205.204 ,-115.948) |

Table 278: Associations with monthly URTI case counts in Phetchaburi

| | Posterior Mean | 95% CrI |
|---|---|---|
| Case Count Lag 1 | -0.178 | (-0.273 ,-0.085) |
| Case Count Lag 2 | -0.697 | (-0.793 ,-0.602) |
| Absolute Humidity Lag 1 | -16.673 | (-35.475 ,3.888) |
| Absolute Humidity Lag 2 | -4.301 | (-20.551 ,21.099) |
| Total Precipitation Lag 1 | -39238.859 | (-113248.021 ,31669.165) |
| Total Precipitation Lag 2 | -31702.171 | (-103795.778 ,35767.172) |
| Relative Humidity Lag 1 | 21.189 | (-159.603 ,215.155) |
| Relative Humidity Lag 2 | 35.501 | (-126.13 ,214.82) |
| Temperature Lag 1 | -238.458 | (-267.457 ,-212.009) |
| Temperature Lag 2 | -229.351 | (-264.348 ,-205.621) |

Table 279: Associations with monthly URTI case counts in Phichit

| | Posterior Mean | 95% CrI |
|---|---|---|
| Case Count Lag 1 | -0.262 | (-0.34 ,-0.185) |
| Case Count Lag 2 | -0.519 | (-0.591 ,-0.444) |
| Absolute Humidity Lag 1 | -9.074 | (-20.753 ,7.624) |
| Absolute Humidity Lag 2 | -10.47 | (-26.921 ,-0.651) |
| Total Precipitation Lag 1 | 32468.032 | (-19820.313 ,85622.967) |
| Total Precipitation Lag 2 | -41311.35 | (-98390.401 ,12468.378) |
| Relative Humidity Lag 1 | -12.851 | (-188.163 ,161.082) |
| Relative Humidity Lag 2 | -5.297 | (-161.507 ,154.192) |
| Temperature Lag 1 | -159.596 | (-184.388 ,-142.548) |
| Temperature Lag 2 | -143.374 | (-158.547 ,-118.775) |

Table 280: Associations with monthly URTI case counts in Phitsanulok

| | Posterior Mean | 95% CrI |
|---|---|---|
| Case Count Lag 1 | -0.399 | (-0.438 ,-0.359) |
| Case Count Lag 2 | -0.691 | (-0.732 ,-0.652) |
| Absolute Humidity Lag 1 | -10.316 | (-28.884 ,11.247) |
| Absolute Humidity Lag 2 | -7.209 | (-23.791 ,14.337) |
| Total Precipitation Lag 1 | 54729.683 | (-12585.922 ,126273.3) |
| Total Precipitation Lag 2 | -70203.166 | (-146771.984 ,3385.758) |
| Relative Humidity Lag 1 | 59.185 | (-173.605 ,318.374) |
| Relative Humidity Lag 2 | -8.304 | (-235.373 ,240.122) |
| Temperature Lag 1 | -151.03 | (-180.62 ,-126.287) |
| Temperature Lag 2 | -144.784 | (-173.904 ,-122.132) |

Table 281: Associations with monthly URTI case counts in Phra Nakhon Si Ayutthaya

| | Posterior Mean | 95% CrI |
|---|---|---|
| Case Count Lag 1 | -0.125 | (-0.233 ,-0.016) |
| Case Count Lag 2 | -0.846 | (-0.957 ,-0.741) |
| Absolute Humidity Lag 1 | -27.669 | (-67.943 ,5.572) |
| Absolute Humidity Lag 2 | -2.434 | (-38.947 ,36.757) |
| Total Precipitation Lag 1 | -192729.484 | (-346136.042 ,-39746.708) |
| Total Precipitation Lag 2 | -68707.418 | (-203441.142 ,63188.654) |
| Relative Humidity Lag 1 | 81.831 | (-243.915 ,410.436) |
| Relative Humidity Lag 2 | -112.206 | (-430.737 ,191.544) |
| Temperature Lag 1 | -208.274 | (-262.925 ,-144.23) |
| Temperature Lag 2 | -188.532 | (-249.443 ,-127.372) |

Table 282: Associations with monthly URTI case counts in Phrae

| | Posterior Mean | 95% CrI |
|---|---|---|
| Case Count Lag 1 | 0.245 | (0.129 ,0.359) |
| Case Count Lag 2 | -0.324 | (-0.438 ,-0.211) |
| Absolute Humidity Lag 1 | -3.169 | (-14.11 ,16.392) |
| Absolute Humidity Lag 2 | -11.576 | (-28.515 ,-1.95) |
| Total Precipitation Lag 1 | 71220.507 | (26367.339 ,116077.13) |
| Total Precipitation Lag 2 | -68692.544 | (-121257.612 ,-18773.115) |
| Relative Humidity Lag 1 | -61.138 | (-211.739 ,84.85) |
| Relative Humidity Lag 2 | 118.847 | (-27.653 ,260.532) |
| Temperature Lag 1 | -143.963 | (-169.773 ,-129.52) |
| Temperature Lag 2 | -123.004 | (-136.115 ,-100.679) |

Table 283: Associations with monthly URTI case counts in Phuket

| | Posterior Mean | 95% CrI |
|---|---|---|
| Case Count Lag 1 | -0.254 | (-0.335 ,-0.174) |
| Case Count Lag 2 | -0.721 | (-0.796 ,-0.641) |
| Absolute Humidity Lag 1 | -27.503 | (-73.216 ,32.093) |
| Absolute Humidity Lag 2 | -20.442 | (-65.3 ,40.622) |
| Total Precipitation Lag 1 | 36795.054 | (-6931.052 ,82950.814) |
| Total Precipitation Lag 2 | -17049.05 | (-61187.53 ,25038.58) |
| Relative Humidity Lag 1 | -385.401 | (-716.756 ,-62.674) |
| Relative Humidity Lag 2 | 494.227 | (206.787 ,773.31) |
| Temperature Lag 1 | -529.942 | (-620.353 ,-459.317) |
| Temperature Lag 2 | -525.909 | (-618.866 ,-456.908) |

Table 284: Associations with monthly URTI case counts in Prachin Buri

| | Posterior Mean | 95% CrI |
|---|---|---|
| Case Count Lag 1 | 0.512 | (0.324 ,0.707) |
| Case Count Lag 2 | -0.838 | (-1.032 ,-0.652) |
| Absolute Humidity Lag 1 | -14.041 | (-55.475 ,40.822) |
| Absolute Humidity Lag 2 | -8.234 | (-50.326 ,37.642) |
| Total Precipitation Lag 1 | -26228.405 | (-165379.054 ,107534.611) |
| Total Precipitation Lag 2 | -123603.435 | (-266108.293 ,7424.393) |
| Relative Humidity Lag 1 | 372.187 | (-56.673 ,824.794) |
| Relative Humidity Lag 2 | -31.534 | (-418.966 ,366.761) |
| Temperature Lag 1 | -243.242 | (-322.582 ,-184.269) |
| Temperature Lag 2 | -214.558 | (-280.433 ,-152.021) |

Table 285: Associations with monthly URTI case counts in Prachuap Khiri Khan

| | Posterior Mean | 95% CrI |
|---|---|---|
| Case Count Lag 1 | -0.063 | (-0.15 ,0.021) |
| Case Count Lag 2 | -0.599 | (-0.683 ,-0.509) |
| Absolute Humidity Lag 1 | -21.999 | (-51.082 ,7.051) |
| Absolute Humidity Lag 2 | -6.145 | (-32.354 ,26.996) |
| Total Precipitation Lag 1 | -71835.29 | (-152882.433 ,6119.282) |
| Total Precipitation Lag 2 | 16415.761 | (-52535.934 ,89699.469) |
| Relative Humidity Lag 1 | 8.72 | (-212.354 ,240.259) |
| Relative Humidity Lag 2 | -39.702 | (-239.8 ,154.465) |
| Temperature Lag 1 | -298.163 | (-343.282 ,-255.66) |
| Temperature Lag 2 | -283.773 | (-332.413 ,-245.73) |

Table 286: Associations with monthly URTI case counts in Ranong

| | Posterior Mean | 95% CrI |
|---|---|---|
| Case Count Lag 1 | 0.623 | (0.357 ,0.873) |
| Case Count Lag 2 | -0.414 | (-0.658 ,-0.148) |
| Absolute Humidity Lag 1 | -24.299 | (-40.466 ,-11.236) |
| Absolute Humidity Lag 2 | -21.18 | (-39.786 ,-8.808) |
| Total Precipitation Lag 1 | -2427.304 | (-21196.269 ,16147.154) |
| Total Precipitation Lag 2 | -2502.251 | (-21611.259 ,15558.585) |
| Relative Humidity Lag 1 | -49.569 | (-170.895 ,51.73) |
| Relative Humidity Lag 2 | 13.786 | (-82.502 ,110.402) |
| Temperature Lag 1 | -392.569 | (-412.823 ,-368.156) |
| Temperature Lag 2 | -384.114 | (-402.328 ,-356.186) |

Table 287: Associations with monthly URTI case counts in Ratchaburi

| | Posterior Mean | 95% CrI |
|---|---|---|
| Case Count Lag 1 | -0.482 | (-0.545 ,-0.42) |
| Case Count Lag 2 | -0.889 | (-0.95 ,-0.827) |
| Absolute Humidity Lag 1 | -36.048 | (-73.604 ,-11.151) |
| Absolute Humidity Lag 2 | -17.12 | (-50.419 ,1.692) |
| Total Precipitation Lag 1 | -67180.967 | (-172948.895 ,31504.621) |
| Total Precipitation Lag 2 | -66974.985 | (-163922.439 ,27200.365) |
| Relative Humidity Lag 1 | 0.53 | (-214.393 ,236.138) |
| Relative Humidity Lag 2 | -42.842 | (-270.44 ,170.695) |
| Temperature Lag 1 | -178.744 | (-216.143 ,-122.827) |
| Temperature Lag 2 | -181.845 | (-210.205 ,-133.721) |

Table 288: Associations with monthly URTI case counts in Rayong

| | Posterior Mean | 95% CrI |
|---|---|---|
| Case Count Lag 1 | -0.336 | (-0.375 ,-0.299) |
| Case Count Lag 2 | -0.689 | (-0.726 ,-0.65) |
| Absolute Humidity Lag 1 | -27.842 | (-77.546 ,16.595) |
| Absolute Humidity Lag 2 | 37.673 | (-16.752 ,129.937) |
| Total Precipitation Lag 1 | -98992.976 | (-181328.762 ,-17316.345) |
| Total Precipitation Lag 2 | 47771.017 | (-24334.794 ,122978.379) |
| Relative Humidity Lag 1 | 40.182 | (-229.062 ,314.328) |
| Relative Humidity Lag 2 | -163.717 | (-431.811 ,101.236) |
| Temperature Lag 1 | -316.837 | (-383.553 ,-243.34) |
| Temperature Lag 2 | -358.001 | (-493.164 ,-276.352) |

Table 289: Associations with monthly URTI case counts in Roi Et

| | Posterior Mean | 95% CrI |
|---|---|---|
| Case Count Lag 1 | 1.598 | (1.287 ,1.902) |
| Case Count Lag 2 | -0.459 | (-0.772 ,-0.146) |
| Absolute Humidity Lag 1 | -14.87 | (-51.18 ,20.759) |
| Absolute Humidity Lag 2 | 6.712 | (-23.076 ,58.121) |
| Total Precipitation Lag 1 | 191384.057 | (26393.003 ,355094.073) |
| Total Precipitation Lag 2 | -213711.862 | (-388540.906 ,-39378.121) |
| Relative Humidity Lag 1 | 23.071 | (-437.168 ,479.185) |
| Relative Humidity Lag 2 | -5.269 | (-465.241 ,448.552) |
| Temperature Lag 1 | -143.4 | (-195.012 ,-92.647) |
| Temperature Lag 2 | -141.393 | (-215.7 ,-99.098) |

Table 290: Associations with monthly URTI case counts in Sa Kaeo

| | Posterior Mean | 95% CrI |
|---|---|---|
| Case Count Lag 1 | -0.042 | (-0.165 ,0.077) |
| Case Count Lag 2 | -0.553 | (-0.669 ,-0.432) |
| Absolute Humidity Lag 1 | -16.508 | (-27.456 ,-9.28) |
| Absolute Humidity Lag 2 | -13.185 | (-23.365 ,-6.033) |
| Total Precipitation Lag 1 | -22606.249 | (-57709.756 ,10810.366) |
| Total Precipitation Lag 2 | -46120.678 | (-80311.047 ,-11153.921) |
| Relative Humidity Lag 1 | 77.528 | (-11.811 ,169.791) |
| Relative Humidity Lag 2 | -13.509 | (-94.492 ,62.253) |
| Temperature Lag 1 | -213.017 | (-223.81 ,-196.907) |
| Temperature Lag 2 | -208.186 | (-218.635 ,-192.489) |

Table 291: Associations with monthly URTI case counts in Sakon Nakhon

| | Posterior Mean | 95% CrI |
|---|---|---|
| Case Count Lag 1 | 0.888 | (0.646 ,1.138) |
| Case Count Lag 2 | -0.889 | (-1.138 ,-0.653) |
| Absolute Humidity Lag 1 | -12.485 | (-27.294 ,0.923) |
| Absolute Humidity Lag 2 | -6.332 | (-20.033 ,7.157) |
| Total Precipitation Lag 1 | 142801.403 | (72699.803 ,210847.325) |
| Total Precipitation Lag 2 | -46687.329 | (-121858.218 ,25926.953) |
| Relative Humidity Lag 1 | -131.149 | (-352.926 ,68.7) |
| Relative Humidity Lag 2 | -54.609 | (-243.917 ,133.236) |
| Temperature Lag 1 | -120.432 | (-138.858 ,-100.657) |
| Temperature Lag 2 | -114.729 | (-132.707 ,-96.136) |

Table 292: Associations with monthly URTI case counts in Samut Prakan

| | Posterior Mean | 95% CrI |
|---|---|---|
| Case Count Lag 1 | 0.18 | (0.097 ,0.266) |
| Case Count Lag 2 | -0.989 | (-1.074 ,-0.904) |
| Absolute Humidity Lag 1 | -3.329 | (-48.291 ,73.223) |
| Absolute Humidity Lag 2 | 13.782 | (-34.966 ,98.854) |
| Total Precipitation Lag 1 | -51353.25 | (-241977.691 ,122490.857) |
| Total Precipitation Lag 2 | 16483.773 | (-142380.588 ,178688.939) |
| Relative Humidity Lag 1 | 9.627 | (-440.571 ,508.479) |
| Relative Humidity Lag 2 | 176.744 | (-243.524 ,641.359) |
| Temperature Lag 1 | -251.21 | (-370.885 ,-181.422) |
| Temperature Lag 2 | -254.524 | (-390.622 ,-180.821) |

Table 293: Associations with monthly URTI case counts in Samut Sakhon

| | Posterior Mean | 95% CrI |
|---|---|---|
| Case Count Lag 1 | -0.188 | (-0.22 ,-0.157) |
| Case Count Lag 2 | -0.374 | (-0.406 ,-0.341) |
| Absolute Humidity Lag 1 | -16.878 | (-32.718 ,-7.448) |
| Absolute Humidity Lag 2 | -8.822 | (-19.642 ,7.617) |
| Total Precipitation Lag 1 | -64926.032 | (-111607.358 ,-17970.68) |
| Total Precipitation Lag 2 | 17866.398 | (-21056.325 ,59470.029) |
| Relative Humidity Lag 1 | 24.364 | (-87.615 ,138.868) |
| Relative Humidity Lag 2 | -82.55 | (-187.044 ,24.973) |
| Temperature Lag 1 | -218.705 | (-233.725 ,-194.321) |
| Temperature Lag 2 | -218.825 | (-244.243 ,-201.895) |

Table 294: Associations with monthly URTI case counts in Samut Songkhram

| | Posterior Mean | 95% CrI |
|---|---|---|
| Case Count Lag 1 | -0.533 | (-0.642 ,-0.428) |
| Case Count Lag 2 | -0.74 | (-0.848 ,-0.628) |
| Absolute Humidity Lag 1 | -14.869 | (-21.895 ,-9.829) |
| Absolute Humidity Lag 2 | -12.784 | (-20.048 ,-8.056) |
| Total Precipitation Lag 1 | -4905.533 | (-29807.452 ,20542.78) |
| Total Precipitation Lag 2 | -1087.808 | (-24010.779 ,23378.848) |
| Relative Humidity Lag 1 | -43.826 | (-106.193 ,15.867) |
| Relative Humidity Lag 2 | -17.522 | (-75.101 ,37.97) |
| Temperature Lag 1 | -210.413 | (-218.284 ,-199.644) |
| Temperature Lag 2 | -205.629 | (-212.835 ,-194.344) |

Table 295: Associations with monthly URTI case counts in Saraburi

| | Posterior Mean | 95% CrI |
|---|---|---|
| Case Count Lag 1 | 0.652 | (0.459 ,0.847) |
| Case Count Lag 2 | -0.961 | (-1.157 ,-0.766) |
| Absolute Humidity Lag 1 | -26.801 | (-71.693 ,-3.814) |
| Absolute Humidity Lag 2 | -7.565 | (-32.061 ,21.18) |
| Total Precipitation Lag 1 | -6094.963 | (-113559.92 ,99652.852) |
| Total Precipitation Lag 2 | -152764.43 | (-259669.487 ,-47754.32) |
| Relative Humidity Lag 1 | -4.739 | (-279.773 ,268.838) |
| Relative Humidity Lag 2 | 57.196 | (-193.631 ,323.712) |
| Temperature Lag 1 | -190.679 | (-225.302 ,-121.72) |
| Temperature Lag 2 | -197.709 | (-241.439 ,-159.184) |

Table 296: Associations with monthly URTI case counts in Satun

| | Posterior Mean | 95% CrI |
|---|---|---|
| Case Count Lag 1 | 0.547 | (0.263 ,0.828) |
| Case Count Lag 2 | -1.233 | (-1.51 ,-0.957) |
| Absolute Humidity Lag 1 | -26.268 | (-52.015 ,-2.829) |
| Absolute Humidity Lag 2 | -25.221 | (-54.301 ,-3.646) |
| Total Precipitation Lag 1 | 8517.62 | (-18849.222 ,38334.591) |
| Total Precipitation Lag 2 | -18480.51 | (-49086.576 ,10935.706) |
| Relative Humidity Lag 1 | -117.227 | (-304.878 ,51.006) |
| Relative Humidity Lag 2 | 258.021 | (104.799 ,417.335) |
| Temperature Lag 1 | -458.879 | (-493.855 ,-419.672) |
| Temperature Lag 2 | -454.831 | (-488.394 ,-408.68) |

Table 297: Associations with monthly URTI case counts in Si Sa Ket

| | Posterior Mean | 95% CrI |
|---|---|---|
| Case Count Lag 1 | 1.795 | (1.422 ,2.178) |
| Case Count Lag 2 | -0.623 | (-1.003 ,-0.233) |
| Absolute Humidity Lag 1 | -28.429 | (-99.644 ,35.181) |
| Absolute Humidity Lag 2 | 10.659 | (-40.381 ,97.484) |
| Total Precipitation Lag 1 | 75540.04 | (-163041.377 ,323092.926) |
| Total Precipitation Lag 2 | -411651.203 | (-673112.223 ,-160173.373) |
| Relative Humidity Lag 1 | 253.264 | (-493.046 ,1025.424) |
| Relative Humidity Lag 2 | 63.971 | (-580.439 ,761.563) |
| Temperature Lag 1 | -164.199 | (-257.069 ,-64.043) |
| Temperature Lag 2 | -168.813 | (-290.611 ,-94.779) |

Table 298: Associations with monthly URTI case counts in Sing Buri

| | Posterior Mean | 95% CrI |
|---|---|---|
| Case Count Lag 1 | 0.004 | (-0.131 ,0.134) |
| Case Count Lag 2 | -0.401 | (-0.527 ,-0.269) |
| Absolute Humidity Lag 1 | -14.974 | (-25.619 ,-9.438) |
| Absolute Humidity Lag 2 | -8.242 | (-13.147 ,0.352) |
| Total Precipitation Lag 1 | -5921.722 | (-34696.788 ,21782.092) |
| Total Precipitation Lag 2 | -20026.907 | (-49032.88 ,7643.833) |
| Relative Humidity Lag 1 | 6.2 | (-57.944 ,72.897) |
| Relative Humidity Lag 2 | -8.517 | (-66.014 ,49.698) |
| Temperature Lag 1 | -168.803 | (-177.743 ,-151.067) |
| Temperature Lag 2 | -176.529 | (-190.453 ,-168.685) |

Table 299: Associations with monthly URTI case counts in Songkhla

| | Posterior Mean | 95% CrI |
|---|---|---|
| Case Count Lag 1 | 0.117 | (-0.041 ,0.282) |
| Case Count Lag 2 | -0.702 | (-0.866 ,-0.536) |
| Absolute Humidity Lag 1 | -30.488 | (-125.275 ,57.093) |
| Absolute Humidity Lag 2 | 21.849 | (-56.324 ,163.503) |
| Total Precipitation Lag 1 | -92006.887 | (-214526.128 ,19507.063) |
| Total Precipitation Lag 2 | 15958.714 | (-99017.225 ,132098.854) |
| Relative Humidity Lag 1 | -44.404 | (-696.815 ,586.521) |
| Relative Humidity Lag 2 | 951.309 | (350.003 ,1575.946) |
| Temperature Lag 1 | -404.09 | (-543.793 ,-256.11) |
| Temperature Lag 2 | -402.19 | (-622.228 ,-278.646) |

Table 300: Associations with monthly URTI case counts in Sukhothai

| | Posterior Mean | 95% CrI |
|---|---|---|
| Case Count Lag 1 | -0.367 | (-0.412 ,-0.324) |
| Case Count Lag 2 | -0.576 | (-0.617 ,-0.536) |
| Absolute Humidity Lag 1 | -10.6 | (-20.955 ,-2.146) |
| Absolute Humidity Lag 2 | -8.031 | (-16.567 ,0.872) |
| Total Precipitation Lag 1 | 52301.185 | (543.545 ,106174.652) |
| Total Precipitation Lag 2 | -26446.303 | (-80695.025 ,26977.507) |
| Relative Humidity Lag 1 | -22.3 | (-164.571 ,103.101) |
| Relative Humidity Lag 2 | 1.032 | (-130.171 ,130.792) |
| Temperature Lag 1 | -129.433 | (-141.802 ,-114.772) |
| Temperature Lag 2 | -127.123 | (-140.545 ,-114.073) |

Table 301: Associations with monthly URTI case counts in Suphan Buri

| | Posterior Mean | 95% CrI |
|---|---|---|
| Case Count Lag 1 | -0.076 | (-0.177 ,0.026) |
| Case Count Lag 2 | -0.697 | (-0.795 ,-0.606) |
| Absolute Humidity Lag 1 | -16.848 | (-36.861 ,-0.484) |
| Absolute Humidity Lag 2 | -8.869 | (-26.66 ,8.263) |
| Total Precipitation Lag 1 | -83417.333 | (-190461.313 ,13367.076) |
| Total Precipitation Lag 2 | -31677.084 | (-129734.414 ,60447.738) |
| Relative Humidity Lag 1 | 28.001 | (-176.571 ,238.98) |
| Relative Humidity Lag 2 | -60.751 | (-252.2 ,124.315) |
| Temperature Lag 1 | -174.169 | (-200.075 ,-143.312) |
| Temperature Lag 2 | -164.539 | (-191.14 ,-136.98) |

Table 302: Associations with monthly URTI case counts in Surat Thani

| | Posterior Mean | 95% CrI |
|---|---|---|
| Case Count Lag 1 | -0.349 | (-0.441 ,-0.263) |
| Case Count Lag 2 | -0.899 | (-0.985 ,-0.811) |
| Absolute Humidity Lag 1 | -23.577 | (-94.889 ,74.744) |
| Absolute Humidity Lag 2 | 11.26 | (-65.117 ,105.987) |
| Total Precipitation Lag 1 | -172131.817 | (-284994.331 ,-56114.429) |
| Total Precipitation Lag 2 | 31866.343 | (-78279.637 ,142246.982) |
| Relative Humidity Lag 1 | -219.082 | (-933.787 ,446.665) |
| Relative Humidity Lag 2 | 577.385 | (-41.202 ,1236.551) |
| Temperature Lag 1 | -397.298 | (-551.963 ,-287.561) |
| Temperature Lag 2 | -327.937 | (-467.477 ,-210.741) |

Table 303: Associations with monthly URTI case counts in Surin

| | Posterior Mean | 95% CrI |
|---|---|---|
| Case Count Lag 1 | -0.028 | (-0.127 ,0.072) |
| Case Count Lag 2 | -0.62 | (-0.719 ,-0.524) |
| Absolute Humidity Lag 1 | -18.828 | (-66.009 ,27.286) |
| Absolute Humidity Lag 2 | -8.777 | (-60.11 ,34.579) |
| Total Precipitation Lag 1 | 157360.322 | (-55867.222 ,373082.6) |
| Total Precipitation Lag 2 | -429688.788 | (-656981.816 ,-205242.911) |
| Relative Humidity Lag 1 | 313.381 | (-293.989 ,943.729) |
| Relative Humidity Lag 2 | 24.927 | (-536.438 ,579.514) |
| Temperature Lag 1 | -159.282 | (-226.463 ,-91.763) |
| Temperature Lag 2 | -138.389 | (-200.573 ,-62.365) |

Table 304: Associations with monthly URTI case counts in Tak

| | Posterior Mean | 95% CrI |
|---|---|---|
| Case Count Lag 1 | 0.176 | (0.022 ,0.332) |
| Case Count Lag 2 | -0.633 | (-0.786 ,-0.479) |
| Absolute Humidity Lag 1 | -9.461 | (-32.524 ,23.35) |
| Absolute Humidity Lag 2 | -13.186 | (-41.343 ,7.994) |
| Total Precipitation Lag 1 | 143550.063 | (64850.064 ,224897.734) |
| Total Precipitation Lag 2 | 41348.842 | (-50202.385 ,137089.044) |
| Relative Humidity Lag 1 | -157.635 | (-427.844 ,95.19) |
| Relative Humidity Lag 2 | -127.337 | (-393.868 ,115.747) |
| Temperature Lag 1 | -173.572 | (-213.949 ,-145.29) |
| Temperature Lag 2 | -161.705 | (-188.636 ,-127.587) |

Table 305: Associations with monthly URTI case counts in Trang

| | Posterior Mean | 95% CrI |
|---|---|---|
| Case Count Lag 1 | -0.403 | (-0.495 ,-0.315) |
| Case Count Lag 2 | -1.042 | (-1.137 ,-0.952) |
| Absolute Humidity Lag 1 | -39.631 | (-109.625 ,0.935) |
| Absolute Humidity Lag 2 | -2.624 | (-47.818 ,61.616) |
| Total Precipitation Lag 1 | 915.017 | (-66005.879 ,66563.989) |
| Total Precipitation Lag 2 | -42645.426 | (-111990.321 ,21422.757) |
| Relative Humidity Lag 1 | -379.586 | (-793.507 ,14.119) |
| Relative Humidity Lag 2 | 650.69 | (293.424 ,1007.014) |
| Temperature Lag 1 | -435.947 | (-501.465 ,-327.027) |
| Temperature Lag 2 | -439.582 | (-539.926 ,-369.295) |

Table 306: Associations with monthly URTI case counts in Trat

| | Posterior Mean | 95% CrI |
|---|---|---|
| Case Count Lag 1 | -0.14 | (-0.23 ,-0.044) |
| Case Count Lag 2 | -0.645 | (-0.736 ,-0.556) |
| Absolute Humidity Lag 1 | -24.281 | (-44.758 ,-4.186) |
| Absolute Humidity Lag 2 | -19.94 | (-37.943 ,5.969) |
| Total Precipitation Lag 1 | 9069.976 | (-12343.715 ,33743.119) |
| Total Precipitation Lag 2 | 4375.438 | (-16507.598 ,27477.925) |
| Relative Humidity Lag 1 | -64.196 | (-181.255 ,39.532) |
| Relative Humidity Lag 2 | -38.126 | (-147.387 ,62.721) |
| Temperature Lag 1 | -387.887 | (-417.129 ,-357.963) |
| Temperature Lag 2 | -387.558 | (-424.254 ,-361.158) |

Table 307: Associations with monthly URTI case counts in Ubon Ratchathani

| | Posterior Mean | 95% CrI |
|---|---|---|
| Case Count Lag 1 | 0.092 | (-0.008 ,0.189) |
| Case Count Lag 2 | -0.705 | (-0.802 ,-0.605) |
| Absolute Humidity Lag 1 | -4.485 | (-103.99 ,144.091) |
| Absolute Humidity Lag 2 | 23.758 | (-66.982 ,185.935) |
| Total Precipitation Lag 1 | 630711.981 | (210805.771 ,1067455.904) |
| Total Precipitation Lag 2 | -572099.604 | (-1041688.346 ,-107304.241) |
| Relative Humidity Lag 1 | -606.49 | (-2095.162 ,729.573) |
| Relative Humidity Lag 2 | 682.845 | (-495.453 ,1978.431) |
| Temperature Lag 1 | -204.431 | (-415.493 ,-65.414) |
| Temperature Lag 2 | -187.313 | (-407.834 ,-57.053) |

Table 308: Associations with monthly URTI case counts in Udon Thani

| | Posterior Mean | 95% CrI |
|---|---|---|
| Case Count Lag 1 | 0.261 | (0.126 ,0.394) |
| Case Count Lag 2 | -0.573 | (-0.705 ,-0.447) |
| Absolute Humidity Lag 1 | -7.339 | (-32.388 ,25.628) |
| Absolute Humidity Lag 2 | -4.964 | (-29.288 ,21.577) |
| Total Precipitation Lag 1 | 258188.143 | (97799.082 ,408572.688) |
| Total Precipitation Lag 2 | -232950.417 | (-399510.394 ,-63284.028) |
| Relative Humidity Lag 1 | -41.415 | (-453.108 ,374.493) |
| Relative Humidity Lag 2 | 139.918 | (-225.04 ,536.411) |
| Temperature Lag 1 | -134.035 | (-179.441 ,-99.764) |
| Temperature Lag 2 | -116.461 | (-152.4 ,-82.387) |

Table 309: Associations with monthly URTI case counts in Uthai Thani

| | Posterior Mean | 95% CrI |
|---|---|---|
| Case Count Lag 1 | -0.049 | (-0.178 ,0.079) |
| Case Count Lag 2 | -0.691 | (-0.818 ,-0.564) |
| Absolute Humidity Lag 1 | -14.621 | (-29.22 ,-6.024) |
| Absolute Humidity Lag 2 | -7.993 | (-16.772 ,2.673) |
| Total Precipitation Lag 1 | -311.981 | (-43849.409 ,42054.879) |
| Total Precipitation Lag 2 | -87978.797 | (-136974.038 ,-40903.801) |
| Relative Humidity Lag 1 | 35.266 | (-84.391 ,160.058) |
| Relative Humidity Lag 2 | 36.917 | (-78.362 ,165.716) |
| Temperature Lag 1 | -148.783 | (-160.104 ,-129.063) |
| Temperature Lag 2 | -150.15 | (-164.629 ,-138.021) |

Table 310: Associations with monthly URTI case counts in Uttaradit

| | Posterior Mean | 95% CrI |
|---|---|---|
| Case Count Lag 1 | -0.301 | (-0.371 ,-0.23) |
| Case Count Lag 2 | -0.677 | (-0.744 ,-0.609) |
| Absolute Humidity Lag 1 | -11.928 | (-30.324 ,7.705) |
| Absolute Humidity Lag 2 | -8.361 | (-26.596 ,7.482) |
| Total Precipitation Lag 1 | 95387.573 | (12472.342 ,173670.382) |
| Total Precipitation Lag 2 | -80284.267 | (-166681.384 ,5757.164) |
| Relative Humidity Lag 1 | 50.298 | (-197.218 ,301.961) |
| Relative Humidity Lag 2 | -47.001 | (-301.209 ,169.947) |
| Temperature Lag 1 | -139.204 | (-165.05 ,-115.037) |
| Temperature Lag 2 | -132.717 | (-154.159 ,-107.749) |

Table 311: Associations with monthly URTI case counts in Yala

| | Posterior Mean | 95% CrI |
|---|---|---|
| Case Count Lag 1 | 3.024 | (2.475 ,3.598) |
| Case Count Lag 2 | -1.393 | (-1.952 ,-0.849) |
| Absolute Humidity Lag 1 | -27.245 | (-91.284 ,15.401) |
| Absolute Humidity Lag 2 | -18.747 | (-85.802 ,24.249) |
| Total Precipitation Lag 1 | -91543.313 | (-182845.975 ,-2851.371) |
| Total Precipitation Lag 2 | 36877.672 | (-46399.14 ,126138.717) |
| Relative Humidity Lag 1 | 38.032 | (-388.954 ,508.751) |
| Relative Humidity Lag 2 | 228.31 | (-191.85 ,661.269) |
| Temperature Lag 1 | -332.674 | (-396.927 ,-238.841) |
| Temperature Lag 2 | -293.776 | (-358.437 ,-194.718) |

Table 312: Associations with monthly URTI case counts in Yasothon

| | Posterior Mean | 95% CrI |
|---|---|---|
| Case Count Lag 1 | 0.631 | (0.414 ,0.832) |
| Case Count Lag 2 | -0.543 | (-0.756 ,-0.327) |
| Absolute Humidity Lag 1 | -10.317 | (-29.542 ,9.684) |
| Absolute Humidity Lag 2 | -6.043 | (-22.185 ,17.3) |
| Total Precipitation Lag 1 | 114334.974 | (25163.832 ,199433.646) |
| Total Precipitation Lag 2 | -96228.786 | (-187632.738 ,-6639.421) |
| Relative Humidity Lag 1 | 71.491 | (-193.094 ,333.921) |
| Relative Humidity Lag 2 | -74.485 | (-316.958 ,167.109) |
| Temperature Lag 1 | -140.624 | (-168.194 ,-113.589) |
| Temperature Lag 2 | -136.943 | (-169.958 ,-114.582) |

# References


Ghysels, E., Sinko, A., & Valkanov, R. (2007). Midas regressions: Further results and new directions. *Econometric reviews*, *26*(1), 53–90.

Park, T., & Casella, G. (2008). The bayesian lasso. *Journal of the American Statistical Association*, *103*(482), 681–686.

Ghysels, E. (2016). Macroeconomics and the reality of mixed frequency data. *Journal of Econometrics*, *193*(2), 294–314.
